# Forecasting distributions of an aquatic invasive species (*Nitellopsis obtusa*) under future climate scenarios

**DOI:** 10.1371/journal.pone.0180930

**Published:** 2017-07-13

**Authors:** Daniel Romero-Alvarez, Luis E. Escobar, Sara Varela, Daniel J. Larkin, Nicholas B. D. Phelps

**Affiliations:** 1 Hospital General Enrique Garcés, Unidad de Epidemiología, Quito, Ecuador; 2 Minnesota Aquatic Invasive Species Research Center, Department of Fisheries, Wildlife, and Conservation Biology, University of Minnesota, St. Paul, MN, United States of America; 3 Museum für Naturkunde, Leibniz Institute for Evolution and Biodiversity Science, Invalidenstraße 43, Berlin, Germany; National Cheng Kung University, TAIWAN

## Abstract

Starry stonewort (*Nitellopsis obtusa*) is an alga that has emerged as an aquatic invasive species of concern in the United States. Where established, starry stonewort can interfere with recreational uses of water bodies and potentially have ecological impacts. Incipient invasion of starry stonewort in Minnesota provides an opportunity to predict future expansion in order to target early detection and strategic management. We used ecological niche models to identify suitable areas for starry stonewort in Minnesota based on global occurrence records and present-day and future climate conditions. We assessed sensitivity of forecasts to different parameters, using four emission scenarios (i.e., RCP 2.6, RCP 4.5, RCP 6, and RCP 8.5) from five future climate models (i.e., CCSM, GISS, IPSL, MIROC, and MRI). From our niche model analyses, we found that (i) occurrences from the entire range, instead of occurrences restricted to the invaded range, provide more informed models; (ii) default settings in Maxent did not provide the best model; (iii) the model calibration area and its background samples impact model performance; (iv) model projections to future climate conditions should be restricted to analogous environments; and (v) forecasts in future climate conditions should include different future climate models and model calibration areas to better capture uncertainty in forecasts. Under present climate, the most suitable areas for starry stonewort are predicted to be found in central and southeastern Minnesota. In the future, suitable areas for starry stonewort are predicted to shift in geographic range under some future climate models and to shrink under others, with most permutations indicating a net decrease of the species’ suitable range. Our suitability maps can serve to design short-term plans for surveillance and education, while future climate models suggest a plausible reduction of starry stonewort spread in the long-term if the trends in climate warming remain.

## Introduction

Starry stonewort (*Nitellopsis obtusa*, Characeae) is a species of concern for both its endangered status (in parts of its native range in Europe and Asia) and its invasive status (in North America). The ‘starry’ of its common name comes from its characteristic star-shaped bulbils, starchy reproductive structures that enable spread via asexual reproduction [1]. In North America, female individuals of this species have not been detected to date [2]. It has a higher ecological plasticity than other charophytes [1,3]. For example, starry stonewort can flourish in hard-water (i.e., water with high mineral content) and habitats of varying depth, light availability, and sediment characteristics [4]. In addition, starry stonewort can grow densely, which may lead to displacement of native aquatic plant species and could have consequences for habitat quality [2]. Dense growth may also impair recreational activities such as swimming, fishing, and boating [1,3]. Although populations of starry stonewort in their native distribution in Europe and Japan have been declining [5–7], the species has shown great capacity to spread as an aquatic invasive species in North America [3,8,9].

In 1978, starry stonewort was first recorded in North America in the St. Lawrence River, where it was likely introduced through ballast water discharge from trans-Atlantic shipping [10]. Marine currents could have played a role in starry stonewort’s dispersion, but this has been not explored. Five years later, starry stonewort was reported for the first time in Michigan, United States [1,10]. To date, starry stonewort has been reported in Indiana, New York, Pennsylvania, Wisconsin, Vermont, Ontario, and, in August 2015, in Minnesota [3,8,11,12]. The introduction of starry stonewort to inland lakes has been speculated to be associated with recreational boat activities from the movement of bulbils and alga fragments between different lakes [1,3].

In light of limited knowledge about the potential spread and impacts of starry stonewort in the Americas, improved knowledge of the species’ invasion ecology is a priority. Among other efforts, identifying areas on the leading edge of the invasion range (e.g., Minnesota) with suitable conditions for starry stonewort is a priority for targeting surveillance and control. Ecological niche modeling can support these efforts. Ecological niche models correlate environmental conditions with species’ occurrence records to identify suitable habitats where a species can persist and increase in population size without the need of further immigration [13]. This methodology has been used successfully with different taxa, scales, and ecosystems [13–15]. Furthermore, ecological niche models can be applied to forecast probable distributions of species over longer time periods, e.g., under future climate scenarios [16–20]. Predicting areas where starry stonewort could establish could inform surveillance efforts for early detection, raise local awareness, and prioritize allocation of resources for control [21].

Local conditions can influence occurrence of starry stonewort in North America. For example, in Lake Ontario, starry stonewort’s distribution is associated with high conductivity, short distances to marinas, and low fetch [3]. In New York, Sleith et al. [1] found high pH and conductivity to be associated with starry stonewort. However, invasive species’ occurrences are defined not only by local-scale characteristics, but also by larger scales of environmental factors that promote or limit spread over space and time [22]. Invasion of starry stonewort in the Americas is likely an ongoing process that has not reached equilibrium, and more water bodies are likely to be affected [8].

**Fig 1:**
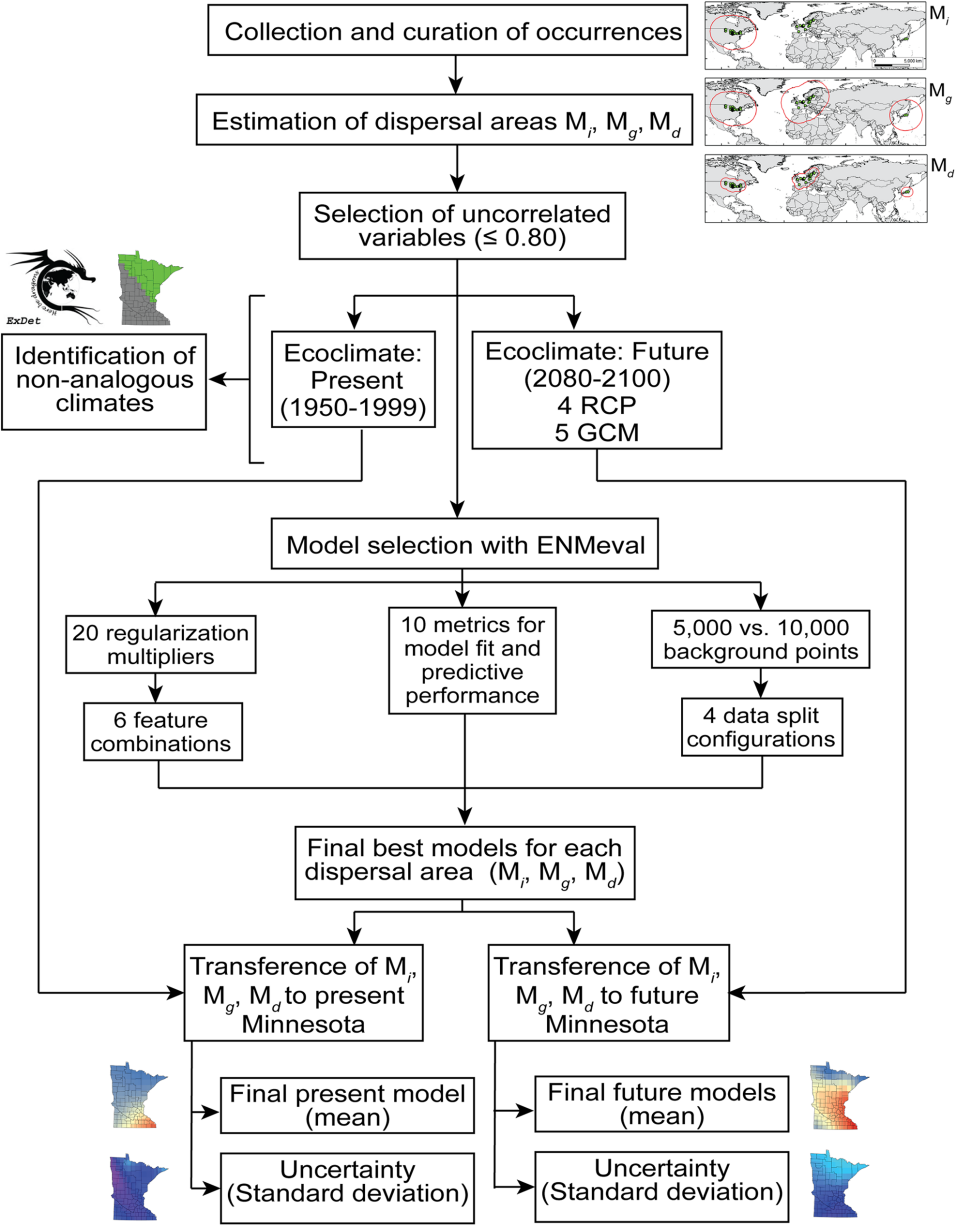
Workflow of the modeling process used in this study. Occurrences were collected, cleaned, and employed to estimate three model calibration regions (i.e., **M**_***i***_, **M**_***g***_, and **M**_***d***_). Present-day climatic variables were restricted to these model calibration regions and compared to future climatic conditions in Minnesota. Models were parametrized using present-day climates in the three model calibration regions and the best models were projected to future climates in Minnesota using five climate models and four RCP scenarios.

Recent reports of starry stonewort in Minnesota provide an opportunity to explore climatic factors that may influence future expansion. Here, we have constructed a series of ecological niche models to answer three main questions: (i) Which areas are vulnerable to starry stonewort invasion in Minnesota under present-day climate conditions? (ii) Which areas in Minnesota have suitable conditions for starry stonewort under future climate scenarios?, and (iii) How do decisions regarding the geographic region used in model calibration influence predictions? We propose a protocol (Fig 1) to improve the workflow of ecological niche models for forecasting species invasions.

## Methods

The ecological niche modeling approach employed was based on the **BAM** framework [23], which summarizes three components to define a species’ spatial range. The first component is **B**, the presence of other organisms that promote (e.g., prey, symbionts) or restrict (e.g., depredators, parasites) the distribution of the species in a region. The second component corresponds to the set of abiotic environmental conditions, **A**, e.g., temperature, that are suitable for a species to persist without need of immigration. The final component, **M**, corresponds to the ability of the species to colonize biotically (**B**) and abiotically (**A**) suitable regions. Thus, the spatial distribution of a species is defined as **B ∩ A ∩ M** [23]. We focused on a broad-scale exploration of **A** and **M**, as a preliminary assessment of the invasion potential of starry stonewort in terms of abiotic suitability and dispersal potential. We estimated **A** based on the association of starry stonewort occurrences with bioclimatic variables across its range, and estimated **M** based on using three regions for model calibration (Fig 1).

### Occurrences

Occurrence records of starry stonewort were published in Escobar et al. [8], which used data from digital repositories including the Global Biodiversity Information Facility (GBIF) [24] and the Global Invasive Species Information Network [25] using the keywords “*Nitellopsis obtusa*,” “*Nitellopsis obtusa* var. *ulvoides*,” and “*Chara obtusa*”. Occurrences from invaded areas in the US were also derived from additional reports and publications [1,4,9,26]. Minnesota records were updated based on 2016 reports of new localities from the Minnesota Department of Natural Resources (MDNR, http://www.dnr.state.mn.us/invasives/ais/infested.html).

Occurrences were individually inspected to assure credibility and geospatial accuracy. All Minnesota, Wisconsin, and New York records have been confirmed by a Characeae expert (Ken Karol, New York Botanical Garden). Michigan has the most records and not all have been verified by experts. It is possible that reports from Michigan (and GBIF or other databases) include false records. Unfortunately, this is the best information that is available at this time. We chose to include all records based on the expectation that the error rate is relatively low and that the invaded region most likely to include false records (Michigan) is in the center of the species’ invaded range, such that false occurrences would be unlikely to have a strong influence on niche estimation.

**Fig 2:**
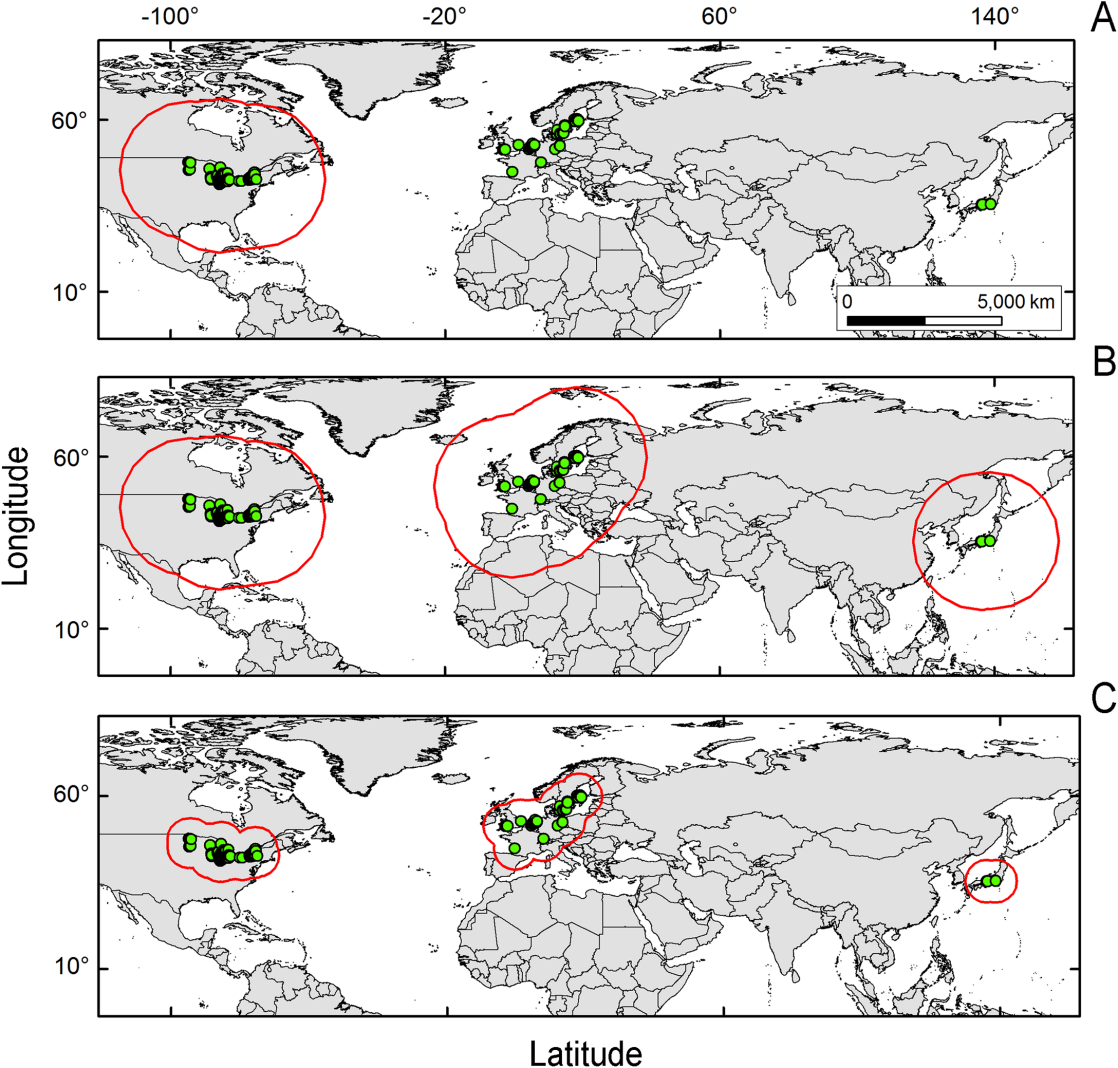
Model calibration region, M, explored in this study. Models were calibrated in three regions (red lines in A, B, and C) based on the distribution of starry stonewort populations (green points). **A.** Model calibration region based on an invasive population approach focused on starry stonewort populations in the invaded area of the United States and a high dispersal potential (i.e., 2,200 km), **M**_***i***_. **B.** Model calibration region considering the entire or global species’ range in the United States, Europe, and Japan and a high dispersal potential (i.e., 2,200 km), **M**_***g***_. **C.** Model calibration region considering the entire or global species’ range in the United States (left map), Europe (central map), and Japan (right map) and a reduced dispersal potential (i.e., 700 km), **M**_***d***_.

Oversampled areas, as a form of sampling bias, can generate model overfit [27]. To prevent this, we calibrated present-day models using occurrences filtered to one-per-cell according to the spatial resolution of cells in our environmental layers [28]. All the remaining occurrences were used for modeling. From the initial pool of 2,260 occurrences, 84 single occurrences (i.e., occupied pixel cells) remained in the entire species’ range: 29 in the native range (34.5%; 2 in Japan, 27 in Europe) and 55 in the invaded range in the US (65.5%; Fig 2).

### Model calibration region M

The selection of **M**, the model calibration region, has a strong influence on ecological niche model predictions [29]. For instance, considering only invasive populations can result in incomplete information about the environmental preferences of the species [13], or be insufficient to characterize environmental tolerances [30]. Explicitly testing different extents of the calibration region facilitates comparison of models and informs interpretation of results [31]. Recent new records for starry stonewort in North America suggest that it may be expanding in North America from east to west and from south to north [8]. As a proxy of the dispersal potential of the species we used two distances for three **M** scenarios. First, we used the maximum distance between all known starry stonewort populations in the US (~2,200 km), as suggested by the data available (i.e., MDNR surveillance: http://www.dnr.state.mn.us/invasives/ais/infested.html) [23]. Considering that the species has been dispersing between distant lakes, we assumed that spatial barriers could be overcome in the model calibration regions. We used this distance as a buffer around starry stonewort occurrences to generate a model calibration region for the invaded range in the US (**M**_***i***_). This area corresponds to a model based on the invasive populations.

Furthermore, to account for starry stonewort environmental preferences across its entire range, we focused on two additional model calibration areas, including both native (Europe and Japan) and invasive populations (US). One of these calibration areas was based on the same maximum distance between all known starry stonewort populations in the US (~2,200 km; **M**_***g***_) and the other was a proxy of the maximum distance between closer neighbors populations in the US (~700 km; **M**_***d***_), which in our case corresponded to the distance between the last detection in Wisconsin and the first detection in Minnesota. We used these distances to generate a buffer around occurrences across the entire species’ range (Fig 2). The **M**_***i***_ scenario encompasses inland and coastal regions of central and eastern Canada and all states in the continental US except those in the far west: California, Nevada, Oregon, Washington, and western portions of Arizona and Idaho. The **M**_***g***_ scenario encompasses all of those areas in addition to Europe, parts of northwestern Africa and Asia (Japan, North and South Korea, and parts of eastern China and Russia). The **M**_***d***_ scenario includes the Upper Midwest region in the US and southeastern Canada, portions of Southern, Northern, and Western Europe, and a small portion of Eastern Europe, and also Japan except by the Hokkaido island (Fig 2). All **M** scenarios included the area of interest for this study (Minnesota).

### Environmental variables

As a proxy of **A**, we used the present-day Ecoclimate dataset (1950–1999) at 50-km spatial resolution [32]. Since starry stonewort occurs in both coastal and inland areas, we used climate variables covering both regions. This climate dataset is derived from the Coupled Model Inter-comparison Project (CMIP5) and combines climatic patterns from multiple general circulation models from inland and marine ecosystems; thus, final climatic layers have global coverage. The role of oceanic dispersal in the invasion process of this species remains uncertain, however, we assumed that marine dispersal could play a role and include climate conditions in terrestrial and marine ecosytems in our model calibration regions. We used climatic variables likely to influence starry stonewort’s macroscale distribution, selecting uncorrelated variables based on correlation coefficients ≤0.80 (Table A in S1 File). Specifically, we used annual mean temperature (°C), mean diurnal temperature range (°C), isothermality (%), temperature seasonality (°C), maximum temperature of the warmest month (°C), mean temperature of the wettest quarter (°C), annual precipitation (mm/m^2^), and precipitation seasonality (%) [32].

Climate models are considerably variable, thus, adding more scenarios of future climate would provide more information regarding the plausible variability in forecasts. Future climatic conditions for the end of the 21^st^ century (2080–2100) were obtained from Ecoclimate, including four representative concentration pathways (RCPs; i.e., 2.6, 4.5, 6, and 8.5 W/m^2^; here after numbers are shown without units) [32]. Each RCP scenario represents potential trajectories of greenhouse gas emissions projected to the future, ranging from the most optimistic (i.e., 2.6) to the worst-case scenario (i.e., 8.5) [32]. RCPs are the most updated climate scenarios from the Intergovernmental Panel on Climate Change (IPCC), Fifth Assessment Report (AR5), and replaced the SRES scenarios previously implemented by the IPCC AR4 [33]. The four RCP scenarios were estimated based on five different future general circulation models (GCM): CCSM, GISS, IPSL, MIROC, and MRI, allowing us to capture the variability in emissions (i.e., RCP scenarios) and climate simulations (e.g., CCSM vs. MRI).

### Non-analogous climate evaluation

We explored areas with non-analogous (novel) climatic conditions between present-day climate in the calibration regions vs. future climate in the projection region of Minnesota. This resulted in a present vs. future comparison and calibration vs. projection regions. This analysis was done using the extrapolation detection (Exdet) tool developed by Mesgaran et al. [34]. Exdet identifies non-analogous environments between calibration and projection regions denoted as type I novelty [*sensu* 34]. Accounting for these non-analogous or novel environments enables a more confident interpretation of models [18,35,36].

### Ecological niche models

Qiao et al. [37] proposed that multiple ecological niche modeling algorithms should be employed to identify the model that best fits with the available data, the study system, and the research question. We used Maxent to perform niche modeling because it enables the use of different variable transformations (features), i.e., linear (L), quadratic (Q), product (P), threshold (T), and hinge (H), and allows for different parameterizations (regularization values). In addition, Maxent allows automatic truncation in novel climates to avoid predictions in non-analogous environments.

Maxent is an occurrences-background algorithm, which estimates the most uniform probable distribution of the occurrences across a selected calibration region [13,38]. The background represents the summary of environmental conditions across the model calibration region. Because we explored two calibration regions (invaded range and two areas from the entire species’ range) the available background varied. We developed models based on 5,000 and also 10,000 background samples.

Here, we tested 20 different regularization coefficient values ranging from 0.1 to 2. The regularization coefficients regulate the complexity of the model, higher values penalize for complexity and thus, produce simpler models (avoiding complex relationships between the data and the variables) that, in general, tend to have larger predictions [39]. Because assessing different configurations is recommended [39–41], we explored models based on six feature combinations reported in the literature: L, LQ, H, LQH, LQHP, and LQHPT [40].

We used raw values from Maxent to assess model fit according to Akaike’s Information Criterion values corrected for small sample size (AICc), which ranks models based on their information content and complexity [42]; the model with the lowest AICc was selected (i.e, ΔAICc = 0) as best reconciling the goals of fitting occurrences with environmental input data and minimizing model complexity [41]. In addition, because low AICc does not represent the ability of the model to predict independent data, we also assessed predictive performance based on the full (AUC_total_) and mean (AUC_mean_) of the area under the curve of the receiver-operating characteristic (AUC) and the difference between training and testing AUC and its variability. These metrics assess if models can discriminate between occurrence and background points, with AUC values ≤0.5 consistent with randomly generated models unable to differentiate between backgrounds and occurrences. Because AUC has been questioned [43,44], we also used independent data to calculated mean omission rates (OR) from binary models based on using 100% (OR_100%_) and 90% (OR_90%_) of training occurrences as thresholds. These metrics enable the proportion of independent occurrences predicted incorrectly to be quantified [40]. Evaluation of model predictions was performed using independent data obtained via dividing the occurrences in two sets, one for model calibration and one for evaluation. Calibration and evaluation data sets were developed based on four different data splitting configurations: (i) using one point at a time for model evaluation (i.e., Jackknife); (ii) apportioning the occurrences into four groups, each with an off-diagonal set for calibration and another for evaluation (i.e., block; as in [45]); (iii) selecting clusters of points and using half for calibration and the other half for evaluation (i.e., Checkerboard1 [40]), and (iv) partitioning the occurrences via cross-validation (*k*-fold; see [40]). Model evaluations were conducted using the R package ENMeval [40].

### Model projection to Minnesota

Once the best regularization coefficient, feature configuration, and number of background points were determined for the calibration regions (Fig 2), the three selected models were projected to environmental conditions in Minnesota. Maxent allows strict model transference during model projection via ‘extrapolation’ and ‘clamping’ being deactivated [36,46]. This practice prevents unrealistic extrapolations of models into non-analogous (novel) environments that could be present in the projection region but absent from the calibration region [46].

In all, to identify the best model by calibration region (**M**_***i***_ vs. **M**_***g***_ vs. **M**_***d***_), we explored 120 parameter configurations (20 regularization coefficients × 6 feature combinations), and two background samples for each regions **M**_***i***_ and **M**_***g***_: 5,000 and 10,000; and 10,000 for **M**_***d***_ which was not explored due to the reduced extent of this calibration area (Table B in S1 File). The best models were projected to 20 future climate scenarios (4 RCP × 5 climate models). To inform interpretation of forecasts, we also estimated uncertainty of all final models. We parameterized final models based on our previous evaluations and generated surfaces of uncertainty using 80% of occurrences in Maxent and performed 25 bootstrap replications using random starting seeds. For final models, we selected the logistic output format in Maxent with clamping and extrapolation deactivated. We used the standard deviation of replicates as an indicator of uncertainty [38,47] (Fig 1) and developed a *t*-test (*α* = 0.05) to compare the continuous suitability values of pixels among models in Minnesota.

Finally, we created an ensemble of models for different future climate scenarios in Minnesota. We averaged the final logistic models and calculated the standard deviations to identify areas where models were consistent (low SD) or diverged (high SD). There is debate about use of model ensembles, due to issues regarding interpretation of continuous units from different algorithms (e.g., general linear models vs. regression trees vs. Maxent) (see [13]). Here, we overcame such discrepancies by using the same suitability value (i.e., Maxent logistic), from the same parameterization so that differences only reflected differences in future climate models for Minnesota. We also estimated the number of lakes in Minnesota comprising the lowest and highest predictions of suitability using lake inventory data from the National Wetlands Inventory of the US Fish & Wildlife Service [48].

## Results

**Fig 3:**
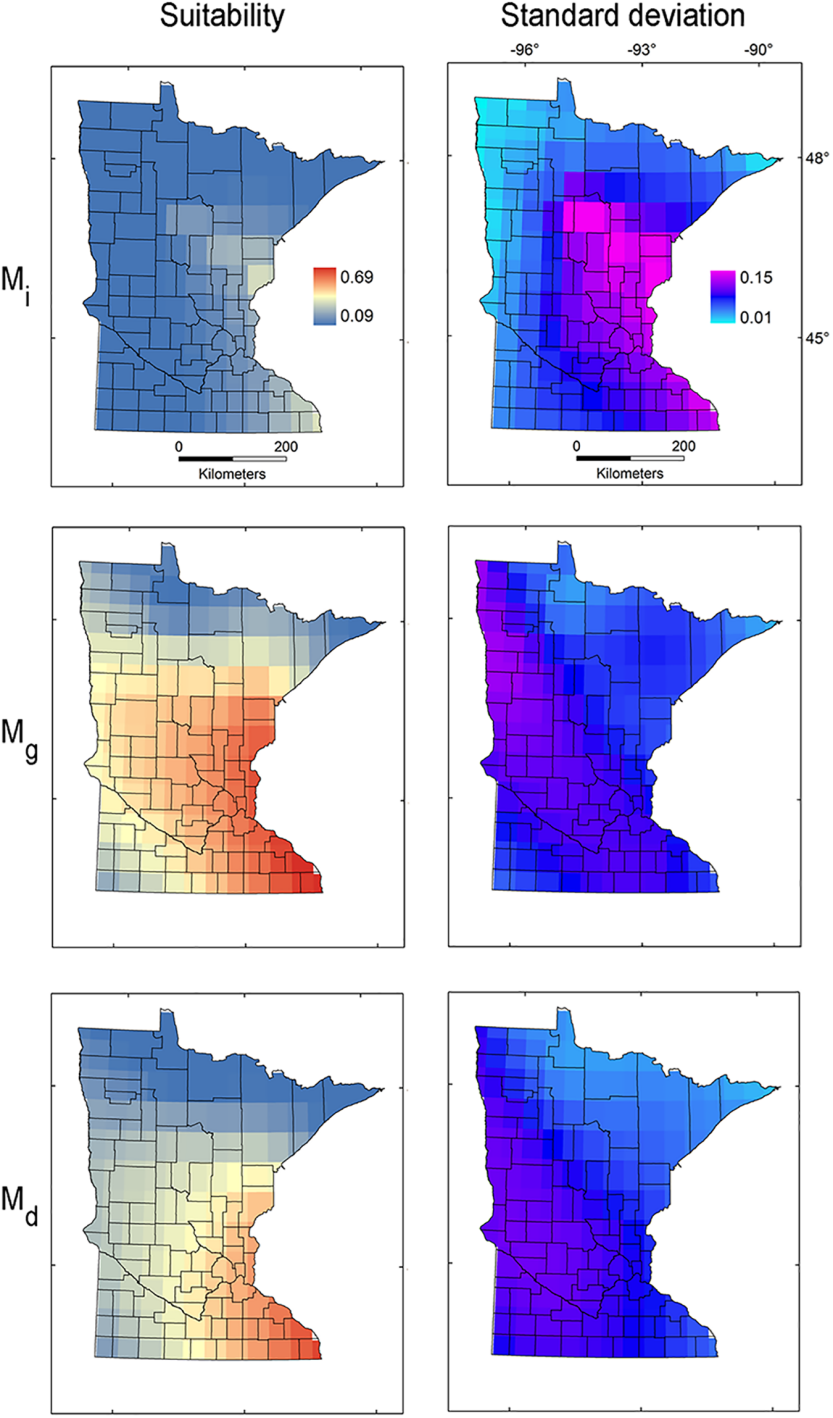
Ecological niche model transference to Minnesota under present-day climate. Ecological niche model predictions based on model calibration region in the invaded range with high dispersal (**M**_***i***_; top), entire species’ range with high dispersal (**M**_***g***_; mid), and entire species’ range with reduced dispersal (**M**_***d***_; bottom) projected to Minnesota to identify areas with high (red) or low (blue) environmental suitability (left) and high (pink) or low (light blue) model uncertainty (right).

Selected regularization coefficients differed by model calibration region: a regularization coefficient of 1.4 with LQHPT features provided the best fit (ΔAICc = 0) and good predictive performance (AUC_total_ = 0.98, AUC_mean_ = 0.96–0.97, OR_100%_ = 0.05–0.09, OR_90% =_ 0.14–0.16) for **M**_***i***_, 0.2 + LQ for **M**_***g***_ (ΔAICc = 0, AUC_total_ = 0.97, AUC_mean_ = 0.95–0.96, OR_100%_ = 0.01–0.04, OR_90%_ = 0.12–0.18), and 0.9 + LQ for **M**_***d***_ (ΔAICc = 0, AUC_total_ = 0.89, AUC_mean_ = 0.85–0.88, OR_100%_ = 0.07–0.19, OR_90%_ = 0.01–0.02; Table B in S1 File). Our evaluations revealed that 10,000 background points provided good model fit and performance for the three model calibration regions explored. Logistic suitability values of starry stonewort models based on **M**_***g***_ (mean = 0.40, sd = 0.13) vs. **M**_***i***_ (mean = 0.13, sd = 0.07) were significantly different (*t* = 1098, df = 544500, *p* < 0.001), with higher suitability predicted when **M**_***g***_ was considered (Fig 3). Logistic suitability values of starry stonewort models based on **M**_***d***_ (mean = 0.30, sd = 0.13) vs. **M**_***i***_, and vs. **M**_***g***_ were also significantly different, with **M**_***d***_ showing higher suitability than **M**_***i***_ (*t* = 717.16, df = 551600, *p* < 0.001) but less than **M**_***g***_ (*t* = 315.76, df = 732220, *p* < 0.001; Fig 3). Model uncertainty was higher in the model calibrated in **M**_***i***_ (**M**_***i***_ vs. **M**_***d***_: *t* = 20.10, df = 592650, *p* < 0.001; sd **M**_***i***_ vs. **M**_***g***_: *t* = 79.35, df = 536950, *p* < 0.001; Fig 3). In present-day models, we found potential areas for starry stonewort distribution in southeast and central Minnesota and also in the Minneapolis-St. Paul metro region. The portion of Minnesota where starry stonewort has been confirmed to date was predicted to have high suitability for the model calibrated based on **M**_***g***_ and **M**_***d***_ (Fig 3).

The **M**_***i***_ model based on the invasive population in the US predicted only a small area of moderate suitability in central and southeastern Minnesota (Fig 3), while the model based on the entire species’ range predicted a broad area of suitability across the state. Models from the global range **M**_***g***_ containing all the occurrences produced predictions with lower uncertainty. The **M**_***d***_ model calibrated based on the entire species range but with reduced dispersal potential predicted suitability resembling something between **M**_***i***_ and **M**_***g***_ (Fig 3). Prediction of starry stonewort suitability from **M**_***d***_ showed the highest uncertainty in western Minnesota.

**Fig 4:**
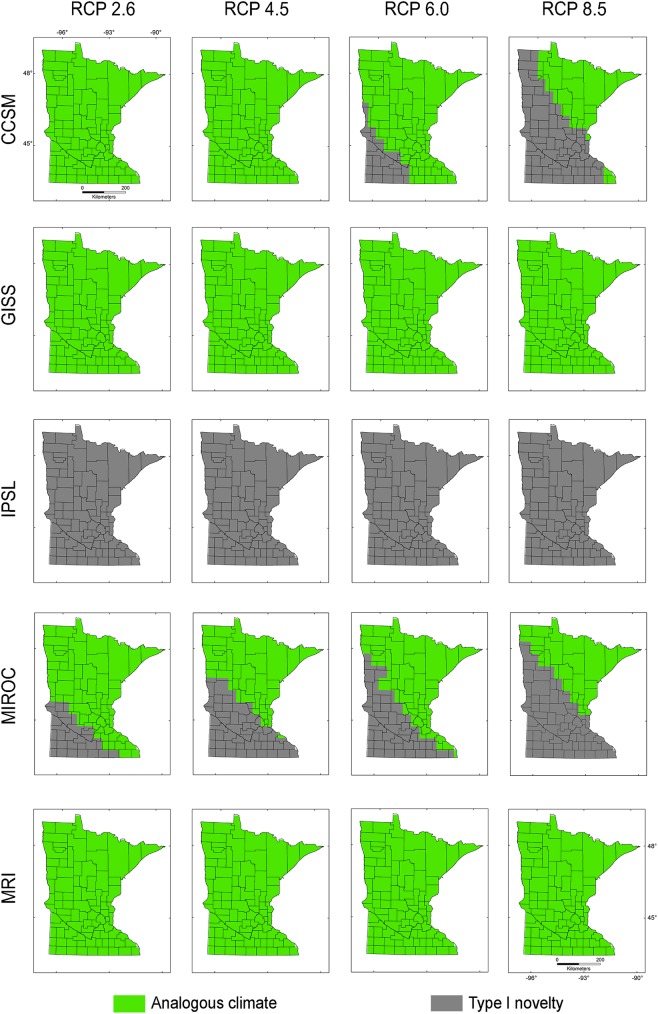
Environmental similarity comparison between the calibration M_*i*_ and the projection region of Minnesota. Exdet tool identified analogous climates between present-day climate in the calibration region from the invaded range and future climate scenarios in the projection region of Minnesota. Areas with analogous (green) and non-analogous environments in Minnesota (grey) were identified for five future climate models (i.e., CCSM, GISS, IPSL, MIROC, MRI) and four RCP scenarios of CO_2_ emissions (i.e., 2.6, 4.5, 6, and 8.5).

**Fig 5:**
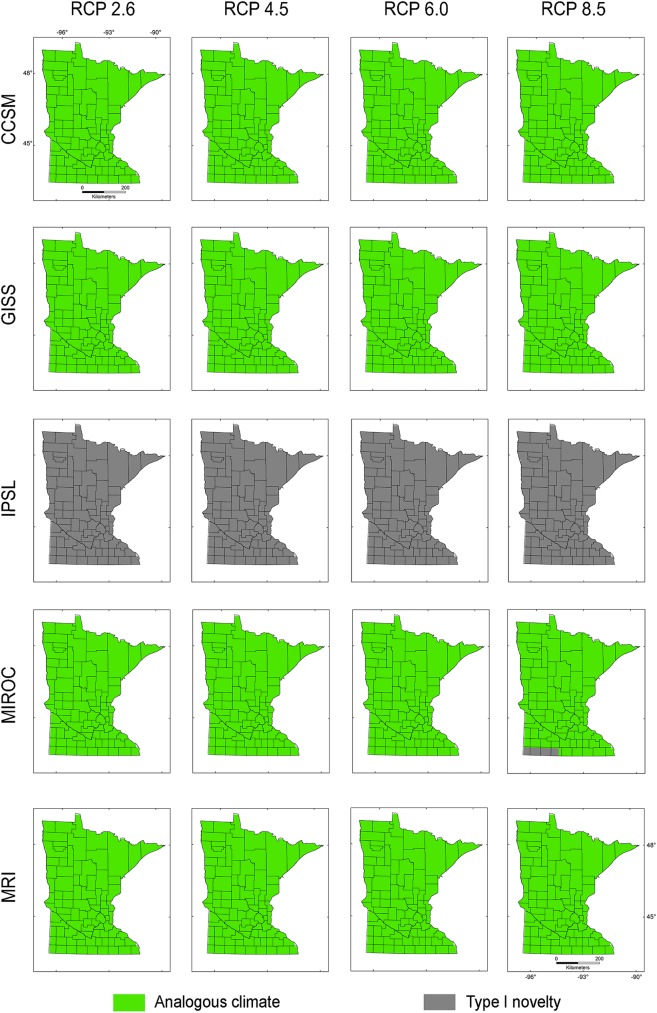
Environmental similarity comparison between the calibration M_*g*_ and the projection region of Minnesota. Legend as in Fig 3.

**Fig 6:**
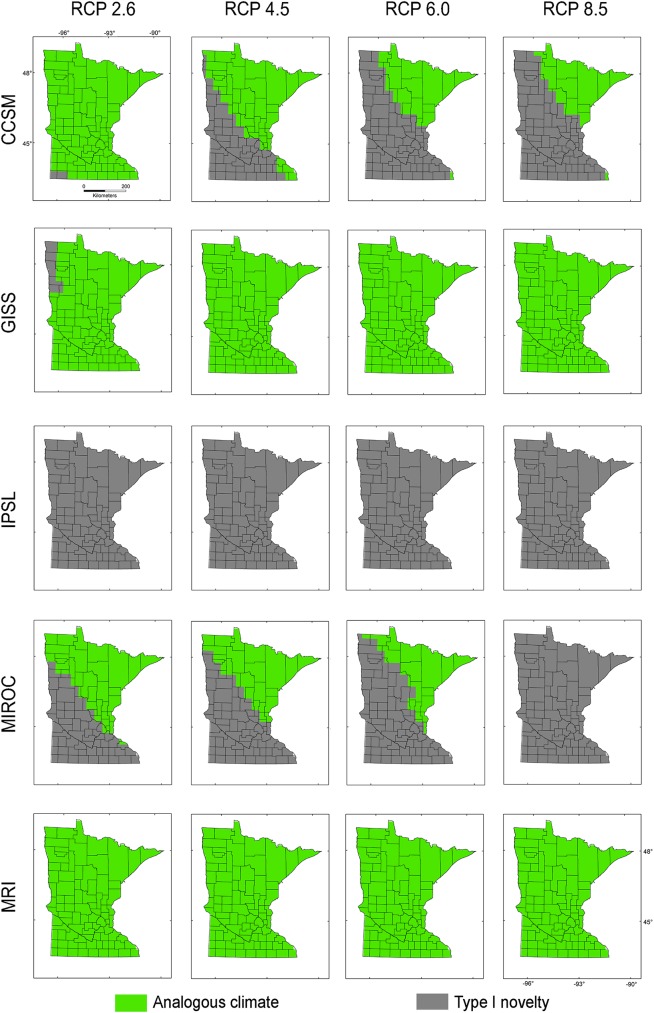
Environmental similarity comparison between the calibration M_*d*_ and the projection region of Minnesota. Legend as in Fig 3.

Present-day climate across **M**_***i***_, **M**_***g***_, and **M**_***d***_ showed non-analogous environments across Minnesota under all RCP scenarios of the IPSL climatic model (Figs 4–6). All MRI emission scenarios showed Minnesota having analogous climates. Other climate models and emission scenarios showed different non-analogous climate configuration according to the **M** scenarios employed (Figs 4–6). For example, **M**_***i***_ under present-day climatic conditions overlapped with future climate conditions for all RCP scenarios in climate models GISS and MRI, RCP 2.6 and 4.5 in CCSM, and maintained environmental similarity in the northeastern part of Minnesota in the MIROC model (Fig 4). This pattern was similar for **M**_***d***_ (Fig 6) despite the lack of analogous environments in MIROC RCP 8.5. Models calibrated based on **M**_***g***_ included analogous environments except in the case of all RCP scenarios in the IPSL model and MIROC RCP 8.5, which showed non-analogous environments in a small region in southwestern Minnesota (Fig 5). According to Exdet, non-analogous conditions for the IPSL model were driven mainly by differences in mean diurnal range, while novel climates in the MIROC RCP 2.6, 4.5, and 8.5 and CCSM RCP 6 and 8.5 were driven by extreme values of maximum temperature of the warmest month (Figs 4–6). Novel climates in MIROC RCP 6 model were explained by the maximum temperature of the warmest month and by the mean temperature of wettest quarter.

**Fig 7:**
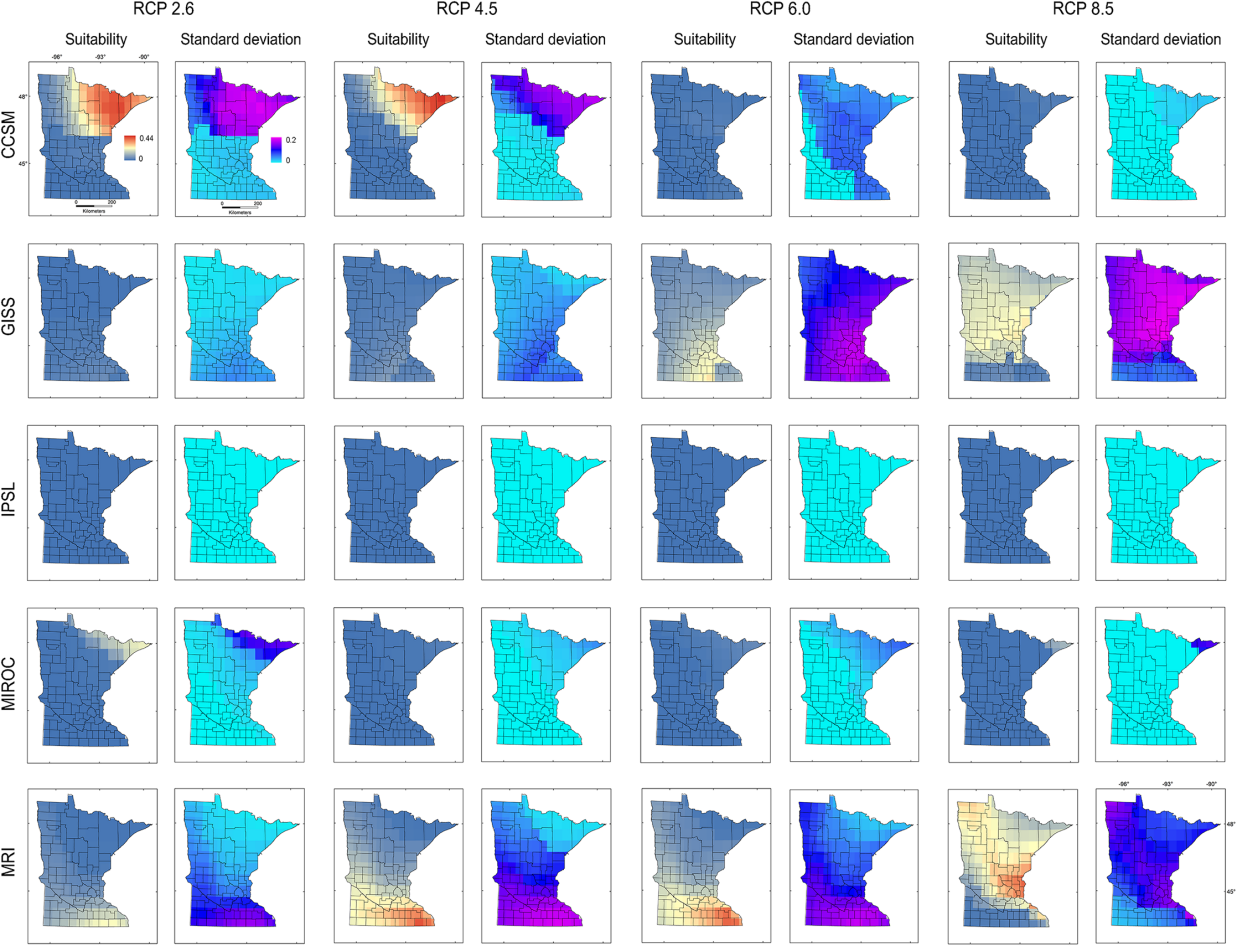
Ecological niche models of starry stonewort calibrated in M_*i*_ and projected to future climate scenarios in Minnesota. Ecological niche model predictions based on model calibration region **M**_***i***_ projected to Minnesota. Areas with high (red) or low (blue) environmental suitability (Suitability, left) and high (pink) or low (light blue) model uncertainty (Standard deviation, right) were identified for five future climate models (i.e., CCSM, GISS, IPSL, MIROC, MRI) and four RCP scenarios of CO_2_ emissions (i.e., 2.6, 4.5, 6, and 8.5).

**Fig 8:**
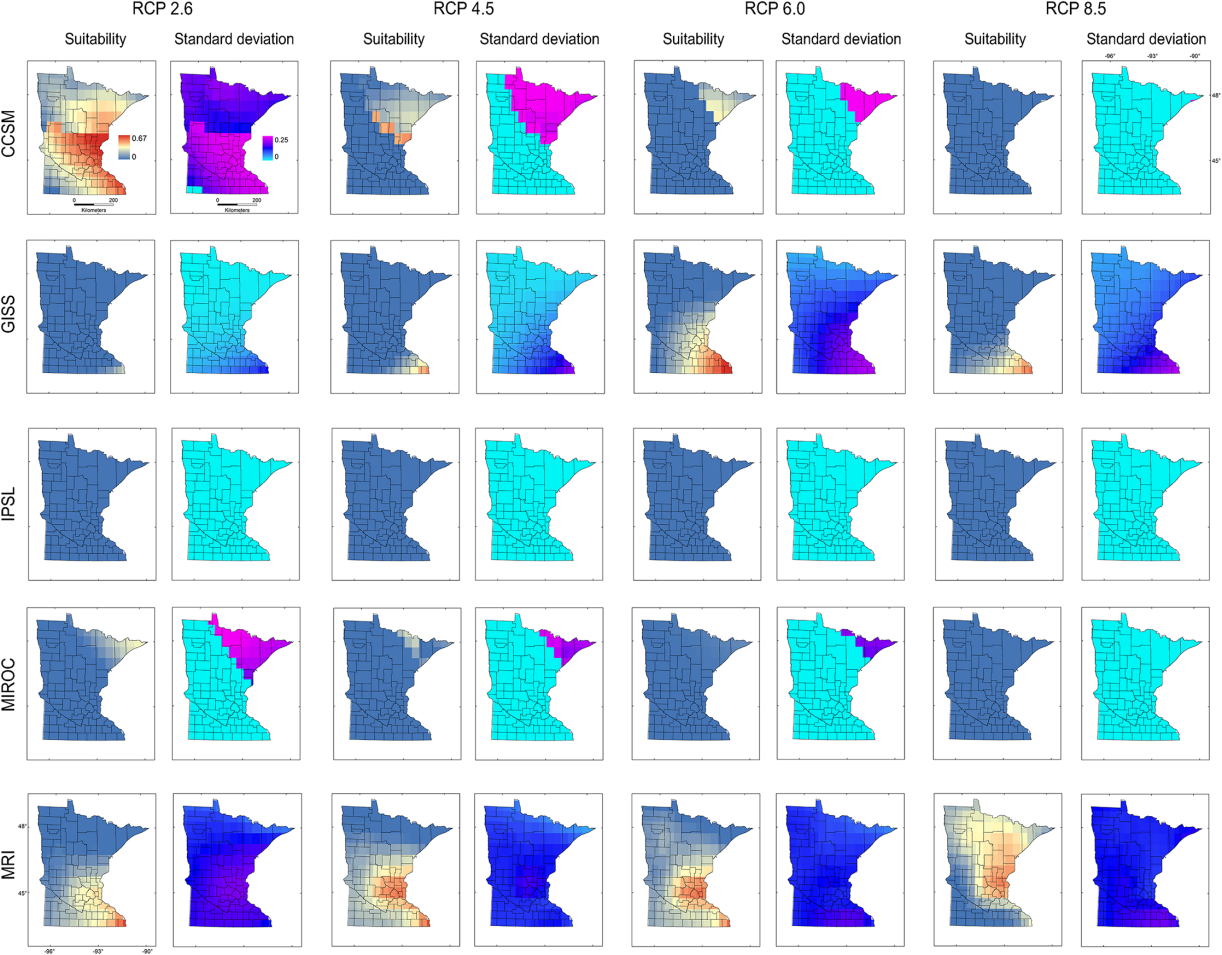
Ecological niche models of starry stonewort calibrated in M_*d*_ and projected to future climate scenarios in Minnesota. Ecological niche model predictions based on model calibration region **M**_***d***_ projected to Minnesota. Legend as in Fig 7.

Models calibrated based on **M**_***i***_ and **M**_***d***_ produced predictions with high uncertainties in Minnesota for all RCP scenarios (Figs 7 and 8). High suitability was predicted for **M**_***i***_ and **M**_***d***_ in scenarios CCSM RCP 2.6 and 4.5, MRI RCP 4.5, 6, and 8.5, and for **M**_***d***_ GISS RCP 6. Additionally, based on **M**_***i***_ and **M**_***d***_, models did not predict suitability under the IPSL climate model or predicted moderate suitability in small areas under the MIROC climate model (Figs 7 and 8), due to the absence of analogous environments (Figs 4 and 6).

**Fig 9:**
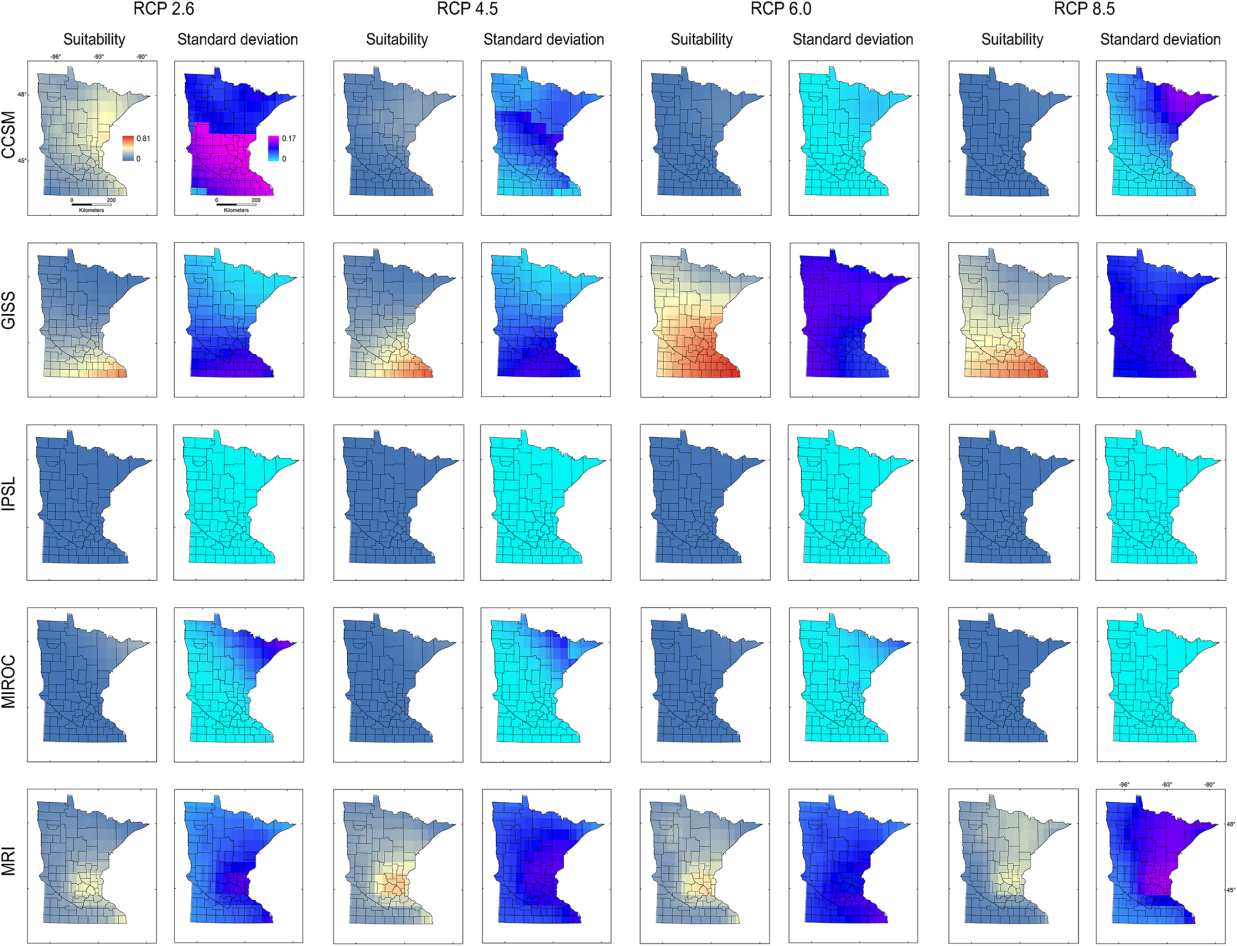
Ecological niche models of starry stonewort calibrated in M_*g*_ and projected to future climate scenarios in Minnesota. Ecological niche model predictions based on model calibration region **M**_***g***_ projected to Minnesota. Legend as in Fig 7.

**Fig 10:**
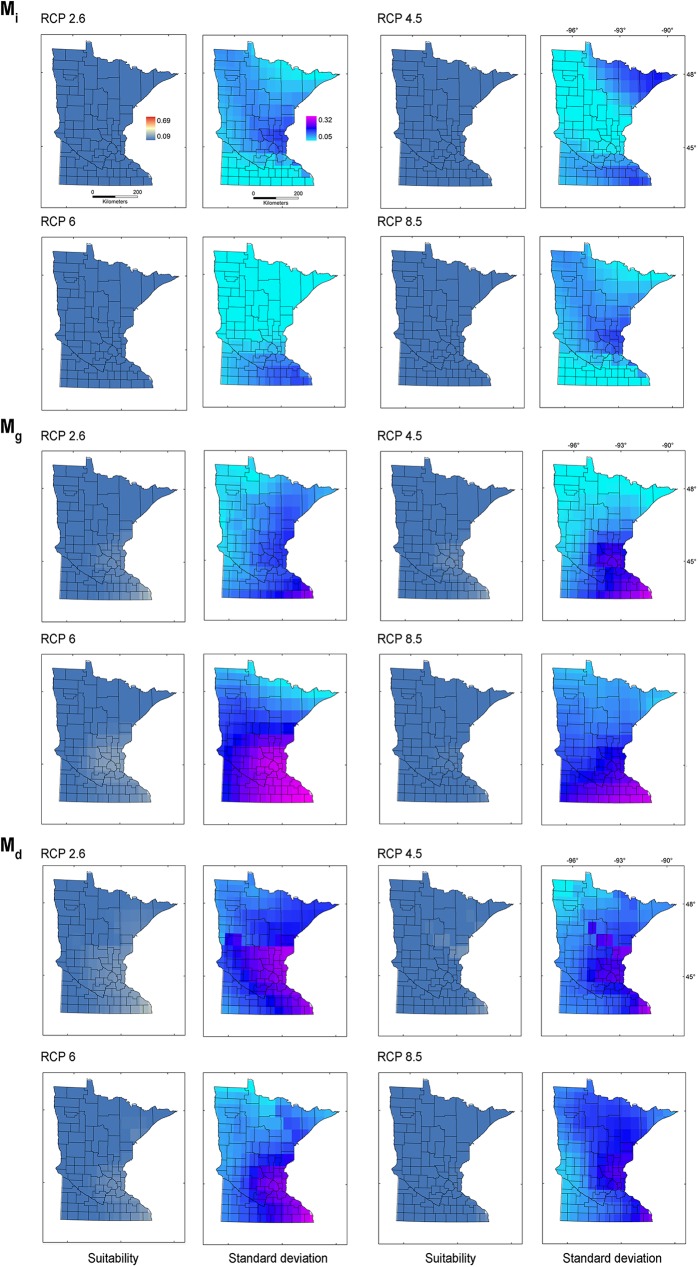
Starry stonewort future climate models ensemble. Model ensemble expressed as the average of continuous models in logistic format (left, ‘Suitability’), showing areas with high (red) or low (blue) suitability from all the RCP emission scenarios in comparison with the maximum range of suitability of climatic models projected to Minnesota in present environmental conditions (i.e., from the lowest [0.09] to the highest [0.69] suitability). Lack of agreement was estimated from the standard deviation of the final models (right, ‘Standard deviation’) and shows areas of high (pink) or low (light blue) disagreement among models. **Top**: Models calibrated in **M**_***i***_ and projected to future climate scenarios in Minnesota. **Mid**: Models calibrated in **M**_***g***_ and projected to future climate scenarios in Minnesota. **Bottom**: Models calibrated in **M**_***d***_ and projected to future climate scenarios in Minnesota.

The models from **M**_***g***_ transferred to future climate predicted an expansion of suitable areas under all GISS scenarios, with reduced suitability for future climate according to CCSM, IPSL, and MIROC (Fig 9). High variability was found for CCSM 2.6 and 8.5, GISS RCP 6, and all MRI scenarios. Some future climate scenarios indicated lack of suitability for starry stonewort throughout Minnesota (Fig 9). Suitability was not predicted for all IPSL scenarios due to non-analogous climates; while MIROC RCP 8.5 and CCSM RCP 8.5 showed unsuitability in analogous environmental conditions in all **M** scenarios. In general, climatic suitability is predicted to decrease under future climate conditions relative to present-day conditions (Fig 3 vs. Fig 10). The model ensemble showed a lack of agreement in predicted suitability among **M** calibration areas and RCP scenarios, with suitability values ranging from 0.01 to 0.12 for **M**_***i***_, 0.05 to 0.28 for **M**_***g***_, and from 0.06 to 0.30 for **M**_***d***_ (Fig 10). Areas with high values of suitability were also areas with high uncertainty in the model ensemble (Fig 10). In general, climatic suitability is predicted to decrease in the number of lakes of Minnesota under future climate conditions relative to present-day conditions except for the scenario RCP 2.6 from the climatic model CCSM and RCP 8.5 from MRI.

## Discussion

### Model predictions

We used a **BAM** ecological niche modeling framework to predict present-day and future climatic suitability throughout Minnesota for the aquatic invasive species starry stonewort. Under most future climate scenarios, the available range is predicted to shrink relative to present-day conditions. Based on the data available and the assumption of niche conservatism [49,50], all future climatic models under all RCP scenarios showed a decrease in suitable range relative to present-day conditions, with the exception of future climatic models: CCSM 2.6 and 4.5, and MRI RCP 4.5, 6, and 8.5 for **M**_***i*,**_ GISS RCP 6 for **M**_***g***_, and CCSM 2.6 and MRI 8.5 for **M**_***d***_, which showed increased areas of suitability with plausible range shifts. All these predictions, however, showed considerable uncertainty (Figs 7–9).

It is possible that our findings underestimate the potential invasiveness of starry stonewort by not capturing the full extent of its climatic tolerance [23]. Escobar et al. [8] recently described environmental tolerances of starry stonewort in its invaded and native ranges and found that invasion was associated with a shift in its realized niche, suggesting niche expansion, i.e., there were environmental conditions occupied by starry stonewort in the invaded range that lacked analogues in the native range [51]. This suggests that invasion potential may exceed what would be anticipated based on past performance alone, and starry stonewort may be able to expand into previously unoccupied environmental space [49,51]. Models could also be underestimating invasion due to overfitting from oversampled areas (i.e., sampling bias) and spatial autocorrelation in climatic variables; however, we minimized this risk by resampling occurrences to one per pixel and using coarse-resolution climatic variables, including data from remotely sensed imagery, to counter high spatial lag associated with data derived solely from climate stations [32,52,53].

The consensus areas of suitability across models (Fig 10) showed a pattern of reduced suitability across all **M** regions, suggesting a potential decline of the starry stonewort under warming climates in terms of the climates where the species is found to date. Model ensembles highlight areas of agreement across predictions, but their interpretation requires caution [17]. The lack of consensus of suitable areas for starry stonewort under future climate in Minnesota reflects the diversity of possible trajectories of future climate (Figs 7–9).

We note that our findings are based on estimated climatic tolerances and a proxy of establishment [23]. Numerous other factors, such as water chemistry, dispersal limitation, and agonistic interactions with resident biota, could limit starry stonewort expansion. However, a recent study of macrophyte communities in invaded lakes suggested plausible dominance of starry stonewort, with native species richness decreasing as starry stonewort increases in biomass [2]. These fine-scale, potentially complex and interacting factors cannot be accounted for in climate-based models, experiments would be needed to test the influence of these factors on starry stonewort population dynamics. Future research should assess how finer-scale abiotic variables (e.g., pH, conductivity, water clarity), biotic interactions, dispersal potential (via boater movement or natural water connectivity), and landscape factors (e.g., densities of roads and boat accesses) influence lake-level risk of starry stonewort invasion. Emergence of sexually reproductive populations could add new and longer-distance dispersal vectors due to small oospores that could potentially be spread by waterbirds or survive overland transport longer than bulbils [21].

### Environmental variables

The environmental variables derived from the Ecoclimate repository are a promising alternative for modeling species distributed across inland and coastal/marine ecosystems [32], providing robust data on climatic variability needed for ecological niche models [54]. The 50-km spatial resolution of Ecoclimate variables mitigate the high spatial lag of finer-resolution climatic layers [52,53], which can produce flawed estimates due to high spatial autocorrelation from statistical downscaling [32,53]. We argue that during exploratory analyses, coarse-scale variables are useful for identifying plausible constraints for species establishment. Subsequent work can then incorporate finer-scale environmental variables (derived from remote sensing or habitat data) to complement climate-based models. Additionally, we developed analyses incorporating five future climate models: CCSM, GISS, IPSL, MIROC, and MRI, and four RCP emission scenarios: 2.6, 4.5, 6, 8.5. This allowed us to investigate a broader range of plausible climate scenarios. Ecological niche modeling of species invasions under future climates should incorporate alternative climate models and emission scenarios to reflect the uncertainty in future conditions.

### The calibration region M

In agreement with previous studies using virtual species [29], our models based on empirical data suggest that a careless definition of the calibration region, **M**, may produce flawed results [23]. Restricting the model calibration region only to the invaded region, **M**_***i***_, in present-day climate (Fig 2), narrowed geographic predictions to southeastern Minnesota—all actual occurrences to date are outside of this region—as a result of the incomplete information provided to the algorithm (Fig 3). In contrast, considering the entire species’ range for the two calibration regions **M**_***g***_ and **M**_***d***_ (Fig 2) included portions of central and central-north Minnesota where starry stonewort has known occurrences (Fig 3). We found that increasing the model calibration area generated an increase in AUC values, but from a practical perspective, accounting for environmental conditions available in the entire range produced forecasts that were more reliable and more precautionary [30]; this suggests that AUC may not accurately reflect model performance due to high sensitivity of this metric to the extent of the model calibration region [29].

**Fig 11:**
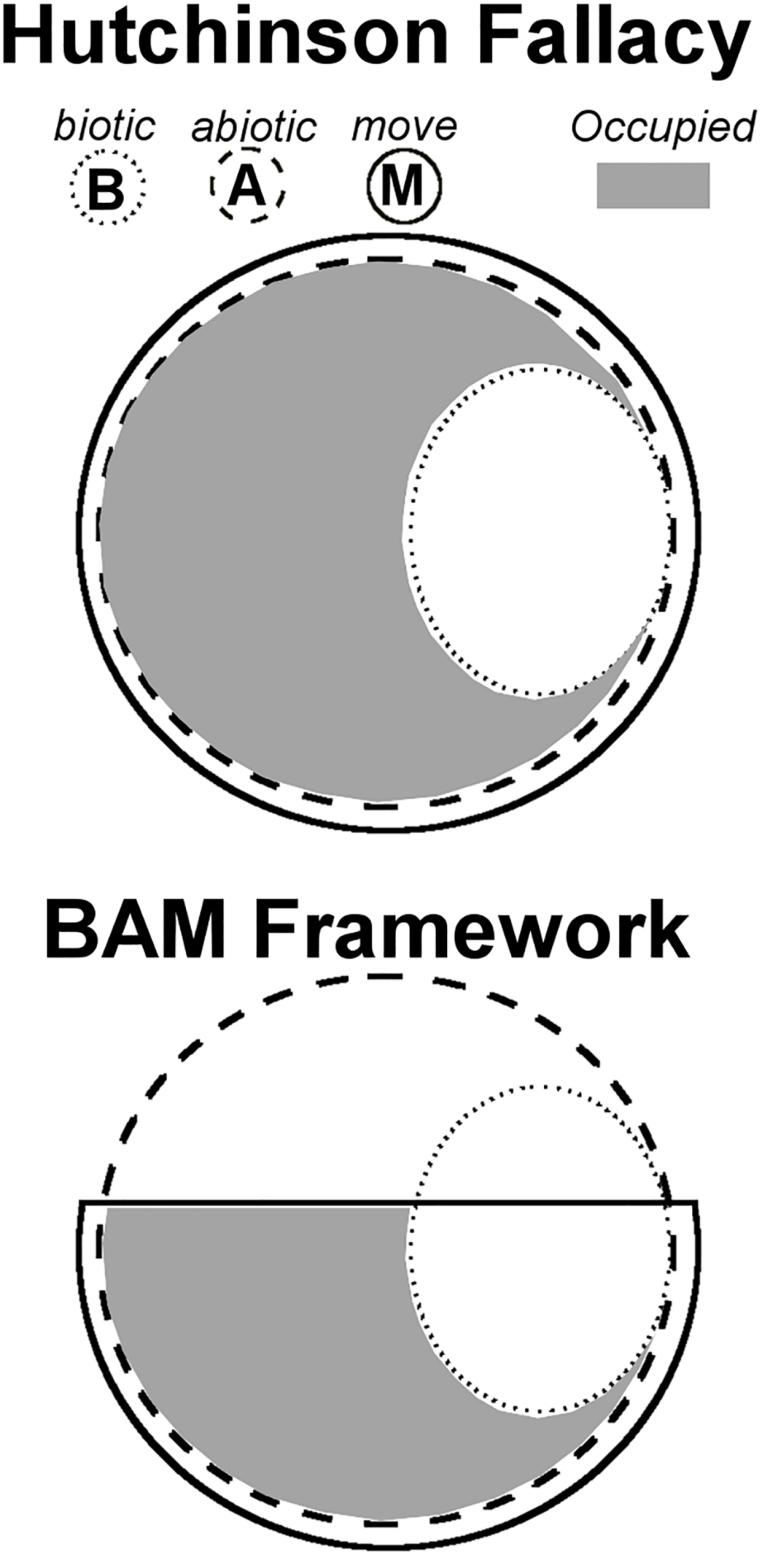
Conceptual framework used for interpretation of predictions. **Top:** The “Hutchinson Fallacy” expressed as the intersect of abiotic (**A**; dashed line) and biotic factors (**B**; dotted line) showing the environments that a species can occupy (gray) or not (white area inside the dotted circle), based on biotic interactions solely (e.g., competitors). Note that under the Hutchinson’s proposal, all the areas environmentally suitable can be reached by the species (i.e., entire circle), suggesting that the movement and dispersal potential of the species (**M**; solid line) is effective to occupy all the suitable conditions (i.e., **A** is contained in **M**). **Bottom**: The “BAM Framework” proposed by Soberón and Peterson [23] to explain that dispersal limitations (**M**) can also restrict the species to occupy (gray) only a portion of all the suitable environments (**A**). Note that in this example, the species can occupy a portion of the environmental conditions suitable due to the limited dispersal potential (i.e., half circle). **A** (abiotic) = environmental conditions suitable for the species; **B** (biotic) = interaction with other species; **M** (move) = movement or dispersal potential of the species.

From a theoretical perspective, niche estimations should be guided by modern ecological niche theory [23]. According to Hutchinson [13,55], ecological niches occur in multidimensional environmental space, and species may not occupy all suitable abiotic environments (**A**) due solely to limiting biotic interactions (**B**; e.g., competition) (Fig 11 top). However, Soberón and Peterson [23] propose that Hutchinson’s ideas were incomplete and that, in addition to **B**, a species can also be limited by its dispersal potential (**M**) (Fig 11 bottom). They propose that species rarely occupy their entire environmental potential and that the Hutchinsonian framework needs to be expanded. The **BAM** framework proposes that for a realistic **A** estimation for an invasive species, studies should include delimitations of **M** allowing a representative characterization of the dispersal potential of the species [23]. In other words, models aiming to estimate a good proxy of **A** should include all the areas where the species occurs, including the full native and invaded ranges. Thus, we stress that ecological niche modeling to forecast current and future biological invasions are dependent upon **M** (Fig 10 bottom). Ecological niche models calibrated in only a portion of the species’ range or under a single **M** scenario may underestimate invasive potential (Fig 3). In this vein, our estimation of dispersal potential based on distance between populations in the invaded range may be confounded by search effort and may not reflect the actual directionality of spread. Genetic/genomic analyses could be used to reconstruct dispersal potential, invasion pathways, and directionality.

The extent of the calibration region was also crucial to establish the presence or absence of novel environments between calibration and projection regions, and between present-day and future climates [34,46]. Models **M**_***i***_ calibrated from the invaded range only, and models **M**_***d***_ calibrated based on a small dispersal potential (Fig 2), showed high levels of truncation of prediction in non-analogous novel climatic conditions across Minnesota, limiting our ability to project models to future scenarios (Figs 4 and 6). Conversely, **M**_***g***_ models from the entire species range with a hypothetical high dispersal identified suitable areas for starry stonewort in Minnesota under present-day and most future climate scenarios (Figs 5 and 9). This provides additional evidence that the calibration region extent plays a key role in ecological niche model projections for species invasions. Thus, model calibration regions should include the full distribution of the studied species under different **M** scenarios to capture the fullest possible set of environmental determinants of physiological tolerance of the organism, providing a firmer biological foundation for calibration region selection [13,31]. We urge researchers and reviewers to put special attention to the justification and biological support of the **M** area selected for model calibration in past and future ecological niche modeling studies.

### Maxent and model evaluation

Current literature advocates Maxent for niche modeling due to its accessibility, user-friendly interface, and supporting literature [39]. However, the potential of Maxent to overestimate or overfit predictions to the data available must be considered [18,27,38,39,41]. Maxent must be fitted for each study case considering the natural history of the species, the data available, and the assumptions involved. The results from our approach to control the extent of the calibration region, which included use of regularization coefficients, information-theory model selection, strict model evaluation, and strict model transference, support the contention that using the default parameterizations of Maxent, while convenient, is an inappropriate approach that can lead to inaccurate conclusions [29,41,46]. Thus, each modeling effort should include detailed individualized parameter selection, and model results should be critically assessed to determine if they are biologically sound, avoiding reliance on single model estimates [37].

Although predicted suitability from our present-day models ranged from minimal to broad across Minnesota (Fig 3), models with the two different calibration regions performed well in terms of omission rates and AUC values [40]. The heterogeneous suitability predicted under the two configurations reflects the sensitivity of ecological niche models to experimental design decisions (Fig 2) [13]; therefore, we propose that uncertainty estimation must be included as an essential component of ecological niche model estimations.

## Conclusions

Starry stonewort is predicted to expand its current geographic range into novel areas across Minnesota under present-day climate conditions. Under future climate conditions, we estimate a reduction in suitability for the species. Our models are a step toward the development of management strategies to prevent and mitigate the spread of this species on the leading edge of its invasion. It is crucial to develop strategic interventions that target the role of human activities in starry stonewort spread. Further, our results suggest that sound forecasts require rigorous model design and evaluations to improve their reliability.

## Supporting information

S1 File **Table A.** Correlation matrix of environmental variables. **Table B.** Summary of model evaluations.
(DOCX)

## References

[1] Sleith RS, Havens AJ, Stewart RA, Karol KG. Distribution of *Nitellopsis obtusa* (Characeae) in New York, U.S.A. Brittonia. 2015;67: 166–172. doi: 10.1007/s12228-015-9372-6

[2] Brainard AS, Schulz KL. Impacts of the cryptic macroalgal invader, *Nitellopsis obtusa*, on macrophyte communities. Freshw Sci. 2016;36: in press. doi: 10.1086/689676

[3] Midwood JDD, Darwin A, Ho ZZ-Y, Rokitnicki-Wojcik D, Grabas G. Environmental factors associated with the distribution of non-native starry stonewort (*Nitellopsis obtusa*) in a Lake Ontario coastal wetland. J Great Lakes Res. 2016;42: 348–355. doi: 10.1016/j.jglr.2016.01.005

[4] Pullman GDD, Crawford G. A decade of starry stonewort in Michigan. LakeLine. 2010;Summer: 36–42.

[5] Joint Nature Conservation Committee. UK priority species pages–Version 2 [Internet]. Peterborough; 2010 [cited 8 Jan 2016]. Available: http://jncc.defra.gov.uk/_speciespages/474.pdf

[6] HELCOM. Baltic Marine Environment Protection Comission—Helsinki Comission. Red list Nitellopsis obtusa [Internet]. 2013 pp. 2012–2014. Available: http://www.helcom.fi/RedListSpeciesInformationSheet/HELCOMRedListNitellopsisobtusa.pdf#search=NitellopsisObtusa

[7] Kato S, Kawai H, Takimoto M, Suga H, Yohda K, Horiya K, et al Occurrence of the endangered species *Nitellopsis obtusa* (Charales, Charophyceae) in western Japan and the genetic differences within and among Japanese populations. Phycol Res. 2014;62: 222–227. doi: 10.1111/pre.12057

[8] Escobar LE, Qiao H, Phelps NBD, Wagner CK, Larkin DJ. Realized niche shift associated with the Eurasian charophyte *Nitellopsis obtusa* becoming invasive in North America. Sci Rep. 2016;6: 29037 doi: 10.1038/srep29037 27363541 10.1038/srep29037 PMC4929560

[9] MISIN. Midwest Invasive Species Information Network. In: Michigan State University [Internet]. 2015 [cited 9 Jan 2016]. Available: http://www.misin.msu.edu/

[10] Geis JW, Schumacher GJ, Raynal DJ, Hyduke NP. Distribution of Nitellopsis obtusa (Charophyceae, Characeae) in the St Lawrence River: A new record for North America. Phycologia. 1981;20: 211–214. doi: 10.2216/i0031-8884-20-2-211.1

[11] Kipp RM, McCarthy M, Fusaro A, Pfingsten IA. Nitellopsis obtusa Nonindigenous Aquatic Species Database, Gainesville, FL, and NOAA Great Lakes Aquatic Nonindigenous Species Information System, Ann Arbor, MI. [Internet]. Available: https://nas.er.usgs.gov/queries/GreatLakes/FactSheet.aspx?NoCache=10/12/2010+4:29:34+AM&SpeciesID=1688&State=&HUCNumb

[12] DNR M. DNR taking further steps to reduce risk of starry stonewort spread [Internet]. St. Paul: Minnesota Department of Natural Resources; 2015 [cited 11 Jan 2016]. Available: http://news.dnr.state.mn.us/2015/10/02/dnr-taking-further-steps-to-reduce-risk-of-starry-stonewort-spread/

[13] Peterson AT, Soberón J, Pearson RG, Anderson RP, Martínez-Meyer E, Nakamura M, et al Ecological Niches and Geographic Distributions. New Jersey: Princeton University Press; 2011.

[14] Peterson AT, Papeş M, Kluza DA. Predicting the potential invasive distributions of four alien plant species in North America. Weed Sci. 2003;51: 863–868. doi: 10.1614/P2002-081

[15] Papeş M, Havel JEE, Vander Zanden MJJ. Using maximum entropy to predict the potential distribution of an invasive freshwater snail. Freshw Biol. 2016;61: 457–471. doi: 10.1111/fwb.12719

[16] Escobar LE, Ryan SJ, Stewart-Ibarra AM, Finkelstein JL, King CA, Qiao H, et al A global map of suitability for coastal Vibrio cholerae under current and future climate conditions. Acta Trop. 2015;149: 202–211. doi: 10.1016/j.actatropica.2015.05.028 26048558 10.1016/j.actatropica.2015.05.028

[17] Wiens JA, Stralberg D, Jongsomjit D, Howell CA, Snyder MA. Niches, models, and climate change: Assessing the assumptions and uncertainties. Proc Natl Acad Sci USA. 2009;106: 19729–19736. doi: 10.1073/pnas.0901639106 19822750 10.1073/pnas.0901639106 PMC2780938

[18] Anderson RP. A framework for using niche models to estimate impacts of climate change on species distributions. Ann N Y Acad Sci. 2013;1297: 8–28. doi: 10.1111/nyas.12264 25098379 10.1111/nyas.12264

[19] Gelviz-Gelvez SM, Pavón NP, Illoldi-Rangel P, Ballesteros-Barrera C. Ecological niche modeling under climate change to select shrubs for ecological restoration in Central Mexico. Ecol Eng. 2015;74: 302–309. doi: 10.1016/j.ecoleng.2014.09.082

[20] Warren DL, Wright AN, Seifert SN, Shaffer HB. Incorporating model complexity and spatial sampling bias into ecological niche models of climate change risks faced by 90 California vertebrate species of concern. Divers Distrib. 2014;20: 334–343. doi: 10.1111/ddi.12160

[21] Lockwood JL, Hoopes MF, Marchetti MP. Invasion Ecology. Malden: Wiley-Blackwell; 2006.

[22] Theoharides KA, Dukes JS. Plant invasion across space and time: Factors affecting nonindigenous species success during four stages of invasion. New Phytol. 2007;176: 256–273. doi: 10.1111/j.1469-8137.2007.02207.x 17822399 10.1111/j.1469-8137.2007.02207.x

[23] Soberón J, Peterson AT. Interpretation of models of fundamental ecological niches and species’ distributional areas. Biodivers Informatics. 2005;2: 1–10.

[24] GBIF. Global Biodiversity Information Faclity [Internet]. 2015 [cited 5 May 2015]. Available: http://www.gbif.org/

[25] GISIN. Global Invasive Species Information Network, Providing Free and Open Access to Invasive Species Data [Internet]. 2015 [cited 25 Oct 2015]. Available: http://www.gisin.org

[26] Mills EL, Leach JH, Carlton JT, Secor CL. Exotic species in the Great Lakes: A history of biotic crises and anthropogenic introductions. J Great Lakes Res. 1993;19: 1–54. doi: 10.1016/S0380-1330(93)71197-1

[27] Radosavljevic A, Anderson RP. Making better Maxent models of species distributions: Complexity, overfitting and evaluation. J Biogeogr. 2014;41: 629–643. doi: 10.1111/jbi.12227

[28] Escobar LE, Lira-Noriega A, Medina-Vogel G, Peterson AT. Potential for spread of White-nose fungus (*Pseudogymnoascus destructans*) in the Americas: Using Maxent and NicheA to assure strict model transference. Geospat Health. 2014;11: 221–229. doi: 10.4081/gh.2014.19 10.4081/gh.2014.19 25545939

[29] Barve N, Barve V, Jiménez-Valverde A, Lira-Noriega A, Maher SP, Peterson AT, et al The crucial role of the accessible area in ecological niche modeling and species distribution modeling. Ecol Modell. 2011;222: 1810–1819. doi: 10.1016/j.ecolmodel.2011.02.011

[30] Broennimann O, Guisan A. Predicting current and future biological invasions: Both native and invaded ranges matter. Biol Lett. 2008;4: 585–589. doi: 10.1098/rsbl.2008.0254 18664415 10.1098/rsbl.2008.0254 PMC2610080

[31] Jiménez-Valverde A, Peterson AT, Soberón J, Overton JM, Aragón P, Lobo JM. Use of niche models in invasive species risk assessments. Biol Invasions. 2011;13: 2785–2797. doi: 10.1007/s10530-011-9963-4

[32] Lima-Ribeiro MS, Varela S, Gonzales-Hernandez J, de Oliveira G, Diniz-Filho JAF, Terrible LC. ecoClimate: A database of climate data from multiple models for past, present, and future for macroecologists and biogeographers. Biodivers Informatics. 2015;10: 1–21.

[33] Harris RMB, Grose MR, Lee G, Bindoff NL, Porfirio LL, Fox-Hughes P. Climate projections for ecologists. Wiley Interdiscip Rev Clim Chang. 2014;5: 621–637. doi: 10.1002/wcc.291

[34] Mesgaran MB, Cousens RD, Webber BL. Here be dragons: A tool for quantifying novelty due to covariate range and correlation change when projecting species distribution models. Divers Distrib. 2014;20: 1147–1159. doi: 10.1111/ddi.12209

[35] Elith J, Kearney M, Phillips SJ. The art of modelling range-shifting species. Methods Ecol Evol. 2010;1: 330–342. doi: 10.1111/j.2041-210X.2010.00036.x

[36] Anderson RP. El modelado de nichos y distribuciones: No es simplemente “clic, clic, clic.” I Simposio de Biogeografía: Actualidad y Retos. Puebla: XII Congreso Nacional de Mastozoología; 2014. pp. 11–27.

[37] Qiao H, Soberón J, Peterson AT. No silver bullets in correlative ecological niche modelling: Insights from testing among many potential algorithms for niche estimation. Methods Ecol Evol. 2015;6: 1126–1136. doi: 10.1111/2041-210X.12397

[38] Phillips SJ, Anderson RP, Schapire RE. Maximum entropy modeling of species geographic distributions. Ecol Modell. 2006;190: 231–259. doi: 10.1016/j.ecolmodel.2005.03.026

[39] Merow C, Smith MJ, Silander JA. A practical guide to MaxEnt for modeling species’ distributions: What it does, and why inputs and settings matter. Ecography. 2013;36: 1058–1069. doi: 10.1111/j.1600-0587.2013.07872.x

[40] Muscarella R, Galante PJ, Soley-Guardia M, Boria RA, Kass JM, Uriarte M, et al ENMeval: An R package for conducting spatially independent evaluations and estimating optimal model complexity for Maxent ecological niche models. Methods Ecol Evol. 2014;5: 1198–1205. doi: 10.1111/2041-210X.12261

[41] Warren DL, Seifert SN. Ecological niche modeling in Maxent: The importance of model complexity and the performance of model selection criteria. Ecol Appl. 2011;21: 335–342. doi: 10.1890/10-1171.1 21563566 10.1890/10-1171.1

[42] Burnham KP, Anderson DR, Huyvaert KP. AIC model selection and multimodel inference in behavioral ecology: Some background, observations, and comparisons. Behav Ecol Sociobiol. 2011;65: 23–35. doi: 10.1007/s00265-010-1029-6

[43] Golicher D, Ford A, Cayuela L, Newton A. Pseudo-absences, pseudo-models and pseudo-niches: Pitfalls of model selection based on the area under the curve. Int J Geogr Inf Sci. 2012;8816: 1–15. doi: 10.1080/13658816.2012.719626

[44] Lobo JM, Jiménez-Valverde A, Real R. AUC: A misleading measure of the performance of predictive distribution models. Glob Ecol Biogeogr. 2007;17: 145–151. doi: 10.1111/j.1466-8238.2007.00358.x

[45] Peterson ATT, Papes M, Soberón J, Papeş M, Soberón J. Rethinking receiver operating characteristic analysis applications in ecological niche modeling. Ecol Modell. 2008;213: 63–72. doi: 10.1016/j.ecolmodel.2007.11.008

[46] Owens HL, Campbell LP, Dornak LL, Saupe EE, Barve N, Soberón J, et al Constraints on interpretation of ecological niche models by limited environmental ranges on calibration areas. Ecol Modell. 2013;263: 10–18. doi: 10.1016/j.ecolmodel.2013.04.011

[47] Elith J, Phillips SJ, Hastie T, Dudík M, Chee YE, Yates CJ. A statistical explanation of Maxent for ecologists. Divers Distrib. 2011;17: 43–57. doi: 10.1111/j.1472-4642.2010.00725.x

[48] U.S. Fish & Wildlife Service. National Wetlands Inventory [Internet]. Falls Church: National Wetlands Inventory; 2015 [cited 15 Feb 2016]. Available: http://www.fws.gov/wetlands/data/State-Downloads.html

[49] Petitpierre B, Kueffer C, Broennimann O, Randin C, Daehler C, Guisan A. Climatic niche shifts are rare among terrestrial plant invaders. Science. 2012;335: 1344–1348. doi: 10.1126/science.1215933 22422981 10.1126/science.1215933

[50] Pearman PB, Guisan A, Broennimann O, Randin CF. Niche dynamics in space and time. Trends Ecol Evol. 2008;23: 149–158. doi: 10.1016/j.tree.2007.11.005 18289716 10.1016/j.tree.2007.11.005

[51] Guisan A, Petitpierre B, Broennimann O, Daehler C, Kueffer C. Unifying niche shift studies: Insights from biological invasions. Trends Ecol Evol. 2014;29: 260–269. doi: 10.1016/j.tree.2014.02.009 24656621 10.1016/j.tree.2014.02.009

[52] Peterson AT. Mapping Disease Transmission Risk: Enriching Models Using Biology and Ecology. Baltimore: Johns Hopkins University Press; 2014.

[53] Escobar LE, Peterson AT. Spatial epidemiology of bat-borne rabies in Colombia. Pan Am J Public Heal. 2013;34: 135–136. 24096979

[54] Waltari E, Schroeder R, McDonald K, Anderson RP, Carnaval A. Bioclimatic variables derived from remote sensing: Assessment and application for species distribution modelling. Methods in Ecology and Evolution. 2014 pp. 1033–1042. doi: 10.1111/2041-210X.12264

[55] Hutchinson GE. Concluding remarks. Cold Spring Harb Symp Quant Biol. 1957;22: 415–427.

