# Supplementary material for: Forecasting distributions of an aquatic invasive species (Nitellopsis obtusa) under future climate scenarios
Source: PLoS One. 2017 Jul 13;12(7):e0180930. doi: 10.1371/journal.pone.0180930 (PMC5509285; doi:10.1371/journal.pone.0180930)
Supplement: S1 File — Table A. Correlation matrix of environmental variables. Table B. Summary of model evaluations. (DOCX) [file pone.0180930.s001.docx]

**Forecasting distributions of an aquatic invasive species (*Nitellopsis obtusa*) under future climate scenarios**

Daniel Romero-Alvarez, Luis E. Escobar^*^, Sara Varela, Daniel J. Larkin, Nicholas B. D. Phelps

*To whom correspondence should be addressed: E-mail: lescobar@umn.edu

**Supporting information S1**

- **S1 File Table A.** Correlation matrix of environmental variables.
- **S1 File Table B.** Summary of model evaluations.

| **Variables** | **Bio1** | **Bio2** | **Bio3** | **Bio4** | **Bio5** | **Bio6** | **Bio7** | **Bio8** | **Bio9** | **Bio10** | **Bio11** | **Bio12** | **Bio13** | **Bio14** | **Bio15** | **Bio16** | **Bio17** | **Bio18** | **Bio19** |
| --- | --- | --- | --- | --- | --- | --- | --- | --- | --- | --- | --- | --- | --- | --- | --- | --- | --- | --- | --- |
| **Bio1** | 1.00 | -0.48 | 0.05 | -0.88 | 0.66 | 0.97 | -0.81 | 0.68 | 0.93 | 0.91 | 0.98 | 0.66 | 0.76 | 0.59 | -0.23 | 0.71 | 0.60 | 0.43 | 0.65 |
| **Bio2** | 1.00 | 1.00 | 0.75 | 0.77 | 0.27 | -0.65 | 0.87 | -0.01 | -0.55 | -0.14 | -0.61 | -0.48 | -0.51 | -0.42 | 0.09 | -0.52 | -0.44 | -0.09 | -0.57 |
| **Bio3** |  |  | 1.00 | 0.23 | 0.55 | -0.11 | 0.37 | 0.27 | 0.00 | 0.29 | -0.05 | -0.05 | -0.04 | -0.01 | -0.13 | -0.06 | -0.03 | 0.27 | -0.16 |
| **Bio4** |  |  |  | 1.00 | -0.24 | -0.96 | 0.98 | -0.39 | -0.90 | -0.60 | -0.95 | -0.73 | -0.80 | -0.65 | 0.28 | -0.78 | -0.66 | -0.40 | -0.75 |
| **Bio5** |  |  |  |  | 1.00 | 0.50 | -0.11 | 0.75 | 0.51 | 0.91 | 0.53 | 0.24 | 0.32 | 0.20 | -0.07 | 0.25 | 0.20 | 0.27 | 0.18 |
| **Bio6** |  |  |  |  |  | 1.00 | -0.92 | 0.57 | 0.95 | 0.80 | 1.00 | 0.69 | 0.78 | 0.62 | -0.23 | 0.75 | 0.63 | 0.39 | 0.71 |
| **Bio7** |  |  |  |  |  |  | 1.00 | -0.31 | -0.85 | -0.50 | -0.90 | -0.69 | -0.75 | -0.61 | 0.23 | -0.74 | -0.63 | -0.33 | -0.73 |
| **Bio8** |  |  |  |  |  |  |  | 1.00 | 0.44 | 0.79 | 0.59 | 0.27 | 0.40 | 0.22 | 0.02 | 0.34 | 0.19 | 0.33 | 0.16 |
| **Bio9** |  |  |  |  |  |  |  |  | 1.00 | 0.78 | 0.95 | 0.68 | 0.74 | 0.61 | -0.30 | 0.71 | 0.63 | 0.36 | 0.72 |
| **Bio10** |  |  |  |  |  |  |  |  |  | 1.00 | 0.82 | 0.49 | 0.59 | 0.43 | -0.15 | 0.52 | 0.43 | 0.37 | 0.46 |
| **Bio11** |  |  |  |  |  |  |  |  |  |  | 1.00 | 0.70 | 0.79 | 0.63 | -0.25 | 0.76 | 0.64 | 0.42 | 0.71 |
| **Bio12** |  |  |  |  |  |  |  |  |  |  |  | 1.00 | 0.95 | 0.97 | -0.64 | 0.97 | 0.98 | 0.85 | 0.96 |
| **Bio13** |  |  |  |  |  |  |  |  |  |  |  |  | 1.00 | 0.88 | -0.41 | 0.99 | 0.88 | 0.79 | 0.89 |
| **Bio14** |  |  |  |  |  |  |  |  |  |  |  |  |  | 1.00 | -0.74 | 0.91 | 0.99 | 0.85 | 0.94 |
| **Bio15** |  |  |  |  |  |  |  |  |  |  |  |  |  |  | 1.00 | -0.47 | -0.73 | -0.56 | -0.62 |
| **Bio16** |  |  |  |  |  |  |  |  |  |  |  |  |  |  |  | 1.00 | 0.91 | 0.81 | 0.92 |
| **Bio17** |  |  |  |  |  |  |  |  |  |  |  |  |  |  |  |  | 1.00 | 0.82 | 0.96 |
| **Bio18** |  |  |  |  |  |  |  |  |  |  |  |  |  |  |  |  |  | 1.00 | 0.70 |
| **Bio19** |  |  |  |  |  |  |  |  |  |  |  |  |  |  |  |  |  |  | 1.00 |

**S1 File Table A. Correlation matrix of environmental variables**. Uncorrelated variables were selected based on a correlation coefficient of ≤0.80 (yellow). Variables above this threshold were removed (pink). Variables description is found in [29].

**S1 File Table B. Summary of model evaluations.** Results of ENMeval tuning experiments considering different feature classes (FC), regularization multipliers (RM), background (BCK), partition methods (jackknife, random-k-fold, block, checkerboar1) and evaluation metrics (∆AICc, AUC total, mean and variability for the AUC mean, AUC difference, and the mean and variability of omission rates [OR] considering 100% training occurrences [MTP, minimum training presence] and 90% of training occurrences [OR 10%]). For details see [41] and the main manuscript. We compare partition methods and metrics considering three calibration areas: (1) the invaded range of starry stonewort in United States (**M*_i_***) and (2) starry stonewort’s entire species range (**M*_g_***), both consider maximum dispersal potential; and (3) the calibration area considering limited dispersal potential (**M*_d_***) (Fig 2 in the main manuscript). BCK was explored with 5,000 vs. 10,000 points for **M*_i_*** and **M***_g_* while for **M*_d_*** only 10,000 BCK points were used due to the reduce extent that resulted in less than 5,000 BCK points. First, models were selected considering their fit to the data and therefore a ∆AICc = 0 (yellow). Then, to select the best combination of feature classes and regularization multiplier between different background or partitions we selected the model with the best predictive performance considering the other metrics employed. Rows are in ascendant order considering the lowest value of ∆AICc = 0.

***Calibration area No 1: Starry stonewort invaded range with maximum dispersal potential (~2,2000 km, M_i_)***

| ***n*** | **BCK** | **Partition** | **FC** | **RM** | **∆AICc** | **AUC test** | **AUC mean** | **AUC variance** | **AUC mean difference** | **AUC var. difference** | **OR mean 10%** | **OR var 10%** | **OR mean MTP** | **OR var MTP** |
| --- | --- | --- | --- | --- | --- | --- | --- | --- | --- | --- | --- | --- | --- | --- |
| 55 | 5,000 | Jakknife | LQ | 1 | 0.00 | 0.97 | 0.97 | 0.08 | 0.02 | 0.06 | 0.11 | 0.10 | 0.05 | 0.05 |
| 55 | 5,000 | Jakknife | LQ | 0.8 | 0.38 | 0.97 | 0.97 | 0.08 | 0.02 | 0.06 | 0.11 | 0.10 | 0.05 | 0.05 |
| 55 | 5,000 | Jakknife | LQ | 1.1 | 0.74 | 0.97 | 0.97 | 0.08 | 0.02 | 0.06 | 0.11 | 0.10 | 0.05 | 0.05 |
| 55 | 5,000 | Jakknife | LQ | 1.2 | 0.96 | 0.97 | 0.97 | 0.08 | 0.02 | 0.06 | 0.11 | 0.10 | 0.05 | 0.05 |
| 55 | 5,000 | Jakknife | LQ | 0.6 | 1.21 | 0.97 | 0.97 | 0.08 | 0.02 | 0.06 | 0.11 | 0.10 | 0.04 | 0.04 |
| 55 | 5,000 | Jakknife | LQ | 0.7 | 1.32 | 0.97 | 0.97 | 0.08 | 0.02 | 0.06 | 0.11 | 0.10 | 0.05 | 0.05 |
| 55 | 5,000 | Jakknife | LQ | 0.9 | 2.33 | 0.97 | 0.97 | 0.08 | 0.02 | 0.06 | 0.11 | 0.10 | 0.05 | 0.05 |
| 55 | 5,000 | Jakknife | LQ | 0.5 | 2.49 | 0.97 | 0.97 | 0.09 | 0.02 | 0.06 | 0.11 | 0.10 | 0.04 | 0.04 |
| 55 | 5,000 | Jakknife | LQ | 1.3 | 3.36 | 0.97 | 0.97 | 0.09 | 0.02 | 0.06 | 0.11 | 0.10 | 0.05 | 0.05 |
| 55 | 5,000 | Jakknife | LQ | 0.4 | 4.74 | 0.97 | 0.97 | 0.09 | 0.02 | 0.07 | 0.11 | 0.10 | 0.04 | 0.04 |
| 55 | 5,000 | Jakknife | LQ | 0.1 | 4.89 | 0.97 | 0.97 | 0.09 | 0.02 | 0.07 | 0.11 | 0.10 | 0.04 | 0.04 |
| 55 | 5,000 | Jakknife | LQ | 0.2 | 4.89 | 0.97 | 0.97 | 0.09 | 0.02 | 0.07 | 0.11 | 0.10 | 0.04 | 0.04 |
| 55 | 5,000 | Jakknife | LQ | 0.3 | 4.89 | 0.97 | 0.97 | 0.09 | 0.02 | 0.07 | 0.11 | 0.10 | 0.04 | 0.04 |
| 55 | 5,000 | Jakknife | LQ | 1.4 | 5.54 | 0.97 | 0.97 | 0.09 | 0.02 | 0.06 | 0.11 | 0.10 | 0.02 | 0.02 |
| 55 | 5,000 | Jakknife | LQHP | 1 | 6.39 | 0.98 | 0.97 | 0.06 | 0.01 | 0.04 | 0.15 | 0.13 | 0.07 | 0.07 |
| 55 | 5,000 | Jakknife | LQ | 1.5 | 7.04 | 0.97 | 0.96 | 0.09 | 0.02 | 0.06 | 0.11 | 0.10 | 0.02 | 0.02 |
| 55 | 5,000 | Jakknife | LQ | 1.6 | 8.84 | 0.97 | 0.96 | 0.09 | 0.02 | 0.06 | 0.11 | 0.10 | 0.02 | 0.02 |
| 55 | 5,000 | Jakknife | LQHP | 1.1 | 10.22 | 0.98 | 0.97 | 0.06 | 0.01 | 0.04 | 0.15 | 0.13 | 0.05 | 0.05 |
| 55 | 5,000 | Jakknife | LQ | 1.7 | 10.85 | 0.97 | 0.96 | 0.09 | 0.02 | 0.06 | 0.11 | 0.10 | 0.02 | 0.02 |
| 55 | 5,000 | Jakknife | LQ | 1.8 | 12.79 | 0.97 | 0.96 | 0.09 | 0.02 | 0.06 | 0.11 | 0.10 | 0.02 | 0.02 |
| 55 | 5,000 | Jakknife | LQ | 1.9 | 14.68 | 0.97 | 0.96 | 0.09 | 0.02 | 0.06 | 0.11 | 0.10 | 0.02 | 0.02 |
| 55 | 5,000 | Jakknife | LQ | 2 | 16.51 | 0.96 | 0.96 | 0.10 | 0.02 | 0.06 | 0.11 | 0.10 | 0.02 | 0.02 |
| 55 | 5,000 | Jakknife | LQHPT | 1.9 | 16.68 | 0.98 | 0.97 | 0.09 | 0.02 | 0.06 | 0.15 | 0.13 | 0.07 | 0.07 |
| 55 | 5,000 | Jakknife | LQHPT | 1.7 | 17.42 | 0.98 | 0.97 | 0.08 | 0.02 | 0.06 | 0.15 | 0.13 | 0.07 | 0.07 |
| 55 | 5,000 | Jakknife | LQHPT | 1.8 | 20.24 | 0.98 | 0.97 | 0.08 | 0.02 | 0.06 | 0.15 | 0.13 | 0.07 | 0.07 |
| 55 | 5,000 | Jakknife | LQH | 1.4 | 22.11 | 0.97 | 0.97 | 0.08 | 0.02 | 0.05 | 0.15 | 0.13 | 0.04 | 0.04 |
| 55 | 5,000 | Jakknife | LQHP | 1.6 | 23.19 | 0.97 | 0.97 | 0.07 | 0.01 | 0.04 | 0.15 | 0.13 | 0.05 | 0.05 |
| 55 | 5,000 | Jakknife | LQH | 1.9 | 24.48 | 0.97 | 0.97 | 0.08 | 0.02 | 0.06 | 0.15 | 0.13 | 0.02 | 0.02 |
| 55 | 5,000 | Jakknife | LQHPT | 1.6 | 25.87 | 0.98 | 0.97 | 0.08 | 0.02 | 0.06 | 0.15 | 0.13 | 0.09 | 0.08 |
| 55 | 5,000 | Jakknife | LQHP | 1.8 | 25.99 | 0.97 | 0.97 | 0.07 | 0.02 | 0.05 | 0.15 | 0.13 | 0.05 | 0.05 |
| 55 | 5,000 | Jakknife | LQH | 2 | 26.07 | 0.97 | 0.97 | 0.09 | 0.02 | 0.06 | 0.15 | 0.13 | 0.02 | 0.02 |
| 55 | 5,000 | Jakknife | LQH | 1.5 | 26.23 | 0.97 | 0.97 | 0.08 | 0.02 | 0.05 | 0.15 | 0.13 | 0.04 | 0.04 |
| 55 | 5,000 | Jakknife | LQH | 1.3 | 26.48 | 0.97 | 0.97 | 0.07 | 0.02 | 0.05 | 0.15 | 0.13 | 0.04 | 0.04 |
| 55 | 5,000 | Jakknife | LQHP | 1.5 | 27.50 | 0.97 | 0.97 | 0.07 | 0.01 | 0.04 | 0.15 | 0.13 | 0.05 | 0.05 |
| 55 | 5,000 | Jakknife | LQHP | 1.4 | 28.83 | 0.97 | 0.97 | 0.06 | 0.02 | 0.04 | 0.15 | 0.13 | 0.07 | 0.07 |
| 55 | 5,000 | Jakknife | LQHPT | 2 | 29.43 | 0.98 | 0.97 | 0.08 | 0.02 | 0.06 | 0.15 | 0.13 | 0.07 | 0.07 |
| 55 | 5,000 | Jakknife | LQHP | 1.7 | 30.27 | 0.97 | 0.97 | 0.07 | 0.02 | 0.05 | 0.15 | 0.13 | 0.05 | 0.05 |
| 55 | 5,000 | Jakknife | LQH | 1.8 | 31.39 | 0.97 | 0.97 | 0.08 | 0.02 | 0.06 | 0.15 | 0.13 | 0.02 | 0.02 |
| 55 | 5,000 | Jakknife | LQHP | 1.3 | 31.57 | 0.98 | 0.97 | 0.06 | 0.02 | 0.04 | 0.15 | 0.13 | 0.07 | 0.07 |
| 55 | 5,000 | Jakknife | LQH | 1.6 | 32.46 | 0.97 | 0.97 | 0.08 | 0.02 | 0.05 | 0.15 | 0.13 | 0.04 | 0.04 |
| 55 | 5,000 | Jakknife | LQHP | 1.2 | 34.50 | 0.98 | 0.97 | 0.06 | 0.01 | 0.04 | 0.15 | 0.13 | 0.05 | 0.05 |
| 55 | 5,000 | Jakknife | LQHP | 2 | 35.59 | 0.97 | 0.97 | 0.07 | 0.02 | 0.05 | 0.15 | 0.13 | 0.04 | 0.04 |
| 55 | 5,000 | Jakknife | LQHP | 0.9 | 37.72 | 0.98 | 0.97 | 0.06 | 0.01 | 0.04 | 0.15 | 0.13 | 0.05 | 0.05 |
| 55 | 5,000 | Jakknife | LQHP | 1.9 | 39.65 | 0.97 | 0.97 | 0.07 | 0.02 | 0.05 | 0.15 | 0.13 | 0.04 | 0.04 |
| 55 | 5,000 | Jakknife | LQH | 1.7 | 40.78 | 0.97 | 0.97 | 0.08 | 0.02 | 0.06 | 0.15 | 0.13 | 0.02 | 0.02 |
| 55 | 5,000 | Jakknife | LQH | 1.2 | 43.45 | 0.97 | 0.97 | 0.07 | 0.02 | 0.05 | 0.15 | 0.13 | 0.07 | 0.07 |
| 55 | 5,000 | Jakknife | LQHPT | 1.4 | 45.72 | 0.98 | 0.97 | 0.08 | 0.02 | 0.06 | 0.15 | 0.13 | 0.11 | 0.10 |
| 55 | 5,000 | Jakknife | LQH | 1.1 | 47.04 | 0.97 | 0.97 | 0.07 | 0.02 | 0.05 | 0.15 | 0.13 | 0.07 | 0.07 |
| 55 | 5,000 | Jakknife | LQHPT | 1.5 | 50.76 | 0.98 | 0.97 | 0.08 | 0.02 | 0.06 | 0.15 | 0.13 | 0.11 | 0.10 |
| 55 | 5,000 | Jakknife | LQHPT | 1.3 | 59.44 | 0.98 | 0.97 | 0.08 | 0.02 | 0.06 | 0.15 | 0.13 | 0.11 | 0.10 |
| 55 | 5,000 | Jakknife | LQHPT | 0.9 | 72.80 | 0.99 | 0.97 | 0.08 | 0.02 | 0.07 | 0.20 | 0.16 | 0.05 | 0.05 |
| 55 | 5,000 | Jakknife | LQHPT | 1.2 | 74.38 | 0.98 | 0.97 | 0.08 | 0.02 | 0.07 | 0.16 | 0.14 | 0.09 | 0.08 |
| 55 | 5,000 | Jakknife | LQHPT | 1.1 | 78.53 | 0.98 | 0.97 | 0.08 | 0.02 | 0.07 | 0.16 | 0.14 | 0.07 | 0.07 |
| 55 | 5,000 | Jakknife | LQHPT | 1 | 96.19 | 0.98 | 0.97 | 0.08 | 0.02 | 0.07 | 0.18 | 0.15 | 0.05 | 0.05 |
| 55 | 5,000 | Jakknife | LQHPT | 0.8 | 111.60 | 0.99 | 0.97 | 0.09 | 0.02 | 0.08 | 0.20 | 0.16 | 0.04 | 0.04 |
| 55 | 5,000 | Jakknife | H | 1.9 | 117.52 | 0.97 | 0.97 | 0.10 | 0.02 | 0.07 | 0.13 | 0.11 | 0.02 | 0.02 |
| 55 | 5,000 | Jakknife | LQHP | 0.8 | 124.85 | 0.98 | 0.97 | 0.06 | 0.01 | 0.04 | 0.15 | 0.13 | 0.04 | 0.04 |
| 55 | 5,000 | Jakknife | LQH | 1 | 127.05 | 0.98 | 0.97 | 0.07 | 0.02 | 0.05 | 0.15 | 0.13 | 0.07 | 0.07 |
| 55 | 5,000 | Jakknife | L | 0.1 | 140.86 | 0.90 | 0.89 | 0.36 | 0.03 | 0.20 | 0.11 | 0.10 | 0.02 | 0.02 |
| 55 | 5,000 | Jakknife | L | 0.2 | 140.86 | 0.90 | 0.89 | 0.36 | 0.03 | 0.20 | 0.11 | 0.10 | 0.02 | 0.02 |
| 55 | 5,000 | Jakknife | L | 0.3 | 140.86 | 0.90 | 0.89 | 0.36 | 0.03 | 0.20 | 0.11 | 0.10 | 0.02 | 0.02 |
| 55 | 5,000 | Jakknife | L | 0.4 | 140.86 | 0.90 | 0.89 | 0.36 | 0.03 | 0.20 | 0.11 | 0.10 | 0.02 | 0.02 |
| 55 | 5,000 | Jakknife | L | 0.5 | 140.86 | 0.90 | 0.89 | 0.36 | 0.03 | 0.20 | 0.11 | 0.10 | 0.02 | 0.02 |
| 55 | 5,000 | Jakknife | L | 0.6 | 141.09 | 0.90 | 0.89 | 0.36 | 0.03 | 0.20 | 0.11 | 0.10 | 0.02 | 0.02 |
| 55 | 5,000 | Jakknife | L | 0.8 | 141.26 | 0.90 | 0.89 | 0.36 | 0.03 | 0.20 | 0.11 | 0.10 | 0.02 | 0.02 |
| 55 | 5,000 | Jakknife | L | 0.7 | 141.47 | 0.90 | 0.89 | 0.36 | 0.03 | 0.20 | 0.11 | 0.10 | 0.02 | 0.02 |
| 55 | 5,000 | Jakknife | L | 0.9 | 141.65 | 0.90 | 0.89 | 0.35 | 0.03 | 0.19 | 0.11 | 0.10 | 0.02 | 0.02 |
| 55 | 5,000 | Jakknife | L | 1 | 142.00 | 0.90 | 0.89 | 0.35 | 0.03 | 0.19 | 0.11 | 0.10 | 0.02 | 0.02 |
| 55 | 5,000 | Jakknife | L | 1.1 | 142.56 | 0.90 | 0.89 | 0.34 | 0.03 | 0.18 | 0.11 | 0.10 | 0.02 | 0.02 |
| 55 | 5,000 | Jakknife | L | 1.2 | 143.35 | 0.90 | 0.89 | 0.33 | 0.03 | 0.18 | 0.11 | 0.10 | 0.02 | 0.02 |
| 55 | 5,000 | Jakknife | L | 1.3 | 144.31 | 0.90 | 0.89 | 0.32 | 0.03 | 0.17 | 0.11 | 0.10 | 0.02 | 0.02 |
| 55 | 5,000 | Jakknife | L | 1.6 | 144.78 | 0.89 | 0.89 | 0.29 | 0.03 | 0.14 | 0.11 | 0.10 | 0.04 | 0.04 |
| 55 | 5,000 | Jakknife | L | 1.4 | 145.61 | 0.90 | 0.89 | 0.31 | 0.03 | 0.16 | 0.11 | 0.10 | 0.04 | 0.04 |
| 55 | 5,000 | Jakknife | L | 1.7 | 145.63 | 0.89 | 0.89 | 0.28 | 0.03 | 0.14 | 0.11 | 0.10 | 0.02 | 0.02 |
| 55 | 5,000 | Jakknife | L | 1.8 | 146.50 | 0.89 | 0.89 | 0.28 | 0.03 | 0.14 | 0.11 | 0.10 | 0.02 | 0.02 |
| 55 | 5,000 | Jakknife | L | 1.5 | 146.57 | 0.89 | 0.89 | 0.29 | 0.03 | 0.15 | 0.11 | 0.10 | 0.04 | 0.04 |
| 55 | 5,000 | Jakknife | L | 1.9 | 147.41 | 0.89 | 0.89 | 0.27 | 0.03 | 0.13 | 0.13 | 0.11 | 0.02 | 0.02 |
| 55 | 5,000 | Jakknife | L | 2 | 148.41 | 0.89 | 0.89 | 0.27 | 0.03 | 0.13 | 0.13 | 0.11 | 0.02 | 0.02 |
| 55 | 5,000 | Jakknife | H | 2 | 242.88 | 0.97 | 0.97 | 0.10 | 0.02 | 0.07 | 0.11 | 0.10 | 0.02 | 0.02 |
| 55 | 5,000 | Jakknife | H | 1.8 | 292.32 | 0.97 | 0.97 | 0.09 | 0.02 | 0.06 | 0.15 | 0.13 | 0.02 | 0.02 |
| 55 | 5,000 | Jakknife | LQHP | 0.7 | 315.54 | 0.98 | 0.97 | 0.05 | 0.01 | 0.04 | 0.13 | 0.11 | 0.05 | 0.05 |
| 55 | 5,000 | Jakknife | LQH | 0.9 | 376.75 | 0.98 | 0.97 | 0.07 | 0.02 | 0.05 | 0.15 | 0.13 | 0.07 | 0.07 |
| 55 | 5,000 | Jakknife | LQH | 0.8 | 422.03 | 0.98 | 0.97 | 0.06 | 0.02 | 0.05 | 0.15 | 0.13 | 0.07 | 0.07 |
| 55 | 5,000 | Jakknife | LQHPT | 0.7 | 430.35 | 0.99 | 0.97 | 0.09 | 0.02 | 0.09 | 0.25 | 0.19 | 0.04 | 0.04 |
| 55 | 5,000 | Jakknife | H | 1.3 | 451.97 | 0.97 | 0.97 | 0.08 | 0.02 | 0.05 | 0.15 | 0.13 | 0.04 | 0.04 |
| 55 | 5,000 | Jakknife | H | 1.5 | 456.61 | 0.97 | 0.97 | 0.09 | 0.02 | 0.06 | 0.15 | 0.13 | 0.02 | 0.02 |
| 55 | 5,000 | Jakknife | H | 1.4 | 603.02 | 0.97 | 0.97 | 0.08 | 0.02 | 0.06 | 0.15 | 0.13 | 0.02 | 0.02 |
| 55 | 5,000 | Jakknife | LQHP | 0.6 | 660.52 | 0.98 | 0.98 | 0.05 | 0.01 | 0.04 | 0.13 | 0.11 | 0.05 | 0.05 |
| 55 | 5,000 | Jakknife | LQH | 0.7 | 667.88 | 0.98 | 0.97 | 0.06 | 0.01 | 0.04 | 0.15 | 0.13 | 0.05 | 0.05 |
| 55 | 5,000 | Jakknife | H | 1.6 | 854.61 | 0.97 | 0.97 | 0.09 | 0.02 | 0.06 | 0.15 | 0.13 | 0.02 | 0.02 |
| 55 | 5,000 | Jakknife | H | 1.7 | 1351.45 | 0.97 | 0.97 | 0.09 | 0.02 | 0.06 | 0.15 | 0.13 | 0.02 | 0.02 |
| 55 | 5,000 | Jakknife | LQH | 0.6 | 5751.47 | 0.98 | 0.98 | 0.05 | 0.01 | 0.04 | 0.15 | 0.13 | 0.05 | 0.05 |
| 55 | 5,000 | Jakknife | H | 0.1 | NA | 0.99 | 0.98 | 0.06 | 0.01 | 0.05 | 0.29 | 0.21 | 0.04 | 0.04 |
| 55 | 5,000 | Jakknife | LQH | 0.1 | NA | 0.99 | 0.98 | 0.06 | 0.01 | 0.05 | 0.31 | 0.22 | 0.04 | 0.04 |
| 55 | 5,000 | Jakknife | LQHP | 0.1 | NA | 0.99 | 0.98 | 0.06 | 0.01 | 0.06 | 0.31 | 0.22 | 0.04 | 0.04 |
| 55 | 5,000 | Jakknife | LQHPT | 0.1 | NA | 0.99 | 0.96 | 0.49 | 0.03 | 0.48 | 0.49 | 0.25 | 0.02 | 0.02 |
| 55 | 5,000 | Jakknife | H | 0.2 | NA | 0.99 | 0.98 | 0.04 | 0.01 | 0.04 | 0.20 | 0.16 | 0.04 | 0.04 |
| 55 | 5,000 | Jakknife | LQH | 0.2 | NA | 0.99 | 0.98 | 0.05 | 0.01 | 0.04 | 0.20 | 0.16 | 0.04 | 0.04 |
| 55 | 5,000 | Jakknife | LQHP | 0.2 | NA | 0.99 | 0.98 | 0.05 | 0.01 | 0.04 | 0.20 | 0.16 | 0.04 | 0.04 |
| 55 | 5,000 | Jakknife | LQHPT | 0.2 | NA | 0.99 | 0.96 | 0.47 | 0.03 | 0.46 | 0.47 | 0.25 | 0.02 | 0.02 |
| 55 | 5,000 | Jakknife | H | 0.3 | NA | 0.99 | 0.98 | 0.04 | 0.01 | 0.04 | 0.18 | 0.15 | 0.04 | 0.04 |
| 55 | 5,000 | Jakknife | LQH | 0.3 | NA | 0.99 | 0.98 | 0.04 | 0.01 | 0.04 | 0.18 | 0.15 | 0.04 | 0.04 |
| 55 | 5,000 | Jakknife | LQHP | 0.3 | NA | 0.99 | 0.98 | 0.05 | 0.01 | 0.04 | 0.18 | 0.15 | 0.04 | 0.04 |
| 55 | 5,000 | Jakknife | LQHPT | 0.3 | NA | 0.99 | 0.97 | 0.41 | 0.03 | 0.40 | 0.44 | 0.25 | 0.02 | 0.02 |
| 55 | 5,000 | Jakknife | H | 0.4 | NA | 0.98 | 0.98 | 0.04 | 0.01 | 0.04 | 0.16 | 0.14 | 0.04 | 0.04 |
| 55 | 5,000 | Jakknife | LQH | 0.4 | NA | 0.98 | 0.98 | 0.04 | 0.01 | 0.04 | 0.18 | 0.15 | 0.05 | 0.05 |
| 55 | 5,000 | Jakknife | LQHP | 0.4 | NA | 0.98 | 0.98 | 0.05 | 0.01 | 0.04 | 0.18 | 0.15 | 0.05 | 0.05 |
| 55 | 5,000 | Jakknife | LQHPT | 0.4 | NA | 0.99 | 0.97 | 0.40 | 0.03 | 0.39 | 0.44 | 0.25 | 0.02 | 0.02 |
| 55 | 5,000 | Jakknife | H | 0.5 | NA | 0.98 | 0.98 | 0.05 | 0.01 | 0.04 | 0.15 | 0.13 | 0.04 | 0.04 |
| 55 | 5,000 | Jakknife | LQH | 0.5 | NA | 0.98 | 0.98 | 0.05 | 0.01 | 0.04 | 0.15 | 0.13 | 0.05 | 0.05 |
| 55 | 5,000 | Jakknife | LQHP | 0.5 | NA | 0.98 | 0.98 | 0.05 | 0.01 | 0.04 | 0.15 | 0.13 | 0.05 | 0.05 |
| 55 | 5,000 | Jakknife | LQHPT | 0.5 | NA | 0.99 | 0.97 | 0.34 | 0.03 | 0.33 | 0.40 | 0.24 | 0.02 | 0.02 |
| 55 | 5,000 | Jakknife | H | 0.6 | NA | 0.98 | 0.98 | 0.05 | 0.01 | 0.04 | 0.16 | 0.14 | 0.04 | 0.04 |
| 55 | 5,000 | Jakknife | LQHPT | 0.6 | NA | 0.99 | 0.97 | 0.17 | 0.02 | 0.17 | 0.38 | 0.24 | 0.02 | 0.02 |
| 55 | 5,000 | Jakknife | H | 0.7 | NA | 0.98 | 0.97 | 0.06 | 0.01 | 0.04 | 0.16 | 0.14 | 0.04 | 0.04 |
| 55 | 5,000 | Jakknife | H | 0.8 | NA | 0.98 | 0.97 | 0.06 | 0.02 | 0.05 | 0.15 | 0.13 | 0.05 | 0.05 |
| 55 | 5,000 | Jakknife | H | 0.9 | NA | 0.98 | 0.97 | 0.07 | 0.02 | 0.05 | 0.15 | 0.13 | 0.05 | 0.05 |
| 55 | 5,000 | Jakknife | H | 1 | NA | 0.98 | 0.97 | 0.07 | 0.02 | 0.05 | 0.15 | 0.13 | 0.05 | 0.05 |
| 55 | 5,000 | Jakknife | H | 1.1 | NA | 0.97 | 0.97 | 0.08 | 0.02 | 0.05 | 0.15 | 0.13 | 0.05 | 0.05 |
| 55 | 5,000 | Jakknife | H | 1.2 | NA | 0.97 | 0.97 | 0.08 | 0.02 | 0.05 | 0.15 | 0.13 | 0.05 | 0.05 |
| 55 | 5,000 | Random-k-fold | LQ | 1 | 0.00 | 0.97 | 0.96 | 0.00 | 0.01 | 0.00 | 0.11 | 0.01 | 0.06 | 0.01 |
| 55 | 5,000 | Random-k-fold | LQ | 0.8 | 0.38 | 0.97 | 0.97 | 0.00 | 0.01 | 0.00 | 0.11 | 0.01 | 0.08 | 0.01 |
| 55 | 5,000 | Random-k-fold | LQ | 1.1 | 0.74 | 0.97 | 0.96 | 0.00 | 0.01 | 0.00 | 0.11 | 0.01 | 0.06 | 0.01 |
| 55 | 5,000 | Random-k-fold | LQ | 1.2 | 0.96 | 0.97 | 0.96 | 0.00 | 0.01 | 0.00 | 0.11 | 0.01 | 0.04 | 0.01 |
| 55 | 5,000 | Random-k-fold | LQ | 0.6 | 1.21 | 0.97 | 0.97 | 0.00 | 0.01 | 0.00 | 0.11 | 0.01 | 0.06 | 0.01 |
| 55 | 5,000 | Random-k-fold | LQ | 0.7 | 1.32 | 0.97 | 0.97 | 0.00 | 0.01 | 0.00 | 0.11 | 0.01 | 0.04 | 0.00 |
| 55 | 5,000 | Random-k-fold | LQ | 0.9 | 2.33 | 0.97 | 0.96 | 0.00 | 0.01 | 0.00 | 0.11 | 0.01 | 0.06 | 0.01 |
| 55 | 5,000 | Random-k-fold | LQ | 0.5 | 2.49 | 0.97 | 0.97 | 0.00 | 0.01 | 0.00 | 0.11 | 0.01 | 0.06 | 0.01 |
| 55 | 5,000 | Random-k-fold | LQ | 1.3 | 3.36 | 0.97 | 0.96 | 0.00 | 0.01 | 0.00 | 0.11 | 0.01 | 0.04 | 0.01 |
| 55 | 5,000 | Random-k-fold | LQ | 0.4 | 4.74 | 0.97 | 0.97 | 0.00 | 0.01 | 0.00 | 0.11 | 0.01 | 0.07 | 0.00 |
| 55 | 5,000 | Random-k-fold | LQ | 0.1 | 4.89 | 0.97 | 0.96 | 0.00 | 0.01 | 0.00 | 0.11 | 0.01 | 0.06 | 0.01 |
| 55 | 5,000 | Random-k-fold | LQ | 0.2 | 4.89 | 0.97 | 0.96 | 0.00 | 0.01 | 0.00 | 0.11 | 0.01 | 0.06 | 0.01 |
| 55 | 5,000 | Random-k-fold | LQ | 0.3 | 4.89 | 0.97 | 0.96 | 0.00 | 0.01 | 0.00 | 0.11 | 0.01 | 0.06 | 0.01 |
| 55 | 5,000 | Random-k-fold | LQ | 1.4 | 5.54 | 0.97 | 0.96 | 0.00 | 0.01 | 0.00 | 0.11 | 0.01 | 0.04 | 0.01 |
| 55 | 5,000 | Random-k-fold | LQHP | 1 | 6.39 | 0.98 | 0.97 | 0.00 | 0.01 | 0.00 | 0.15 | 0.00 | 0.09 | 0.01 |
| 55 | 5,000 | Random-k-fold | LQ | 1.5 | 7.04 | 0.97 | 0.96 | 0.00 | 0.01 | 0.00 | 0.11 | 0.01 | 0.04 | 0.01 |
| 55 | 5,000 | Random-k-fold | LQ | 1.6 | 8.84 | 0.97 | 0.96 | 0.00 | 0.01 | 0.00 | 0.11 | 0.01 | 0.04 | 0.01 |
| 55 | 5,000 | Random-k-fold | LQHP | 1.1 | 10.22 | 0.98 | 0.97 | 0.00 | 0.01 | 0.00 | 0.15 | 0.00 | 0.09 | 0.01 |
| 55 | 5,000 | Random-k-fold | LQ | 1.7 | 10.85 | 0.97 | 0.96 | 0.00 | 0.01 | 0.00 | 0.11 | 0.01 | 0.04 | 0.01 |
| 55 | 5,000 | Random-k-fold | LQ | 1.8 | 12.79 | 0.97 | 0.96 | 0.00 | 0.01 | 0.00 | 0.11 | 0.01 | 0.04 | 0.01 |
| 55 | 5,000 | Random-k-fold | LQ | 1.9 | 14.68 | 0.97 | 0.96 | 0.00 | 0.01 | 0.00 | 0.11 | 0.01 | 0.04 | 0.01 |
| 55 | 5,000 | Random-k-fold | LQ | 2 | 16.51 | 0.96 | 0.96 | 0.00 | 0.01 | 0.00 | 0.11 | 0.01 | 0.04 | 0.01 |
| 55 | 5,000 | Random-k-fold | LQHPT | 1.9 | 16.68 | 0.98 | 0.97 | 0.00 | 0.01 | 0.00 | 0.16 | 0.00 | 0.04 | 0.00 |
| 55 | 5,000 | Random-k-fold | LQHPT | 1.7 | 17.42 | 0.98 | 0.97 | 0.00 | 0.01 | 0.00 | 0.16 | 0.00 | 0.04 | 0.00 |
| 55 | 5,000 | Random-k-fold | LQHPT | 1.8 | 20.24 | 0.98 | 0.97 | 0.00 | 0.01 | 0.00 | 0.16 | 0.00 | 0.04 | 0.00 |
| 55 | 5,000 | Random-k-fold | LQH | 1.4 | 22.11 | 0.97 | 0.97 | 0.00 | 0.01 | 0.00 | 0.15 | 0.00 | 0.08 | 0.01 |
| 55 | 5,000 | Random-k-fold | LQHP | 1.6 | 23.19 | 0.97 | 0.97 | 0.00 | 0.01 | 0.00 | 0.13 | 0.01 | 0.08 | 0.01 |
| 55 | 5,000 | Random-k-fold | LQH | 1.9 | 24.48 | 0.97 | 0.96 | 0.00 | 0.01 | 0.00 | 0.13 | 0.01 | 0.06 | 0.01 |
| 55 | 5,000 | Random-k-fold | LQHPT | 1.6 | 25.87 | 0.98 | 0.97 | 0.00 | 0.01 | 0.00 | 0.16 | 0.00 | 0.07 | 0.00 |
| 55 | 5,000 | Random-k-fold | LQHP | 1.8 | 25.99 | 0.97 | 0.97 | 0.00 | 0.01 | 0.00 | 0.13 | 0.01 | 0.06 | 0.01 |
| 55 | 5,000 | Random-k-fold | LQH | 2 | 26.07 | 0.97 | 0.96 | 0.00 | 0.01 | 0.00 | 0.15 | 0.00 | 0.06 | 0.01 |
| 55 | 5,000 | Random-k-fold | LQH | 1.5 | 26.23 | 0.97 | 0.96 | 0.00 | 0.01 | 0.00 | 0.15 | 0.00 | 0.08 | 0.01 |
| 55 | 5,000 | Random-k-fold | LQH | 1.3 | 26.48 | 0.97 | 0.97 | 0.00 | 0.01 | 0.00 | 0.15 | 0.00 | 0.08 | 0.01 |
| 55 | 5,000 | Random-k-fold | LQHP | 1.5 | 27.50 | 0.97 | 0.97 | 0.00 | 0.01 | 0.00 | 0.13 | 0.01 | 0.08 | 0.01 |
| 55 | 5,000 | Random-k-fold | LQHP | 1.4 | 28.83 | 0.97 | 0.97 | 0.00 | 0.01 | 0.00 | 0.15 | 0.00 | 0.09 | 0.01 |
| 55 | 5,000 | Random-k-fold | LQHPT | 2 | 29.43 | 0.98 | 0.97 | 0.00 | 0.01 | 0.00 | 0.16 | 0.00 | 0.04 | 0.00 |
| 55 | 5,000 | Random-k-fold | LQHP | 1.7 | 30.27 | 0.97 | 0.97 | 0.00 | 0.01 | 0.00 | 0.13 | 0.01 | 0.08 | 0.01 |
| 55 | 5,000 | Random-k-fold | LQH | 1.8 | 31.39 | 0.97 | 0.96 | 0.00 | 0.01 | 0.00 | 0.13 | 0.01 | 0.06 | 0.01 |
| 55 | 5,000 | Random-k-fold | LQHP | 1.3 | 31.57 | 0.98 | 0.97 | 0.00 | 0.01 | 0.00 | 0.15 | 0.00 | 0.09 | 0.01 |
| 55 | 5,000 | Random-k-fold | LQH | 1.6 | 32.46 | 0.97 | 0.96 | 0.00 | 0.01 | 0.00 | 0.13 | 0.01 | 0.06 | 0.01 |
| 55 | 5,000 | Random-k-fold | LQHP | 1.2 | 34.50 | 0.98 | 0.97 | 0.00 | 0.01 | 0.00 | 0.15 | 0.00 | 0.09 | 0.01 |
| 55 | 5,000 | Random-k-fold | LQHP | 2 | 35.59 | 0.97 | 0.97 | 0.00 | 0.01 | 0.00 | 0.15 | 0.00 | 0.06 | 0.01 |
| 55 | 5,000 | Random-k-fold | LQHP | 0.9 | 37.72 | 0.98 | 0.97 | 0.00 | 0.01 | 0.00 | 0.15 | 0.00 | 0.09 | 0.01 |
| 55 | 5,000 | Random-k-fold | LQHP | 1.9 | 39.65 | 0.97 | 0.97 | 0.00 | 0.01 | 0.00 | 0.15 | 0.00 | 0.06 | 0.01 |
| 55 | 5,000 | Random-k-fold | LQH | 1.7 | 40.78 | 0.97 | 0.96 | 0.00 | 0.01 | 0.00 | 0.15 | 0.00 | 0.06 | 0.01 |
| 55 | 5,000 | Random-k-fold | LQH | 1.2 | 43.45 | 0.97 | 0.97 | 0.00 | 0.01 | 0.00 | 0.15 | 0.00 | 0.08 | 0.01 |
| 55 | 5,000 | Random-k-fold | LQHPT | 1.4 | 45.72 | 0.98 | 0.97 | 0.00 | 0.01 | 0.00 | 0.16 | 0.00 | 0.07 | 0.00 |
| 55 | 5,000 | Random-k-fold | LQH | 1.1 | 47.04 | 0.97 | 0.97 | 0.00 | 0.01 | 0.00 | 0.15 | 0.00 | 0.08 | 0.01 |
| 55 | 5,000 | Random-k-fold | LQHPT | 1.5 | 50.76 | 0.98 | 0.97 | 0.00 | 0.01 | 0.00 | 0.16 | 0.00 | 0.07 | 0.00 |
| 55 | 5,000 | Random-k-fold | LQHPT | 1.3 | 59.44 | 0.98 | 0.97 | 0.00 | 0.01 | 0.00 | 0.16 | 0.00 | 0.09 | 0.01 |
| 55 | 5,000 | Random-k-fold | LQHPT | 0.9 | 72.80 | 0.99 | 0.97 | 0.00 | 0.01 | 0.00 | 0.18 | 0.01 | 0.09 | 0.01 |
| 55 | 5,000 | Random-k-fold | LQHPT | 1.2 | 74.38 | 0.98 | 0.97 | 0.00 | 0.01 | 0.00 | 0.16 | 0.00 | 0.11 | 0.01 |
| 55 | 5,000 | Random-k-fold | LQHPT | 1.1 | 78.53 | 0.98 | 0.97 | 0.00 | 0.01 | 0.00 | 0.16 | 0.00 | 0.11 | 0.01 |
| 55 | 5,000 | Random-k-fold | LQHPT | 1 | 96.19 | 0.98 | 0.97 | 0.00 | 0.01 | 0.00 | 0.16 | 0.00 | 0.11 | 0.01 |
| 55 | 5,000 | Random-k-fold | LQHPT | 0.8 | 111.60 | 0.99 | 0.97 | 0.00 | 0.01 | 0.00 | 0.20 | 0.00 | 0.07 | 0.00 |
| 55 | 5,000 | Random-k-fold | H | 1.9 | 117.52 | 0.97 | 0.96 | 0.00 | 0.01 | 0.00 | 0.11 | 0.01 | 0.02 | 0.00 |
| 55 | 5,000 | Random-k-fold | LQHP | 0.8 | 124.85 | 0.98 | 0.97 | 0.00 | 0.01 | 0.00 | 0.16 | 0.00 | 0.07 | 0.00 |
| 55 | 5,000 | Random-k-fold | LQH | 1 | 127.05 | 0.98 | 0.97 | 0.00 | 0.01 | 0.00 | 0.15 | 0.00 | 0.09 | 0.01 |
| 55 | 5,000 | Random-k-fold | L | 0.1 | 140.86 | 0.90 | 0.89 | 0.00 | 0.01 | 0.00 | 0.09 | 0.00 | 0.00 | 0.00 |
| 55 | 5,000 | Random-k-fold | L | 0.2 | 140.86 | 0.90 | 0.89 | 0.00 | 0.01 | 0.00 | 0.09 | 0.00 | 0.00 | 0.00 |
| 55 | 5,000 | Random-k-fold | L | 0.3 | 140.86 | 0.90 | 0.89 | 0.00 | 0.01 | 0.00 | 0.09 | 0.00 | 0.00 | 0.00 |
| 55 | 5,000 | Random-k-fold | L | 0.4 | 140.86 | 0.90 | 0.89 | 0.00 | 0.01 | 0.00 | 0.09 | 0.00 | 0.00 | 0.00 |
| 55 | 5,000 | Random-k-fold | L | 0.5 | 140.86 | 0.90 | 0.89 | 0.00 | 0.01 | 0.00 | 0.09 | 0.00 | 0.00 | 0.00 |
| 55 | 5,000 | Random-k-fold | L | 0.6 | 141.09 | 0.90 | 0.89 | 0.00 | 0.01 | 0.00 | 0.09 | 0.00 | 0.00 | 0.00 |
| 55 | 5,000 | Random-k-fold | L | 0.8 | 141.26 | 0.90 | 0.89 | 0.00 | 0.01 | 0.00 | 0.09 | 0.00 | 0.00 | 0.00 |
| 55 | 5,000 | Random-k-fold | L | 0.7 | 141.47 | 0.90 | 0.89 | 0.00 | 0.01 | 0.00 | 0.09 | 0.00 | 0.00 | 0.00 |
| 55 | 5,000 | Random-k-fold | L | 0.9 | 141.65 | 0.90 | 0.89 | 0.00 | 0.01 | 0.00 | 0.09 | 0.00 | 0.00 | 0.00 |
| 55 | 5,000 | Random-k-fold | L | 1 | 142.00 | 0.90 | 0.89 | 0.00 | 0.01 | 0.00 | 0.13 | 0.00 | 0.00 | 0.00 |
| 55 | 5,000 | Random-k-fold | L | 1.1 | 142.56 | 0.90 | 0.89 | 0.00 | 0.01 | 0.00 | 0.13 | 0.00 | 0.00 | 0.00 |
| 55 | 5,000 | Random-k-fold | L | 1.2 | 143.35 | 0.90 | 0.89 | 0.00 | 0.01 | 0.00 | 0.13 | 0.00 | 0.02 | 0.00 |
| 55 | 5,000 | Random-k-fold | L | 1.3 | 144.31 | 0.90 | 0.89 | 0.00 | 0.01 | 0.00 | 0.13 | 0.00 | 0.02 | 0.00 |
| 55 | 5,000 | Random-k-fold | L | 1.6 | 144.78 | 0.89 | 0.89 | 0.00 | 0.01 | 0.00 | 0.14 | 0.01 | 0.02 | 0.00 |
| 55 | 5,000 | Random-k-fold | L | 1.4 | 145.61 | 0.90 | 0.89 | 0.00 | 0.01 | 0.00 | 0.14 | 0.01 | 0.02 | 0.00 |
| 55 | 5,000 | Random-k-fold | L | 1.7 | 145.63 | 0.89 | 0.89 | 0.00 | 0.01 | 0.00 | 0.11 | 0.01 | 0.02 | 0.00 |
| 55 | 5,000 | Random-k-fold | L | 1.8 | 146.50 | 0.89 | 0.89 | 0.00 | 0.01 | 0.00 | 0.11 | 0.01 | 0.02 | 0.00 |
| 55 | 5,000 | Random-k-fold | L | 1.5 | 146.57 | 0.89 | 0.89 | 0.00 | 0.01 | 0.00 | 0.14 | 0.01 | 0.02 | 0.00 |
| 55 | 5,000 | Random-k-fold | L | 1.9 | 147.41 | 0.89 | 0.88 | 0.00 | 0.01 | 0.00 | 0.11 | 0.01 | 0.02 | 0.00 |
| 55 | 5,000 | Random-k-fold | L | 2 | 148.41 | 0.89 | 0.88 | 0.00 | 0.01 | 0.00 | 0.13 | 0.00 | 0.02 | 0.00 |
| 55 | 5,000 | Random-k-fold | H | 2 | 242.88 | 0.97 | 0.96 | 0.00 | 0.01 | 0.00 | 0.11 | 0.01 | 0.02 | 0.00 |
| 55 | 5,000 | Random-k-fold | H | 1.8 | 292.32 | 0.97 | 0.96 | 0.00 | 0.01 | 0.00 | 0.13 | 0.01 | 0.02 | 0.00 |
| 55 | 5,000 | Random-k-fold | LQHP | 0.7 | 315.54 | 0.98 | 0.97 | 0.00 | 0.01 | 0.00 | 0.16 | 0.00 | 0.07 | 0.00 |
| 55 | 5,000 | Random-k-fold | LQH | 0.9 | 376.75 | 0.98 | 0.97 | 0.00 | 0.01 | 0.00 | 0.16 | 0.00 | 0.09 | 0.01 |
| 55 | 5,000 | Random-k-fold | LQH | 0.8 | 422.03 | 0.98 | 0.97 | 0.00 | 0.01 | 0.00 | 0.16 | 0.00 | 0.09 | 0.01 |
| 55 | 5,000 | Random-k-fold | LQHPT | 0.7 | 430.35 | 0.99 | 0.97 | 0.00 | 0.01 | 0.00 | 0.22 | 0.00 | 0.05 | 0.00 |
| 55 | 5,000 | Random-k-fold | H | 1.3 | 451.97 | 0.97 | 0.97 | 0.00 | 0.01 | 0.00 | 0.13 | 0.01 | 0.04 | 0.00 |
| 55 | 5,000 | Random-k-fold | H | 1.5 | 456.61 | 0.97 | 0.97 | 0.00 | 0.01 | 0.00 | 0.13 | 0.01 | 0.04 | 0.00 |
| 55 | 5,000 | Random-k-fold | H | 1.4 | 603.02 | 0.97 | 0.97 | 0.00 | 0.01 | 0.00 | 0.13 | 0.01 | 0.04 | 0.00 |
| 55 | 5,000 | Random-k-fold | LQHP | 0.6 | 660.52 | 0.98 | 0.97 | 0.00 | 0.01 | 0.00 | 0.15 | 0.00 | 0.07 | 0.00 |
| 55 | 5,000 | Random-k-fold | LQH | 0.7 | 667.88 | 0.98 | 0.97 | 0.00 | 0.01 | 0.00 | 0.15 | 0.00 | 0.09 | 0.01 |
| 55 | 5,000 | Random-k-fold | H | 1.6 | 854.61 | 0.97 | 0.96 | 0.00 | 0.01 | 0.00 | 0.13 | 0.01 | 0.04 | 0.00 |
| 55 | 5,000 | Random-k-fold | H | 1.7 | 1351.45 | 0.97 | 0.96 | 0.00 | 0.01 | 0.00 | 0.13 | 0.01 | 0.04 | 0.00 |
| 55 | 5,000 | Random-k-fold | LQH | 0.6 | 5751.47 | 0.98 | 0.97 | 0.00 | 0.01 | 0.00 | 0.15 | 0.00 | 0.09 | 0.01 |
| 55 | 5,000 | Random-k-fold | H | 0.1 | NA | 0.99 | 0.98 | 0.00 | 0.01 | 0.00 | 0.22 | 0.00 | 0.05 | 0.00 |
| 55 | 5,000 | Random-k-fold | LQH | 0.1 | NA | 0.99 | 0.98 | 0.00 | 0.01 | 0.00 | 0.20 | 0.01 | 0.05 | 0.00 |
| 55 | 5,000 | Random-k-fold | LQHP | 0.1 | NA | 0.99 | 0.98 | 0.00 | 0.01 | 0.00 | 0.24 | 0.01 | 0.05 | 0.00 |
| 55 | 5,000 | Random-k-fold | LQHPT | 0.1 | NA | 0.99 | 0.95 | 0.00 | 0.04 | 0.00 | 0.60 | 0.01 | 0.13 | 0.06 |
| 55 | 5,000 | Random-k-fold | H | 0.2 | NA | 0.99 | 0.98 | 0.00 | 0.01 | 0.00 | 0.20 | 0.01 | 0.04 | 0.00 |
| 55 | 5,000 | Random-k-fold | LQH | 0.2 | NA | 0.99 | 0.98 | 0.00 | 0.01 | 0.00 | 0.20 | 0.01 | 0.04 | 0.00 |
| 55 | 5,000 | Random-k-fold | LQHP | 0.2 | NA | 0.99 | 0.98 | 0.00 | 0.01 | 0.00 | 0.20 | 0.01 | 0.05 | 0.00 |
| 55 | 5,000 | Random-k-fold | LQHPT | 0.2 | NA | 0.99 | 0.95 | 0.00 | 0.04 | 0.00 | 0.59 | 0.02 | 0.11 | 0.05 |
| 55 | 5,000 | Random-k-fold | H | 0.3 | NA | 0.99 | 0.98 | 0.00 | 0.01 | 0.00 | 0.18 | 0.00 | 0.04 | 0.00 |
| 55 | 5,000 | Random-k-fold | LQH | 0.3 | NA | 0.99 | 0.98 | 0.00 | 0.01 | 0.00 | 0.22 | 0.00 | 0.04 | 0.00 |
| 55 | 5,000 | Random-k-fold | LQHP | 0.3 | NA | 0.99 | 0.98 | 0.00 | 0.01 | 0.00 | 0.24 | 0.01 | 0.04 | 0.00 |
| 55 | 5,000 | Random-k-fold | LQHPT | 0.3 | NA | 0.99 | 0.95 | 0.00 | 0.04 | 0.00 | 0.51 | 0.06 | 0.07 | 0.02 |
| 55 | 5,000 | Random-k-fold | H | 0.4 | NA | 0.98 | 0.98 | 0.00 | 0.01 | 0.00 | 0.20 | 0.00 | 0.05 | 0.00 |
| 55 | 5,000 | Random-k-fold | LQH | 0.4 | NA | 0.98 | 0.98 | 0.00 | 0.01 | 0.00 | 0.18 | 0.00 | 0.05 | 0.00 |
| 55 | 5,000 | Random-k-fold | LQHP | 0.4 | NA | 0.98 | 0.98 | 0.00 | 0.01 | 0.00 | 0.20 | 0.00 | 0.05 | 0.00 |
| 55 | 5,000 | Random-k-fold | LQHPT | 0.4 | NA | 0.99 | 0.96 | 0.00 | 0.03 | 0.00 | 0.48 | 0.04 | 0.07 | 0.01 |
| 55 | 5,000 | Random-k-fold | H | 0.5 | NA | 0.98 | 0.97 | 0.00 | 0.01 | 0.00 | 0.20 | 0.00 | 0.09 | 0.01 |
| 55 | 5,000 | Random-k-fold | LQH | 0.5 | NA | 0.98 | 0.97 | 0.00 | 0.01 | 0.00 | 0.18 | 0.00 | 0.07 | 0.00 |
| 55 | 5,000 | Random-k-fold | LQHP | 0.5 | NA | 0.98 | 0.97 | 0.00 | 0.01 | 0.00 | 0.16 | 0.00 | 0.07 | 0.00 |
| 55 | 5,000 | Random-k-fold | LQHPT | 0.5 | NA | 0.99 | 0.97 | 0.00 | 0.02 | 0.00 | 0.44 | 0.04 | 0.07 | 0.01 |
| 55 | 5,000 | Random-k-fold | H | 0.6 | NA | 0.98 | 0.97 | 0.00 | 0.01 | 0.00 | 0.16 | 0.00 | 0.09 | 0.01 |
| 55 | 5,000 | Random-k-fold | LQHPT | 0.6 | NA | 0.99 | 0.97 | 0.00 | 0.02 | 0.00 | 0.24 | 0.01 | 0.07 | 0.00 |
| 55 | 5,000 | Random-k-fold | H | 0.7 | NA | 0.98 | 0.97 | 0.00 | 0.01 | 0.00 | 0.16 | 0.00 | 0.09 | 0.01 |
| 55 | 5,000 | Random-k-fold | H | 0.8 | NA | 0.98 | 0.97 | 0.00 | 0.01 | 0.00 | 0.16 | 0.00 | 0.09 | 0.01 |
| 55 | 5,000 | Random-k-fold | H | 0.9 | NA | 0.98 | 0.97 | 0.00 | 0.01 | 0.00 | 0.16 | 0.00 | 0.07 | 0.00 |
| 55 | 5,000 | Random-k-fold | H | 1 | NA | 0.98 | 0.97 | 0.00 | 0.01 | 0.00 | 0.15 | 0.00 | 0.07 | 0.00 |
| 55 | 5,000 | Random-k-fold | H | 1.1 | NA | 0.97 | 0.97 | 0.00 | 0.01 | 0.00 | 0.13 | 0.01 | 0.04 | 0.00 |
| 55 | 5,000 | Random-k-fold | H | 1.2 | NA | 0.97 | 0.97 | 0.00 | 0.01 | 0.00 | 0.13 | 0.01 | 0.04 | 0.00 |
| 55 | 5,000 | Block | LQ | 1 | 0.00 | 0.97 | 0.96 | 0.00 | 0.01 | 0.00 | 0.13 | 0.06 | 0.11 | 0.05 |
| 55 | 5,000 | Block | LQ | 0.8 | 0.38 | 0.97 | 0.96 | 0.00 | 0.01 | 0.00 | 0.13 | 0.06 | 0.13 | 0.06 |
| 55 | 5,000 | Block | LQ | 1.1 | 0.74 | 0.97 | 0.96 | 0.00 | 0.01 | 0.00 | 0.13 | 0.06 | 0.05 | 0.01 |
| 55 | 5,000 | Block | LQ | 1.2 | 0.96 | 0.97 | 0.96 | 0.00 | 0.00 | 0.00 | 0.11 | 0.05 | 0.04 | 0.01 |
| 55 | 5,000 | Block | LQ | 0.6 | 1.21 | 0.97 | 0.96 | 0.00 | 0.01 | 0.00 | 0.13 | 0.06 | 0.13 | 0.06 |
| 55 | 5,000 | Block | LQ | 0.7 | 1.32 | 0.97 | 0.96 | 0.00 | 0.01 | 0.00 | 0.13 | 0.06 | 0.13 | 0.06 |
| 55 | 5,000 | Block | LQ | 0.9 | 2.33 | 0.97 | 0.96 | 0.00 | 0.01 | 0.00 | 0.13 | 0.06 | 0.13 | 0.06 |
| 55 | 5,000 | Block | LQ | 0.5 | 2.49 | 0.97 | 0.96 | 0.00 | 0.02 | 0.00 | 0.13 | 0.06 | 0.13 | 0.06 |
| 55 | 5,000 | Block | LQ | 1.3 | 3.36 | 0.97 | 0.96 | 0.00 | 0.00 | 0.00 | 0.07 | 0.02 | 0.04 | 0.01 |
| 55 | 5,000 | Block | LQ | 0.4 | 4.74 | 0.97 | 0.95 | 0.00 | 0.02 | 0.00 | 0.14 | 0.08 | 0.13 | 0.06 |
| 55 | 5,000 | Block | LQ | 0.1 | 4.89 | 0.97 | 0.95 | 0.00 | 0.02 | 0.00 | 0.14 | 0.08 | 0.13 | 0.06 |
| 55 | 5,000 | Block | LQ | 0.2 | 4.89 | 0.97 | 0.95 | 0.00 | 0.02 | 0.00 | 0.14 | 0.08 | 0.13 | 0.06 |
| 55 | 5,000 | Block | LQ | 0.3 | 4.89 | 0.97 | 0.95 | 0.00 | 0.02 | 0.00 | 0.14 | 0.08 | 0.13 | 0.06 |
| 55 | 5,000 | Block | LQ | 1.4 | 5.54 | 0.97 | 0.96 | 0.00 | 0.00 | 0.00 | 0.07 | 0.02 | 0.04 | 0.01 |
| 55 | 5,000 | Block | LQHP | 1 | 6.39 | 0.98 | 0.97 | 0.00 | 0.01 | 0.00 | 0.14 | 0.06 | 0.05 | 0.01 |
| 55 | 5,000 | Block | LQ | 1.5 | 7.04 | 0.97 | 0.96 | 0.00 | 0.00 | 0.00 | 0.07 | 0.02 | 0.04 | 0.01 |
| 55 | 5,000 | Block | LQ | 1.6 | 8.84 | 0.97 | 0.96 | 0.00 | 0.00 | 0.00 | 0.07 | 0.02 | 0.04 | 0.01 |
| 55 | 5,000 | Block | LQHP | 1.1 | 10.22 | 0.98 | 0.97 | 0.00 | 0.01 | 0.00 | 0.13 | 0.06 | 0.05 | 0.01 |
| 55 | 5,000 | Block | LQ | 1.7 | 10.85 | 0.97 | 0.96 | 0.00 | 0.00 | 0.00 | 0.07 | 0.02 | 0.02 | 0.00 |
| 55 | 5,000 | Block | LQ | 1.8 | 12.79 | 0.97 | 0.96 | 0.00 | 0.00 | 0.00 | 0.05 | 0.01 | 0.02 | 0.00 |
| 55 | 5,000 | Block | LQ | 1.9 | 14.68 | 0.97 | 0.96 | 0.00 | 0.00 | 0.00 | 0.05 | 0.01 | 0.02 | 0.00 |
| 55 | 5,000 | Block | LQ | 2 | 16.51 | 0.96 | 0.96 | 0.00 | 0.00 | 0.00 | 0.04 | 0.01 | 0.02 | 0.00 |
| 55 | 5,000 | Block | LQHPT | 1.9 | 16.68 | 0.98 | 0.96 | 0.00 | 0.01 | 0.00 | 0.13 | 0.06 | 0.07 | 0.02 |
| 55 | 5,000 | Block | LQHPT | 1.7 | 17.42 | 0.98 | 0.96 | 0.00 | 0.01 | 0.00 | 0.14 | 0.06 | 0.05 | 0.01 |
| 55 | 5,000 | Block | LQHPT | 1.8 | 20.24 | 0.98 | 0.96 | 0.00 | 0.01 | 0.00 | 0.14 | 0.06 | 0.09 | 0.03 |
| 55 | 5,000 | Block | LQH | 1.4 | 22.11 | 0.97 | 0.96 | 0.00 | 0.01 | 0.00 | 0.13 | 0.06 | 0.11 | 0.05 |
| 55 | 5,000 | Block | LQHP | 1.6 | 23.19 | 0.97 | 0.97 | 0.00 | 0.00 | 0.00 | 0.05 | 0.01 | 0.00 | 0.00 |
| 55 | 5,000 | Block | LQH | 1.9 | 24.48 | 0.97 | 0.96 | 0.00 | 0.01 | 0.00 | 0.13 | 0.06 | 0.02 | 0.00 |
| 55 | 5,000 | Block | LQHPT | 1.6 | 25.87 | 0.98 | 0.96 | 0.00 | 0.01 | 0.00 | 0.14 | 0.06 | 0.09 | 0.03 |
| 55 | 5,000 | Block | LQHP | 1.8 | 25.99 | 0.97 | 0.97 | 0.00 | 0.00 | 0.00 | 0.07 | 0.02 | 0.00 | 0.00 |
| 55 | 5,000 | Block | LQH | 2 | 26.07 | 0.97 | 0.96 | 0.00 | 0.01 | 0.00 | 0.13 | 0.06 | 0.02 | 0.00 |
| 55 | 5,000 | Block | LQH | 1.5 | 26.23 | 0.97 | 0.96 | 0.00 | 0.01 | 0.00 | 0.13 | 0.06 | 0.05 | 0.01 |
| 55 | 5,000 | Block | LQH | 1.3 | 26.48 | 0.97 | 0.96 | 0.00 | 0.01 | 0.00 | 0.13 | 0.06 | 0.11 | 0.05 |
| 55 | 5,000 | Block | LQHP | 1.5 | 27.50 | 0.97 | 0.97 | 0.00 | 0.01 | 0.00 | 0.07 | 0.02 | 0.02 | 0.00 |
| 55 | 5,000 | Block | LQHP | 1.4 | 28.83 | 0.97 | 0.97 | 0.00 | 0.01 | 0.00 | 0.11 | 0.05 | 0.04 | 0.01 |
| 55 | 5,000 | Block | LQHPT | 2 | 29.43 | 0.98 | 0.96 | 0.00 | 0.01 | 0.00 | 0.11 | 0.05 | 0.05 | 0.01 |
| 55 | 5,000 | Block | LQHP | 1.7 | 30.27 | 0.97 | 0.97 | 0.00 | 0.01 | 0.00 | 0.11 | 0.05 | 0.00 | 0.00 |
| 55 | 5,000 | Block | LQH | 1.8 | 31.39 | 0.97 | 0.96 | 0.00 | 0.01 | 0.00 | 0.13 | 0.06 | 0.07 | 0.02 |
| 55 | 5,000 | Block | LQHP | 1.3 | 31.57 | 0.98 | 0.97 | 0.00 | 0.01 | 0.00 | 0.11 | 0.05 | 0.04 | 0.01 |
| 55 | 5,000 | Block | LQH | 1.6 | 32.46 | 0.97 | 0.96 | 0.00 | 0.01 | 0.00 | 0.13 | 0.06 | 0.09 | 0.03 |
| 55 | 5,000 | Block | LQHP | 1.2 | 34.50 | 0.98 | 0.97 | 0.00 | 0.01 | 0.00 | 0.13 | 0.06 | 0.05 | 0.01 |
| 55 | 5,000 | Block | LQHP | 2 | 35.59 | 0.97 | 0.97 | 0.00 | 0.01 | 0.00 | 0.07 | 0.02 | 0.00 | 0.00 |
| 55 | 5,000 | Block | LQHP | 0.9 | 37.72 | 0.98 | 0.97 | 0.00 | 0.01 | 0.00 | 0.13 | 0.06 | 0.05 | 0.01 |
| 55 | 5,000 | Block | LQHP | 1.9 | 39.65 | 0.97 | 0.97 | 0.00 | 0.01 | 0.00 | 0.07 | 0.02 | 0.00 | 0.00 |
| 55 | 5,000 | Block | LQH | 1.7 | 40.78 | 0.97 | 0.96 | 0.00 | 0.01 | 0.00 | 0.13 | 0.06 | 0.04 | 0.01 |
| 55 | 5,000 | Block | LQH | 1.2 | 43.45 | 0.97 | 0.96 | 0.00 | 0.01 | 0.00 | 0.13 | 0.06 | 0.13 | 0.06 |
| 55 | 5,000 | Block | LQHPT | 1.4 | 45.72 | 0.98 | 0.96 | 0.00 | 0.01 | 0.00 | 0.16 | 0.06 | 0.09 | 0.03 |
| 55 | 5,000 | Block | LQH | 1.1 | 47.04 | 0.97 | 0.96 | 0.00 | 0.01 | 0.00 | 0.14 | 0.06 | 0.13 | 0.06 |
| 55 | 5,000 | Block | LQHPT | 1.5 | 50.76 | 0.98 | 0.96 | 0.00 | 0.01 | 0.00 | 0.14 | 0.06 | 0.09 | 0.03 |
| 55 | 5,000 | Block | LQHPT | 1.3 | 59.44 | 0.98 | 0.96 | 0.00 | 0.01 | 0.00 | 0.18 | 0.06 | 0.09 | 0.03 |
| 55 | 5,000 | Block | LQHPT | 0.9 | 72.80 | 0.99 | 0.96 | 0.00 | 0.02 | 0.00 | 0.25 | 0.07 | 0.13 | 0.04 |
| 55 | 5,000 | Block | LQHPT | 1.2 | 74.38 | 0.98 | 0.96 | 0.00 | 0.02 | 0.00 | 0.18 | 0.06 | 0.09 | 0.03 |
| 55 | 5,000 | Block | LQHPT | 1.1 | 78.53 | 0.98 | 0.96 | 0.00 | 0.02 | 0.00 | 0.18 | 0.06 | 0.11 | 0.03 |
| 55 | 5,000 | Block | LQHPT | 1 | 96.19 | 0.98 | 0.96 | 0.00 | 0.02 | 0.00 | 0.20 | 0.06 | 0.11 | 0.03 |
| 55 | 5,000 | Block | LQHPT | 0.8 | 111.60 | 0.99 | 0.96 | 0.00 | 0.02 | 0.00 | 0.27 | 0.08 | 0.13 | 0.04 |
| 55 | 5,000 | Block | H | 1.9 | 117.52 | 0.97 | 0.96 | 0.00 | 0.01 | 0.00 | 0.13 | 0.06 | 0.02 | 0.00 |
| 55 | 5,000 | Block | LQHP | 0.8 | 124.85 | 0.98 | 0.97 | 0.00 | 0.01 | 0.00 | 0.14 | 0.06 | 0.05 | 0.01 |
| 55 | 5,000 | Block | LQH | 1 | 127.05 | 0.98 | 0.96 | 0.00 | 0.01 | 0.00 | 0.16 | 0.05 | 0.13 | 0.06 |
| 55 | 5,000 | Block | L | 0.1 | 140.86 | 0.90 | 0.88 | 0.00 | 0.00 | 0.00 | 0.02 | 0.00 | 0.00 | 0.00 |
| 55 | 5,000 | Block | L | 0.2 | 140.86 | 0.90 | 0.88 | 0.00 | 0.00 | 0.00 | 0.02 | 0.00 | 0.00 | 0.00 |
| 55 | 5,000 | Block | L | 0.3 | 140.86 | 0.90 | 0.88 | 0.00 | 0.00 | 0.00 | 0.02 | 0.00 | 0.00 | 0.00 |
| 55 | 5,000 | Block | L | 0.4 | 140.86 | 0.90 | 0.88 | 0.00 | 0.00 | 0.00 | 0.02 | 0.00 | 0.00 | 0.00 |
| 55 | 5,000 | Block | L | 0.5 | 140.86 | 0.90 | 0.88 | 0.00 | 0.00 | 0.00 | 0.02 | 0.00 | 0.00 | 0.00 |
| 55 | 5,000 | Block | L | 0.6 | 141.09 | 0.90 | 0.88 | 0.00 | 0.00 | 0.00 | 0.02 | 0.00 | 0.00 | 0.00 |
| 55 | 5,000 | Block | L | 0.8 | 141.26 | 0.90 | 0.88 | 0.00 | 0.00 | 0.00 | 0.02 | 0.00 | 0.00 | 0.00 |
| 55 | 5,000 | Block | L | 0.7 | 141.47 | 0.90 | 0.88 | 0.00 | 0.00 | 0.00 | 0.02 | 0.00 | 0.00 | 0.00 |
| 55 | 5,000 | Block | L | 0.9 | 141.65 | 0.90 | 0.88 | 0.00 | 0.00 | 0.00 | 0.02 | 0.00 | 0.00 | 0.00 |
| 55 | 5,000 | Block | L | 1 | 142.00 | 0.90 | 0.88 | 0.00 | 0.00 | 0.00 | 0.02 | 0.00 | 0.00 | 0.00 |
| 55 | 5,000 | Block | L | 1.1 | 142.56 | 0.90 | 0.88 | 0.00 | 0.00 | 0.00 | 0.02 | 0.00 | 0.00 | 0.00 |
| 55 | 5,000 | Block | L | 1.2 | 143.35 | 0.90 | 0.88 | 0.00 | 0.00 | 0.00 | 0.02 | 0.00 | 0.00 | 0.00 |
| 55 | 5,000 | Block | L | 1.3 | 144.31 | 0.90 | 0.88 | 0.00 | 0.00 | 0.00 | 0.02 | 0.00 | 0.00 | 0.00 |
| 55 | 5,000 | Block | L | 1.6 | 144.78 | 0.89 | 0.88 | 0.00 | 0.00 | 0.00 | 0.00 | 0.00 | 0.00 | 0.00 |
| 55 | 5,000 | Block | L | 1.4 | 145.61 | 0.90 | 0.88 | 0.00 | 0.00 | 0.00 | 0.02 | 0.00 | 0.00 | 0.00 |
| 55 | 5,000 | Block | L | 1.7 | 145.63 | 0.89 | 0.88 | 0.00 | 0.00 | 0.00 | 0.00 | 0.00 | 0.00 | 0.00 |
| 55 | 5,000 | Block | L | 1.8 | 146.50 | 0.89 | 0.87 | 0.00 | 0.00 | 0.00 | 0.00 | 0.00 | 0.00 | 0.00 |
| 55 | 5,000 | Block | L | 1.5 | 146.57 | 0.89 | 0.88 | 0.00 | 0.00 | 0.00 | 0.02 | 0.00 | 0.00 | 0.00 |
| 55 | 5,000 | Block | L | 1.9 | 147.41 | 0.89 | 0.87 | 0.00 | 0.00 | 0.00 | 0.00 | 0.00 | 0.00 | 0.00 |
| 55 | 5,000 | Block | L | 2 | 148.41 | 0.89 | 0.87 | 0.00 | 0.00 | 0.00 | 0.00 | 0.00 | 0.00 | 0.00 |
| 55 | 5,000 | Block | H | 2 | 242.88 | 0.97 | 0.96 | 0.00 | 0.01 | 0.00 | 0.13 | 0.06 | 0.02 | 0.00 |
| 55 | 5,000 | Block | H | 1.8 | 292.32 | 0.97 | 0.96 | 0.00 | 0.01 | 0.00 | 0.13 | 0.06 | 0.02 | 0.00 |
| 55 | 5,000 | Block | LQHP | 0.7 | 315.54 | 0.98 | 0.97 | 0.00 | 0.01 | 0.00 | 0.14 | 0.06 | 0.05 | 0.01 |
| 55 | 5,000 | Block | LQH | 0.9 | 376.75 | 0.98 | 0.97 | 0.00 | 0.01 | 0.00 | 0.16 | 0.05 | 0.13 | 0.06 |
| 55 | 5,000 | Block | LQH | 0.8 | 422.03 | 0.98 | 0.96 | 0.00 | 0.01 | 0.00 | 0.18 | 0.05 | 0.13 | 0.06 |
| 55 | 5,000 | Block | LQHPT | 0.7 | 430.35 | 0.99 | 0.96 | 0.00 | 0.03 | 0.00 | 0.31 | 0.08 | 0.11 | 0.03 |
| 55 | 5,000 | Block | H | 1.3 | 451.97 | 0.97 | 0.97 | 0.00 | 0.00 | 0.00 | 0.09 | 0.03 | 0.00 | 0.00 |
| 55 | 5,000 | Block | H | 1.5 | 456.61 | 0.97 | 0.97 | 0.00 | 0.01 | 0.00 | 0.09 | 0.03 | 0.00 | 0.00 |
| 55 | 5,000 | Block | H | 1.4 | 603.02 | 0.97 | 0.97 | 0.00 | 0.01 | 0.00 | 0.09 | 0.03 | 0.00 | 0.00 |
| 55 | 5,000 | Block | LQHP | 0.6 | 660.52 | 0.98 | 0.97 | 0.00 | 0.01 | 0.00 | 0.22 | 0.05 | 0.05 | 0.01 |
| 55 | 5,000 | Block | LQH | 0.7 | 667.88 | 0.98 | 0.97 | 0.00 | 0.01 | 0.00 | 0.18 | 0.05 | 0.13 | 0.06 |
| 55 | 5,000 | Block | H | 1.6 | 854.61 | 0.97 | 0.97 | 0.00 | 0.01 | 0.00 | 0.13 | 0.06 | 0.02 | 0.00 |
| 55 | 5,000 | Block | H | 1.7 | 1351.45 | 0.97 | 0.96 | 0.00 | 0.01 | 0.00 | 0.13 | 0.06 | 0.02 | 0.00 |
| 55 | 5,000 | Block | LQH | 0.6 | 5751.47 | 0.98 | 0.97 | 0.00 | 0.01 | 0.00 | 0.22 | 0.05 | 0.07 | 0.01 |
| 55 | 5,000 | Block | H | 0.1 | NA | 0.99 | 0.95 | 0.00 | 0.01 | 0.00 | 0.23 | 0.05 | 0.02 | 0.00 |
| 55 | 5,000 | Block | LQH | 0.1 | NA | 0.99 | 0.95 | 0.00 | 0.01 | 0.00 | 0.20 | 0.04 | 0.02 | 0.00 |
| 55 | 5,000 | Block | LQHP | 0.1 | NA | 0.99 | 0.96 | 0.00 | 0.00 | 0.00 | 0.22 | 0.03 | 0.02 | 0.00 |
| 55 | 5,000 | Block | LQHPT | 0.1 | NA | 0.99 | 0.91 | 0.02 | 0.07 | 0.02 | 0.53 | 0.01 | 0.18 | 0.10 |
| 55 | 5,000 | Block | H | 0.2 | NA | 0.99 | 0.97 | 0.00 | 0.00 | 0.00 | 0.20 | 0.02 | 0.05 | 0.01 |
| 55 | 5,000 | Block | LQH | 0.2 | NA | 0.99 | 0.97 | 0.00 | 0.00 | 0.00 | 0.20 | 0.02 | 0.05 | 0.01 |
| 55 | 5,000 | Block | LQHP | 0.2 | NA | 0.99 | 0.97 | 0.00 | 0.00 | 0.00 | 0.20 | 0.02 | 0.02 | 0.00 |
| 55 | 5,000 | Block | LQHPT | 0.2 | NA | 0.99 | 0.92 | 0.02 | 0.07 | 0.02 | 0.58 | 0.01 | 0.18 | 0.10 |
| 55 | 5,000 | Block | H | 0.3 | NA | 0.99 | 0.97 | 0.00 | 0.00 | 0.00 | 0.16 | 0.02 | 0.04 | 0.01 |
| 55 | 5,000 | Block | LQH | 0.3 | NA | 0.99 | 0.97 | 0.00 | 0.00 | 0.00 | 0.20 | 0.04 | 0.02 | 0.00 |
| 55 | 5,000 | Block | LQHP | 0.3 | NA | 0.99 | 0.97 | 0.00 | 0.00 | 0.00 | 0.20 | 0.04 | 0.02 | 0.00 |
| 55 | 5,000 | Block | LQHPT | 0.3 | NA | 0.99 | 0.92 | 0.02 | 0.06 | 0.02 | 0.55 | 0.01 | 0.16 | 0.08 |
| 55 | 5,000 | Block | H | 0.4 | NA | 0.98 | 0.97 | 0.00 | 0.01 | 0.00 | 0.16 | 0.02 | 0.04 | 0.01 |
| 55 | 5,000 | Block | LQH | 0.4 | NA | 0.98 | 0.97 | 0.00 | 0.01 | 0.00 | 0.18 | 0.04 | 0.04 | 0.00 |
| 55 | 5,000 | Block | LQHP | 0.4 | NA | 0.98 | 0.97 | 0.00 | 0.01 | 0.00 | 0.18 | 0.04 | 0.04 | 0.01 |
| 55 | 5,000 | Block | LQHPT | 0.4 | NA | 0.99 | 0.93 | 0.02 | 0.06 | 0.02 | 0.47 | 0.05 | 0.14 | 0.08 |
| 55 | 5,000 | Block | H | 0.5 | NA | 0.98 | 0.97 | 0.00 | 0.01 | 0.00 | 0.16 | 0.02 | 0.05 | 0.00 |
| 55 | 5,000 | Block | LQH | 0.5 | NA | 0.98 | 0.97 | 0.00 | 0.01 | 0.00 | 0.25 | 0.06 | 0.07 | 0.01 |
| 55 | 5,000 | Block | LQHP | 0.5 | NA | 0.98 | 0.97 | 0.00 | 0.01 | 0.00 | 0.22 | 0.04 | 0.04 | 0.01 |
| 55 | 5,000 | Block | LQHPT | 0.5 | NA | 0.99 | 0.93 | 0.02 | 0.06 | 0.03 | 0.47 | 0.06 | 0.13 | 0.06 |
| 55 | 5,000 | Block | H | 0.6 | NA | 0.98 | 0.97 | 0.00 | 0.01 | 0.00 | 0.13 | 0.02 | 0.02 | 0.00 |
| 55 | 5,000 | Block | LQHPT | 0.6 | NA | 0.99 | 0.94 | 0.01 | 0.04 | 0.01 | 0.34 | 0.12 | 0.13 | 0.06 |
| 55 | 5,000 | Block | H | 0.7 | NA | 0.98 | 0.97 | 0.00 | 0.00 | 0.00 | 0.11 | 0.01 | 0.02 | 0.00 |
| 55 | 5,000 | Block | H | 0.8 | NA | 0.98 | 0.97 | 0.00 | 0.00 | 0.00 | 0.11 | 0.01 | 0.02 | 0.00 |
| 55 | 5,000 | Block | H | 0.9 | NA | 0.98 | 0.97 | 0.00 | 0.00 | 0.00 | 0.13 | 0.01 | 0.02 | 0.00 |
| 55 | 5,000 | Block | H | 1 | NA | 0.98 | 0.97 | 0.00 | 0.00 | 0.00 | 0.13 | 0.03 | 0.00 | 0.00 |
| 55 | 5,000 | Block | H | 1.1 | NA | 0.97 | 0.97 | 0.00 | 0.00 | 0.00 | 0.11 | 0.03 | 0.00 | 0.00 |
| 55 | 5,000 | Block | H | 1.2 | NA | 0.97 | 0.97 | 0.00 | 0.00 | 0.00 | 0.09 | 0.03 | 0.00 | 0.00 |
| 55 | 5,000 | Checkerboar1 | LQ | 1 | 0.00 | 0.97 | 0.96 | 0.00 | 0.00 | 0.00 | 0.09 | 0.00 | 0.03 | 0.00 |
| 55 | 5,000 | Checkerboar1 | LQ | 0.8 | 0.38 | 0.97 | 0.96 | 0.00 | 0.00 | 0.00 | 0.09 | 0.00 | 0.05 | 0.01 |
| 55 | 5,000 | Checkerboar1 | LQ | 1.1 | 0.74 | 0.97 | 0.96 | 0.00 | 0.00 | 0.00 | 0.09 | 0.00 | 0.03 | 0.00 |
| 55 | 5,000 | Checkerboar1 | LQ | 1.2 | 0.96 | 0.97 | 0.96 | 0.00 | 0.00 | 0.00 | 0.09 | 0.00 | 0.03 | 0.00 |
| 55 | 5,000 | Checkerboar1 | LQ | 0.6 | 1.21 | 0.97 | 0.97 | 0.00 | 0.00 | 0.00 | 0.11 | 0.00 | 0.07 | 0.00 |
| 55 | 5,000 | Checkerboar1 | LQ | 0.7 | 1.32 | 0.97 | 0.97 | 0.00 | 0.00 | 0.00 | 0.11 | 0.00 | 0.07 | 0.00 |
| 55 | 5,000 | Checkerboar1 | LQ | 0.9 | 2.33 | 0.97 | 0.96 | 0.00 | 0.00 | 0.00 | 0.09 | 0.00 | 0.05 | 0.01 |
| 55 | 5,000 | Checkerboar1 | LQ | 0.5 | 2.49 | 0.97 | 0.97 | 0.00 | 0.00 | 0.00 | 0.11 | 0.00 | 0.09 | 0.00 |
| 55 | 5,000 | Checkerboar1 | LQ | 1.3 | 3.36 | 0.97 | 0.96 | 0.00 | 0.00 | 0.00 | 0.09 | 0.00 | 0.03 | 0.00 |
| 55 | 5,000 | Checkerboar1 | LQ | 0.4 | 4.74 | 0.97 | 0.97 | 0.00 | 0.00 | 0.00 | 0.11 | 0.00 | 0.09 | 0.00 |
| 55 | 5,000 | Checkerboar1 | LQ | 0.1 | 4.89 | 0.97 | 0.97 | 0.00 | 0.01 | 0.00 | 0.11 | 0.00 | 0.09 | 0.00 |
| 55 | 5,000 | Checkerboar1 | LQ | 0.2 | 4.89 | 0.97 | 0.97 | 0.00 | 0.01 | 0.00 | 0.11 | 0.00 | 0.09 | 0.00 |
| 55 | 5,000 | Checkerboar1 | LQ | 0.3 | 4.89 | 0.97 | 0.97 | 0.00 | 0.00 | 0.00 | 0.12 | 0.00 | 0.09 | 0.00 |
| 55 | 5,000 | Checkerboar1 | LQ | 1.4 | 5.54 | 0.97 | 0.96 | 0.00 | 0.00 | 0.00 | 0.09 | 0.00 | 0.03 | 0.00 |
| 55 | 5,000 | Checkerboar1 | LQHP | 1 | 6.39 | 0.98 | 0.97 | 0.00 | 0.00 | 0.00 | 0.11 | 0.00 | 0.05 | 0.00 |
| 55 | 5,000 | Checkerboar1 | LQ | 1.5 | 7.04 | 0.97 | 0.96 | 0.00 | 0.00 | 0.00 | 0.09 | 0.00 | 0.03 | 0.00 |
| 55 | 5,000 | Checkerboar1 | LQ | 1.6 | 8.84 | 0.97 | 0.96 | 0.00 | 0.00 | 0.00 | 0.09 | 0.00 | 0.03 | 0.00 |
| 55 | 5,000 | Checkerboar1 | LQHP | 1.1 | 10.22 | 0.98 | 0.97 | 0.00 | 0.00 | 0.00 | 0.09 | 0.00 | 0.05 | 0.00 |
| 55 | 5,000 | Checkerboar1 | LQ | 1.7 | 10.85 | 0.97 | 0.96 | 0.00 | 0.00 | 0.00 | 0.09 | 0.00 | 0.03 | 0.00 |
| 55 | 5,000 | Checkerboar1 | LQ | 1.8 | 12.79 | 0.97 | 0.96 | 0.00 | 0.00 | 0.00 | 0.09 | 0.00 | 0.03 | 0.00 |
| 55 | 5,000 | Checkerboar1 | LQ | 1.9 | 14.68 | 0.97 | 0.96 | 0.00 | 0.00 | 0.00 | 0.09 | 0.00 | 0.03 | 0.00 |
| 55 | 5,000 | Checkerboar1 | LQ | 2 | 16.51 | 0.96 | 0.96 | 0.00 | 0.00 | 0.00 | 0.09 | 0.00 | 0.03 | 0.00 |
| 55 | 5,000 | Checkerboar1 | LQHPT | 1.9 | 16.68 | 0.98 | 0.97 | 0.00 | 0.01 | 0.00 | 0.13 | 0.00 | 0.07 | 0.00 |
| 55 | 5,000 | Checkerboar1 | LQHPT | 1.7 | 17.42 | 0.98 | 0.97 | 0.00 | 0.01 | 0.00 | 0.15 | 0.00 | 0.07 | 0.00 |
| 55 | 5,000 | Checkerboar1 | LQHPT | 1.8 | 20.24 | 0.98 | 0.97 | 0.00 | 0.01 | 0.00 | 0.13 | 0.00 | 0.07 | 0.00 |
| 55 | 5,000 | Checkerboar1 | LQH | 1.4 | 22.11 | 0.97 | 0.97 | 0.00 | 0.00 | 0.00 | 0.09 | 0.00 | 0.05 | 0.00 |
| 55 | 5,000 | Checkerboar1 | LQHP | 1.6 | 23.19 | 0.97 | 0.97 | 0.00 | 0.00 | 0.00 | 0.09 | 0.00 | 0.05 | 0.00 |
| 55 | 5,000 | Checkerboar1 | LQH | 1.9 | 24.48 | 0.97 | 0.96 | 0.00 | 0.00 | 0.00 | 0.09 | 0.00 | 0.04 | 0.00 |
| 55 | 5,000 | Checkerboar1 | LQHPT | 1.6 | 25.87 | 0.98 | 0.97 | 0.00 | 0.01 | 0.00 | 0.15 | 0.00 | 0.07 | 0.00 |
| 55 | 5,000 | Checkerboar1 | LQHP | 1.8 | 25.99 | 0.97 | 0.97 | 0.00 | 0.00 | 0.00 | 0.09 | 0.00 | 0.05 | 0.00 |
| 55 | 5,000 | Checkerboar1 | LQH | 2 | 26.07 | 0.97 | 0.96 | 0.00 | 0.00 | 0.00 | 0.09 | 0.00 | 0.05 | 0.00 |
| 55 | 5,000 | Checkerboar1 | LQH | 1.5 | 26.23 | 0.97 | 0.97 | 0.00 | 0.00 | 0.00 | 0.09 | 0.00 | 0.05 | 0.00 |
| 55 | 5,000 | Checkerboar1 | LQH | 1.3 | 26.48 | 0.97 | 0.97 | 0.00 | 0.00 | 0.00 | 0.07 | 0.00 | 0.05 | 0.00 |
| 55 | 5,000 | Checkerboar1 | LQHP | 1.5 | 27.50 | 0.97 | 0.97 | 0.00 | 0.00 | 0.00 | 0.09 | 0.00 | 0.05 | 0.00 |
| 55 | 5,000 | Checkerboar1 | LQHP | 1.4 | 28.83 | 0.97 | 0.97 | 0.00 | 0.00 | 0.00 | 0.09 | 0.00 | 0.05 | 0.00 |
| 55 | 5,000 | Checkerboar1 | LQHPT | 2 | 29.43 | 0.98 | 0.97 | 0.00 | 0.01 | 0.00 | 0.11 | 0.00 | 0.07 | 0.00 |
| 55 | 5,000 | Checkerboar1 | LQHP | 1.7 | 30.27 | 0.97 | 0.97 | 0.00 | 0.00 | 0.00 | 0.09 | 0.00 | 0.05 | 0.00 |
| 55 | 5,000 | Checkerboar1 | LQH | 1.8 | 31.39 | 0.97 | 0.97 | 0.00 | 0.00 | 0.00 | 0.09 | 0.00 | 0.05 | 0.00 |
| 55 | 5,000 | Checkerboar1 | LQHP | 1.3 | 31.57 | 0.98 | 0.97 | 0.00 | 0.00 | 0.00 | 0.09 | 0.00 | 0.05 | 0.00 |
| 55 | 5,000 | Checkerboar1 | LQH | 1.6 | 32.46 | 0.97 | 0.97 | 0.00 | 0.00 | 0.00 | 0.09 | 0.00 | 0.04 | 0.00 |
| 55 | 5,000 | Checkerboar1 | LQHP | 1.2 | 34.50 | 0.98 | 0.97 | 0.00 | 0.00 | 0.00 | 0.09 | 0.00 | 0.05 | 0.00 |
| 55 | 5,000 | Checkerboar1 | LQHP | 2 | 35.59 | 0.97 | 0.97 | 0.00 | 0.00 | 0.00 | 0.09 | 0.00 | 0.05 | 0.00 |
| 55 | 5,000 | Checkerboar1 | LQHP | 0.9 | 37.72 | 0.98 | 0.97 | 0.00 | 0.00 | 0.00 | 0.11 | 0.00 | 0.05 | 0.00 |
| 55 | 5,000 | Checkerboar1 | LQHP | 1.9 | 39.65 | 0.97 | 0.97 | 0.00 | 0.00 | 0.00 | 0.09 | 0.00 | 0.05 | 0.00 |
| 55 | 5,000 | Checkerboar1 | LQH | 1.7 | 40.78 | 0.97 | 0.97 | 0.00 | 0.00 | 0.00 | 0.09 | 0.00 | 0.05 | 0.00 |
| 55 | 5,000 | Checkerboar1 | LQH | 1.2 | 43.45 | 0.97 | 0.97 | 0.00 | 0.00 | 0.00 | 0.09 | 0.00 | 0.05 | 0.00 |
| 55 | 5,000 | Checkerboar1 | LQHPT | 1.4 | 45.72 | 0.98 | 0.97 | 0.00 | 0.01 | 0.00 | 0.15 | 0.00 | 0.09 | 0.00 |
| 55 | 5,000 | Checkerboar1 | LQH | 1.1 | 47.04 | 0.97 | 0.97 | 0.00 | 0.00 | 0.00 | 0.07 | 0.00 | 0.05 | 0.00 |
| 55 | 5,000 | Checkerboar1 | LQHPT | 1.5 | 50.76 | 0.98 | 0.97 | 0.00 | 0.01 | 0.00 | 0.15 | 0.00 | 0.09 | 0.00 |
| 55 | 5,000 | Checkerboar1 | LQHPT | 1.3 | 59.44 | 0.98 | 0.97 | 0.00 | 0.01 | 0.00 | 0.15 | 0.00 | 0.09 | 0.00 |
| 55 | 5,000 | Checkerboar1 | LQHPT | 0.9 | 72.80 | 0.99 | 0.97 | 0.00 | 0.01 | 0.00 | 0.22 | 0.00 | 0.09 | 0.00 |
| 55 | 5,000 | Checkerboar1 | LQHPT | 1.2 | 74.38 | 0.98 | 0.97 | 0.00 | 0.01 | 0.00 | 0.15 | 0.00 | 0.09 | 0.00 |
| 55 | 5,000 | Checkerboar1 | LQHPT | 1.1 | 78.53 | 0.98 | 0.97 | 0.00 | 0.01 | 0.00 | 0.17 | 0.00 | 0.09 | 0.00 |
| 55 | 5,000 | Checkerboar1 | LQHPT | 1 | 96.19 | 0.98 | 0.97 | 0.00 | 0.01 | 0.00 | 0.20 | 0.00 | 0.11 | 0.00 |
| 55 | 5,000 | Checkerboar1 | LQHPT | 0.8 | 111.60 | 0.99 | 0.97 | 0.00 | 0.01 | 0.00 | 0.24 | 0.00 | 0.11 | 0.00 |
| 55 | 5,000 | Checkerboar1 | H | 1.9 | 117.52 | 0.97 | 0.96 | 0.00 | 0.00 | 0.00 | 0.07 | 0.00 | 0.03 | 0.00 |
| 55 | 5,000 | Checkerboar1 | LQHP | 0.8 | 124.85 | 0.98 | 0.97 | 0.00 | 0.00 | 0.00 | 0.11 | 0.00 | 0.04 | 0.00 |
| 55 | 5,000 | Checkerboar1 | LQH | 1 | 127.05 | 0.98 | 0.97 | 0.00 | 0.00 | 0.00 | 0.07 | 0.00 | 0.05 | 0.00 |
| 55 | 5,000 | Checkerboar1 | L | 0.1 | 140.86 | 0.90 | 0.89 | 0.00 | 0.01 | 0.00 | 0.11 | 0.00 | 0.02 | 0.00 |
| 55 | 5,000 | Checkerboar1 | L | 0.2 | 140.86 | 0.90 | 0.89 | 0.00 | 0.01 | 0.00 | 0.11 | 0.00 | 0.02 | 0.00 |
| 55 | 5,000 | Checkerboar1 | L | 0.3 | 140.86 | 0.90 | 0.89 | 0.00 | 0.01 | 0.00 | 0.11 | 0.00 | 0.02 | 0.00 |
| 55 | 5,000 | Checkerboar1 | L | 0.4 | 140.86 | 0.90 | 0.89 | 0.00 | 0.01 | 0.00 | 0.09 | 0.00 | 0.02 | 0.00 |
| 55 | 5,000 | Checkerboar1 | L | 0.5 | 140.86 | 0.90 | 0.89 | 0.00 | 0.01 | 0.00 | 0.09 | 0.00 | 0.00 | 0.00 |
| 55 | 5,000 | Checkerboar1 | L | 0.6 | 141.09 | 0.90 | 0.89 | 0.00 | 0.01 | 0.00 | 0.11 | 0.01 | 0.02 | 0.00 |
| 55 | 5,000 | Checkerboar1 | L | 0.8 | 141.26 | 0.90 | 0.89 | 0.00 | 0.01 | 0.00 | 0.06 | 0.00 | 0.04 | 0.00 |
| 55 | 5,000 | Checkerboar1 | L | 0.7 | 141.47 | 0.90 | 0.89 | 0.00 | 0.01 | 0.00 | 0.11 | 0.00 | 0.02 | 0.00 |
| 55 | 5,000 | Checkerboar1 | L | 0.9 | 141.65 | 0.90 | 0.89 | 0.00 | 0.01 | 0.00 | 0.06 | 0.00 | 0.04 | 0.00 |
| 55 | 5,000 | Checkerboar1 | L | 1 | 142.00 | 0.90 | 0.89 | 0.00 | 0.01 | 0.00 | 0.06 | 0.00 | 0.04 | 0.00 |
| 55 | 5,000 | Checkerboar1 | L | 1.1 | 142.56 | 0.90 | 0.89 | 0.00 | 0.01 | 0.00 | 0.06 | 0.00 | 0.04 | 0.00 |
| 55 | 5,000 | Checkerboar1 | L | 1.2 | 143.35 | 0.90 | 0.88 | 0.00 | 0.01 | 0.00 | 0.07 | 0.00 | 0.04 | 0.00 |
| 55 | 5,000 | Checkerboar1 | L | 1.3 | 144.31 | 0.90 | 0.88 | 0.00 | 0.01 | 0.00 | 0.07 | 0.00 | 0.04 | 0.00 |
| 55 | 5,000 | Checkerboar1 | L | 1.6 | 144.78 | 0.89 | 0.87 | 0.00 | 0.01 | 0.00 | 0.07 | 0.00 | 0.02 | 0.00 |
| 55 | 5,000 | Checkerboar1 | L | 1.4 | 145.61 | 0.90 | 0.87 | 0.00 | 0.01 | 0.00 | 0.07 | 0.00 | 0.04 | 0.00 |
| 55 | 5,000 | Checkerboar1 | L | 1.7 | 145.63 | 0.89 | 0.87 | 0.00 | 0.01 | 0.00 | 0.07 | 0.00 | 0.02 | 0.00 |
| 55 | 5,000 | Checkerboar1 | L | 1.8 | 146.50 | 0.89 | 0.87 | 0.00 | 0.01 | 0.00 | 0.07 | 0.00 | 0.02 | 0.00 |
| 55 | 5,000 | Checkerboar1 | L | 1.5 | 146.57 | 0.89 | 0.87 | 0.00 | 0.01 | 0.00 | 0.07 | 0.00 | 0.02 | 0.00 |
| 55 | 5,000 | Checkerboar1 | L | 1.9 | 147.41 | 0.89 | 0.87 | 0.00 | 0.01 | 0.00 | 0.07 | 0.00 | 0.02 | 0.00 |
| 55 | 5,000 | Checkerboar1 | L | 2 | 148.41 | 0.89 | 0.87 | 0.00 | 0.01 | 0.00 | 0.07 | 0.00 | 0.02 | 0.00 |
| 55 | 5,000 | Checkerboar1 | H | 2 | 242.88 | 0.97 | 0.96 | 0.00 | 0.00 | 0.00 | 0.07 | 0.00 | 0.03 | 0.00 |
| 55 | 5,000 | Checkerboar1 | H | 1.8 | 292.32 | 0.97 | 0.96 | 0.00 | 0.00 | 0.00 | 0.07 | 0.00 | 0.03 | 0.00 |
| 55 | 5,000 | Checkerboar1 | LQHP | 0.7 | 315.54 | 0.98 | 0.97 | 0.00 | 0.00 | 0.00 | 0.11 | 0.00 | 0.05 | 0.00 |
| 55 | 5,000 | Checkerboar1 | LQH | 0.9 | 376.75 | 0.98 | 0.97 | 0.00 | 0.00 | 0.00 | 0.05 | 0.00 | 0.05 | 0.00 |
| 55 | 5,000 | Checkerboar1 | LQH | 0.8 | 422.03 | 0.98 | 0.97 | 0.00 | 0.00 | 0.00 | 0.05 | 0.00 | 0.05 | 0.00 |
| 55 | 5,000 | Checkerboar1 | LQHPT | 0.7 | 430.35 | 0.99 | 0.97 | 0.00 | 0.02 | 0.00 | 0.26 | 0.01 | 0.11 | 0.00 |
| 55 | 5,000 | Checkerboar1 | H | 1.3 | 451.97 | 0.97 | 0.97 | 0.00 | 0.00 | 0.00 | 0.09 | 0.00 | 0.05 | 0.00 |
| 55 | 5,000 | Checkerboar1 | H | 1.5 | 456.61 | 0.97 | 0.97 | 0.00 | 0.00 | 0.00 | 0.09 | 0.00 | 0.07 | 0.00 |
| 55 | 5,000 | Checkerboar1 | H | 1.4 | 603.02 | 0.97 | 0.97 | 0.00 | 0.00 | 0.00 | 0.09 | 0.00 | 0.05 | 0.00 |
| 55 | 5,000 | Checkerboar1 | LQHP | 0.6 | 660.52 | 0.98 | 0.97 | 0.00 | 0.01 | 0.00 | 0.11 | 0.00 | 0.05 | 0.00 |
| 55 | 5,000 | Checkerboar1 | LQH | 0.7 | 667.88 | 0.98 | 0.97 | 0.00 | 0.00 | 0.00 | 0.09 | 0.00 | 0.05 | 0.00 |
| 55 | 5,000 | Checkerboar1 | H | 1.6 | 854.61 | 0.97 | 0.97 | 0.00 | 0.00 | 0.00 | 0.09 | 0.00 | 0.07 | 0.00 |
| 55 | 5,000 | Checkerboar1 | H | 1.7 | 1351.45 | 0.97 | 0.96 | 0.00 | 0.00 | 0.00 | 0.09 | 0.00 | 0.03 | 0.00 |
| 55 | 5,000 | Checkerboar1 | LQH | 0.6 | 5751.47 | 0.98 | 0.97 | 0.00 | 0.00 | 0.00 | 0.11 | 0.00 | 0.04 | 0.00 |
| 55 | 5,000 | Checkerboar1 | H | 0.1 | NA | 0.99 | 0.98 | 0.00 | 0.01 | 0.00 | 0.31 | 0.00 | 0.18 | 0.02 |
| 55 | 5,000 | Checkerboar1 | LQH | 0.1 | NA | 0.99 | 0.98 | 0.00 | 0.01 | 0.00 | 0.31 | 0.00 | 0.18 | 0.02 |
| 55 | 5,000 | Checkerboar1 | LQHP | 0.1 | NA | 0.99 | 0.98 | 0.00 | 0.01 | 0.00 | 0.31 | 0.00 | 0.16 | 0.01 |
| 55 | 5,000 | Checkerboar1 | LQHPT | 0.1 | NA | 0.99 | 0.93 | 0.00 | 0.06 | 0.00 | 0.44 | 0.00 | 0.40 | 0.00 |
| 55 | 5,000 | Checkerboar1 | H | 0.2 | NA | 0.99 | 0.98 | 0.00 | 0.01 | 0.00 | 0.23 | 0.00 | 0.05 | 0.00 |
| 55 | 5,000 | Checkerboar1 | LQH | 0.2 | NA | 0.99 | 0.98 | 0.00 | 0.01 | 0.00 | 0.23 | 0.00 | 0.05 | 0.00 |
| 55 | 5,000 | Checkerboar1 | LQHP | 0.2 | NA | 0.99 | 0.98 | 0.00 | 0.01 | 0.00 | 0.23 | 0.00 | 0.05 | 0.00 |
| 55 | 5,000 | Checkerboar1 | LQHPT | 0.2 | NA | 0.99 | 0.94 | 0.00 | 0.05 | 0.00 | 0.42 | 0.00 | 0.34 | 0.00 |
| 55 | 5,000 | Checkerboar1 | H | 0.3 | NA | 0.99 | 0.98 | 0.00 | 0.01 | 0.00 | 0.20 | 0.00 | 0.04 | 0.00 |
| 55 | 5,000 | Checkerboar1 | LQH | 0.3 | NA | 0.99 | 0.98 | 0.00 | 0.01 | 0.00 | 0.20 | 0.00 | 0.05 | 0.00 |
| 55 | 5,000 | Checkerboar1 | LQHP | 0.3 | NA | 0.99 | 0.98 | 0.00 | 0.01 | 0.00 | 0.18 | 0.01 | 0.05 | 0.00 |
| 55 | 5,000 | Checkerboar1 | LQHPT | 0.3 | NA | 0.99 | 0.94 | 0.00 | 0.06 | 0.00 | 0.40 | 0.00 | 0.35 | 0.00 |
| 55 | 5,000 | Checkerboar1 | H | 0.4 | NA | 0.98 | 0.97 | 0.00 | 0.01 | 0.00 | 0.15 | 0.00 | 0.04 | 0.00 |
| 55 | 5,000 | Checkerboar1 | LQH | 0.4 | NA | 0.98 | 0.97 | 0.00 | 0.01 | 0.00 | 0.13 | 0.00 | 0.05 | 0.00 |
| 55 | 5,000 | Checkerboar1 | LQHP | 0.4 | NA | 0.98 | 0.97 | 0.00 | 0.01 | 0.00 | 0.14 | 0.00 | 0.05 | 0.00 |
| 55 | 5,000 | Checkerboar1 | LQHPT | 0.4 | NA | 0.99 | 0.94 | 0.00 | 0.05 | 0.00 | 0.38 | 0.00 | 0.25 | 0.01 |
| 55 | 5,000 | Checkerboar1 | H | 0.5 | NA | 0.98 | 0.97 | 0.00 | 0.01 | 0.00 | 0.15 | 0.00 | 0.06 | 0.00 |
| 55 | 5,000 | Checkerboar1 | LQH | 0.5 | NA | 0.98 | 0.97 | 0.00 | 0.01 | 0.00 | 0.13 | 0.00 | 0.05 | 0.00 |
| 55 | 5,000 | Checkerboar1 | LQHP | 0.5 | NA | 0.98 | 0.97 | 0.00 | 0.01 | 0.00 | 0.11 | 0.00 | 0.05 | 0.00 |
| 55 | 5,000 | Checkerboar1 | LQHPT | 0.5 | NA | 0.99 | 0.96 | 0.00 | 0.03 | 0.00 | 0.36 | 0.00 | 0.16 | 0.00 |
| 55 | 5,000 | Checkerboar1 | H | 0.6 | NA | 0.98 | 0.97 | 0.00 | 0.01 | 0.00 | 0.13 | 0.00 | 0.07 | 0.00 |
| 55 | 5,000 | Checkerboar1 | LQHPT | 0.6 | NA | 0.99 | 0.97 | 0.00 | 0.02 | 0.00 | 0.26 | 0.01 | 0.15 | 0.00 |
| 55 | 5,000 | Checkerboar1 | H | 0.7 | NA | 0.98 | 0.97 | 0.00 | 0.00 | 0.00 | 0.11 | 0.00 | 0.05 | 0.00 |
| 55 | 5,000 | Checkerboar1 | H | 0.8 | NA | 0.98 | 0.97 | 0.00 | 0.00 | 0.00 | 0.13 | 0.00 | 0.05 | 0.00 |
| 55 | 5,000 | Checkerboar1 | H | 0.9 | NA | 0.98 | 0.97 | 0.00 | 0.00 | 0.00 | 0.11 | 0.00 | 0.05 | 0.00 |
| 55 | 5,000 | Checkerboar1 | H | 1 | NA | 0.98 | 0.97 | 0.00 | 0.00 | 0.00 | 0.09 | 0.00 | 0.05 | 0.00 |
| 55 | 5,000 | Checkerboar1 | H | 1.1 | NA | 0.97 | 0.97 | 0.00 | 0.00 | 0.00 | 0.09 | 0.00 | 0.05 | 0.00 |
| 55 | 5,000 | Checkerboar1 | H | 1.2 | NA | 0.97 | 0.97 | 0.00 | 0.00 | 0.00 | 0.09 | 0.00 | 0.05 | 0.00 |
| 55 | 10,000 | Jackknife | LQHPT | 1.4 | 0.00 | 0.98 | 0.97 | 0.08 | 0.02 | 0.06 | 0.15 | 0.13 | 0.07 | 0.07 |
| 55 | 10,000 | Jackknife | LQ | 1 | 4.63 | 0.97 | 0.97 | 0.08 | 0.02 | 0.06 | 0.11 | 0.10 | 0.02 | 0.02 |
| 55 | 10,000 | Jackknife | LQ | 0.6 | 5.17 | 0.97 | 0.97 | 0.09 | 0.02 | 0.06 | 0.11 | 0.10 | 0.05 | 0.05 |
| 55 | 10,000 | Jackknife | LQ | 0.7 | 5.98 | 0.97 | 0.97 | 0.08 | 0.02 | 0.06 | 0.11 | 0.10 | 0.02 | 0.02 |
| 55 | 10,000 | Jackknife | LQ | 1.1 | 6.20 | 0.97 | 0.97 | 0.08 | 0.02 | 0.06 | 0.11 | 0.10 | 0.04 | 0.04 |
| 55 | 10,000 | Jackknife | LQ | 0.9 | 7.18 | 0.97 | 0.97 | 0.08 | 0.02 | 0.06 | 0.11 | 0.10 | 0.02 | 0.02 |
| 55 | 10,000 | Jackknife | LQ | 1.2 | 7.76 | 0.97 | 0.96 | 0.08 | 0.02 | 0.06 | 0.11 | 0.10 | 0.04 | 0.04 |
| 55 | 10,000 | Jackknife | LQ | 0.5 | 7.96 | 0.97 | 0.97 | 0.09 | 0.02 | 0.06 | 0.11 | 0.10 | 0.05 | 0.05 |
| 55 | 10,000 | Jackknife | LQ | 0.1 | 9.38 | 0.97 | 0.97 | 0.09 | 0.02 | 0.07 | 0.11 | 0.10 | 0.04 | 0.04 |
| 55 | 10,000 | Jackknife | LQ | 0.2 | 9.38 | 0.97 | 0.97 | 0.09 | 0.02 | 0.07 | 0.11 | 0.10 | 0.04 | 0.04 |
| 55 | 10,000 | Jackknife | LQ | 0.3 | 9.38 | 0.97 | 0.97 | 0.09 | 0.02 | 0.07 | 0.11 | 0.10 | 0.04 | 0.04 |
| 55 | 10,000 | Jackknife | LQ | 1.3 | 9.74 | 0.97 | 0.96 | 0.08 | 0.02 | 0.06 | 0.11 | 0.10 | 0.04 | 0.04 |
| 55 | 10,000 | Jackknife | LQ | 0.4 | 10.48 | 0.97 | 0.97 | 0.09 | 0.02 | 0.06 | 0.11 | 0.10 | 0.04 | 0.04 |
| 55 | 10,000 | Jackknife | LQ | 1.4 | 11.65 | 0.97 | 0.96 | 0.09 | 0.02 | 0.06 | 0.11 | 0.10 | 0.04 | 0.04 |
| 55 | 10,000 | Jackknife | LQ | 0.8 | 12.71 | 0.97 | 0.97 | 0.08 | 0.02 | 0.06 | 0.11 | 0.10 | 0.04 | 0.04 |
| 55 | 10,000 | Jackknife | LQ | 1.5 | 13.40 | 0.97 | 0.96 | 0.09 | 0.02 | 0.06 | 0.11 | 0.10 | 0.02 | 0.02 |
| 55 | 10,000 | Jackknife | LQ | 1.6 | 15.67 | 0.97 | 0.96 | 0.09 | 0.02 | 0.06 | 0.11 | 0.10 | 0.02 | 0.02 |
| 55 | 10,000 | Jackknife | LQ | 1.7 | 17.85 | 0.97 | 0.96 | 0.09 | 0.02 | 0.06 | 0.11 | 0.10 | 0.02 | 0.02 |
| 55 | 10,000 | Jackknife | LQHPT | 1.5 | 18.22 | 0.98 | 0.97 | 0.08 | 0.02 | 0.06 | 0.15 | 0.13 | 0.07 | 0.07 |
| 55 | 10,000 | Jackknife | LQ | 1.8 | 19.38 | 0.96 | 0.96 | 0.09 | 0.02 | 0.06 | 0.11 | 0.10 | 0.02 | 0.02 |
| 55 | 10,000 | Jackknife | LQHPT | 1.1 | 21.13 | 0.98 | 0.97 | 0.08 | 0.02 | 0.06 | 0.20 | 0.16 | 0.09 | 0.08 |
| 55 | 10,000 | Jackknife | LQ | 1.9 | 21.68 | 0.96 | 0.96 | 0.10 | 0.02 | 0.07 | 0.13 | 0.11 | 0.02 | 0.02 |
| 55 | 10,000 | Jackknife | LQ | 2 | 23.67 | 0.96 | 0.96 | 0.10 | 0.02 | 0.07 | 0.13 | 0.11 | 0.02 | 0.02 |
| 55 | 10,000 | Jackknife | LQHPT | 1.3 | 24.33 | 0.98 | 0.97 | 0.08 | 0.02 | 0.06 | 0.15 | 0.13 | 0.07 | 0.07 |
| 55 | 10,000 | Jackknife | LQHP | 1.3 | 24.33 | 0.97 | 0.97 | 0.07 | 0.02 | 0.04 | 0.15 | 0.13 | 0.05 | 0.05 |
| 55 | 10,000 | Jackknife | LQHP | 1.2 | 32.23 | 0.97 | 0.97 | 0.06 | 0.02 | 0.04 | 0.15 | 0.13 | 0.05 | 0.05 |
| 55 | 10,000 | Jackknife | LQH | 1.6 | 33.46 | 0.97 | 0.97 | 0.09 | 0.02 | 0.06 | 0.15 | 0.13 | 0.09 | 0.08 |
| 55 | 10,000 | Jackknife | LQHP | 1.1 | 33.91 | 0.97 | 0.97 | 0.06 | 0.02 | 0.04 | 0.15 | 0.13 | 0.07 | 0.07 |
| 55 | 10,000 | Jackknife | LQHP | 1 | 37.32 | 0.98 | 0.97 | 0.06 | 0.02 | 0.04 | 0.15 | 0.13 | 0.07 | 0.07 |
| 55 | 10,000 | Jackknife | LQHP | 1.5 | 37.93 | 0.97 | 0.97 | 0.07 | 0.02 | 0.05 | 0.15 | 0.13 | 0.05 | 0.05 |
| 55 | 10,000 | Jackknife | LQHPT | 1.8 | 38.03 | 0.98 | 0.97 | 0.09 | 0.02 | 0.06 | 0.15 | 0.13 | 0.07 | 0.07 |
| 55 | 10,000 | Jackknife | LQHP | 2 | 38.33 | 0.97 | 0.97 | 0.08 | 0.02 | 0.05 | 0.15 | 0.13 | 0.05 | 0.05 |
| 55 | 10,000 | Jackknife | LQHPT | 1.6 | 39.98 | 0.98 | 0.97 | 0.08 | 0.02 | 0.06 | 0.15 | 0.13 | 0.07 | 0.07 |
| 55 | 10,000 | Jackknife | LQHPT | 1.2 | 40.75 | 0.98 | 0.97 | 0.08 | 0.02 | 0.06 | 0.18 | 0.15 | 0.09 | 0.08 |
| 55 | 10,000 | Jackknife | LQH | 1.4 | 40.84 | 0.97 | 0.97 | 0.08 | 0.02 | 0.06 | 0.15 | 0.13 | 0.09 | 0.08 |
| 55 | 10,000 | Jackknife | LQHP | 1.9 | 40.89 | 0.97 | 0.97 | 0.08 | 0.02 | 0.05 | 0.15 | 0.13 | 0.05 | 0.05 |
| 55 | 10,000 | Jackknife | LQHPT | 1.7 | 42.44 | 0.98 | 0.97 | 0.09 | 0.02 | 0.06 | 0.15 | 0.13 | 0.07 | 0.07 |
| 55 | 10,000 | Jackknife | LQH | 1.7 | 45.25 | 0.97 | 0.96 | 0.09 | 0.02 | 0.06 | 0.15 | 0.13 | 0.07 | 0.07 |
| 55 | 10,000 | Jackknife | LQHP | 1.6 | 46.37 | 0.97 | 0.97 | 0.07 | 0.02 | 0.05 | 0.15 | 0.13 | 0.05 | 0.05 |
| 55 | 10,000 | Jackknife | LQH | 1.5 | 46.63 | 0.97 | 0.97 | 0.09 | 0.02 | 0.06 | 0.15 | 0.13 | 0.09 | 0.08 |
| 55 | 10,000 | Jackknife | LQHPT | 1.9 | 46.68 | 0.97 | 0.97 | 0.09 | 0.02 | 0.06 | 0.15 | 0.13 | 0.07 | 0.07 |
| 55 | 10,000 | Jackknife | LQH | 1.8 | 46.92 | 0.97 | 0.96 | 0.09 | 0.02 | 0.06 | 0.15 | 0.13 | 0.05 | 0.05 |
| 55 | 10,000 | Jackknife | LQHP | 1.4 | 48.18 | 0.97 | 0.97 | 0.07 | 0.02 | 0.04 | 0.15 | 0.13 | 0.05 | 0.05 |
| 55 | 10,000 | Jackknife | LQHPT | 1 | 52.41 | 0.98 | 0.97 | 0.07 | 0.02 | 0.06 | 0.24 | 0.18 | 0.07 | 0.07 |
| 55 | 10,000 | Jackknife | LQHPT | 2 | 55.86 | 0.97 | 0.97 | 0.09 | 0.02 | 0.06 | 0.15 | 0.13 | 0.07 | 0.07 |
| 55 | 10,000 | Jackknife | LQHP | 1.8 | 56.03 | 0.97 | 0.97 | 0.07 | 0.02 | 0.05 | 0.15 | 0.13 | 0.05 | 0.05 |
| 55 | 10,000 | Jackknife | LQH | 2 | 57.46 | 0.97 | 0.96 | 0.09 | 0.02 | 0.06 | 0.15 | 0.13 | 0.04 | 0.04 |
| 55 | 10,000 | Jackknife | LQHP | 1.7 | 67.72 | 0.97 | 0.97 | 0.07 | 0.02 | 0.05 | 0.15 | 0.13 | 0.05 | 0.05 |
| 55 | 10,000 | Jackknife | LQH | 1.9 | 67.89 | 0.97 | 0.96 | 0.09 | 0.02 | 0.06 | 0.15 | 0.13 | 0.04 | 0.04 |
| 55 | 10,000 | Jackknife | LQHPT | 0.9 | 78.07 | 0.98 | 0.97 | 0.07 | 0.02 | 0.06 | 0.27 | 0.20 | 0.07 | 0.07 |
| 55 | 10,000 | Jackknife | LQH | 1.3 | 86.28 | 0.97 | 0.97 | 0.08 | 0.02 | 0.05 | 0.15 | 0.13 | 0.09 | 0.08 |
| 55 | 10,000 | Jackknife | LQH | 1.2 | 93.10 | 0.97 | 0.97 | 0.08 | 0.02 | 0.05 | 0.15 | 0.13 | 0.07 | 0.07 |
| 55 | 10,000 | Jackknife | LQHP | 0.8 | 104.22 | 0.98 | 0.97 | 0.06 | 0.02 | 0.04 | 0.16 | 0.14 | 0.05 | 0.05 |
| 55 | 10,000 | Jackknife | LQHP | 0.7 | 110.50 | 0.98 | 0.97 | 0.06 | 0.02 | 0.04 | 0.20 | 0.16 | 0.04 | 0.04 |
| 55 | 10,000 | Jackknife | LQHP | 0.9 | 123.27 | 0.98 | 0.97 | 0.06 | 0.02 | 0.04 | 0.15 | 0.13 | 0.07 | 0.07 |
| 55 | 10,000 | Jackknife | L | 0.6 | 144.74 | 0.90 | 0.90 | 0.34 | 0.03 | 0.19 | 0.11 | 0.10 | 0.02 | 0.02 |
| 55 | 10,000 | Jackknife | L | 0.1 | 144.79 | 0.90 | 0.90 | 0.35 | 0.03 | 0.20 | 0.11 | 0.10 | 0.02 | 0.02 |
| 55 | 10,000 | Jackknife | L | 0.2 | 144.79 | 0.90 | 0.90 | 0.35 | 0.03 | 0.20 | 0.11 | 0.10 | 0.02 | 0.02 |
| 55 | 10,000 | Jackknife | L | 0.3 | 144.79 | 0.90 | 0.90 | 0.35 | 0.03 | 0.20 | 0.11 | 0.10 | 0.02 | 0.02 |
| 55 | 10,000 | Jackknife | L | 0.4 | 144.79 | 0.90 | 0.90 | 0.35 | 0.03 | 0.20 | 0.11 | 0.10 | 0.02 | 0.02 |
| 55 | 10,000 | Jackknife | L | 0.5 | 144.79 | 0.90 | 0.90 | 0.35 | 0.03 | 0.20 | 0.11 | 0.10 | 0.02 | 0.02 |
| 55 | 10,000 | Jackknife | L | 0.7 | 144.89 | 0.90 | 0.90 | 0.34 | 0.03 | 0.19 | 0.11 | 0.10 | 0.02 | 0.02 |
| 55 | 10,000 | Jackknife | L | 0.8 | 145.27 | 0.90 | 0.90 | 0.34 | 0.03 | 0.19 | 0.11 | 0.10 | 0.02 | 0.02 |
| 55 | 10,000 | Jackknife | L | 0.9 | 145.53 | 0.90 | 0.90 | 0.34 | 0.03 | 0.19 | 0.11 | 0.10 | 0.02 | 0.02 |
| 55 | 10,000 | Jackknife | L | 1 | 145.82 | 0.90 | 0.90 | 0.33 | 0.03 | 0.19 | 0.11 | 0.10 | 0.02 | 0.02 |
| 55 | 10,000 | Jackknife | L | 1.1 | 146.32 | 0.90 | 0.90 | 0.33 | 0.03 | 0.18 | 0.11 | 0.10 | 0.02 | 0.02 |
| 55 | 10,000 | Jackknife | L | 1.2 | 147.16 | 0.90 | 0.90 | 0.32 | 0.03 | 0.18 | 0.11 | 0.10 | 0.02 | 0.02 |
| 55 | 10,000 | Jackknife | L | 1.7 | 147.47 | 0.90 | 0.90 | 0.27 | 0.03 | 0.14 | 0.13 | 0.11 | 0.02 | 0.02 |
| 55 | 10,000 | Jackknife | L | 1.5 | 147.81 | 0.90 | 0.90 | 0.29 | 0.03 | 0.15 | 0.11 | 0.10 | 0.04 | 0.04 |
| 55 | 10,000 | Jackknife | L | 1.3 | 148.24 | 0.90 | 0.90 | 0.31 | 0.03 | 0.17 | 0.11 | 0.10 | 0.02 | 0.02 |
| 55 | 10,000 | Jackknife | L | 1.8 | 148.38 | 0.90 | 0.90 | 0.27 | 0.03 | 0.13 | 0.13 | 0.11 | 0.02 | 0.02 |
| 55 | 10,000 | Jackknife | L | 1.6 | 148.92 | 0.90 | 0.90 | 0.28 | 0.03 | 0.14 | 0.13 | 0.11 | 0.04 | 0.04 |
| 55 | 10,000 | Jackknife | L | 1.4 | 149.34 | 0.90 | 0.90 | 0.30 | 0.03 | 0.16 | 0.11 | 0.10 | 0.02 | 0.02 |
| 55 | 10,000 | Jackknife | L | 1.9 | 149.35 | 0.90 | 0.90 | 0.26 | 0.03 | 0.13 | 0.13 | 0.11 | 0.02 | 0.02 |
| 55 | 10,000 | Jackknife | L | 2 | 150.07 | 0.90 | 0.90 | 0.26 | 0.03 | 0.13 | 0.11 | 0.10 | 0.02 | 0.02 |
| 55 | 10,000 | Jackknife | LQH | 1.1 | 163.90 | 0.97 | 0.97 | 0.08 | 0.02 | 0.05 | 0.15 | 0.13 | 0.07 | 0.07 |
| 55 | 10,000 | Jackknife | LQH | 1 | 217.07 | 0.98 | 0.97 | 0.07 | 0.02 | 0.05 | 0.15 | 0.13 | 0.07 | 0.07 |
| 55 | 10,000 | Jackknife | LQH | 0.7 | 222.92 | 0.98 | 0.97 | 0.06 | 0.02 | 0.04 | 0.24 | 0.18 | 0.04 | 0.04 |
| 55 | 10,000 | Jackknife | LQHPT | 0.7 | 246.55 | 0.99 | 0.97 | 0.07 | 0.02 | 0.06 | 0.29 | 0.21 | 0.05 | 0.05 |
| 55 | 10,000 | Jackknife | LQHPT | 0.8 | 293.67 | 0.98 | 0.97 | 0.07 | 0.02 | 0.06 | 0.27 | 0.20 | 0.05 | 0.05 |
| 55 | 10,000 | Jackknife | LQH | 0.8 | 328.56 | 0.98 | 0.97 | 0.06 | 0.02 | 0.05 | 0.20 | 0.16 | 0.04 | 0.04 |
| 55 | 10,000 | Jackknife | H | 1.4 | 362.06 | 0.97 | 0.96 | 0.09 | 0.02 | 0.06 | 0.15 | 0.13 | 0.02 | 0.02 |
| 55 | 10,000 | Jackknife | H | 2 | 472.34 | 0.97 | 0.96 | 0.10 | 0.02 | 0.07 | 0.15 | 0.13 | 0.02 | 0.02 |
| 55 | 10,000 | Jackknife | LQH | 0.9 | 582.77 | 0.98 | 0.97 | 0.07 | 0.02 | 0.05 | 0.15 | 0.13 | 0.05 | 0.05 |
| 55 | 10,000 | Jackknife | H | 1.3 | 606.40 | 0.97 | 0.97 | 0.09 | 0.02 | 0.06 | 0.15 | 0.13 | 0.04 | 0.04 |
| 55 | 10,000 | Jackknife | LQHP | 0.6 | 668.11 | 0.98 | 0.97 | 0.05 | 0.02 | 0.04 | 0.24 | 0.18 | 0.05 | 0.05 |
| 55 | 10,000 | Jackknife | H | 1.7 | 721.23 | 0.97 | 0.96 | 0.09 | 0.02 | 0.06 | 0.15 | 0.13 | 0.02 | 0.02 |
| 55 | 10,000 | Jackknife | LQHPT | 0.6 | 768.44 | 0.99 | 0.97 | 0.08 | 0.02 | 0.07 | 0.27 | 0.20 | 0.04 | 0.04 |
| 55 | 10,000 | Jackknife | LQH | 0.6 | 1304.64 | 0.98 | 0.97 | 0.06 | 0.02 | 0.04 | 0.22 | 0.17 | 0.04 | 0.04 |
| 55 | 10,000 | Jackknife | H | 1.5 | 1354.10 | 0.97 | 0.96 | 0.09 | 0.02 | 0.06 | 0.15 | 0.13 | 0.02 | 0.02 |
| 55 | 10,000 | Jackknife | H | 1.2 | 1838.75 | 0.97 | 0.97 | 0.08 | 0.02 | 0.06 | 0.15 | 0.13 | 0.05 | 0.05 |
| 55 | 10,000 | Jackknife | LQHPT | 0.5 | 2736.81 | 0.99 | 0.97 | 0.10 | 0.02 | 0.09 | 0.27 | 0.20 | 0.05 | 0.05 |
| 55 | 10,000 | Jackknife | H | 1.8 | 2844.43 | 0.97 | 0.96 | 0.10 | 0.02 | 0.07 | 0.15 | 0.13 | 0.02 | 0.02 |
| 55 | 10,000 | Jackknife | H | 1.9 | 5816.50 | 0.97 | 0.96 | 0.10 | 0.02 | 0.07 | 0.15 | 0.13 | 0.02 | 0.02 |
| 55 | 10,000 | Jackknife | H | 0.1 | NA | 0.99 | 0.98 | 0.04 | 0.01 | 0.04 | 0.27 | 0.20 | 0.04 | 0.04 |
| 55 | 10,000 | Jackknife | LQH | 0.1 | NA | 0.99 | 0.98 | 0.04 | 0.01 | 0.04 | 0.25 | 0.19 | 0.04 | 0.04 |
| 55 | 10,000 | Jackknife | LQHP | 0.1 | NA | 0.99 | 0.98 | 0.04 | 0.01 | 0.04 | 0.25 | 0.19 | 0.04 | 0.04 |
| 55 | 10,000 | Jackknife | LQHPT | 0.1 | NA | 0.99 | 0.96 | 0.24 | 0.03 | 0.24 | 0.58 | 0.25 | 0.13 | 0.11 |
| 55 | 10,000 | Jackknife | H | 0.2 | NA | 0.99 | 0.98 | 0.04 | 0.01 | 0.04 | 0.20 | 0.16 | 0.04 | 0.04 |
| 55 | 10,000 | Jackknife | LQH | 0.2 | NA | 0.99 | 0.98 | 0.04 | 0.01 | 0.04 | 0.20 | 0.16 | 0.04 | 0.04 |
| 55 | 10,000 | Jackknife | LQHP | 0.2 | NA | 0.99 | 0.98 | 0.04 | 0.01 | 0.04 | 0.18 | 0.15 | 0.04 | 0.04 |
| 55 | 10,000 | Jackknife | LQHPT | 0.2 | NA | 0.99 | 0.96 | 0.18 | 0.03 | 0.18 | 0.58 | 0.25 | 0.09 | 0.08 |
| 55 | 10,000 | Jackknife | H | 0.3 | NA | 0.99 | 0.98 | 0.05 | 0.01 | 0.04 | 0.18 | 0.15 | 0.04 | 0.04 |
| 55 | 10,000 | Jackknife | LQH | 0.3 | NA | 0.99 | 0.98 | 0.05 | 0.01 | 0.04 | 0.16 | 0.14 | 0.04 | 0.04 |
| 55 | 10,000 | Jackknife | LQHP | 0.3 | NA | 0.99 | 0.98 | 0.05 | 0.01 | 0.04 | 0.16 | 0.14 | 0.04 | 0.04 |
| 55 | 10,000 | Jackknife | LQHPT | 0.3 | NA | 0.99 | 0.96 | 0.18 | 0.03 | 0.18 | 0.38 | 0.24 | 0.09 | 0.08 |
| 55 | 10,000 | Jackknife | H | 0.4 | NA | 0.98 | 0.98 | 0.05 | 0.01 | 0.04 | 0.22 | 0.17 | 0.04 | 0.04 |
| 55 | 10,000 | Jackknife | LQH | 0.4 | NA | 0.98 | 0.98 | 0.05 | 0.01 | 0.04 | 0.22 | 0.17 | 0.04 | 0.04 |
| 55 | 10,000 | Jackknife | LQHP | 0.4 | NA | 0.98 | 0.98 | 0.05 | 0.01 | 0.04 | 0.24 | 0.18 | 0.04 | 0.04 |
| 55 | 10,000 | Jackknife | LQHPT | 0.4 | NA | 0.99 | 0.97 | 0.12 | 0.02 | 0.11 | 0.27 | 0.20 | 0.07 | 0.07 |
| 55 | 10,000 | Jackknife | H | 0.5 | NA | 0.98 | 0.97 | 0.05 | 0.01 | 0.04 | 0.22 | 0.17 | 0.04 | 0.04 |
| 55 | 10,000 | Jackknife | LQH | 0.5 | NA | 0.98 | 0.97 | 0.05 | 0.01 | 0.04 | 0.22 | 0.17 | 0.04 | 0.04 |
| 55 | 10,000 | Jackknife | LQHP | 0.5 | NA | 0.98 | 0.97 | 0.05 | 0.01 | 0.04 | 0.25 | 0.19 | 0.04 | 0.04 |
| 55 | 10,000 | Jackknife | H | 0.6 | NA | 0.98 | 0.97 | 0.06 | 0.02 | 0.04 | 0.20 | 0.16 | 0.04 | 0.04 |
| 55 | 10,000 | Jackknife | H | 0.7 | NA | 0.98 | 0.97 | 0.06 | 0.02 | 0.05 | 0.20 | 0.16 | 0.04 | 0.04 |
| 55 | 10,000 | Jackknife | H | 0.8 | NA | 0.98 | 0.97 | 0.06 | 0.02 | 0.05 | 0.16 | 0.14 | 0.05 | 0.05 |
| 55 | 10,000 | Jackknife | H | 0.9 | NA | 0.98 | 0.97 | 0.07 | 0.02 | 0.05 | 0.16 | 0.14 | 0.05 | 0.05 |
| 55 | 10,000 | Jackknife | H | 1 | NA | 0.97 | 0.97 | 0.08 | 0.02 | 0.05 | 0.15 | 0.13 | 0.05 | 0.05 |
| 55 | 10,000 | Jackknife | H | 1.1 | NA | 0.97 | 0.97 | 0.08 | 0.02 | 0.06 | 0.15 | 0.13 | 0.05 | 0.05 |
| 55 | 10,000 | Jackknife | H | 1.6 | NA | 0.97 | 0.96 | 0.09 | 0.02 | 0.06 | 0.15 | 0.13 | 0.02 | 0.02 |
| 55 | 10,000 | Random-k-fold | LQHPT | 1.4 | 0.00 | 0.98 | 0.97 | 0.00 | 0.01 | 0.00 | 0.16 | 0.00 | 0.07 | 0.00 |
| 55 | 10,000 | Random-k-fold | LQ | 1 | 4.63 | 0.97 | 0.96 | 0.00 | 0.00 | 0.00 | 0.11 | 0.00 | 0.05 | 0.00 |
| 55 | 10,000 | Random-k-fold | LQ | 0.6 | 5.17 | 0.97 | 0.97 | 0.00 | 0.00 | 0.00 | 0.11 | 0.00 | 0.04 | 0.00 |
| 55 | 10,000 | Random-k-fold | LQ | 0.7 | 5.98 | 0.97 | 0.97 | 0.00 | 0.00 | 0.00 | 0.11 | 0.00 | 0.04 | 0.00 |
| 55 | 10,000 | Random-k-fold | LQ | 1.1 | 6.20 | 0.97 | 0.96 | 0.00 | 0.00 | 0.00 | 0.11 | 0.00 | 0.02 | 0.00 |
| 55 | 10,000 | Random-k-fold | LQ | 0.9 | 7.18 | 0.97 | 0.96 | 0.00 | 0.00 | 0.00 | 0.11 | 0.00 | 0.04 | 0.00 |
| 55 | 10,000 | Random-k-fold | LQ | 1.2 | 7.76 | 0.97 | 0.96 | 0.00 | 0.00 | 0.00 | 0.11 | 0.00 | 0.02 | 0.00 |
| 55 | 10,000 | Random-k-fold | LQ | 0.5 | 7.96 | 0.97 | 0.97 | 0.00 | 0.00 | 0.00 | 0.11 | 0.00 | 0.04 | 0.00 |
| 55 | 10,000 | Random-k-fold | LQ | 0.1 | 9.38 | 0.97 | 0.96 | 0.00 | 0.01 | 0.00 | 0.11 | 0.00 | 0.05 | 0.00 |
| 55 | 10,000 | Random-k-fold | LQ | 0.2 | 9.38 | 0.97 | 0.96 | 0.00 | 0.01 | 0.00 | 0.11 | 0.00 | 0.05 | 0.00 |
| 55 | 10,000 | Random-k-fold | LQ | 0.3 | 9.38 | 0.97 | 0.96 | 0.00 | 0.01 | 0.00 | 0.11 | 0.00 | 0.05 | 0.00 |
| 55 | 10,000 | Random-k-fold | LQ | 1.3 | 9.74 | 0.97 | 0.96 | 0.00 | 0.00 | 0.00 | 0.13 | 0.00 | 0.02 | 0.00 |
| 55 | 10,000 | Random-k-fold | LQ | 0.4 | 10.48 | 0.97 | 0.97 | 0.00 | 0.00 | 0.00 | 0.11 | 0.00 | 0.05 | 0.00 |
| 55 | 10,000 | Random-k-fold | LQ | 1.4 | 11.65 | 0.97 | 0.96 | 0.00 | 0.00 | 0.00 | 0.13 | 0.00 | 0.02 | 0.00 |
| 55 | 10,000 | Random-k-fold | LQ | 0.8 | 12.71 | 0.97 | 0.97 | 0.00 | 0.00 | 0.00 | 0.11 | 0.00 | 0.04 | 0.00 |
| 55 | 10,000 | Random-k-fold | LQ | 1.5 | 13.40 | 0.97 | 0.96 | 0.00 | 0.00 | 0.00 | 0.13 | 0.00 | 0.02 | 0.00 |
| 55 | 10,000 | Random-k-fold | LQ | 1.6 | 15.67 | 0.97 | 0.96 | 0.00 | 0.00 | 0.00 | 0.13 | 0.00 | 0.02 | 0.00 |
| 55 | 10,000 | Random-k-fold | LQ | 1.7 | 17.85 | 0.97 | 0.96 | 0.00 | 0.00 | 0.00 | 0.13 | 0.00 | 0.02 | 0.00 |
| 55 | 10,000 | Random-k-fold | LQHPT | 1.5 | 18.22 | 0.98 | 0.97 | 0.00 | 0.01 | 0.00 | 0.16 | 0.00 | 0.05 | 0.00 |
| 55 | 10,000 | Random-k-fold | LQ | 1.8 | 19.38 | 0.96 | 0.96 | 0.00 | 0.00 | 0.00 | 0.13 | 0.00 | 0.02 | 0.00 |
| 55 | 10,000 | Random-k-fold | LQHPT | 1.1 | 21.13 | 0.98 | 0.97 | 0.00 | 0.01 | 0.00 | 0.18 | 0.00 | 0.09 | 0.01 |
| 55 | 10,000 | Random-k-fold | LQ | 1.9 | 21.68 | 0.96 | 0.96 | 0.00 | 0.00 | 0.00 | 0.13 | 0.00 | 0.02 | 0.00 |
| 55 | 10,000 | Random-k-fold | LQ | 2 | 23.67 | 0.96 | 0.96 | 0.00 | 0.00 | 0.00 | 0.13 | 0.00 | 0.02 | 0.00 |
| 55 | 10,000 | Random-k-fold | LQHPT | 1.3 | 24.33 | 0.98 | 0.97 | 0.00 | 0.01 | 0.00 | 0.16 | 0.00 | 0.07 | 0.00 |
| 55 | 10,000 | Random-k-fold | LQHP | 1.3 | 24.33 | 0.97 | 0.97 | 0.00 | 0.00 | 0.00 | 0.15 | 0.00 | 0.09 | 0.00 |
| 55 | 10,000 | Random-k-fold | LQHP | 1.2 | 32.23 | 0.97 | 0.97 | 0.00 | 0.00 | 0.00 | 0.13 | 0.00 | 0.09 | 0.00 |
| 55 | 10,000 | Random-k-fold | LQH | 1.6 | 33.46 | 0.97 | 0.97 | 0.00 | 0.00 | 0.00 | 0.16 | 0.00 | 0.09 | 0.01 |
| 55 | 10,000 | Random-k-fold | LQHP | 1.1 | 33.91 | 0.97 | 0.97 | 0.00 | 0.00 | 0.00 | 0.13 | 0.00 | 0.07 | 0.00 |
| 55 | 10,000 | Random-k-fold | LQHP | 1 | 37.32 | 0.98 | 0.97 | 0.00 | 0.00 | 0.00 | 0.13 | 0.00 | 0.07 | 0.00 |
| 55 | 10,000 | Random-k-fold | LQHP | 1.5 | 37.93 | 0.97 | 0.97 | 0.00 | 0.00 | 0.00 | 0.15 | 0.00 | 0.07 | 0.00 |
| 55 | 10,000 | Random-k-fold | LQHPT | 1.8 | 38.03 | 0.98 | 0.97 | 0.00 | 0.01 | 0.00 | 0.16 | 0.00 | 0.07 | 0.00 |
| 55 | 10,000 | Random-k-fold | LQHP | 2 | 38.33 | 0.97 | 0.97 | 0.00 | 0.00 | 0.00 | 0.15 | 0.00 | 0.07 | 0.01 |
| 55 | 10,000 | Random-k-fold | LQHPT | 1.6 | 39.98 | 0.98 | 0.97 | 0.00 | 0.01 | 0.00 | 0.16 | 0.00 | 0.07 | 0.00 |
| 55 | 10,000 | Random-k-fold | LQHPT | 1.2 | 40.75 | 0.98 | 0.97 | 0.00 | 0.01 | 0.00 | 0.16 | 0.00 | 0.09 | 0.01 |
| 55 | 10,000 | Random-k-fold | LQH | 1.4 | 40.84 | 0.97 | 0.97 | 0.00 | 0.01 | 0.00 | 0.15 | 0.00 | 0.09 | 0.01 |
| 55 | 10,000 | Random-k-fold | LQHP | 1.9 | 40.89 | 0.97 | 0.97 | 0.00 | 0.00 | 0.00 | 0.15 | 0.00 | 0.07 | 0.00 |
| 55 | 10,000 | Random-k-fold | LQHPT | 1.7 | 42.44 | 0.98 | 0.97 | 0.00 | 0.01 | 0.00 | 0.16 | 0.00 | 0.07 | 0.00 |
| 55 | 10,000 | Random-k-fold | LQH | 1.7 | 45.25 | 0.97 | 0.96 | 0.00 | 0.00 | 0.00 | 0.16 | 0.00 | 0.09 | 0.01 |
| 55 | 10,000 | Random-k-fold | LQHP | 1.6 | 46.37 | 0.97 | 0.97 | 0.00 | 0.00 | 0.00 | 0.15 | 0.00 | 0.07 | 0.00 |
| 55 | 10,000 | Random-k-fold | LQH | 1.5 | 46.63 | 0.97 | 0.97 | 0.00 | 0.00 | 0.00 | 0.13 | 0.00 | 0.09 | 0.01 |
| 55 | 10,000 | Random-k-fold | LQHPT | 1.9 | 46.68 | 0.97 | 0.97 | 0.00 | 0.01 | 0.00 | 0.16 | 0.00 | 0.05 | 0.00 |
| 55 | 10,000 | Random-k-fold | LQH | 1.8 | 46.92 | 0.97 | 0.96 | 0.00 | 0.00 | 0.00 | 0.16 | 0.00 | 0.09 | 0.01 |
| 55 | 10,000 | Random-k-fold | LQHP | 1.4 | 48.18 | 0.97 | 0.97 | 0.00 | 0.00 | 0.00 | 0.15 | 0.00 | 0.07 | 0.00 |
| 55 | 10,000 | Random-k-fold | LQHPT | 1 | 52.41 | 0.98 | 0.97 | 0.00 | 0.01 | 0.00 | 0.23 | 0.01 | 0.09 | 0.01 |
| 55 | 10,000 | Random-k-fold | LQHPT | 2 | 55.86 | 0.97 | 0.97 | 0.00 | 0.01 | 0.00 | 0.16 | 0.00 | 0.05 | 0.00 |
| 55 | 10,000 | Random-k-fold | LQHP | 1.8 | 56.03 | 0.97 | 0.97 | 0.00 | 0.00 | 0.00 | 0.15 | 0.00 | 0.07 | 0.00 |
| 55 | 10,000 | Random-k-fold | LQH | 2 | 57.46 | 0.97 | 0.96 | 0.00 | 0.00 | 0.00 | 0.15 | 0.00 | 0.09 | 0.01 |
| 55 | 10,000 | Random-k-fold | LQHP | 1.7 | 67.72 | 0.97 | 0.97 | 0.00 | 0.00 | 0.00 | 0.15 | 0.00 | 0.07 | 0.01 |
| 55 | 10,000 | Random-k-fold | LQH | 1.9 | 67.89 | 0.97 | 0.96 | 0.00 | 0.00 | 0.00 | 0.16 | 0.00 | 0.09 | 0.01 |
| 55 | 10,000 | Random-k-fold | LQHPT | 0.9 | 78.07 | 0.98 | 0.97 | 0.00 | 0.01 | 0.00 | 0.23 | 0.01 | 0.09 | 0.01 |
| 55 | 10,000 | Random-k-fold | LQH | 1.3 | 86.28 | 0.97 | 0.97 | 0.00 | 0.01 | 0.00 | 0.13 | 0.00 | 0.11 | 0.00 |
| 55 | 10,000 | Random-k-fold | LQH | 1.2 | 93.10 | 0.97 | 0.97 | 0.00 | 0.01 | 0.00 | 0.13 | 0.00 | 0.11 | 0.00 |
| 55 | 10,000 | Random-k-fold | LQHP | 0.8 | 104.22 | 0.98 | 0.97 | 0.00 | 0.01 | 0.00 | 0.16 | 0.00 | 0.05 | 0.00 |
| 55 | 10,000 | Random-k-fold | LQHP | 0.7 | 110.50 | 0.98 | 0.97 | 0.00 | 0.01 | 0.00 | 0.18 | 0.00 | 0.05 | 0.00 |
| 55 | 10,000 | Random-k-fold | LQHP | 0.9 | 123.27 | 0.98 | 0.97 | 0.00 | 0.01 | 0.00 | 0.13 | 0.00 | 0.07 | 0.00 |
| 55 | 10,000 | Random-k-fold | L | 0.6 | 144.74 | 0.90 | 0.90 | 0.00 | 0.01 | 0.00 | 0.13 | 0.01 | 0.02 | 0.00 |
| 55 | 10,000 | Random-k-fold | L | 0.1 | 144.79 | 0.90 | 0.90 | 0.00 | 0.01 | 0.00 | 0.13 | 0.01 | 0.02 | 0.00 |
| 55 | 10,000 | Random-k-fold | L | 0.2 | 144.79 | 0.90 | 0.90 | 0.00 | 0.01 | 0.00 | 0.13 | 0.01 | 0.02 | 0.00 |
| 55 | 10,000 | Random-k-fold | L | 0.3 | 144.79 | 0.90 | 0.90 | 0.00 | 0.01 | 0.00 | 0.13 | 0.01 | 0.02 | 0.00 |
| 55 | 10,000 | Random-k-fold | L | 0.4 | 144.79 | 0.90 | 0.90 | 0.00 | 0.01 | 0.00 | 0.13 | 0.01 | 0.02 | 0.00 |
| 55 | 10,000 | Random-k-fold | L | 0.5 | 144.79 | 0.90 | 0.90 | 0.00 | 0.01 | 0.00 | 0.13 | 0.01 | 0.02 | 0.00 |
| 55 | 10,000 | Random-k-fold | L | 0.7 | 144.89 | 0.90 | 0.90 | 0.00 | 0.01 | 0.00 | 0.13 | 0.01 | 0.02 | 0.00 |
| 55 | 10,000 | Random-k-fold | L | 0.8 | 145.27 | 0.90 | 0.90 | 0.00 | 0.01 | 0.00 | 0.13 | 0.01 | 0.02 | 0.00 |
| 55 | 10,000 | Random-k-fold | L | 0.9 | 145.53 | 0.90 | 0.90 | 0.00 | 0.01 | 0.00 | 0.13 | 0.01 | 0.02 | 0.00 |
| 55 | 10,000 | Random-k-fold | L | 1 | 145.82 | 0.90 | 0.90 | 0.00 | 0.01 | 0.00 | 0.13 | 0.01 | 0.02 | 0.00 |
| 55 | 10,000 | Random-k-fold | L | 1.1 | 146.32 | 0.90 | 0.90 | 0.00 | 0.01 | 0.00 | 0.13 | 0.01 | 0.02 | 0.00 |
| 55 | 10,000 | Random-k-fold | L | 1.2 | 147.16 | 0.90 | 0.90 | 0.00 | 0.01 | 0.00 | 0.13 | 0.01 | 0.02 | 0.00 |
| 55 | 10,000 | Random-k-fold | L | 1.7 | 147.47 | 0.90 | 0.89 | 0.00 | 0.01 | 0.00 | 0.13 | 0.01 | 0.02 | 0.00 |
| 55 | 10,000 | Random-k-fold | L | 1.5 | 147.81 | 0.90 | 0.90 | 0.00 | 0.01 | 0.00 | 0.13 | 0.01 | 0.02 | 0.00 |
| 55 | 10,000 | Random-k-fold | L | 1.3 | 148.24 | 0.90 | 0.90 | 0.00 | 0.01 | 0.00 | 0.13 | 0.01 | 0.02 | 0.00 |
| 55 | 10,000 | Random-k-fold | L | 1.8 | 148.38 | 0.90 | 0.89 | 0.00 | 0.01 | 0.00 | 0.13 | 0.01 | 0.02 | 0.00 |
| 55 | 10,000 | Random-k-fold | L | 1.6 | 148.92 | 0.90 | 0.90 | 0.00 | 0.01 | 0.00 | 0.13 | 0.01 | 0.02 | 0.00 |
| 55 | 10,000 | Random-k-fold | L | 1.4 | 149.34 | 0.90 | 0.90 | 0.00 | 0.01 | 0.00 | 0.13 | 0.01 | 0.02 | 0.00 |
| 55 | 10,000 | Random-k-fold | L | 1.9 | 149.35 | 0.90 | 0.89 | 0.00 | 0.01 | 0.00 | 0.13 | 0.01 | 0.02 | 0.00 |
| 55 | 10,000 | Random-k-fold | L | 2 | 150.07 | 0.90 | 0.89 | 0.00 | 0.01 | 0.00 | 0.13 | 0.01 | 0.02 | 0.00 |
| 55 | 10,000 | Random-k-fold | LQH | 1.1 | 163.90 | 0.97 | 0.97 | 0.00 | 0.01 | 0.00 | 0.15 | 0.00 | 0.11 | 0.00 |
| 55 | 10,000 | Random-k-fold | LQH | 1 | 217.07 | 0.98 | 0.97 | 0.00 | 0.01 | 0.00 | 0.15 | 0.00 | 0.11 | 0.00 |
| 55 | 10,000 | Random-k-fold | LQH | 0.7 | 222.92 | 0.98 | 0.97 | 0.00 | 0.01 | 0.00 | 0.18 | 0.00 | 0.09 | 0.00 |
| 55 | 10,000 | Random-k-fold | LQHPT | 0.7 | 246.55 | 0.99 | 0.97 | 0.00 | 0.02 | 0.00 | 0.31 | 0.01 | 0.11 | 0.01 |
| 55 | 10,000 | Random-k-fold | LQHPT | 0.8 | 293.67 | 0.98 | 0.97 | 0.00 | 0.02 | 0.00 | 0.27 | 0.01 | 0.09 | 0.01 |
| 55 | 10,000 | Random-k-fold | LQH | 0.8 | 328.56 | 0.98 | 0.97 | 0.00 | 0.01 | 0.00 | 0.16 | 0.00 | 0.09 | 0.00 |
| 55 | 10,000 | Random-k-fold | H | 1.4 | 362.06 | 0.97 | 0.97 | 0.00 | 0.00 | 0.00 | 0.15 | 0.00 | 0.09 | 0.00 |
| 55 | 10,000 | Random-k-fold | H | 2 | 472.34 | 0.97 | 0.96 | 0.00 | 0.00 | 0.00 | 0.15 | 0.00 | 0.04 | 0.00 |
| 55 | 10,000 | Random-k-fold | LQH | 0.9 | 582.77 | 0.98 | 0.97 | 0.00 | 0.01 | 0.00 | 0.15 | 0.00 | 0.11 | 0.00 |
| 55 | 10,000 | Random-k-fold | H | 1.3 | 606.40 | 0.97 | 0.97 | 0.00 | 0.00 | 0.00 | 0.15 | 0.00 | 0.09 | 0.01 |
| 55 | 10,000 | Random-k-fold | LQHP | 0.6 | 668.11 | 0.98 | 0.97 | 0.00 | 0.01 | 0.00 | 0.20 | 0.01 | 0.05 | 0.00 |
| 55 | 10,000 | Random-k-fold | H | 1.7 | 721.23 | 0.97 | 0.96 | 0.00 | 0.00 | 0.00 | 0.15 | 0.00 | 0.05 | 0.00 |
| 55 | 10,000 | Random-k-fold | LQHPT | 0.6 | 768.44 | 0.99 | 0.97 | 0.00 | 0.02 | 0.00 | 0.34 | 0.02 | 0.13 | 0.01 |
| 55 | 10,000 | Random-k-fold | LQH | 0.6 | 1304.64 | 0.98 | 0.97 | 0.00 | 0.01 | 0.00 | 0.22 | 0.01 | 0.07 | 0.00 |
| 55 | 10,000 | Random-k-fold | H | 1.5 | 1354.10 | 0.97 | 0.97 | 0.00 | 0.00 | 0.00 | 0.15 | 0.00 | 0.05 | 0.00 |
| 55 | 10,000 | Random-k-fold | H | 1.2 | 1838.75 | 0.97 | 0.97 | 0.00 | 0.00 | 0.00 | 0.15 | 0.00 | 0.09 | 0.01 |
| 55 | 10,000 | Random-k-fold | LQHPT | 0.5 | 2736.81 | 0.99 | 0.96 | 0.00 | 0.02 | 0.00 | 0.40 | 0.00 | 0.15 | 0.01 |
| 55 | 10,000 | Random-k-fold | H | 1.8 | 2844.43 | 0.97 | 0.96 | 0.00 | 0.00 | 0.00 | 0.15 | 0.00 | 0.05 | 0.00 |
| 55 | 10,000 | Random-k-fold | H | 1.9 | 5816.50 | 0.97 | 0.96 | 0.00 | 0.00 | 0.00 | 0.15 | 0.00 | 0.04 | 0.00 |
| 55 | 10,000 | Random-k-fold | H | 0.1 | NA | 0.99 | 0.97 | 0.00 | 0.01 | 0.00 | 0.33 | 0.01 | 0.13 | 0.02 |
| 55 | 10,000 | Random-k-fold | LQH | 0.1 | NA | 0.99 | 0.97 | 0.00 | 0.02 | 0.00 | 0.33 | 0.01 | 0.13 | 0.02 |
| 55 | 10,000 | Random-k-fold | LQHP | 0.1 | NA | 0.99 | 0.97 | 0.00 | 0.02 | 0.00 | 0.31 | 0.00 | 0.13 | 0.02 |
| 55 | 10,000 | Random-k-fold | LQHPT | 0.1 | NA | 0.99 | 0.94 | 0.00 | 0.05 | 0.00 | 0.71 | 0.03 | 0.29 | 0.01 |
| 55 | 10,000 | Random-k-fold | H | 0.2 | NA | 0.99 | 0.97 | 0.00 | 0.01 | 0.00 | 0.27 | 0.01 | 0.11 | 0.02 |
| 55 | 10,000 | Random-k-fold | LQH | 0.2 | NA | 0.99 | 0.97 | 0.00 | 0.01 | 0.00 | 0.25 | 0.01 | 0.11 | 0.02 |
| 55 | 10,000 | Random-k-fold | LQHP | 0.2 | NA | 0.99 | 0.97 | 0.00 | 0.01 | 0.00 | 0.23 | 0.01 | 0.11 | 0.02 |
| 55 | 10,000 | Random-k-fold | LQHPT | 0.2 | NA | 0.99 | 0.94 | 0.00 | 0.05 | 0.00 | 0.66 | 0.02 | 0.23 | 0.02 |
| 55 | 10,000 | Random-k-fold | H | 0.3 | NA | 0.99 | 0.97 | 0.00 | 0.01 | 0.00 | 0.22 | 0.01 | 0.05 | 0.00 |
| 55 | 10,000 | Random-k-fold | LQH | 0.3 | NA | 0.99 | 0.97 | 0.00 | 0.01 | 0.00 | 0.23 | 0.01 | 0.07 | 0.00 |
| 55 | 10,000 | Random-k-fold | LQHP | 0.3 | NA | 0.99 | 0.97 | 0.00 | 0.01 | 0.00 | 0.23 | 0.01 | 0.09 | 0.01 |
| 55 | 10,000 | Random-k-fold | LQHPT | 0.3 | NA | 0.99 | 0.95 | 0.00 | 0.04 | 0.00 | 0.60 | 0.03 | 0.23 | 0.02 |
| 55 | 10,000 | Random-k-fold | H | 0.4 | NA | 0.98 | 0.97 | 0.00 | 0.01 | 0.00 | 0.23 | 0.02 | 0.05 | 0.00 |
| 55 | 10,000 | Random-k-fold | LQH | 0.4 | NA | 0.98 | 0.97 | 0.00 | 0.01 | 0.00 | 0.23 | 0.02 | 0.05 | 0.00 |
| 55 | 10,000 | Random-k-fold | LQHP | 0.4 | NA | 0.98 | 0.97 | 0.00 | 0.01 | 0.00 | 0.25 | 0.01 | 0.05 | 0.00 |
| 55 | 10,000 | Random-k-fold | LQHPT | 0.4 | NA | 0.99 | 0.96 | 0.00 | 0.03 | 0.00 | 0.45 | 0.02 | 0.16 | 0.01 |
| 55 | 10,000 | Random-k-fold | H | 0.5 | NA | 0.98 | 0.97 | 0.00 | 0.01 | 0.00 | 0.25 | 0.02 | 0.04 | 0.00 |
| 55 | 10,000 | Random-k-fold | LQH | 0.5 | NA | 0.98 | 0.97 | 0.00 | 0.01 | 0.00 | 0.23 | 0.02 | 0.05 | 0.00 |
| 55 | 10,000 | Random-k-fold | LQHP | 0.5 | NA | 0.98 | 0.97 | 0.00 | 0.01 | 0.00 | 0.23 | 0.02 | 0.05 | 0.00 |
| 55 | 10,000 | Random-k-fold | H | 0.6 | NA | 0.98 | 0.97 | 0.00 | 0.01 | 0.00 | 0.23 | 0.01 | 0.04 | 0.00 |
| 55 | 10,000 | Random-k-fold | H | 0.7 | NA | 0.98 | 0.97 | 0.00 | 0.01 | 0.00 | 0.22 | 0.01 | 0.07 | 0.00 |
| 55 | 10,000 | Random-k-fold | H | 0.8 | NA | 0.98 | 0.97 | 0.00 | 0.01 | 0.00 | 0.18 | 0.00 | 0.09 | 0.01 |
| 55 | 10,000 | Random-k-fold | H | 0.9 | NA | 0.98 | 0.97 | 0.00 | 0.01 | 0.00 | 0.15 | 0.00 | 0.09 | 0.01 |
| 55 | 10,000 | Random-k-fold | H | 1 | NA | 0.97 | 0.97 | 0.00 | 0.01 | 0.00 | 0.15 | 0.00 | 0.11 | 0.00 |
| 55 | 10,000 | Random-k-fold | H | 1.1 | NA | 0.97 | 0.97 | 0.00 | 0.01 | 0.00 | 0.15 | 0.00 | 0.11 | 0.00 |
| 55 | 10,000 | Random-k-fold | H | 1.6 | NA | 0.97 | 0.97 | 0.00 | 0.00 | 0.00 | 0.15 | 0.00 | 0.05 | 0.00 |
| 55 | 10,000 | Block | LQHPT | 1.4 | 0.00 | 0.98 | 0.96 | 0.00 | 0.01 | 0.00 | 0.14 | 0.06 | 0.09 | 0.03 |
| 55 | 10,000 | Block | LQ | 1 | 4.63 | 0.97 | 0.96 | 0.00 | 0.01 | 0.00 | 0.13 | 0.06 | 0.09 | 0.03 |
| 55 | 10,000 | Block | LQ | 0.6 | 5.17 | 0.97 | 0.96 | 0.00 | 0.01 | 0.00 | 0.13 | 0.06 | 0.13 | 0.06 |
| 55 | 10,000 | Block | LQ | 0.7 | 5.98 | 0.97 | 0.96 | 0.00 | 0.01 | 0.00 | 0.13 | 0.06 | 0.13 | 0.06 |
| 55 | 10,000 | Block | LQ | 1.1 | 6.20 | 0.97 | 0.96 | 0.00 | 0.01 | 0.00 | 0.11 | 0.05 | 0.07 | 0.02 |
| 55 | 10,000 | Block | LQ | 0.9 | 7.18 | 0.97 | 0.96 | 0.00 | 0.01 | 0.00 | 0.13 | 0.06 | 0.11 | 0.05 |
| 55 | 10,000 | Block | LQ | 1.2 | 7.76 | 0.97 | 0.96 | 0.00 | 0.01 | 0.00 | 0.09 | 0.03 | 0.04 | 0.01 |
| 55 | 10,000 | Block | LQ | 0.5 | 7.96 | 0.97 | 0.96 | 0.00 | 0.02 | 0.00 | 0.13 | 0.06 | 0.13 | 0.06 |
| 55 | 10,000 | Block | LQ | 0.1 | 9.38 | 0.97 | 0.95 | 0.00 | 0.02 | 0.00 | 0.13 | 0.06 | 0.13 | 0.06 |
| 55 | 10,000 | Block | LQ | 0.2 | 9.38 | 0.97 | 0.95 | 0.00 | 0.02 | 0.00 | 0.13 | 0.06 | 0.13 | 0.06 |
| 55 | 10,000 | Block | LQ | 0.3 | 9.38 | 0.97 | 0.95 | 0.00 | 0.02 | 0.00 | 0.13 | 0.06 | 0.13 | 0.06 |
| 55 | 10,000 | Block | LQ | 1.3 | 9.74 | 0.97 | 0.96 | 0.00 | 0.01 | 0.00 | 0.09 | 0.03 | 0.04 | 0.01 |
| 55 | 10,000 | Block | LQ | 0.4 | 10.48 | 0.97 | 0.96 | 0.00 | 0.02 | 0.00 | 0.13 | 0.06 | 0.13 | 0.06 |
| 55 | 10,000 | Block | LQ | 1.4 | 11.65 | 0.97 | 0.96 | 0.00 | 0.00 | 0.00 | 0.09 | 0.03 | 0.04 | 0.01 |
| 55 | 10,000 | Block | LQ | 0.8 | 12.71 | 0.97 | 0.96 | 0.00 | 0.01 | 0.00 | 0.13 | 0.06 | 0.13 | 0.06 |
| 55 | 10,000 | Block | LQ | 1.5 | 13.40 | 0.97 | 0.96 | 0.00 | 0.00 | 0.00 | 0.07 | 0.02 | 0.04 | 0.01 |
| 55 | 10,000 | Block | LQ | 1.6 | 15.67 | 0.97 | 0.96 | 0.00 | 0.00 | 0.00 | 0.07 | 0.02 | 0.04 | 0.01 |
| 55 | 10,000 | Block | LQ | 1.7 | 17.85 | 0.97 | 0.96 | 0.00 | 0.00 | 0.00 | 0.07 | 0.02 | 0.04 | 0.01 |
| 55 | 10,000 | Block | LQHPT | 1.5 | 18.22 | 0.98 | 0.97 | 0.00 | 0.01 | 0.00 | 0.13 | 0.06 | 0.09 | 0.03 |
| 55 | 10,000 | Block | LQ | 1.8 | 19.38 | 0.96 | 0.96 | 0.00 | 0.00 | 0.00 | 0.09 | 0.03 | 0.04 | 0.01 |
| 55 | 10,000 | Block | LQHPT | 1.1 | 21.13 | 0.98 | 0.97 | 0.00 | 0.01 | 0.00 | 0.16 | 0.06 | 0.11 | 0.05 |
| 55 | 10,000 | Block | LQ | 1.9 | 21.68 | 0.96 | 0.96 | 0.00 | 0.00 | 0.00 | 0.09 | 0.03 | 0.04 | 0.01 |
| 55 | 10,000 | Block | LQ | 2 | 23.67 | 0.96 | 0.96 | 0.00 | 0.00 | 0.00 | 0.09 | 0.03 | 0.04 | 0.01 |
| 55 | 10,000 | Block | LQHPT | 1.3 | 24.33 | 0.98 | 0.96 | 0.00 | 0.01 | 0.00 | 0.14 | 0.06 | 0.09 | 0.03 |
| 55 | 10,000 | Block | LQHP | 1.3 | 24.33 | 0.97 | 0.97 | 0.00 | 0.01 | 0.00 | 0.11 | 0.05 | 0.04 | 0.01 |
| 55 | 10,000 | Block | LQHP | 1.2 | 32.23 | 0.97 | 0.97 | 0.00 | 0.01 | 0.00 | 0.13 | 0.06 | 0.05 | 0.01 |
| 55 | 10,000 | Block | LQH | 1.6 | 33.46 | 0.97 | 0.96 | 0.00 | 0.01 | 0.00 | 0.13 | 0.06 | 0.09 | 0.03 |
| 55 | 10,000 | Block | LQHP | 1.1 | 33.91 | 0.97 | 0.97 | 0.00 | 0.01 | 0.00 | 0.13 | 0.06 | 0.05 | 0.01 |
| 55 | 10,000 | Block | LQHP | 1 | 37.32 | 0.98 | 0.97 | 0.00 | 0.01 | 0.00 | 0.13 | 0.06 | 0.05 | 0.01 |
| 55 | 10,000 | Block | LQHP | 1.5 | 37.93 | 0.97 | 0.97 | 0.00 | 0.01 | 0.00 | 0.07 | 0.02 | 0.04 | 0.01 |
| 55 | 10,000 | Block | LQHPT | 1.8 | 38.03 | 0.98 | 0.97 | 0.00 | 0.01 | 0.00 | 0.13 | 0.06 | 0.09 | 0.03 |
| 55 | 10,000 | Block | LQHP | 2 | 38.33 | 0.97 | 0.97 | 0.00 | 0.01 | 0.00 | 0.05 | 0.01 | 0.02 | 0.00 |
| 55 | 10,000 | Block | LQHPT | 1.6 | 39.98 | 0.98 | 0.97 | 0.00 | 0.01 | 0.00 | 0.13 | 0.06 | 0.09 | 0.03 |
| 55 | 10,000 | Block | LQHPT | 1.2 | 40.75 | 0.98 | 0.96 | 0.00 | 0.01 | 0.00 | 0.14 | 0.06 | 0.09 | 0.03 |
| 55 | 10,000 | Block | LQH | 1.4 | 40.84 | 0.97 | 0.96 | 0.00 | 0.01 | 0.00 | 0.14 | 0.06 | 0.11 | 0.05 |
| 55 | 10,000 | Block | LQHP | 1.9 | 40.89 | 0.97 | 0.97 | 0.00 | 0.01 | 0.00 | 0.07 | 0.02 | 0.02 | 0.00 |
| 55 | 10,000 | Block | LQHPT | 1.7 | 42.44 | 0.98 | 0.97 | 0.00 | 0.01 | 0.00 | 0.13 | 0.06 | 0.09 | 0.03 |
| 55 | 10,000 | Block | LQH | 1.7 | 45.25 | 0.97 | 0.96 | 0.00 | 0.01 | 0.00 | 0.13 | 0.06 | 0.07 | 0.02 |
| 55 | 10,000 | Block | LQHP | 1.6 | 46.37 | 0.97 | 0.97 | 0.00 | 0.01 | 0.00 | 0.07 | 0.02 | 0.04 | 0.01 |
| 55 | 10,000 | Block | LQH | 1.5 | 46.63 | 0.97 | 0.96 | 0.00 | 0.01 | 0.00 | 0.14 | 0.06 | 0.13 | 0.06 |
| 55 | 10,000 | Block | LQHPT | 1.9 | 46.68 | 0.97 | 0.97 | 0.00 | 0.01 | 0.00 | 0.13 | 0.06 | 0.09 | 0.03 |
| 55 | 10,000 | Block | LQH | 1.8 | 46.92 | 0.97 | 0.96 | 0.00 | 0.01 | 0.00 | 0.13 | 0.06 | 0.07 | 0.02 |
| 55 | 10,000 | Block | LQHP | 1.4 | 48.18 | 0.97 | 0.97 | 0.00 | 0.01 | 0.00 | 0.11 | 0.05 | 0.04 | 0.01 |
| 55 | 10,000 | Block | LQHPT | 1 | 52.41 | 0.98 | 0.96 | 0.00 | 0.02 | 0.00 | 0.18 | 0.06 | 0.13 | 0.06 |
| 55 | 10,000 | Block | LQHPT | 2 | 55.86 | 0.97 | 0.97 | 0.00 | 0.01 | 0.00 | 0.13 | 0.06 | 0.09 | 0.03 |
| 55 | 10,000 | Block | LQHP | 1.8 | 56.03 | 0.97 | 0.97 | 0.00 | 0.00 | 0.00 | 0.05 | 0.01 | 0.02 | 0.00 |
| 55 | 10,000 | Block | LQH | 2 | 57.46 | 0.97 | 0.96 | 0.00 | 0.01 | 0.00 | 0.13 | 0.06 | 0.04 | 0.01 |
| 55 | 10,000 | Block | LQHP | 1.7 | 67.72 | 0.97 | 0.97 | 0.00 | 0.01 | 0.00 | 0.07 | 0.02 | 0.04 | 0.01 |
| 55 | 10,000 | Block | LQH | 1.9 | 67.89 | 0.97 | 0.96 | 0.00 | 0.01 | 0.00 | 0.13 | 0.06 | 0.04 | 0.01 |
| 55 | 10,000 | Block | LQHPT | 0.9 | 78.07 | 0.98 | 0.96 | 0.00 | 0.02 | 0.00 | 0.21 | 0.06 | 0.13 | 0.06 |
| 55 | 10,000 | Block | LQH | 1.3 | 86.28 | 0.97 | 0.96 | 0.00 | 0.01 | 0.00 | 0.14 | 0.06 | 0.13 | 0.06 |
| 55 | 10,000 | Block | LQH | 1.2 | 93.10 | 0.97 | 0.96 | 0.00 | 0.01 | 0.00 | 0.14 | 0.06 | 0.13 | 0.06 |
| 55 | 10,000 | Block | LQHP | 0.8 | 104.22 | 0.98 | 0.97 | 0.00 | 0.01 | 0.00 | 0.16 | 0.05 | 0.05 | 0.01 |
| 55 | 10,000 | Block | LQHP | 0.7 | 110.50 | 0.98 | 0.97 | 0.00 | 0.01 | 0.00 | 0.20 | 0.05 | 0.05 | 0.01 |
| 55 | 10,000 | Block | LQHP | 0.9 | 123.27 | 0.98 | 0.97 | 0.00 | 0.01 | 0.00 | 0.14 | 0.06 | 0.05 | 0.01 |
| 55 | 10,000 | Block | L | 0.6 | 144.74 | 0.90 | 0.89 | 0.00 | 0.00 | 0.00 | 0.02 | 0.00 | 0.00 | 0.00 |
| 55 | 10,000 | Block | L | 0.1 | 144.79 | 0.90 | 0.89 | 0.00 | 0.00 | 0.00 | 0.02 | 0.00 | 0.00 | 0.00 |
| 55 | 10,000 | Block | L | 0.2 | 144.79 | 0.90 | 0.89 | 0.00 | 0.00 | 0.00 | 0.02 | 0.00 | 0.00 | 0.00 |
| 55 | 10,000 | Block | L | 0.3 | 144.79 | 0.90 | 0.89 | 0.00 | 0.00 | 0.00 | 0.02 | 0.00 | 0.00 | 0.00 |
| 55 | 10,000 | Block | L | 0.4 | 144.79 | 0.90 | 0.89 | 0.00 | 0.00 | 0.00 | 0.02 | 0.00 | 0.00 | 0.00 |
| 55 | 10,000 | Block | L | 0.5 | 144.79 | 0.90 | 0.89 | 0.00 | 0.00 | 0.00 | 0.02 | 0.00 | 0.00 | 0.00 |
| 55 | 10,000 | Block | L | 0.7 | 144.89 | 0.90 | 0.89 | 0.00 | 0.00 | 0.00 | 0.02 | 0.00 | 0.00 | 0.00 |
| 55 | 10,000 | Block | L | 0.8 | 145.27 | 0.90 | 0.89 | 0.00 | 0.00 | 0.00 | 0.02 | 0.00 | 0.00 | 0.00 |
| 55 | 10,000 | Block | L | 0.9 | 145.53 | 0.90 | 0.89 | 0.00 | 0.00 | 0.00 | 0.02 | 0.00 | 0.00 | 0.00 |
| 55 | 10,000 | Block | L | 1 | 145.82 | 0.90 | 0.89 | 0.00 | 0.00 | 0.00 | 0.02 | 0.00 | 0.00 | 0.00 |
| 55 | 10,000 | Block | L | 1.1 | 146.32 | 0.90 | 0.89 | 0.00 | 0.00 | 0.00 | 0.02 | 0.00 | 0.00 | 0.00 |
| 55 | 10,000 | Block | L | 1.2 | 147.16 | 0.90 | 0.89 | 0.00 | 0.00 | 0.00 | 0.02 | 0.00 | 0.00 | 0.00 |
| 55 | 10,000 | Block | L | 1.7 | 147.47 | 0.90 | 0.89 | 0.00 | 0.00 | 0.00 | 0.00 | 0.00 | 0.00 | 0.00 |
| 55 | 10,000 | Block | L | 1.5 | 147.81 | 0.90 | 0.89 | 0.00 | 0.00 | 0.00 | 0.02 | 0.00 | 0.00 | 0.00 |
| 55 | 10,000 | Block | L | 1.3 | 148.24 | 0.90 | 0.89 | 0.00 | 0.00 | 0.00 | 0.02 | 0.00 | 0.00 | 0.00 |
| 55 | 10,000 | Block | L | 1.8 | 148.38 | 0.90 | 0.88 | 0.00 | 0.00 | 0.00 | 0.00 | 0.00 | 0.00 | 0.00 |
| 55 | 10,000 | Block | L | 1.6 | 148.92 | 0.90 | 0.89 | 0.00 | 0.00 | 0.00 | 0.00 | 0.00 | 0.00 | 0.00 |
| 55 | 10,000 | Block | L | 1.4 | 149.34 | 0.90 | 0.89 | 0.00 | 0.00 | 0.00 | 0.02 | 0.00 | 0.00 | 0.00 |
| 55 | 10,000 | Block | L | 1.9 | 149.35 | 0.90 | 0.88 | 0.00 | 0.00 | 0.00 | 0.00 | 0.00 | 0.00 | 0.00 |
| 55 | 10,000 | Block | L | 2 | 150.07 | 0.90 | 0.88 | 0.00 | 0.00 | 0.00 | 0.00 | 0.00 | 0.00 | 0.00 |
| 55 | 10,000 | Block | LQH | 1.1 | 163.90 | 0.97 | 0.96 | 0.00 | 0.01 | 0.00 | 0.14 | 0.06 | 0.13 | 0.06 |
| 55 | 10,000 | Block | LQH | 1 | 217.07 | 0.98 | 0.97 | 0.00 | 0.01 | 0.00 | 0.14 | 0.06 | 0.13 | 0.06 |
| 55 | 10,000 | Block | LQH | 0.7 | 222.92 | 0.98 | 0.96 | 0.00 | 0.02 | 0.00 | 0.22 | 0.05 | 0.13 | 0.06 |
| 55 | 10,000 | Block | LQHPT | 0.7 | 246.55 | 0.99 | 0.96 | 0.00 | 0.02 | 0.00 | 0.25 | 0.05 | 0.14 | 0.06 |
| 55 | 10,000 | Block | LQHPT | 0.8 | 293.67 | 0.98 | 0.96 | 0.00 | 0.02 | 0.00 | 0.23 | 0.06 | 0.14 | 0.06 |
| 55 | 10,000 | Block | LQH | 0.8 | 328.56 | 0.98 | 0.96 | 0.00 | 0.02 | 0.00 | 0.20 | 0.05 | 0.13 | 0.06 |
| 55 | 10,000 | Block | H | 1.4 | 362.06 | 0.97 | 0.97 | 0.00 | 0.01 | 0.00 | 0.13 | 0.06 | 0.04 | 0.01 |
| 55 | 10,000 | Block | H | 2 | 472.34 | 0.97 | 0.96 | 0.00 | 0.01 | 0.00 | 0.13 | 0.06 | 0.05 | 0.01 |
| 55 | 10,000 | Block | LQH | 0.9 | 582.77 | 0.98 | 0.97 | 0.00 | 0.01 | 0.00 | 0.16 | 0.05 | 0.11 | 0.05 |
| 55 | 10,000 | Block | H | 1.3 | 606.40 | 0.97 | 0.97 | 0.00 | 0.01 | 0.00 | 0.13 | 0.06 | 0.04 | 0.01 |
| 55 | 10,000 | Block | LQHP | 0.6 | 668.11 | 0.98 | 0.97 | 0.00 | 0.01 | 0.00 | 0.23 | 0.06 | 0.05 | 0.01 |
| 55 | 10,000 | Block | H | 1.7 | 721.23 | 0.97 | 0.96 | 0.00 | 0.01 | 0.00 | 0.13 | 0.06 | 0.04 | 0.01 |
| 55 | 10,000 | Block | LQHPT | 0.6 | 768.44 | 0.99 | 0.96 | 0.00 | 0.02 | 0.00 | 0.27 | 0.04 | 0.14 | 0.06 |
| 55 | 10,000 | Block | LQH | 0.6 | 1304.64 | 0.98 | 0.97 | 0.00 | 0.01 | 0.00 | 0.23 | 0.06 | 0.13 | 0.06 |
| 55 | 10,000 | Block | H | 1.5 | 1354.10 | 0.97 | 0.96 | 0.00 | 0.01 | 0.00 | 0.13 | 0.06 | 0.04 | 0.01 |
| 55 | 10,000 | Block | H | 1.2 | 1838.75 | 0.97 | 0.97 | 0.00 | 0.01 | 0.00 | 0.13 | 0.06 | 0.04 | 0.01 |
| 55 | 10,000 | Block | LQHPT | 0.5 | 2736.81 | 0.99 | 0.96 | 0.00 | 0.02 | 0.00 | 0.29 | 0.04 | 0.14 | 0.06 |
| 55 | 10,000 | Block | H | 1.8 | 2844.43 | 0.97 | 0.96 | 0.00 | 0.01 | 0.00 | 0.13 | 0.06 | 0.04 | 0.01 |
| 55 | 10,000 | Block | H | 1.9 | 5816.50 | 0.97 | 0.96 | 0.00 | 0.01 | 0.00 | 0.13 | 0.06 | 0.04 | 0.01 |
| 55 | 10,000 | Block | H | 0.1 | NA | 0.99 | 0.96 | 0.00 | 0.00 | 0.00 | 0.22 | 0.01 | 0.04 | 0.01 |
| 55 | 10,000 | Block | LQH | 0.1 | NA | 0.99 | 0.96 | 0.00 | 0.00 | 0.00 | 0.22 | 0.01 | 0.04 | 0.01 |
| 55 | 10,000 | Block | LQHP | 0.1 | NA | 0.99 | 0.96 | 0.00 | 0.00 | 0.00 | 0.22 | 0.01 | 0.04 | 0.01 |
| 55 | 10,000 | Block | LQHPT | 0.1 | NA | 0.99 | 0.88 | 0.05 | 0.09 | 0.06 | 0.54 | 0.03 | 0.25 | 0.06 |
| 55 | 10,000 | Block | H | 0.2 | NA | 0.99 | 0.97 | 0.00 | 0.00 | 0.00 | 0.16 | 0.02 | 0.02 | 0.00 |
| 55 | 10,000 | Block | LQH | 0.2 | NA | 0.99 | 0.97 | 0.00 | 0.00 | 0.00 | 0.14 | 0.02 | 0.02 | 0.00 |
| 55 | 10,000 | Block | LQHP | 0.2 | NA | 0.99 | 0.97 | 0.00 | 0.00 | 0.00 | 0.16 | 0.02 | 0.02 | 0.00 |
| 55 | 10,000 | Block | LQHPT | 0.2 | NA | 0.99 | 0.88 | 0.05 | 0.09 | 0.06 | 0.50 | 0.06 | 0.23 | 0.07 |
| 55 | 10,000 | Block | H | 0.3 | NA | 0.99 | 0.97 | 0.00 | 0.01 | 0.00 | 0.20 | 0.03 | 0.04 | 0.01 |
| 55 | 10,000 | Block | LQH | 0.3 | NA | 0.99 | 0.97 | 0.00 | 0.01 | 0.00 | 0.16 | 0.02 | 0.04 | 0.01 |
| 55 | 10,000 | Block | LQHP | 0.3 | NA | 0.99 | 0.97 | 0.00 | 0.01 | 0.00 | 0.18 | 0.03 | 0.04 | 0.01 |
| 55 | 10,000 | Block | LQHPT | 0.3 | NA | 0.99 | 0.89 | 0.06 | 0.09 | 0.06 | 0.41 | 0.02 | 0.20 | 0.06 |
| 55 | 10,000 | Block | H | 0.4 | NA | 0.98 | 0.97 | 0.00 | 0.01 | 0.00 | 0.18 | 0.03 | 0.05 | 0.01 |
| 55 | 10,000 | Block | LQH | 0.4 | NA | 0.98 | 0.97 | 0.00 | 0.01 | 0.00 | 0.18 | 0.03 | 0.05 | 0.01 |
| 55 | 10,000 | Block | LQHP | 0.4 | NA | 0.98 | 0.97 | 0.00 | 0.01 | 0.00 | 0.22 | 0.04 | 0.04 | 0.01 |
| 55 | 10,000 | Block | LQHPT | 0.4 | NA | 0.99 | 0.94 | 0.01 | 0.04 | 0.01 | 0.36 | 0.03 | 0.14 | 0.06 |
| 55 | 10,000 | Block | H | 0.5 | NA | 0.98 | 0.97 | 0.00 | 0.01 | 0.00 | 0.18 | 0.03 | 0.05 | 0.01 |
| 55 | 10,000 | Block | LQH | 0.5 | NA | 0.98 | 0.97 | 0.00 | 0.01 | 0.00 | 0.23 | 0.06 | 0.09 | 0.03 |
| 55 | 10,000 | Block | LQHP | 0.5 | NA | 0.98 | 0.97 | 0.00 | 0.01 | 0.00 | 0.23 | 0.06 | 0.05 | 0.01 |
| 55 | 10,000 | Block | H | 0.6 | NA | 0.98 | 0.97 | 0.00 | 0.01 | 0.00 | 0.18 | 0.03 | 0.05 | 0.01 |
| 55 | 10,000 | Block | H | 0.7 | NA | 0.98 | 0.97 | 0.00 | 0.01 | 0.00 | 0.18 | 0.03 | 0.05 | 0.01 |
| 55 | 10,000 | Block | H | 0.8 | NA | 0.98 | 0.97 | 0.00 | 0.01 | 0.00 | 0.14 | 0.02 | 0.05 | 0.01 |
| 55 | 10,000 | Block | H | 0.9 | NA | 0.98 | 0.97 | 0.00 | 0.01 | 0.00 | 0.14 | 0.02 | 0.05 | 0.01 |
| 55 | 10,000 | Block | H | 1 | NA | 0.97 | 0.97 | 0.00 | 0.01 | 0.00 | 0.13 | 0.04 | 0.05 | 0.01 |
| 55 | 10,000 | Block | H | 1.1 | NA | 0.97 | 0.97 | 0.00 | 0.01 | 0.00 | 0.14 | 0.06 | 0.04 | 0.01 |
| 55 | 10,000 | Block | H | 1.6 | NA | 0.97 | 0.96 | 0.00 | 0.01 | 0.00 | 0.13 | 0.06 | 0.04 | 0.01 |
| 55 | 10,000 | Checkerboar1 | LQHPT | 1.4 | 0.00 | 0.98 | 0.97 | 0.00 | 0.01 | 0.00 | 0.14 | 0.00 | 0.05 | 0.00 |
| 55 | 10,000 | Checkerboar1 | LQ | 1 | 4.63 | 0.97 | 0.96 | 0.00 | 0.00 | 0.00 | 0.09 | 0.00 | 0.05 | 0.01 |
| 55 | 10,000 | Checkerboar1 | LQ | 0.6 | 5.17 | 0.97 | 0.97 | 0.00 | 0.00 | 0.00 | 0.11 | 0.00 | 0.05 | 0.01 |
| 55 | 10,000 | Checkerboar1 | LQ | 0.7 | 5.98 | 0.97 | 0.96 | 0.00 | 0.00 | 0.00 | 0.09 | 0.00 | 0.05 | 0.01 |
| 55 | 10,000 | Checkerboar1 | LQ | 1.1 | 6.20 | 0.97 | 0.96 | 0.00 | 0.00 | 0.00 | 0.09 | 0.00 | 0.05 | 0.01 |
| 55 | 10,000 | Checkerboar1 | LQ | 0.9 | 7.18 | 0.97 | 0.96 | 0.00 | 0.00 | 0.00 | 0.09 | 0.00 | 0.05 | 0.01 |
| 55 | 10,000 | Checkerboar1 | LQ | 1.2 | 7.76 | 0.97 | 0.96 | 0.00 | 0.00 | 0.00 | 0.09 | 0.00 | 0.03 | 0.00 |
| 55 | 10,000 | Checkerboar1 | LQ | 0.5 | 7.96 | 0.97 | 0.97 | 0.00 | 0.00 | 0.00 | 0.11 | 0.00 | 0.05 | 0.01 |
| 55 | 10,000 | Checkerboar1 | LQ | 0.1 | 9.38 | 0.97 | 0.97 | 0.00 | 0.01 | 0.00 | 0.11 | 0.00 | 0.07 | 0.00 |
| 55 | 10,000 | Checkerboar1 | LQ | 0.2 | 9.38 | 0.97 | 0.97 | 0.00 | 0.01 | 0.00 | 0.12 | 0.00 | 0.07 | 0.00 |
| 55 | 10,000 | Checkerboar1 | LQ | 0.3 | 9.38 | 0.97 | 0.97 | 0.00 | 0.01 | 0.00 | 0.11 | 0.00 | 0.09 | 0.00 |
| 55 | 10,000 | Checkerboar1 | LQ | 1.3 | 9.74 | 0.97 | 0.96 | 0.00 | 0.00 | 0.00 | 0.09 | 0.00 | 0.03 | 0.00 |
| 55 | 10,000 | Checkerboar1 | LQ | 0.4 | 10.48 | 0.97 | 0.97 | 0.00 | 0.01 | 0.00 | 0.11 | 0.00 | 0.07 | 0.00 |
| 55 | 10,000 | Checkerboar1 | LQ | 1.4 | 11.65 | 0.97 | 0.96 | 0.00 | 0.00 | 0.00 | 0.09 | 0.00 | 0.03 | 0.00 |
| 55 | 10,000 | Checkerboar1 | LQ | 0.8 | 12.71 | 0.97 | 0.96 | 0.00 | 0.00 | 0.00 | 0.09 | 0.00 | 0.05 | 0.01 |
| 55 | 10,000 | Checkerboar1 | LQ | 1.5 | 13.40 | 0.97 | 0.96 | 0.00 | 0.00 | 0.00 | 0.09 | 0.00 | 0.03 | 0.00 |
| 55 | 10,000 | Checkerboar1 | LQ | 1.6 | 15.67 | 0.97 | 0.96 | 0.00 | 0.00 | 0.00 | 0.09 | 0.00 | 0.03 | 0.00 |
| 55 | 10,000 | Checkerboar1 | LQ | 1.7 | 17.85 | 0.97 | 0.96 | 0.00 | 0.00 | 0.00 | 0.09 | 0.00 | 0.03 | 0.00 |
| 55 | 10,000 | Checkerboar1 | LQHPT | 1.5 | 18.22 | 0.98 | 0.97 | 0.00 | 0.01 | 0.00 | 0.14 | 0.00 | 0.05 | 0.00 |
| 55 | 10,000 | Checkerboar1 | LQ | 1.8 | 19.38 | 0.96 | 0.96 | 0.00 | 0.00 | 0.00 | 0.09 | 0.00 | 0.03 | 0.00 |
| 55 | 10,000 | Checkerboar1 | LQHPT | 1.1 | 21.13 | 0.98 | 0.97 | 0.00 | 0.01 | 0.00 | 0.18 | 0.00 | 0.13 | 0.00 |
| 55 | 10,000 | Checkerboar1 | LQ | 1.9 | 21.68 | 0.96 | 0.96 | 0.00 | 0.00 | 0.00 | 0.09 | 0.00 | 0.03 | 0.00 |
| 55 | 10,000 | Checkerboar1 | LQ | 2 | 23.67 | 0.96 | 0.96 | 0.00 | 0.00 | 0.00 | 0.09 | 0.00 | 0.03 | 0.00 |
| 55 | 10,000 | Checkerboar1 | LQHPT | 1.3 | 24.33 | 0.98 | 0.97 | 0.00 | 0.01 | 0.00 | 0.14 | 0.00 | 0.05 | 0.00 |
| 55 | 10,000 | Checkerboar1 | LQHP | 1.3 | 24.33 | 0.97 | 0.97 | 0.00 | 0.00 | 0.00 | 0.13 | 0.00 | 0.07 | 0.00 |
| 55 | 10,000 | Checkerboar1 | LQHP | 1.2 | 32.23 | 0.97 | 0.97 | 0.00 | 0.00 | 0.00 | 0.13 | 0.00 | 0.09 | 0.00 |
| 55 | 10,000 | Checkerboar1 | LQH | 1.6 | 33.46 | 0.97 | 0.96 | 0.00 | 0.00 | 0.00 | 0.14 | 0.00 | 0.04 | 0.00 |
| 55 | 10,000 | Checkerboar1 | LQHP | 1.1 | 33.91 | 0.97 | 0.97 | 0.00 | 0.00 | 0.00 | 0.13 | 0.00 | 0.09 | 0.00 |
| 55 | 10,000 | Checkerboar1 | LQHP | 1 | 37.32 | 0.98 | 0.97 | 0.00 | 0.00 | 0.00 | 0.13 | 0.00 | 0.07 | 0.00 |
| 55 | 10,000 | Checkerboar1 | LQHP | 1.5 | 37.93 | 0.97 | 0.97 | 0.00 | 0.00 | 0.00 | 0.11 | 0.00 | 0.05 | 0.00 |
| 55 | 10,000 | Checkerboar1 | LQHPT | 1.8 | 38.03 | 0.98 | 0.96 | 0.00 | 0.01 | 0.00 | 0.14 | 0.00 | 0.03 | 0.00 |
| 55 | 10,000 | Checkerboar1 | LQHP | 2 | 38.33 | 0.97 | 0.96 | 0.00 | 0.00 | 0.00 | 0.09 | 0.00 | 0.05 | 0.00 |
| 55 | 10,000 | Checkerboar1 | LQHPT | 1.6 | 39.98 | 0.98 | 0.97 | 0.00 | 0.01 | 0.00 | 0.14 | 0.00 | 0.05 | 0.00 |
| 55 | 10,000 | Checkerboar1 | LQHPT | 1.2 | 40.75 | 0.98 | 0.97 | 0.00 | 0.01 | 0.00 | 0.18 | 0.00 | 0.07 | 0.00 |
| 55 | 10,000 | Checkerboar1 | LQH | 1.4 | 40.84 | 0.97 | 0.97 | 0.00 | 0.00 | 0.00 | 0.13 | 0.00 | 0.04 | 0.00 |
| 55 | 10,000 | Checkerboar1 | LQHP | 1.9 | 40.89 | 0.97 | 0.96 | 0.00 | 0.00 | 0.00 | 0.09 | 0.00 | 0.05 | 0.00 |
| 55 | 10,000 | Checkerboar1 | LQHPT | 1.7 | 42.44 | 0.98 | 0.97 | 0.00 | 0.01 | 0.00 | 0.14 | 0.00 | 0.05 | 0.00 |
| 55 | 10,000 | Checkerboar1 | LQH | 1.7 | 45.25 | 0.97 | 0.96 | 0.00 | 0.00 | 0.00 | 0.14 | 0.00 | 0.04 | 0.00 |
| 55 | 10,000 | Checkerboar1 | LQHP | 1.6 | 46.37 | 0.97 | 0.96 | 0.00 | 0.00 | 0.00 | 0.11 | 0.00 | 0.05 | 0.00 |
| 55 | 10,000 | Checkerboar1 | LQH | 1.5 | 46.63 | 0.97 | 0.97 | 0.00 | 0.00 | 0.00 | 0.14 | 0.00 | 0.04 | 0.00 |
| 55 | 10,000 | Checkerboar1 | LQHPT | 1.9 | 46.68 | 0.97 | 0.96 | 0.00 | 0.01 | 0.00 | 0.14 | 0.00 | 0.03 | 0.00 |
| 55 | 10,000 | Checkerboar1 | LQH | 1.8 | 46.92 | 0.97 | 0.96 | 0.00 | 0.00 | 0.00 | 0.13 | 0.00 | 0.04 | 0.00 |
| 55 | 10,000 | Checkerboar1 | LQHP | 1.4 | 48.18 | 0.97 | 0.97 | 0.00 | 0.00 | 0.00 | 0.13 | 0.00 | 0.07 | 0.00 |
| 55 | 10,000 | Checkerboar1 | LQHPT | 1 | 52.41 | 0.98 | 0.97 | 0.00 | 0.01 | 0.00 | 0.18 | 0.00 | 0.14 | 0.00 |
| 55 | 10,000 | Checkerboar1 | LQHPT | 2 | 55.86 | 0.97 | 0.96 | 0.00 | 0.01 | 0.00 | 0.14 | 0.00 | 0.03 | 0.00 |
| 55 | 10,000 | Checkerboar1 | LQHP | 1.8 | 56.03 | 0.97 | 0.96 | 0.00 | 0.00 | 0.00 | 0.09 | 0.00 | 0.05 | 0.00 |
| 55 | 10,000 | Checkerboar1 | LQH | 2 | 57.46 | 0.97 | 0.96 | 0.00 | 0.00 | 0.00 | 0.09 | 0.00 | 0.04 | 0.00 |
| 55 | 10,000 | Checkerboar1 | LQHP | 1.7 | 67.72 | 0.97 | 0.96 | 0.00 | 0.00 | 0.00 | 0.09 | 0.00 | 0.05 | 0.00 |
| 55 | 10,000 | Checkerboar1 | LQH | 1.9 | 67.89 | 0.97 | 0.96 | 0.00 | 0.00 | 0.00 | 0.11 | 0.00 | 0.04 | 0.00 |
| 55 | 10,000 | Checkerboar1 | LQHPT | 0.9 | 78.07 | 0.98 | 0.97 | 0.00 | 0.01 | 0.00 | 0.18 | 0.00 | 0.14 | 0.00 |
| 55 | 10,000 | Checkerboar1 | LQH | 1.3 | 86.28 | 0.97 | 0.97 | 0.00 | 0.00 | 0.00 | 0.13 | 0.00 | 0.04 | 0.00 |
| 55 | 10,000 | Checkerboar1 | LQH | 1.2 | 93.10 | 0.97 | 0.97 | 0.00 | 0.00 | 0.00 | 0.13 | 0.00 | 0.04 | 0.00 |
| 55 | 10,000 | Checkerboar1 | LQHP | 0.8 | 104.22 | 0.98 | 0.97 | 0.00 | 0.00 | 0.00 | 0.11 | 0.00 | 0.05 | 0.00 |
| 55 | 10,000 | Checkerboar1 | LQHP | 0.7 | 110.50 | 0.98 | 0.97 | 0.00 | 0.00 | 0.00 | 0.11 | 0.00 | 0.07 | 0.00 |
| 55 | 10,000 | Checkerboar1 | LQHP | 0.9 | 123.27 | 0.98 | 0.97 | 0.00 | 0.00 | 0.00 | 0.11 | 0.00 | 0.07 | 0.00 |
| 55 | 10,000 | Checkerboar1 | L | 0.6 | 144.74 | 0.90 | 0.90 | 0.00 | 0.01 | 0.00 | 0.07 | 0.00 | 0.02 | 0.00 |
| 55 | 10,000 | Checkerboar1 | L | 0.1 | 144.79 | 0.90 | 0.90 | 0.00 | 0.01 | 0.00 | 0.11 | 0.01 | 0.03 | 0.00 |
| 55 | 10,000 | Checkerboar1 | L | 0.2 | 144.79 | 0.90 | 0.90 | 0.00 | 0.01 | 0.00 | 0.11 | 0.01 | 0.03 | 0.00 |
| 55 | 10,000 | Checkerboar1 | L | 0.3 | 144.79 | 0.90 | 0.90 | 0.00 | 0.01 | 0.00 | 0.11 | 0.01 | 0.03 | 0.00 |
| 55 | 10,000 | Checkerboar1 | L | 0.4 | 144.79 | 0.90 | 0.90 | 0.00 | 0.01 | 0.00 | 0.11 | 0.01 | 0.02 | 0.00 |
| 55 | 10,000 | Checkerboar1 | L | 0.5 | 144.79 | 0.90 | 0.90 | 0.00 | 0.01 | 0.00 | 0.09 | 0.00 | 0.02 | 0.00 |
| 55 | 10,000 | Checkerboar1 | L | 0.7 | 144.89 | 0.90 | 0.90 | 0.00 | 0.01 | 0.00 | 0.09 | 0.00 | 0.02 | 0.00 |
| 55 | 10,000 | Checkerboar1 | L | 0.8 | 145.27 | 0.90 | 0.90 | 0.00 | 0.01 | 0.00 | 0.07 | 0.00 | 0.04 | 0.00 |
| 55 | 10,000 | Checkerboar1 | L | 0.9 | 145.53 | 0.90 | 0.89 | 0.00 | 0.01 | 0.00 | 0.07 | 0.00 | 0.04 | 0.00 |
| 55 | 10,000 | Checkerboar1 | L | 1 | 145.82 | 0.90 | 0.89 | 0.00 | 0.01 | 0.00 | 0.07 | 0.00 | 0.04 | 0.00 |
| 55 | 10,000 | Checkerboar1 | L | 1.1 | 146.32 | 0.90 | 0.89 | 0.00 | 0.01 | 0.00 | 0.07 | 0.00 | 0.04 | 0.00 |
| 55 | 10,000 | Checkerboar1 | L | 1.2 | 147.16 | 0.90 | 0.89 | 0.00 | 0.01 | 0.00 | 0.11 | 0.00 | 0.04 | 0.00 |
| 55 | 10,000 | Checkerboar1 | L | 1.7 | 147.47 | 0.90 | 0.88 | 0.00 | 0.01 | 0.00 | 0.11 | 0.00 | 0.02 | 0.00 |
| 55 | 10,000 | Checkerboar1 | L | 1.5 | 147.81 | 0.90 | 0.88 | 0.00 | 0.01 | 0.00 | 0.11 | 0.00 | 0.02 | 0.00 |
| 55 | 10,000 | Checkerboar1 | L | 1.3 | 148.24 | 0.90 | 0.88 | 0.00 | 0.01 | 0.00 | 0.11 | 0.00 | 0.04 | 0.00 |
| 55 | 10,000 | Checkerboar1 | L | 1.8 | 148.38 | 0.90 | 0.88 | 0.00 | 0.01 | 0.00 | 0.11 | 0.00 | 0.02 | 0.00 |
| 55 | 10,000 | Checkerboar1 | L | 1.6 | 148.92 | 0.90 | 0.88 | 0.00 | 0.01 | 0.00 | 0.11 | 0.00 | 0.02 | 0.00 |
| 55 | 10,000 | Checkerboar1 | L | 1.4 | 149.34 | 0.90 | 0.88 | 0.00 | 0.01 | 0.00 | 0.11 | 0.00 | 0.04 | 0.00 |
| 55 | 10,000 | Checkerboar1 | L | 1.9 | 149.35 | 0.90 | 0.88 | 0.00 | 0.01 | 0.00 | 0.09 | 0.00 | 0.02 | 0.00 |
| 55 | 10,000 | Checkerboar1 | L | 2 | 150.07 | 0.90 | 0.87 | 0.00 | 0.01 | 0.00 | 0.09 | 0.00 | 0.02 | 0.00 |
| 55 | 10,000 | Checkerboar1 | LQH | 1.1 | 163.90 | 0.97 | 0.97 | 0.00 | 0.00 | 0.00 | 0.13 | 0.00 | 0.05 | 0.00 |
| 55 | 10,000 | Checkerboar1 | LQH | 1 | 217.07 | 0.98 | 0.97 | 0.00 | 0.00 | 0.00 | 0.13 | 0.00 | 0.05 | 0.00 |
| 55 | 10,000 | Checkerboar1 | LQH | 0.7 | 222.92 | 0.98 | 0.97 | 0.00 | 0.00 | 0.00 | 0.11 | 0.00 | 0.07 | 0.00 |
| 55 | 10,000 | Checkerboar1 | LQHPT | 0.7 | 246.55 | 0.99 | 0.97 | 0.00 | 0.01 | 0.00 | 0.26 | 0.01 | 0.11 | 0.00 |
| 55 | 10,000 | Checkerboar1 | LQHPT | 0.8 | 293.67 | 0.98 | 0.97 | 0.00 | 0.01 | 0.00 | 0.18 | 0.00 | 0.13 | 0.00 |
| 55 | 10,000 | Checkerboar1 | LQH | 0.8 | 328.56 | 0.98 | 0.97 | 0.00 | 0.00 | 0.00 | 0.11 | 0.00 | 0.07 | 0.00 |
| 55 | 10,000 | Checkerboar1 | H | 1.4 | 362.06 | 0.97 | 0.96 | 0.00 | 0.00 | 0.00 | 0.07 | 0.00 | 0.03 | 0.00 |
| 55 | 10,000 | Checkerboar1 | H | 2 | 472.34 | 0.97 | 0.96 | 0.00 | 0.00 | 0.00 | 0.09 | 0.00 | 0.03 | 0.00 |
| 55 | 10,000 | Checkerboar1 | LQH | 0.9 | 582.77 | 0.98 | 0.97 | 0.00 | 0.00 | 0.00 | 0.11 | 0.00 | 0.09 | 0.00 |
| 55 | 10,000 | Checkerboar1 | H | 1.3 | 606.40 | 0.97 | 0.96 | 0.00 | 0.00 | 0.00 | 0.07 | 0.00 | 0.03 | 0.00 |
| 55 | 10,000 | Checkerboar1 | LQHP | 0.6 | 668.11 | 0.98 | 0.97 | 0.00 | 0.00 | 0.00 | 0.09 | 0.00 | 0.07 | 0.00 |
| 55 | 10,000 | Checkerboar1 | H | 1.7 | 721.23 | 0.97 | 0.96 | 0.00 | 0.00 | 0.00 | 0.09 | 0.00 | 0.03 | 0.00 |
| 55 | 10,000 | Checkerboar1 | LQHPT | 0.6 | 768.44 | 0.99 | 0.97 | 0.00 | 0.02 | 0.00 | 0.22 | 0.00 | 0.12 | 0.00 |
| 55 | 10,000 | Checkerboar1 | LQH | 0.6 | 1304.64 | 0.98 | 0.97 | 0.00 | 0.00 | 0.00 | 0.11 | 0.00 | 0.05 | 0.00 |
| 55 | 10,000 | Checkerboar1 | H | 1.5 | 1354.10 | 0.97 | 0.96 | 0.00 | 0.00 | 0.00 | 0.09 | 0.00 | 0.03 | 0.00 |
| 55 | 10,000 | Checkerboar1 | H | 1.2 | 1838.75 | 0.97 | 0.96 | 0.00 | 0.00 | 0.00 | 0.07 | 0.00 | 0.03 | 0.00 |
| 55 | 10,000 | Checkerboar1 | LQHPT | 0.5 | 2736.81 | 0.99 | 0.97 | 0.00 | 0.02 | 0.00 | 0.25 | 0.00 | 0.18 | 0.00 |
| 55 | 10,000 | Checkerboar1 | H | 1.8 | 2844.43 | 0.97 | 0.96 | 0.00 | 0.00 | 0.00 | 0.09 | 0.00 | 0.03 | 0.00 |
| 55 | 10,000 | Checkerboar1 | H | 1.9 | 5816.50 | 0.97 | 0.96 | 0.00 | 0.00 | 0.00 | 0.09 | 0.00 | 0.03 | 0.00 |
| 55 | 10,000 | Checkerboar1 | H | 0.1 | NA | 0.99 | 0.98 | 0.00 | 0.01 | 0.00 | 0.33 | 0.00 | 0.19 | 0.05 |
| 55 | 10,000 | Checkerboar1 | LQH | 0.1 | NA | 0.99 | 0.98 | 0.00 | 0.01 | 0.00 | 0.33 | 0.00 | 0.19 | 0.05 |
| 55 | 10,000 | Checkerboar1 | LQHP | 0.1 | NA | 0.99 | 0.98 | 0.00 | 0.01 | 0.00 | 0.33 | 0.00 | 0.19 | 0.05 |
| 55 | 10,000 | Checkerboar1 | LQHPT | 0.1 | NA | 0.99 | 0.95 | 0.00 | 0.05 | 0.00 | 0.64 | 0.00 | 0.35 | 0.00 |
| 55 | 10,000 | Checkerboar1 | H | 0.2 | NA | 0.99 | 0.98 | 0.00 | 0.01 | 0.00 | 0.25 | 0.00 | 0.05 | 0.00 |
| 55 | 10,000 | Checkerboar1 | LQH | 0.2 | NA | 0.99 | 0.98 | 0.00 | 0.01 | 0.00 | 0.22 | 0.00 | 0.05 | 0.00 |
| 55 | 10,000 | Checkerboar1 | LQHP | 0.2 | NA | 0.99 | 0.98 | 0.00 | 0.01 | 0.00 | 0.22 | 0.00 | 0.05 | 0.00 |
| 55 | 10,000 | Checkerboar1 | LQHPT | 0.2 | NA | 0.99 | 0.95 | 0.00 | 0.04 | 0.00 | 0.50 | 0.01 | 0.41 | 0.04 |
| 55 | 10,000 | Checkerboar1 | H | 0.3 | NA | 0.99 | 0.98 | 0.00 | 0.01 | 0.00 | 0.20 | 0.00 | 0.04 | 0.00 |
| 55 | 10,000 | Checkerboar1 | LQH | 0.3 | NA | 0.99 | 0.98 | 0.00 | 0.01 | 0.00 | 0.20 | 0.00 | 0.05 | 0.00 |
| 55 | 10,000 | Checkerboar1 | LQHP | 0.3 | NA | 0.99 | 0.98 | 0.00 | 0.01 | 0.00 | 0.22 | 0.00 | 0.05 | 0.00 |
| 55 | 10,000 | Checkerboar1 | LQHPT | 0.3 | NA | 0.99 | 0.96 | 0.00 | 0.03 | 0.00 | 0.40 | 0.02 | 0.22 | 0.00 |
| 55 | 10,000 | Checkerboar1 | H | 0.4 | NA | 0.98 | 0.97 | 0.00 | 0.01 | 0.00 | 0.14 | 0.00 | 0.05 | 0.00 |
| 55 | 10,000 | Checkerboar1 | LQH | 0.4 | NA | 0.98 | 0.97 | 0.00 | 0.01 | 0.00 | 0.15 | 0.00 | 0.05 | 0.00 |
| 55 | 10,000 | Checkerboar1 | LQHP | 0.4 | NA | 0.98 | 0.98 | 0.00 | 0.01 | 0.00 | 0.14 | 0.00 | 0.07 | 0.00 |
| 55 | 10,000 | Checkerboar1 | LQHPT | 0.4 | NA | 0.99 | 0.97 | 0.00 | 0.02 | 0.00 | 0.27 | 0.00 | 0.20 | 0.00 |
| 55 | 10,000 | Checkerboar1 | H | 0.5 | NA | 0.98 | 0.97 | 0.00 | 0.01 | 0.00 | 0.11 | 0.00 | 0.05 | 0.00 |
| 55 | 10,000 | Checkerboar1 | LQH | 0.5 | NA | 0.98 | 0.97 | 0.00 | 0.01 | 0.00 | 0.11 | 0.00 | 0.05 | 0.00 |
| 55 | 10,000 | Checkerboar1 | LQHP | 0.5 | NA | 0.98 | 0.97 | 0.00 | 0.01 | 0.00 | 0.11 | 0.00 | 0.09 | 0.00 |
| 55 | 10,000 | Checkerboar1 | H | 0.6 | NA | 0.98 | 0.97 | 0.00 | 0.01 | 0.00 | 0.11 | 0.00 | 0.07 | 0.00 |
| 55 | 10,000 | Checkerboar1 | H | 0.7 | NA | 0.98 | 0.97 | 0.00 | 0.00 | 0.00 | 0.09 | 0.00 | 0.05 | 0.00 |
| 55 | 10,000 | Checkerboar1 | H | 0.8 | NA | 0.98 | 0.97 | 0.00 | 0.00 | 0.00 | 0.13 | 0.00 | 0.05 | 0.00 |
| 55 | 10,000 | Checkerboar1 | H | 0.9 | NA | 0.98 | 0.97 | 0.00 | 0.00 | 0.00 | 0.13 | 0.00 | 0.05 | 0.00 |
| 55 | 10,000 | Checkerboar1 | H | 1 | NA | 0.97 | 0.97 | 0.00 | 0.00 | 0.00 | 0.11 | 0.00 | 0.05 | 0.00 |
| 55 | 10,000 | Checkerboar1 | H | 1.1 | NA | 0.97 | 0.96 | 0.00 | 0.00 | 0.00 | 0.09 | 0.00 | 0.05 | 0.00 |
| 55 | 10,000 | Checkerboar1 | H | 1.6 | NA | 0.97 | 0.96 | 0.00 | 0.00 | 0.00 | 0.09 | 0.00 | 0.03 | 0.00 |

***Calibration area No 2: Starry stonewort entire species range considering maximum dispersal potential (~2,200 km, M_g_)***

| ***n*** | **BCK** | **Partition** | **FC** | **RM** | **∆AICc** | **AUC test** | **AUC mean** | **AUC varianza** | **AUC mean difference** | **AUC var difference** | **OR mean 10%** | **OR var 10%** | **OR mean MTP** | **OR var MTP** |
| --- | --- | --- | --- | --- | --- | --- | --- | --- | --- | --- | --- | --- | --- | --- |
| 84 | 5,000 | Jakknife | LQHPT | 2 | 0.00 | 0.98 | 0.96 | 0.26 | 0.02 | 0.21 | 0.18 | 0.15 | 0.04 | 0.04 |
| 84 | 5,000 | Jakknife | LQ | 0.6 | 3.20 | 0.97 | 0.96 | 0.30 | 0.02 | 0.24 | 0.12 | 0.11 | 0.01 | 0.01 |
| 84 | 5,000 | Jakknife | LQ | 0.8 | 6.39 | 0.97 | 0.96 | 0.37 | 0.02 | 0.31 | 0.12 | 0.11 | 0.01 | 0.01 |
| 84 | 5,000 | Jakknife | LQHPT | 1.9 | 6.48 | 0.98 | 0.96 | 0.26 | 0.02 | 0.21 | 0.17 | 0.14 | 0.04 | 0.04 |
| 84 | 5,000 | Jakknife | LQ | 0.1 | 8.12 | 0.97 | 0.96 | 0.29 | 0.02 | 0.24 | 0.12 | 0.11 | 0.01 | 0.01 |
| 84 | 5,000 | Jakknife | LQ | 0.2 | 8.12 | 0.97 | 0.96 | 0.29 | 0.02 | 0.24 | 0.12 | 0.11 | 0.01 | 0.01 |
| 84 | 5,000 | Jakknife | LQ | 0.3 | 8.12 | 0.97 | 0.96 | 0.29 | 0.02 | 0.24 | 0.12 | 0.11 | 0.01 | 0.01 |
| 84 | 5,000 | Jakknife | LQ | 0.4 | 8.12 | 0.97 | 0.96 | 0.29 | 0.02 | 0.24 | 0.12 | 0.11 | 0.01 | 0.01 |
| 84 | 5,000 | Jakknife | LQ | 0.5 | 8.12 | 0.97 | 0.96 | 0.30 | 0.02 | 0.25 | 0.12 | 0.11 | 0.01 | 0.01 |
| 84 | 5,000 | Jakknife | LQHPT | 1.6 | 9.14 | 0.98 | 0.97 | 0.26 | 0.02 | 0.21 | 0.17 | 0.14 | 0.04 | 0.04 |
| 84 | 5,000 | Jakknife | LQ | 0.7 | 10.32 | 0.97 | 0.96 | 0.29 | 0.02 | 0.23 | 0.12 | 0.11 | 0.01 | 0.01 |
| 84 | 5,000 | Jakknife | LQ | 1.1 | 14.68 | 0.97 | 0.96 | 0.32 | 0.02 | 0.26 | 0.12 | 0.11 | 0.01 | 0.01 |
| 84 | 5,000 | Jakknife | LQ | 1.2 | 15.01 | 0.97 | 0.96 | 0.32 | 0.02 | 0.27 | 0.12 | 0.11 | 0.01 | 0.01 |
| 84 | 5,000 | Jakknife | LQHPT | 1.7 | 17.04 | 0.98 | 0.96 | 0.26 | 0.02 | 0.21 | 0.18 | 0.15 | 0.04 | 0.04 |
| 84 | 5,000 | Jakknife | LQ | 0.9 | 17.96 | 0.97 | 0.96 | 0.34 | 0.02 | 0.28 | 0.11 | 0.10 | 0.01 | 0.01 |
| 84 | 5,000 | Jakknife | LQ | 1.6 | 17.98 | 0.97 | 0.96 | 0.30 | 0.02 | 0.24 | 0.13 | 0.12 | 0.01 | 0.01 |
| 84 | 5,000 | Jakknife | LQ | 1 | 18.95 | 0.97 | 0.96 | 0.35 | 0.02 | 0.29 | 0.12 | 0.11 | 0.01 | 0.01 |
| 84 | 5,000 | Jakknife | LQ | 1.5 | 21.47 | 0.97 | 0.96 | 0.35 | 0.02 | 0.28 | 0.17 | 0.14 | 0.01 | 0.01 |
| 84 | 5,000 | Jakknife | LQHPT | 1.5 | 22.80 | 0.98 | 0.96 | 0.26 | 0.02 | 0.22 | 0.17 | 0.14 | 0.04 | 0.04 |
| 84 | 5,000 | Jakknife | LQ | 1.3 | 23.89 | 0.97 | 0.96 | 0.38 | 0.02 | 0.31 | 0.13 | 0.12 | 0.01 | 0.01 |
| 84 | 5,000 | Jakknife | LQ | 1.4 | 25.56 | 0.97 | 0.96 | 0.34 | 0.02 | 0.28 | 0.13 | 0.12 | 0.01 | 0.01 |
| 84 | 5,000 | Jakknife | LQ | 1.8 | 27.07 | 0.97 | 0.96 | 0.31 | 0.02 | 0.24 | 0.16 | 0.13 | 0.01 | 0.01 |
| 84 | 5,000 | Jakknife | LQ | 1.9 | 29.36 | 0.97 | 0.96 | 0.30 | 0.02 | 0.23 | 0.17 | 0.14 | 0.01 | 0.01 |
| 84 | 5,000 | Jakknife | LQ | 1.7 | 30.76 | 0.97 | 0.96 | 0.32 | 0.02 | 0.25 | 0.17 | 0.14 | 0.01 | 0.01 |
| 84 | 5,000 | Jakknife | LQHPT | 1.8 | 32.96 | 0.98 | 0.97 | 0.25 | 0.02 | 0.20 | 0.17 | 0.14 | 0.04 | 0.04 |
| 84 | 5,000 | Jakknife | LQ | 2 | 33.79 | 0.97 | 0.96 | 0.29 | 0.02 | 0.22 | 0.13 | 0.12 | 0.01 | 0.01 |
| 84 | 5,000 | Jakknife | LQHPT | 1.4 | 82.28 | 0.98 | 0.96 | 0.28 | 0.03 | 0.23 | 0.17 | 0.14 | 0.04 | 0.04 |
| 84 | 5,000 | Jakknife | LQHP | 1.6 | 95.53 | 0.97 | 0.96 | 0.24 | 0.02 | 0.19 | 0.16 | 0.13 | 0.02 | 0.02 |
| 84 | 5,000 | Jakknife | LQH | 1.6 | 98.50 | 0.97 | 0.96 | 0.35 | 0.02 | 0.27 | 0.12 | 0.11 | 0.04 | 0.04 |
| 84 | 5,000 | Jakknife | LQH | 1.5 | 102.89 | 0.97 | 0.96 | 0.36 | 0.02 | 0.29 | 0.12 | 0.11 | 0.04 | 0.04 |
| 84 | 5,000 | Jakknife | LQHPT | 1.2 | 103.16 | 0.98 | 0.96 | 0.29 | 0.03 | 0.25 | 0.18 | 0.15 | 0.05 | 0.05 |
| 84 | 5,000 | Jakknife | LQHP | 1.4 | 103.74 | 0.97 | 0.97 | 0.23 | 0.02 | 0.18 | 0.14 | 0.13 | 0.01 | 0.01 |
| 84 | 5,000 | Jakknife | LQHP | 1.5 | 114.87 | 0.97 | 0.97 | 0.24 | 0.02 | 0.19 | 0.14 | 0.13 | 0.02 | 0.02 |
| 84 | 5,000 | Jakknife | LQHP | 1.7 | 122.08 | 0.97 | 0.97 | 0.24 | 0.02 | 0.18 | 0.16 | 0.13 | 0.02 | 0.02 |
| 84 | 5,000 | Jakknife | LQHPT | 1.3 | 127.86 | 0.98 | 0.96 | 0.29 | 0.03 | 0.25 | 0.18 | 0.15 | 0.04 | 0.04 |
| 84 | 5,000 | Jakknife | LQH | 2 | 141.53 | 0.97 | 0.96 | 0.42 | 0.03 | 0.33 | 0.11 | 0.10 | 0.04 | 0.04 |
| 84 | 5,000 | Jakknife | LQH | 1.8 | 142.56 | 0.97 | 0.96 | 0.38 | 0.03 | 0.30 | 0.12 | 0.11 | 0.04 | 0.04 |
| 84 | 5,000 | Jakknife | LQHP | 1.3 | 146.08 | 0.97 | 0.97 | 0.23 | 0.02 | 0.18 | 0.14 | 0.13 | 0.01 | 0.01 |
| 84 | 5,000 | Jakknife | LQHP | 1.8 | 151.80 | 0.97 | 0.96 | 0.24 | 0.02 | 0.19 | 0.16 | 0.13 | 0.02 | 0.02 |
| 84 | 5,000 | Jakknife | LQH | 1.9 | 156.70 | 0.97 | 0.96 | 0.39 | 0.03 | 0.31 | 0.12 | 0.11 | 0.04 | 0.04 |
| 84 | 5,000 | Jakknife | LQH | 1.7 | 158.91 | 0.97 | 0.96 | 0.38 | 0.03 | 0.30 | 0.12 | 0.11 | 0.04 | 0.04 |
| 84 | 5,000 | Jakknife | LQH | 1.4 | 161.80 | 0.97 | 0.96 | 0.32 | 0.02 | 0.25 | 0.12 | 0.11 | 0.04 | 0.04 |
| 84 | 5,000 | Jakknife | LQH | 1.3 | 168.47 | 0.97 | 0.96 | 0.31 | 0.02 | 0.25 | 0.14 | 0.13 | 0.04 | 0.04 |
| 84 | 5,000 | Jakknife | LQHP | 1.9 | 176.62 | 0.97 | 0.96 | 0.25 | 0.02 | 0.20 | 0.16 | 0.13 | 0.02 | 0.02 |
| 84 | 5,000 | Jakknife | LQHP | 2 | 179.32 | 0.97 | 0.96 | 0.26 | 0.02 | 0.20 | 0.16 | 0.13 | 0.02 | 0.02 |
| 84 | 5,000 | Jakknife | LQHPT | 1.1 | 200.45 | 0.98 | 0.96 | 0.30 | 0.03 | 0.26 | 0.20 | 0.16 | 0.02 | 0.02 |
| 84 | 5,000 | Jakknife | H | 1.2 | 201.40 | 0.97 | 0.96 | 0.31 | 0.02 | 0.24 | 0.14 | 0.13 | 0.04 | 0.04 |
| 84 | 5,000 | Jakknife | LQHP | 1.2 | 216.66 | 0.97 | 0.97 | 0.22 | 0.02 | 0.18 | 0.12 | 0.11 | 0.01 | 0.01 |
| 84 | 5,000 | Jakknife | LQH | 1.1 | 224.66 | 0.97 | 0.96 | 0.29 | 0.02 | 0.23 | 0.16 | 0.13 | 0.04 | 0.04 |
| 84 | 5,000 | Jakknife | LQH | 1.2 | 229.40 | 0.97 | 0.96 | 0.30 | 0.02 | 0.24 | 0.14 | 0.13 | 0.04 | 0.04 |
| 84 | 5,000 | Jakknife | H | 1.4 | 268.14 | 0.97 | 0.96 | 0.32 | 0.02 | 0.25 | 0.14 | 0.13 | 0.04 | 0.04 |
| 84 | 5,000 | Jakknife | L | 0.1 | 296.13 | 0.82 | 0.82 | 3.00 | 0.09 | 1.55 | 0.11 | 0.10 | 0.00 | 0.00 |
| 84 | 5,000 | Jakknife | L | 0.2 | 296.13 | 0.82 | 0.82 | 3.00 | 0.09 | 1.55 | 0.11 | 0.10 | 0.00 | 0.00 |
| 84 | 5,000 | Jakknife | L | 0.3 | 296.13 | 0.82 | 0.82 | 3.00 | 0.09 | 1.55 | 0.11 | 0.10 | 0.00 | 0.00 |
| 84 | 5,000 | Jakknife | L | 0.4 | 296.13 | 0.82 | 0.82 | 3.00 | 0.09 | 1.55 | 0.11 | 0.10 | 0.00 | 0.00 |
| 84 | 5,000 | Jakknife | L | 0.5 | 296.13 | 0.82 | 0.82 | 3.00 | 0.09 | 1.55 | 0.11 | 0.10 | 0.00 | 0.00 |
| 84 | 5,000 | Jakknife | L | 0.6 | 296.13 | 0.82 | 0.82 | 3.00 | 0.09 | 1.55 | 0.11 | 0.10 | 0.00 | 0.00 |
| 84 | 5,000 | Jakknife | L | 0.7 | 296.15 | 0.82 | 0.81 | 3.00 | 0.09 | 1.55 | 0.11 | 0.10 | 0.00 | 0.00 |
| 84 | 5,000 | Jakknife | L | 0.8 | 296.24 | 0.82 | 0.81 | 3.00 | 0.09 | 1.55 | 0.11 | 0.10 | 0.00 | 0.00 |
| 84 | 5,000 | Jakknife | L | 0.9 | 296.31 | 0.82 | 0.81 | 2.99 | 0.09 | 1.55 | 0.11 | 0.10 | 0.00 | 0.00 |
| 84 | 5,000 | Jakknife | L | 1 | 296.43 | 0.82 | 0.81 | 2.99 | 0.09 | 1.55 | 0.11 | 0.10 | 0.00 | 0.00 |
| 84 | 5,000 | Jakknife | L | 1.1 | 296.48 | 0.82 | 0.81 | 2.99 | 0.09 | 1.55 | 0.11 | 0.10 | 0.00 | 0.00 |
| 84 | 5,000 | Jakknife | L | 1.2 | 296.59 | 0.82 | 0.81 | 2.99 | 0.09 | 1.55 | 0.11 | 0.10 | 0.00 | 0.00 |
| 84 | 5,000 | Jakknife | L | 1.3 | 296.74 | 0.82 | 0.81 | 2.99 | 0.09 | 1.55 | 0.11 | 0.10 | 0.00 | 0.00 |
| 84 | 5,000 | Jakknife | L | 1.4 | 296.93 | 0.82 | 0.81 | 2.99 | 0.09 | 1.55 | 0.11 | 0.10 | 0.00 | 0.00 |
| 84 | 5,000 | Jakknife | L | 1.5 | 297.10 | 0.82 | 0.81 | 2.98 | 0.09 | 1.54 | 0.11 | 0.10 | 0.00 | 0.00 |
| 84 | 5,000 | Jakknife | L | 1.6 | 297.32 | 0.82 | 0.81 | 2.98 | 0.09 | 1.54 | 0.11 | 0.10 | 0.00 | 0.00 |
| 84 | 5,000 | Jakknife | L | 1.7 | 297.57 | 0.82 | 0.81 | 2.98 | 0.09 | 1.54 | 0.11 | 0.10 | 0.00 | 0.00 |
| 84 | 5,000 | Jakknife | L | 1.8 | 300.25 | 0.82 | 0.81 | 2.97 | 0.09 | 1.53 | 0.11 | 0.10 | 0.00 | 0.00 |
| 84 | 5,000 | Jakknife | L | 1.9 | 300.52 | 0.82 | 0.81 | 2.97 | 0.09 | 1.53 | 0.11 | 0.10 | 0.00 | 0.00 |
| 84 | 5,000 | Jakknife | L | 2 | 300.80 | 0.82 | 0.81 | 2.96 | 0.09 | 1.52 | 0.11 | 0.10 | 0.00 | 0.00 |
| 84 | 5,000 | Jakknife | H | 1.9 | 320.19 | 0.97 | 0.96 | 0.37 | 0.03 | 0.29 | 0.11 | 0.10 | 0.04 | 0.04 |
| 84 | 5,000 | Jakknife | H | 1.3 | 323.20 | 0.97 | 0.96 | 0.31 | 0.02 | 0.25 | 0.14 | 0.13 | 0.04 | 0.04 |
| 84 | 5,000 | Jakknife | LQHP | 1 | 392.93 | 0.98 | 0.97 | 0.21 | 0.02 | 0.18 | 0.14 | 0.13 | 0.01 | 0.01 |
| 84 | 5,000 | Jakknife | H | 2 | 417.69 | 0.97 | 0.96 | 0.38 | 0.03 | 0.30 | 0.11 | 0.10 | 0.04 | 0.04 |
| 84 | 5,000 | Jakknife | H | 1.1 | 419.27 | 0.97 | 0.96 | 0.30 | 0.02 | 0.23 | 0.14 | 0.13 | 0.04 | 0.04 |
| 84 | 5,000 | Jakknife | H | 1.5 | 433.88 | 0.97 | 0.96 | 0.33 | 0.02 | 0.26 | 0.12 | 0.11 | 0.04 | 0.04 |
| 84 | 5,000 | Jakknife | H | 1.6 | 436.82 | 0.97 | 0.96 | 0.35 | 0.02 | 0.27 | 0.12 | 0.11 | 0.04 | 0.04 |
| 84 | 5,000 | Jakknife | LQHPT | 1 | 457.38 | 0.99 | 0.97 | 0.32 | 0.03 | 0.29 | 0.18 | 0.15 | 0.05 | 0.05 |
| 84 | 5,000 | Jakknife | H | 1.7 | 507.22 | 0.97 | 0.96 | 0.35 | 0.03 | 0.28 | 0.12 | 0.11 | 0.04 | 0.04 |
| 84 | 5,000 | Jakknife | LQH | 1 | 515.16 | 0.97 | 0.96 | 0.29 | 0.02 | 0.24 | 0.16 | 0.13 | 0.04 | 0.04 |
| 84 | 5,000 | Jakknife | H | 1.8 | 645.06 | 0.97 | 0.96 | 0.36 | 0.03 | 0.29 | 0.12 | 0.11 | 0.04 | 0.04 |
| 84 | 5,000 | Jakknife | H | 1 | 668.95 | 0.97 | 0.96 | 0.29 | 0.02 | 0.23 | 0.14 | 0.13 | 0.04 | 0.04 |
| 84 | 5,000 | Jakknife | LQHP | 1.1 | 889.13 | 0.98 | 0.97 | 0.22 | 0.02 | 0.18 | 0.13 | 0.12 | 0.02 | 0.02 |
| 84 | 5,000 | Jakknife | LQHPT | 0.9 | 1346.06 | 0.99 | 0.96 | 0.40 | 0.03 | 0.37 | 0.20 | 0.16 | 0.07 | 0.07 |
| 84 | 5,000 | Jakknife | LQH | 0.9 | 1452.44 | 0.97 | 0.96 | 0.29 | 0.02 | 0.24 | 0.14 | 0.13 | 0.04 | 0.04 |
| 84 | 5,000 | Jakknife | H | 0.9 | 2020.67 | 0.97 | 0.96 | 0.28 | 0.02 | 0.23 | 0.14 | 0.13 | 0.04 | 0.04 |
| 84 | 5,000 | Jakknife | LQHP | 0.9 | 2456.86 | 0.98 | 0.97 | 0.22 | 0.02 | 0.18 | 0.17 | 0.14 | 0.02 | 0.02 |
| 84 | 5,000 | Jakknife | H | 0.8 | 13355.69 | 0.98 | 0.96 | 0.28 | 0.02 | 0.23 | 0.14 | 0.13 | 0.04 | 0.04 |
| 84 | 5,000 | Jakknife | H | 0.1 | NA | 0.99 | 0.98 | 0.14 | 0.02 | 0.13 | 0.22 | 0.17 | 0.07 | 0.07 |
| 84 | 5,000 | Jakknife | LQH | 0.1 | NA | 0.99 | 0.98 | 0.14 | 0.02 | 0.13 | 0.20 | 0.16 | 0.08 | 0.08 |
| 84 | 5,000 | Jakknife | LQHP | 0.1 | NA | 0.99 | 0.98 | 0.14 | 0.02 | 0.12 | 0.22 | 0.17 | 0.06 | 0.06 |
| 84 | 5,000 | Jakknife | LQHPT | 0.1 | NA | 0.99 | 0.96 | 0.61 | 0.03 | 0.58 | 0.41 | 0.24 | 0.05 | 0.05 |
| 84 | 5,000 | Jakknife | H | 0.2 | NA | 0.99 | 0.98 | 0.15 | 0.02 | 0.13 | 0.20 | 0.16 | 0.07 | 0.07 |
| 84 | 5,000 | Jakknife | LQH | 0.2 | NA | 0.99 | 0.98 | 0.14 | 0.02 | 0.13 | 0.20 | 0.16 | 0.06 | 0.06 |
| 84 | 5,000 | Jakknife | LQHP | 0.2 | NA | 0.99 | 0.98 | 0.15 | 0.02 | 0.13 | 0.22 | 0.17 | 0.05 | 0.05 |
| 84 | 5,000 | Jakknife | LQHPT | 0.2 | NA | 0.99 | 0.97 | 0.32 | 0.03 | 0.31 | 0.36 | 0.23 | 0.07 | 0.07 |
| 84 | 5,000 | Jakknife | H | 0.3 | NA | 0.99 | 0.97 | 0.17 | 0.02 | 0.15 | 0.19 | 0.16 | 0.05 | 0.05 |
| 84 | 5,000 | Jakknife | LQH | 0.3 | NA | 0.99 | 0.97 | 0.17 | 0.02 | 0.15 | 0.19 | 0.16 | 0.05 | 0.05 |
| 84 | 5,000 | Jakknife | LQHP | 0.3 | NA | 0.99 | 0.98 | 0.16 | 0.02 | 0.14 | 0.18 | 0.15 | 0.02 | 0.02 |
| 84 | 5,000 | Jakknife | LQHPT | 0.3 | NA | 0.99 | 0.98 | 0.17 | 0.02 | 0.16 | 0.34 | 0.23 | 0.07 | 0.07 |
| 84 | 5,000 | Jakknife | H | 0.4 | NA | 0.98 | 0.97 | 0.19 | 0.02 | 0.16 | 0.18 | 0.15 | 0.04 | 0.04 |
| 84 | 5,000 | Jakknife | LQH | 0.4 | NA | 0.98 | 0.97 | 0.19 | 0.02 | 0.16 | 0.18 | 0.15 | 0.04 | 0.04 |
| 84 | 5,000 | Jakknife | LQHP | 0.4 | NA | 0.98 | 0.97 | 0.17 | 0.02 | 0.15 | 0.19 | 0.16 | 0.02 | 0.02 |
| 84 | 5,000 | Jakknife | LQHPT | 0.4 | NA | 0.99 | 0.97 | 0.26 | 0.02 | 0.25 | 0.31 | 0.22 | 0.05 | 0.05 |
| 84 | 5,000 | Jakknife | H | 0.5 | NA | 0.98 | 0.97 | 0.21 | 0.02 | 0.18 | 0.12 | 0.11 | 0.04 | 0.04 |
| 84 | 5,000 | Jakknife | LQH | 0.5 | NA | 0.98 | 0.97 | 0.22 | 0.02 | 0.18 | 0.12 | 0.11 | 0.04 | 0.04 |
| 84 | 5,000 | Jakknife | LQHP | 0.5 | NA | 0.98 | 0.97 | 0.18 | 0.02 | 0.15 | 0.17 | 0.14 | 0.02 | 0.02 |
| 84 | 5,000 | Jakknife | LQHPT | 0.5 | NA | 0.99 | 0.97 | 0.24 | 0.02 | 0.23 | 0.29 | 0.21 | 0.07 | 0.07 |
| 84 | 5,000 | Jakknife | H | 0.6 | NA | 0.98 | 0.97 | 0.24 | 0.02 | 0.19 | 0.14 | 0.13 | 0.04 | 0.04 |
| 84 | 5,000 | Jakknife | LQH | 0.6 | NA | 0.98 | 0.97 | 0.26 | 0.02 | 0.21 | 0.14 | 0.13 | 0.04 | 0.04 |
| 84 | 5,000 | Jakknife | LQHP | 0.6 | NA | 0.98 | 0.97 | 0.20 | 0.02 | 0.17 | 0.16 | 0.13 | 0.02 | 0.02 |
| 84 | 5,000 | Jakknife | LQHPT | 0.6 | NA | 0.99 | 0.97 | 0.30 | 0.03 | 0.29 | 0.31 | 0.22 | 0.06 | 0.06 |
| 84 | 5,000 | Jakknife | H | 0.7 | NA | 0.98 | 0.97 | 0.27 | 0.02 | 0.22 | 0.14 | 0.13 | 0.04 | 0.04 |
| 84 | 5,000 | Jakknife | LQH | 0.7 | NA | 0.98 | 0.96 | 0.29 | 0.02 | 0.24 | 0.14 | 0.13 | 0.04 | 0.04 |
| 84 | 5,000 | Jakknife | LQHP | 0.7 | NA | 0.98 | 0.97 | 0.20 | 0.02 | 0.17 | 0.16 | 0.13 | 0.02 | 0.02 |
| 84 | 5,000 | Jakknife | LQHPT | 0.7 | NA | 0.99 | 0.97 | 0.36 | 0.03 | 0.34 | 0.24 | 0.19 | 0.02 | 0.02 |
| 84 | 5,000 | Jakknife | LQH | 0.8 | NA | 0.98 | 0.96 | 0.29 | 0.02 | 0.24 | 0.14 | 0.13 | 0.04 | 0.04 |
| 84 | 5,000 | Jakknife | LQHP | 0.8 | NA | 0.98 | 0.97 | 0.22 | 0.02 | 0.18 | 0.16 | 0.13 | 0.02 | 0.02 |
| 84 | 5,000 | Jakknife | LQHPT | 0.8 | NA | 0.99 | 0.97 | 0.43 | 0.03 | 0.41 | 0.20 | 0.16 | 0.05 | 0.05 |
| 84 | 5,000 | Random-k-fold | LQHPT | 2 | 0.00 | 0.98 | 0.96 | 0.00 | 0.02 | 0.00 | 0.18 | 0.00 | 0.05 | 0.00 |
| 84 | 5,000 | Random-k-fold | LQ | 0.6 | 3.20 | 0.97 | 0.96 | 0.00 | 0.01 | 0.00 | 0.14 | 0.00 | 0.04 | 0.00 |
| 84 | 5,000 | Random-k-fold | LQ | 0.8 | 6.39 | 0.97 | 0.95 | 0.00 | 0.01 | 0.00 | 0.16 | 0.00 | 0.04 | 0.00 |
| 84 | 5,000 | Random-k-fold | LQHPT | 1.9 | 6.48 | 0.98 | 0.96 | 0.00 | 0.02 | 0.00 | 0.18 | 0.00 | 0.05 | 0.00 |
| 84 | 5,000 | Random-k-fold | LQ | 0.1 | 8.12 | 0.97 | 0.96 | 0.00 | 0.01 | 0.00 | 0.14 | 0.00 | 0.04 | 0.00 |
| 84 | 5,000 | Random-k-fold | LQ | 0.2 | 8.12 | 0.97 | 0.96 | 0.00 | 0.01 | 0.00 | 0.14 | 0.00 | 0.04 | 0.00 |
| 84 | 5,000 | Random-k-fold | LQ | 0.3 | 8.12 | 0.97 | 0.96 | 0.00 | 0.01 | 0.00 | 0.14 | 0.00 | 0.04 | 0.00 |
| 84 | 5,000 | Random-k-fold | LQ | 0.4 | 8.12 | 0.97 | 0.96 | 0.00 | 0.01 | 0.00 | 0.14 | 0.00 | 0.04 | 0.00 |
| 84 | 5,000 | Random-k-fold | LQ | 0.5 | 8.12 | 0.97 | 0.96 | 0.00 | 0.01 | 0.00 | 0.14 | 0.00 | 0.04 | 0.00 |
| 84 | 5,000 | Random-k-fold | LQHPT | 1.6 | 9.14 | 0.98 | 0.96 | 0.00 | 0.02 | 0.00 | 0.20 | 0.00 | 0.07 | 0.00 |
| 84 | 5,000 | Random-k-fold | LQ | 0.7 | 10.32 | 0.97 | 0.96 | 0.00 | 0.01 | 0.00 | 0.16 | 0.00 | 0.04 | 0.00 |
| 84 | 5,000 | Random-k-fold | LQ | 1.1 | 14.68 | 0.97 | 0.95 | 0.00 | 0.02 | 0.00 | 0.16 | 0.00 | 0.04 | 0.00 |
| 84 | 5,000 | Random-k-fold | LQ | 1.2 | 15.01 | 0.97 | 0.95 | 0.00 | 0.02 | 0.00 | 0.14 | 0.01 | 0.04 | 0.00 |
| 84 | 5,000 | Random-k-fold | LQHPT | 1.7 | 17.04 | 0.98 | 0.96 | 0.00 | 0.02 | 0.00 | 0.20 | 0.00 | 0.06 | 0.00 |
| 84 | 5,000 | Random-k-fold | LQ | 0.9 | 17.96 | 0.97 | 0.95 | 0.00 | 0.02 | 0.00 | 0.14 | 0.00 | 0.04 | 0.00 |
| 84 | 5,000 | Random-k-fold | LQ | 1.6 | 17.98 | 0.97 | 0.95 | 0.00 | 0.02 | 0.00 | 0.13 | 0.00 | 0.04 | 0.00 |
| 84 | 5,000 | Random-k-fold | LQ | 1 | 18.95 | 0.97 | 0.95 | 0.00 | 0.02 | 0.00 | 0.16 | 0.00 | 0.04 | 0.00 |
| 84 | 5,000 | Random-k-fold | LQ | 1.5 | 21.47 | 0.97 | 0.95 | 0.00 | 0.02 | 0.00 | 0.13 | 0.00 | 0.04 | 0.00 |
| 84 | 5,000 | Random-k-fold | LQHPT | 1.5 | 22.80 | 0.98 | 0.96 | 0.00 | 0.02 | 0.00 | 0.20 | 0.00 | 0.07 | 0.00 |
| 84 | 5,000 | Random-k-fold | LQ | 1.3 | 23.89 | 0.97 | 0.95 | 0.00 | 0.01 | 0.00 | 0.14 | 0.01 | 0.04 | 0.00 |
| 84 | 5,000 | Random-k-fold | LQ | 1.4 | 25.56 | 0.97 | 0.95 | 0.00 | 0.02 | 0.00 | 0.13 | 0.00 | 0.04 | 0.00 |
| 84 | 5,000 | Random-k-fold | LQ | 1.8 | 27.07 | 0.97 | 0.95 | 0.00 | 0.02 | 0.00 | 0.13 | 0.00 | 0.04 | 0.00 |
| 84 | 5,000 | Random-k-fold | LQ | 1.9 | 29.36 | 0.97 | 0.95 | 0.00 | 0.02 | 0.00 | 0.13 | 0.00 | 0.04 | 0.00 |
| 84 | 5,000 | Random-k-fold | LQ | 1.7 | 30.76 | 0.97 | 0.95 | 0.00 | 0.02 | 0.00 | 0.13 | 0.00 | 0.04 | 0.00 |
| 84 | 5,000 | Random-k-fold | LQHPT | 1.8 | 32.96 | 0.98 | 0.96 | 0.00 | 0.02 | 0.00 | 0.19 | 0.00 | 0.06 | 0.00 |
| 84 | 5,000 | Random-k-fold | LQ | 2 | 33.79 | 0.97 | 0.94 | 0.00 | 0.02 | 0.00 | 0.13 | 0.00 | 0.04 | 0.00 |
| 84 | 5,000 | Random-k-fold | LQHPT | 1.4 | 82.28 | 0.98 | 0.96 | 0.00 | 0.02 | 0.00 | 0.22 | 0.00 | 0.07 | 0.00 |
| 84 | 5,000 | Random-k-fold | LQHP | 1.6 | 95.53 | 0.97 | 0.96 | 0.00 | 0.01 | 0.00 | 0.13 | 0.00 | 0.04 | 0.00 |
| 84 | 5,000 | Random-k-fold | LQH | 1.6 | 98.50 | 0.97 | 0.96 | 0.00 | 0.01 | 0.00 | 0.11 | 0.00 | 0.04 | 0.00 |
| 84 | 5,000 | Random-k-fold | LQH | 1.5 | 102.89 | 0.97 | 0.96 | 0.00 | 0.01 | 0.00 | 0.11 | 0.00 | 0.02 | 0.00 |
| 84 | 5,000 | Random-k-fold | LQHPT | 1.2 | 103.16 | 0.98 | 0.96 | 0.00 | 0.02 | 0.00 | 0.22 | 0.01 | 0.07 | 0.00 |
| 84 | 5,000 | Random-k-fold | LQHP | 1.4 | 103.74 | 0.97 | 0.96 | 0.00 | 0.01 | 0.00 | 0.13 | 0.00 | 0.04 | 0.00 |
| 84 | 5,000 | Random-k-fold | LQHP | 1.5 | 114.87 | 0.97 | 0.96 | 0.00 | 0.01 | 0.00 | 0.13 | 0.00 | 0.04 | 0.00 |
| 84 | 5,000 | Random-k-fold | LQHP | 1.7 | 122.08 | 0.97 | 0.96 | 0.00 | 0.01 | 0.00 | 0.11 | 0.00 | 0.04 | 0.00 |
| 84 | 5,000 | Random-k-fold | LQHPT | 1.3 | 127.86 | 0.98 | 0.96 | 0.00 | 0.02 | 0.00 | 0.22 | 0.00 | 0.07 | 0.00 |
| 84 | 5,000 | Random-k-fold | LQH | 2 | 141.53 | 0.97 | 0.95 | 0.00 | 0.01 | 0.00 | 0.13 | 0.00 | 0.04 | 0.00 |
| 84 | 5,000 | Random-k-fold | LQH | 1.8 | 142.56 | 0.97 | 0.95 | 0.00 | 0.01 | 0.00 | 0.13 | 0.00 | 0.04 | 0.00 |
| 84 | 5,000 | Random-k-fold | LQHP | 1.3 | 146.08 | 0.97 | 0.96 | 0.00 | 0.01 | 0.00 | 0.13 | 0.00 | 0.05 | 0.00 |
| 84 | 5,000 | Random-k-fold | LQHP | 1.8 | 151.80 | 0.97 | 0.96 | 0.00 | 0.01 | 0.00 | 0.11 | 0.00 | 0.04 | 0.00 |
| 84 | 5,000 | Random-k-fold | LQH | 1.9 | 156.70 | 0.97 | 0.95 | 0.00 | 0.01 | 0.00 | 0.13 | 0.00 | 0.04 | 0.00 |
| 84 | 5,000 | Random-k-fold | LQH | 1.7 | 158.91 | 0.97 | 0.95 | 0.00 | 0.01 | 0.00 | 0.13 | 0.00 | 0.04 | 0.00 |
| 84 | 5,000 | Random-k-fold | LQH | 1.4 | 161.80 | 0.97 | 0.96 | 0.00 | 0.01 | 0.00 | 0.11 | 0.00 | 0.05 | 0.00 |
| 84 | 5,000 | Random-k-fold | LQH | 1.3 | 168.47 | 0.97 | 0.96 | 0.00 | 0.01 | 0.00 | 0.11 | 0.00 | 0.05 | 0.00 |
| 84 | 5,000 | Random-k-fold | LQHP | 1.9 | 176.62 | 0.97 | 0.96 | 0.00 | 0.01 | 0.00 | 0.11 | 0.00 | 0.04 | 0.00 |
| 84 | 5,000 | Random-k-fold | LQHP | 2 | 179.32 | 0.97 | 0.95 | 0.00 | 0.01 | 0.00 | 0.11 | 0.00 | 0.04 | 0.00 |
| 84 | 5,000 | Random-k-fold | LQHPT | 1.1 | 200.45 | 0.98 | 0.96 | 0.00 | 0.02 | 0.00 | 0.24 | 0.01 | 0.07 | 0.00 |
| 84 | 5,000 | Random-k-fold | H | 1.2 | 201.40 | 0.97 | 0.96 | 0.00 | 0.01 | 0.00 | 0.11 | 0.00 | 0.05 | 0.00 |
| 84 | 5,000 | Random-k-fold | LQHP | 1.2 | 216.66 | 0.97 | 0.96 | 0.00 | 0.01 | 0.00 | 0.13 | 0.00 | 0.05 | 0.00 |
| 84 | 5,000 | Random-k-fold | LQH | 1.1 | 224.66 | 0.97 | 0.96 | 0.00 | 0.01 | 0.00 | 0.12 | 0.00 | 0.05 | 0.00 |
| 84 | 5,000 | Random-k-fold | LQH | 1.2 | 229.40 | 0.97 | 0.96 | 0.00 | 0.01 | 0.00 | 0.11 | 0.00 | 0.05 | 0.00 |
| 84 | 5,000 | Random-k-fold | H | 1.4 | 268.14 | 0.97 | 0.96 | 0.00 | 0.01 | 0.00 | 0.11 | 0.00 | 0.04 | 0.00 |
| 84 | 5,000 | Random-k-fold | L | 0.1 | 296.13 | 0.82 | 0.81 | 0.01 | 0.03 | 0.00 | 0.13 | 0.01 | 0.00 | 0.00 |
| 84 | 5,000 | Random-k-fold | L | 0.2 | 296.13 | 0.82 | 0.81 | 0.01 | 0.03 | 0.00 | 0.13 | 0.01 | 0.00 | 0.00 |
| 84 | 5,000 | Random-k-fold | L | 0.3 | 296.13 | 0.82 | 0.81 | 0.01 | 0.03 | 0.00 | 0.13 | 0.01 | 0.00 | 0.00 |
| 84 | 5,000 | Random-k-fold | L | 0.4 | 296.13 | 0.82 | 0.81 | 0.01 | 0.03 | 0.00 | 0.13 | 0.01 | 0.00 | 0.00 |
| 84 | 5,000 | Random-k-fold | L | 0.5 | 296.13 | 0.82 | 0.81 | 0.01 | 0.03 | 0.00 | 0.13 | 0.01 | 0.00 | 0.00 |
| 84 | 5,000 | Random-k-fold | L | 0.6 | 296.13 | 0.82 | 0.81 | 0.01 | 0.03 | 0.00 | 0.13 | 0.01 | 0.00 | 0.00 |
| 84 | 5,000 | Random-k-fold | L | 0.7 | 296.15 | 0.82 | 0.81 | 0.01 | 0.03 | 0.00 | 0.13 | 0.01 | 0.00 | 0.00 |
| 84 | 5,000 | Random-k-fold | L | 0.8 | 296.24 | 0.82 | 0.81 | 0.01 | 0.03 | 0.00 | 0.13 | 0.01 | 0.00 | 0.00 |
| 84 | 5,000 | Random-k-fold | L | 0.9 | 296.31 | 0.82 | 0.81 | 0.01 | 0.03 | 0.00 | 0.13 | 0.01 | 0.00 | 0.00 |
| 84 | 5,000 | Random-k-fold | L | 1 | 296.43 | 0.82 | 0.81 | 0.01 | 0.03 | 0.00 | 0.13 | 0.01 | 0.00 | 0.00 |
| 84 | 5,000 | Random-k-fold | L | 1.1 | 296.48 | 0.82 | 0.81 | 0.01 | 0.03 | 0.00 | 0.13 | 0.01 | 0.00 | 0.00 |
| 84 | 5,000 | Random-k-fold | L | 1.2 | 296.59 | 0.82 | 0.81 | 0.01 | 0.03 | 0.00 | 0.13 | 0.01 | 0.00 | 0.00 |
| 84 | 5,000 | Random-k-fold | L | 1.3 | 296.74 | 0.82 | 0.81 | 0.01 | 0.03 | 0.00 | 0.13 | 0.01 | 0.00 | 0.00 |
| 84 | 5,000 | Random-k-fold | L | 1.4 | 296.93 | 0.82 | 0.81 | 0.01 | 0.03 | 0.00 | 0.13 | 0.01 | 0.00 | 0.00 |
| 84 | 5,000 | Random-k-fold | L | 1.5 | 297.10 | 0.82 | 0.81 | 0.01 | 0.03 | 0.00 | 0.13 | 0.01 | 0.00 | 0.00 |
| 84 | 5,000 | Random-k-fold | L | 1.6 | 297.32 | 0.82 | 0.81 | 0.01 | 0.03 | 0.00 | 0.13 | 0.01 | 0.00 | 0.00 |
| 84 | 5,000 | Random-k-fold | L | 1.7 | 297.57 | 0.82 | 0.81 | 0.01 | 0.03 | 0.00 | 0.13 | 0.01 | 0.00 | 0.00 |
| 84 | 5,000 | Random-k-fold | L | 1.8 | 300.25 | 0.82 | 0.81 | 0.01 | 0.03 | 0.00 | 0.13 | 0.01 | 0.00 | 0.00 |
| 84 | 5,000 | Random-k-fold | L | 1.9 | 300.52 | 0.82 | 0.81 | 0.01 | 0.03 | 0.00 | 0.13 | 0.01 | 0.00 | 0.00 |
| 84 | 5,000 | Random-k-fold | L | 2 | 300.80 | 0.82 | 0.81 | 0.01 | 0.03 | 0.00 | 0.13 | 0.01 | 0.00 | 0.00 |
| 84 | 5,000 | Random-k-fold | H | 1.9 | 320.19 | 0.97 | 0.95 | 0.00 | 0.01 | 0.00 | 0.11 | 0.00 | 0.04 | 0.00 |
| 84 | 5,000 | Random-k-fold | H | 1.3 | 323.20 | 0.97 | 0.96 | 0.00 | 0.01 | 0.00 | 0.11 | 0.00 | 0.04 | 0.00 |
| 84 | 5,000 | Random-k-fold | LQHP | 1 | 392.93 | 0.98 | 0.96 | 0.00 | 0.01 | 0.00 | 0.13 | 0.00 | 0.04 | 0.00 |
| 84 | 5,000 | Random-k-fold | H | 2 | 417.69 | 0.97 | 0.95 | 0.00 | 0.01 | 0.00 | 0.11 | 0.00 | 0.04 | 0.00 |
| 84 | 5,000 | Random-k-fold | H | 1.1 | 419.27 | 0.97 | 0.96 | 0.00 | 0.01 | 0.00 | 0.13 | 0.00 | 0.05 | 0.00 |
| 84 | 5,000 | Random-k-fold | H | 1.5 | 433.88 | 0.97 | 0.96 | 0.00 | 0.01 | 0.00 | 0.11 | 0.00 | 0.04 | 0.00 |
| 84 | 5,000 | Random-k-fold | H | 1.6 | 436.82 | 0.97 | 0.96 | 0.00 | 0.01 | 0.00 | 0.11 | 0.00 | 0.04 | 0.00 |
| 84 | 5,000 | Random-k-fold | LQHPT | 1 | 457.38 | 0.99 | 0.96 | 0.00 | 0.02 | 0.00 | 0.24 | 0.01 | 0.10 | 0.01 |
| 84 | 5,000 | Random-k-fold | H | 1.7 | 507.22 | 0.97 | 0.95 | 0.00 | 0.01 | 0.00 | 0.11 | 0.00 | 0.04 | 0.00 |
| 84 | 5,000 | Random-k-fold | LQH | 1 | 515.16 | 0.97 | 0.96 | 0.00 | 0.01 | 0.00 | 0.13 | 0.00 | 0.05 | 0.00 |
| 84 | 5,000 | Random-k-fold | H | 1.8 | 645.06 | 0.97 | 0.95 | 0.00 | 0.01 | 0.00 | 0.11 | 0.00 | 0.04 | 0.00 |
| 84 | 5,000 | Random-k-fold | H | 1 | 668.95 | 0.97 | 0.96 | 0.00 | 0.01 | 0.00 | 0.13 | 0.00 | 0.05 | 0.00 |
| 84 | 5,000 | Random-k-fold | LQHP | 1.1 | 889.13 | 0.98 | 0.96 | 0.00 | 0.01 | 0.00 | 0.14 | 0.00 | 0.05 | 0.00 |
| 84 | 5,000 | Random-k-fold | LQHPT | 0.9 | 1346.06 | 0.99 | 0.96 | 0.00 | 0.02 | 0.00 | 0.24 | 0.01 | 0.10 | 0.01 |
| 84 | 5,000 | Random-k-fold | LQH | 0.9 | 1452.44 | 0.97 | 0.96 | 0.00 | 0.01 | 0.00 | 0.13 | 0.00 | 0.05 | 0.00 |
| 84 | 5,000 | Random-k-fold | H | 0.9 | 2020.67 | 0.97 | 0.96 | 0.00 | 0.01 | 0.00 | 0.14 | 0.00 | 0.05 | 0.00 |
| 84 | 5,000 | Random-k-fold | LQHP | 0.9 | 2456.86 | 0.98 | 0.96 | 0.00 | 0.01 | 0.00 | 0.14 | 0.00 | 0.05 | 0.00 |
| 84 | 5,000 | Random-k-fold | H | 0.8 | 13355.69 | 0.98 | 0.96 | 0.00 | 0.02 | 0.00 | 0.16 | 0.00 | 0.05 | 0.00 |
| 84 | 5,000 | Random-k-fold | H | 0.1 | NA | 0.99 | 0.97 | 0.00 | 0.02 | 0.00 | 0.19 | 0.01 | 0.10 | 0.00 |
| 84 | 5,000 | Random-k-fold | LQH | 0.1 | NA | 0.99 | 0.97 | 0.00 | 0.02 | 0.00 | 0.20 | 0.00 | 0.11 | 0.01 |
| 84 | 5,000 | Random-k-fold | LQHP | 0.1 | NA | 0.99 | 0.97 | 0.00 | 0.02 | 0.00 | 0.18 | 0.01 | 0.12 | 0.01 |
| 84 | 5,000 | Random-k-fold | LQHPT | 0.1 | NA | 0.99 | 0.95 | 0.00 | 0.05 | 0.00 | 0.40 | 0.02 | 0.17 | 0.00 |
| 84 | 5,000 | Random-k-fold | H | 0.2 | NA | 0.99 | 0.97 | 0.00 | 0.02 | 0.00 | 0.19 | 0.00 | 0.08 | 0.00 |
| 84 | 5,000 | Random-k-fold | LQH | 0.2 | NA | 0.99 | 0.97 | 0.00 | 0.02 | 0.00 | 0.19 | 0.00 | 0.08 | 0.00 |
| 84 | 5,000 | Random-k-fold | LQHP | 0.2 | NA | 0.99 | 0.97 | 0.00 | 0.02 | 0.00 | 0.18 | 0.01 | 0.08 | 0.00 |
| 84 | 5,000 | Random-k-fold | LQHPT | 0.2 | NA | 0.99 | 0.96 | 0.00 | 0.04 | 0.00 | 0.46 | 0.02 | 0.17 | 0.01 |
| 84 | 5,000 | Random-k-fold | H | 0.3 | NA | 0.99 | 0.96 | 0.00 | 0.02 | 0.00 | 0.19 | 0.00 | 0.05 | 0.00 |
| 84 | 5,000 | Random-k-fold | LQH | 0.3 | NA | 0.99 | 0.96 | 0.00 | 0.02 | 0.00 | 0.19 | 0.00 | 0.05 | 0.00 |
| 84 | 5,000 | Random-k-fold | LQHP | 0.3 | NA | 0.99 | 0.96 | 0.00 | 0.02 | 0.00 | 0.19 | 0.00 | 0.05 | 0.00 |
| 84 | 5,000 | Random-k-fold | LQHPT | 0.3 | NA | 0.99 | 0.96 | 0.00 | 0.04 | 0.00 | 0.37 | 0.02 | 0.17 | 0.00 |
| 84 | 5,000 | Random-k-fold | H | 0.4 | NA | 0.98 | 0.96 | 0.00 | 0.02 | 0.00 | 0.18 | 0.00 | 0.05 | 0.00 |
| 84 | 5,000 | Random-k-fold | LQH | 0.4 | NA | 0.98 | 0.96 | 0.00 | 0.02 | 0.00 | 0.18 | 0.00 | 0.05 | 0.00 |
| 84 | 5,000 | Random-k-fold | LQHP | 0.4 | NA | 0.98 | 0.96 | 0.00 | 0.02 | 0.00 | 0.18 | 0.01 | 0.05 | 0.00 |
| 84 | 5,000 | Random-k-fold | LQHPT | 0.4 | NA | 0.99 | 0.96 | 0.00 | 0.03 | 0.00 | 0.39 | 0.02 | 0.16 | 0.00 |
| 84 | 5,000 | Random-k-fold | H | 0.5 | NA | 0.98 | 0.96 | 0.00 | 0.02 | 0.00 | 0.18 | 0.00 | 0.05 | 0.00 |
| 84 | 5,000 | Random-k-fold | LQH | 0.5 | NA | 0.98 | 0.96 | 0.00 | 0.02 | 0.00 | 0.18 | 0.00 | 0.05 | 0.00 |
| 84 | 5,000 | Random-k-fold | LQHP | 0.5 | NA | 0.98 | 0.96 | 0.00 | 0.02 | 0.00 | 0.18 | 0.01 | 0.05 | 0.00 |
| 84 | 5,000 | Random-k-fold | LQHPT | 0.5 | NA | 0.99 | 0.96 | 0.00 | 0.03 | 0.00 | 0.39 | 0.01 | 0.12 | 0.00 |
| 84 | 5,000 | Random-k-fold | H | 0.6 | NA | 0.98 | 0.96 | 0.00 | 0.02 | 0.00 | 0.17 | 0.00 | 0.05 | 0.00 |
| 84 | 5,000 | Random-k-fold | LQH | 0.6 | NA | 0.98 | 0.96 | 0.00 | 0.02 | 0.00 | 0.17 | 0.00 | 0.05 | 0.00 |
| 84 | 5,000 | Random-k-fold | LQHP | 0.6 | NA | 0.98 | 0.96 | 0.00 | 0.02 | 0.00 | 0.19 | 0.01 | 0.05 | 0.00 |
| 84 | 5,000 | Random-k-fold | LQHPT | 0.6 | NA | 0.99 | 0.96 | 0.00 | 0.03 | 0.00 | 0.31 | 0.01 | 0.13 | 0.00 |
| 84 | 5,000 | Random-k-fold | H | 0.7 | NA | 0.98 | 0.96 | 0.00 | 0.02 | 0.00 | 0.16 | 0.00 | 0.05 | 0.00 |
| 84 | 5,000 | Random-k-fold | LQH | 0.7 | NA | 0.98 | 0.96 | 0.00 | 0.02 | 0.00 | 0.16 | 0.00 | 0.05 | 0.00 |
| 84 | 5,000 | Random-k-fold | LQHP | 0.7 | NA | 0.98 | 0.96 | 0.00 | 0.02 | 0.00 | 0.18 | 0.00 | 0.05 | 0.00 |
| 84 | 5,000 | Random-k-fold | LQHPT | 0.7 | NA | 0.99 | 0.96 | 0.00 | 0.03 | 0.00 | 0.26 | 0.02 | 0.08 | 0.01 |
| 84 | 5,000 | Random-k-fold | LQH | 0.8 | NA | 0.98 | 0.96 | 0.00 | 0.01 | 0.00 | 0.16 | 0.00 | 0.05 | 0.00 |
| 84 | 5,000 | Random-k-fold | LQHP | 0.8 | NA | 0.98 | 0.96 | 0.00 | 0.02 | 0.00 | 0.18 | 0.00 | 0.05 | 0.00 |
| 84 | 5,000 | Random-k-fold | LQHPT | 0.8 | NA | 0.99 | 0.96 | 0.00 | 0.03 | 0.00 | 0.23 | 0.01 | 0.10 | 0.01 |
| 84 | 5,000 | Block | LQHPT | 2 | 0.00 | 0.98 | 0.93 | 0.01 | 0.05 | 0.01 | 0.28 | 0.08 | 0.11 | 0.02 |
| 84 | 5,000 | Block | LQ | 0.6 | 3.20 | 0.97 | 0.95 | 0.00 | 0.02 | 0.00 | 0.12 | 0.01 | 0.02 | 0.00 |
| 84 | 5,000 | Block | LQ | 0.8 | 6.39 | 0.97 | 0.95 | 0.00 | 0.03 | 0.00 | 0.13 | 0.01 | 0.05 | 0.00 |
| 84 | 5,000 | Block | LQHPT | 1.9 | 6.48 | 0.98 | 0.93 | 0.01 | 0.05 | 0.01 | 0.30 | 0.09 | 0.10 | 0.01 |
| 84 | 5,000 | Block | LQ | 0.1 | 8.12 | 0.97 | 0.95 | 0.00 | 0.02 | 0.00 | 0.16 | 0.01 | 0.02 | 0.00 |
| 84 | 5,000 | Block | LQ | 0.2 | 8.12 | 0.97 | 0.95 | 0.00 | 0.03 | 0.00 | 0.15 | 0.01 | 0.05 | 0.00 |
| 84 | 5,000 | Block | LQ | 0.3 | 8.12 | 0.97 | 0.95 | 0.00 | 0.02 | 0.00 | 0.15 | 0.01 | 0.02 | 0.00 |
| 84 | 5,000 | Block | LQ | 0.4 | 8.12 | 0.97 | 0.94 | 0.00 | 0.03 | 0.00 | 0.16 | 0.02 | 0.05 | 0.00 |
| 84 | 5,000 | Block | LQ | 0.5 | 8.12 | 0.97 | 0.95 | 0.00 | 0.02 | 0.00 | 0.13 | 0.01 | 0.02 | 0.00 |
| 84 | 5,000 | Block | LQHPT | 1.6 | 9.14 | 0.98 | 0.93 | 0.01 | 0.05 | 0.01 | 0.35 | 0.08 | 0.12 | 0.01 |
| 84 | 5,000 | Block | LQ | 0.7 | 10.32 | 0.97 | 0.95 | 0.00 | 0.02 | 0.00 | 0.13 | 0.01 | 0.05 | 0.00 |
| 84 | 5,000 | Block | LQ | 1.1 | 14.68 | 0.97 | 0.94 | 0.00 | 0.03 | 0.00 | 0.13 | 0.01 | 0.07 | 0.01 |
| 84 | 5,000 | Block | LQ | 1.2 | 15.01 | 0.97 | 0.94 | 0.00 | 0.03 | 0.00 | 0.13 | 0.01 | 0.07 | 0.01 |
| 84 | 5,000 | Block | LQHPT | 1.7 | 17.04 | 0.98 | 0.93 | 0.01 | 0.05 | 0.01 | 0.33 | 0.08 | 0.11 | 0.02 |
| 84 | 5,000 | Block | LQ | 0.9 | 17.96 | 0.97 | 0.95 | 0.00 | 0.02 | 0.00 | 0.13 | 0.01 | 0.05 | 0.00 |
| 84 | 5,000 | Block | LQ | 1.6 | 17.98 | 0.97 | 0.94 | 0.01 | 0.03 | 0.00 | 0.18 | 0.03 | 0.07 | 0.01 |
| 84 | 5,000 | Block | LQ | 1 | 18.95 | 0.97 | 0.95 | 0.00 | 0.02 | 0.00 | 0.13 | 0.01 | 0.07 | 0.01 |
| 84 | 5,000 | Block | LQ | 1.5 | 21.47 | 0.97 | 0.94 | 0.01 | 0.03 | 0.00 | 0.17 | 0.03 | 0.07 | 0.01 |
| 84 | 5,000 | Block | LQHPT | 1.5 | 22.80 | 0.98 | 0.93 | 0.01 | 0.05 | 0.01 | 0.40 | 0.13 | 0.13 | 0.01 |
| 84 | 5,000 | Block | LQ | 1.3 | 23.89 | 0.97 | 0.94 | 0.00 | 0.03 | 0.00 | 0.13 | 0.01 | 0.07 | 0.01 |
| 84 | 5,000 | Block | LQ | 1.4 | 25.56 | 0.97 | 0.94 | 0.01 | 0.03 | 0.00 | 0.16 | 0.02 | 0.07 | 0.01 |
| 84 | 5,000 | Block | LQ | 1.8 | 27.07 | 0.97 | 0.94 | 0.01 | 0.03 | 0.00 | 0.18 | 0.04 | 0.07 | 0.01 |
| 84 | 5,000 | Block | LQ | 1.9 | 29.36 | 0.97 | 0.93 | 0.01 | 0.03 | 0.00 | 0.19 | 0.05 | 0.07 | 0.01 |
| 84 | 5,000 | Block | LQ | 1.7 | 30.76 | 0.97 | 0.94 | 0.01 | 0.03 | 0.00 | 0.18 | 0.04 | 0.07 | 0.01 |
| 84 | 5,000 | Block | LQHPT | 1.8 | 32.96 | 0.98 | 0.93 | 0.01 | 0.05 | 0.01 | 0.33 | 0.08 | 0.11 | 0.02 |
| 84 | 5,000 | Block | LQ | 2 | 33.79 | 0.97 | 0.93 | 0.01 | 0.03 | 0.01 | 0.19 | 0.05 | 0.07 | 0.01 |
| 84 | 5,000 | Block | LQHPT | 1.4 | 82.28 | 0.98 | 0.93 | 0.01 | 0.05 | 0.01 | 0.44 | 0.14 | 0.13 | 0.01 |
| 84 | 5,000 | Block | LQHP | 1.6 | 95.53 | 0.97 | 0.93 | 0.01 | 0.04 | 0.00 | 0.18 | 0.02 | 0.06 | 0.01 |
| 84 | 5,000 | Block | LQH | 1.6 | 98.50 | 0.97 | 0.94 | 0.01 | 0.03 | 0.00 | 0.18 | 0.02 | 0.05 | 0.01 |
| 84 | 5,000 | Block | LQH | 1.5 | 102.89 | 0.97 | 0.94 | 0.01 | 0.03 | 0.00 | 0.18 | 0.02 | 0.06 | 0.01 |
| 84 | 5,000 | Block | LQHPT | 1.2 | 103.16 | 0.98 | 0.93 | 0.01 | 0.06 | 0.01 | 0.44 | 0.14 | 0.13 | 0.01 |
| 84 | 5,000 | Block | LQHP | 1.4 | 103.74 | 0.97 | 0.94 | 0.01 | 0.04 | 0.00 | 0.19 | 0.02 | 0.06 | 0.01 |
| 84 | 5,000 | Block | LQHP | 1.5 | 114.87 | 0.97 | 0.94 | 0.01 | 0.04 | 0.00 | 0.19 | 0.02 | 0.06 | 0.01 |
| 84 | 5,000 | Block | LQHP | 1.7 | 122.08 | 0.97 | 0.93 | 0.01 | 0.04 | 0.00 | 0.18 | 0.02 | 0.06 | 0.01 |
| 84 | 5,000 | Block | LQHPT | 1.3 | 127.86 | 0.98 | 0.93 | 0.01 | 0.05 | 0.01 | 0.44 | 0.14 | 0.13 | 0.01 |
| 84 | 5,000 | Block | LQH | 2 | 141.53 | 0.97 | 0.94 | 0.01 | 0.03 | 0.00 | 0.17 | 0.02 | 0.05 | 0.01 |
| 84 | 5,000 | Block | LQH | 1.8 | 142.56 | 0.97 | 0.94 | 0.01 | 0.03 | 0.00 | 0.17 | 0.02 | 0.05 | 0.01 |
| 84 | 5,000 | Block | LQHP | 1.3 | 146.08 | 0.97 | 0.94 | 0.01 | 0.04 | 0.00 | 0.19 | 0.02 | 0.06 | 0.01 |
| 84 | 5,000 | Block | LQHP | 1.8 | 151.80 | 0.97 | 0.93 | 0.01 | 0.04 | 0.00 | 0.17 | 0.02 | 0.06 | 0.01 |
| 84 | 5,000 | Block | LQH | 1.9 | 156.70 | 0.97 | 0.94 | 0.01 | 0.03 | 0.00 | 0.17 | 0.02 | 0.05 | 0.01 |
| 84 | 5,000 | Block | LQH | 1.7 | 158.91 | 0.97 | 0.94 | 0.01 | 0.03 | 0.00 | 0.17 | 0.02 | 0.05 | 0.01 |
| 84 | 5,000 | Block | LQH | 1.4 | 161.80 | 0.97 | 0.94 | 0.01 | 0.03 | 0.00 | 0.18 | 0.02 | 0.06 | 0.01 |
| 84 | 5,000 | Block | LQH | 1.3 | 168.47 | 0.97 | 0.94 | 0.01 | 0.04 | 0.00 | 0.18 | 0.02 | 0.06 | 0.01 |
| 84 | 5,000 | Block | LQHP | 1.9 | 176.62 | 0.97 | 0.93 | 0.01 | 0.04 | 0.00 | 0.17 | 0.02 | 0.06 | 0.01 |
| 84 | 5,000 | Block | LQHP | 2 | 179.32 | 0.97 | 0.93 | 0.01 | 0.04 | 0.00 | 0.16 | 0.02 | 0.05 | 0.01 |
| 84 | 5,000 | Block | LQHPT | 1.1 | 200.45 | 0.98 | 0.92 | 0.01 | 0.06 | 0.01 | 0.44 | 0.14 | 0.13 | 0.01 |
| 84 | 5,000 | Block | H | 1.2 | 201.40 | 0.97 | 0.94 | 0.01 | 0.04 | 0.00 | 0.19 | 0.02 | 0.06 | 0.01 |
| 84 | 5,000 | Block | LQHP | 1.2 | 216.66 | 0.97 | 0.94 | 0.01 | 0.04 | 0.00 | 0.19 | 0.02 | 0.06 | 0.01 |
| 84 | 5,000 | Block | LQH | 1.1 | 224.66 | 0.97 | 0.94 | 0.00 | 0.04 | 0.00 | 0.22 | 0.03 | 0.06 | 0.01 |
| 84 | 5,000 | Block | LQH | 1.2 | 229.40 | 0.97 | 0.94 | 0.00 | 0.04 | 0.00 | 0.19 | 0.02 | 0.06 | 0.01 |
| 84 | 5,000 | Block | H | 1.4 | 268.14 | 0.97 | 0.94 | 0.01 | 0.04 | 0.00 | 0.19 | 0.02 | 0.06 | 0.01 |
| 84 | 5,000 | Block | L | 0.1 | 296.13 | 0.82 | 0.72 | 0.07 | 0.12 | 0.07 | 0.29 | 0.12 | 0.13 | 0.03 |
| 84 | 5,000 | Block | L | 0.2 | 296.13 | 0.82 | 0.72 | 0.07 | 0.12 | 0.07 | 0.29 | 0.12 | 0.13 | 0.03 |
| 84 | 5,000 | Block | L | 0.3 | 296.13 | 0.82 | 0.72 | 0.07 | 0.12 | 0.07 | 0.29 | 0.12 | 0.13 | 0.03 |
| 84 | 5,000 | Block | L | 0.4 | 296.13 | 0.82 | 0.72 | 0.07 | 0.12 | 0.07 | 0.29 | 0.12 | 0.13 | 0.03 |
| 84 | 5,000 | Block | L | 0.5 | 296.13 | 0.82 | 0.72 | 0.07 | 0.12 | 0.07 | 0.29 | 0.12 | 0.13 | 0.03 |
| 84 | 5,000 | Block | L | 0.6 | 296.13 | 0.82 | 0.72 | 0.07 | 0.12 | 0.07 | 0.29 | 0.12 | 0.13 | 0.03 |
| 84 | 5,000 | Block | L | 0.7 | 296.15 | 0.82 | 0.72 | 0.07 | 0.12 | 0.07 | 0.29 | 0.12 | 0.13 | 0.03 |
| 84 | 5,000 | Block | L | 0.8 | 296.24 | 0.82 | 0.72 | 0.06 | 0.12 | 0.07 | 0.29 | 0.12 | 0.12 | 0.03 |
| 84 | 5,000 | Block | L | 0.9 | 296.31 | 0.82 | 0.72 | 0.06 | 0.12 | 0.07 | 0.29 | 0.12 | 0.12 | 0.03 |
| 84 | 5,000 | Block | L | 1 | 296.43 | 0.82 | 0.72 | 0.06 | 0.12 | 0.07 | 0.29 | 0.12 | 0.12 | 0.03 |
| 84 | 5,000 | Block | L | 1.1 | 296.48 | 0.82 | 0.72 | 0.06 | 0.12 | 0.07 | 0.29 | 0.12 | 0.12 | 0.03 |
| 84 | 5,000 | Block | L | 1.2 | 296.59 | 0.82 | 0.72 | 0.06 | 0.12 | 0.07 | 0.29 | 0.12 | 0.12 | 0.03 |
| 84 | 5,000 | Block | L | 1.3 | 296.74 | 0.82 | 0.72 | 0.06 | 0.12 | 0.07 | 0.29 | 0.12 | 0.12 | 0.03 |
| 84 | 5,000 | Block | L | 1.4 | 296.93 | 0.82 | 0.72 | 0.06 | 0.12 | 0.06 | 0.29 | 0.12 | 0.10 | 0.04 |
| 84 | 5,000 | Block | L | 1.5 | 297.10 | 0.82 | 0.72 | 0.06 | 0.12 | 0.06 | 0.29 | 0.12 | 0.10 | 0.04 |
| 84 | 5,000 | Block | L | 1.6 | 297.32 | 0.82 | 0.72 | 0.06 | 0.11 | 0.06 | 0.29 | 0.12 | 0.10 | 0.04 |
| 84 | 5,000 | Block | L | 1.7 | 297.57 | 0.82 | 0.72 | 0.05 | 0.11 | 0.06 | 0.29 | 0.12 | 0.10 | 0.04 |
| 84 | 5,000 | Block | L | 1.8 | 300.25 | 0.82 | 0.72 | 0.05 | 0.11 | 0.06 | 0.28 | 0.11 | 0.10 | 0.04 |
| 84 | 5,000 | Block | L | 1.9 | 300.52 | 0.82 | 0.72 | 0.05 | 0.11 | 0.06 | 0.28 | 0.11 | 0.10 | 0.04 |
| 84 | 5,000 | Block | L | 2 | 300.80 | 0.82 | 0.72 | 0.05 | 0.11 | 0.06 | 0.28 | 0.11 | 0.10 | 0.04 |
| 84 | 5,000 | Block | H | 1.9 | 320.19 | 0.97 | 0.94 | 0.01 | 0.03 | 0.00 | 0.17 | 0.02 | 0.06 | 0.01 |
| 84 | 5,000 | Block | H | 1.3 | 323.20 | 0.97 | 0.94 | 0.01 | 0.04 | 0.00 | 0.19 | 0.02 | 0.06 | 0.01 |
| 84 | 5,000 | Block | LQHP | 1 | 392.93 | 0.98 | 0.94 | 0.00 | 0.04 | 0.00 | 0.23 | 0.02 | 0.06 | 0.01 |
| 84 | 5,000 | Block | H | 2 | 417.69 | 0.97 | 0.94 | 0.01 | 0.03 | 0.00 | 0.17 | 0.02 | 0.06 | 0.01 |
| 84 | 5,000 | Block | H | 1.1 | 419.27 | 0.97 | 0.94 | 0.01 | 0.04 | 0.00 | 0.22 | 0.03 | 0.06 | 0.01 |
| 84 | 5,000 | Block | H | 1.5 | 433.88 | 0.97 | 0.94 | 0.01 | 0.03 | 0.00 | 0.19 | 0.02 | 0.06 | 0.01 |
| 84 | 5,000 | Block | H | 1.6 | 436.82 | 0.97 | 0.94 | 0.01 | 0.03 | 0.00 | 0.18 | 0.02 | 0.06 | 0.01 |
| 84 | 5,000 | Block | LQHPT | 1 | 457.38 | 0.99 | 0.91 | 0.01 | 0.07 | 0.01 | 0.47 | 0.16 | 0.16 | 0.01 |
| 84 | 5,000 | Block | H | 1.7 | 507.22 | 0.97 | 0.94 | 0.01 | 0.03 | 0.00 | 0.17 | 0.02 | 0.06 | 0.01 |
| 84 | 5,000 | Block | LQH | 1 | 515.16 | 0.97 | 0.94 | 0.00 | 0.04 | 0.00 | 0.23 | 0.03 | 0.07 | 0.01 |
| 84 | 5,000 | Block | H | 1.8 | 645.06 | 0.97 | 0.94 | 0.01 | 0.03 | 0.00 | 0.17 | 0.02 | 0.06 | 0.01 |
| 84 | 5,000 | Block | H | 1 | 668.95 | 0.97 | 0.94 | 0.01 | 0.04 | 0.00 | 0.23 | 0.03 | 0.07 | 0.01 |
| 84 | 5,000 | Block | LQHP | 1.1 | 889.13 | 0.98 | 0.94 | 0.01 | 0.04 | 0.00 | 0.19 | 0.02 | 0.06 | 0.01 |
| 84 | 5,000 | Block | LQHPT | 0.9 | 1346.06 | 0.99 | 0.90 | 0.02 | 0.09 | 0.03 | 0.57 | 0.15 | 0.19 | 0.05 |
| 84 | 5,000 | Block | LQH | 0.9 | 1452.44 | 0.97 | 0.94 | 0.00 | 0.04 | 0.00 | 0.24 | 0.04 | 0.07 | 0.01 |
| 84 | 5,000 | Block | H | 0.9 | 2020.67 | 0.97 | 0.94 | 0.00 | 0.04 | 0.00 | 0.23 | 0.03 | 0.07 | 0.01 |
| 84 | 5,000 | Block | LQHP | 0.9 | 2456.86 | 0.98 | 0.94 | 0.00 | 0.04 | 0.00 | 0.28 | 0.03 | 0.06 | 0.01 |
| 84 | 5,000 | Block | H | 0.8 | 13355.69 | 0.98 | 0.94 | 0.00 | 0.05 | 0.00 | 0.27 | 0.03 | 0.07 | 0.01 |
| 84 | 5,000 | Block | H | 0.1 | NA | 0.99 | 0.89 | 0.03 | 0.10 | 0.03 | 0.70 | 0.07 | 0.44 | 0.11 |
| 84 | 5,000 | Block | LQH | 0.1 | NA | 0.99 | 0.89 | 0.03 | 0.10 | 0.03 | 0.73 | 0.05 | 0.44 | 0.11 |
| 84 | 5,000 | Block | LQHP | 0.1 | NA | 0.99 | 0.90 | 0.02 | 0.09 | 0.02 | 0.76 | 0.03 | 0.40 | 0.10 |
| 84 | 5,000 | Block | LQHPT | 0.1 | NA | 0.99 | 0.83 | 0.01 | 0.16 | 0.01 | 0.77 | 0.02 | 0.48 | 0.01 |
| 84 | 5,000 | Block | H | 0.2 | NA | 0.99 | 0.92 | 0.01 | 0.07 | 0.01 | 0.65 | 0.11 | 0.39 | 0.12 |
| 84 | 5,000 | Block | LQH | 0.2 | NA | 0.99 | 0.92 | 0.01 | 0.07 | 0.01 | 0.65 | 0.11 | 0.40 | 0.12 |
| 84 | 5,000 | Block | LQHP | 0.2 | NA | 0.99 | 0.92 | 0.01 | 0.07 | 0.01 | 0.64 | 0.10 | 0.38 | 0.12 |
| 84 | 5,000 | Block | LQHPT | 0.2 | NA | 0.99 | 0.82 | 0.01 | 0.17 | 0.01 | 0.76 | 0.02 | 0.53 | 0.01 |
| 84 | 5,000 | Block | H | 0.3 | NA | 0.99 | 0.92 | 0.01 | 0.07 | 0.01 | 0.57 | 0.15 | 0.29 | 0.12 |
| 84 | 5,000 | Block | LQH | 0.3 | NA | 0.99 | 0.92 | 0.01 | 0.07 | 0.01 | 0.57 | 0.15 | 0.29 | 0.12 |
| 84 | 5,000 | Block | LQHP | 0.3 | NA | 0.99 | 0.93 | 0.01 | 0.06 | 0.01 | 0.58 | 0.13 | 0.26 | 0.09 |
| 84 | 5,000 | Block | LQHPT | 0.3 | NA | 0.99 | 0.82 | 0.02 | 0.17 | 0.02 | 0.77 | 0.03 | 0.58 | 0.01 |
| 84 | 5,000 | Block | H | 0.4 | NA | 0.98 | 0.93 | 0.01 | 0.06 | 0.01 | 0.56 | 0.14 | 0.20 | 0.04 |
| 84 | 5,000 | Block | LQH | 0.4 | NA | 0.98 | 0.93 | 0.01 | 0.06 | 0.01 | 0.56 | 0.14 | 0.20 | 0.04 |
| 84 | 5,000 | Block | LQHP | 0.4 | NA | 0.98 | 0.93 | 0.01 | 0.06 | 0.01 | 0.53 | 0.14 | 0.21 | 0.04 |
| 84 | 5,000 | Block | LQHPT | 0.4 | NA | 0.99 | 0.84 | 0.01 | 0.15 | 0.01 | 0.77 | 0.03 | 0.52 | 0.01 |
| 84 | 5,000 | Block | H | 0.5 | NA | 0.98 | 0.93 | 0.01 | 0.05 | 0.01 | 0.52 | 0.12 | 0.14 | 0.03 |
| 84 | 5,000 | Block | LQH | 0.5 | NA | 0.98 | 0.93 | 0.01 | 0.06 | 0.01 | 0.52 | 0.13 | 0.13 | 0.02 |
| 84 | 5,000 | Block | LQHP | 0.5 | NA | 0.98 | 0.93 | 0.01 | 0.05 | 0.01 | 0.50 | 0.12 | 0.15 | 0.02 |
| 84 | 5,000 | Block | LQHPT | 0.5 | NA | 0.99 | 0.84 | 0.02 | 0.14 | 0.02 | 0.74 | 0.04 | 0.46 | 0.02 |
| 84 | 5,000 | Block | H | 0.6 | NA | 0.98 | 0.93 | 0.00 | 0.05 | 0.00 | 0.50 | 0.11 | 0.10 | 0.02 |
| 84 | 5,000 | Block | LQH | 0.6 | NA | 0.98 | 0.93 | 0.00 | 0.05 | 0.00 | 0.48 | 0.11 | 0.10 | 0.01 |
| 84 | 5,000 | Block | LQHP | 0.6 | NA | 0.98 | 0.94 | 0.01 | 0.05 | 0.00 | 0.47 | 0.11 | 0.13 | 0.01 |
| 84 | 5,000 | Block | LQHPT | 0.6 | NA | 0.99 | 0.86 | 0.02 | 0.13 | 0.02 | 0.71 | 0.06 | 0.37 | 0.02 |
| 84 | 5,000 | Block | H | 0.7 | NA | 0.98 | 0.93 | 0.00 | 0.05 | 0.00 | 0.38 | 0.07 | 0.07 | 0.01 |
| 84 | 5,000 | Block | LQH | 0.7 | NA | 0.98 | 0.94 | 0.00 | 0.05 | 0.00 | 0.35 | 0.06 | 0.07 | 0.01 |
| 84 | 5,000 | Block | LQHP | 0.7 | NA | 0.98 | 0.94 | 0.00 | 0.05 | 0.00 | 0.42 | 0.10 | 0.11 | 0.01 |
| 84 | 5,000 | Block | LQHPT | 0.7 | NA | 0.99 | 0.87 | 0.02 | 0.12 | 0.02 | 0.71 | 0.07 | 0.29 | 0.06 |
| 84 | 5,000 | Block | LQH | 0.8 | NA | 0.98 | 0.94 | 0.00 | 0.05 | 0.00 | 0.29 | 0.05 | 0.07 | 0.01 |
| 84 | 5,000 | Block | LQHP | 0.8 | NA | 0.98 | 0.94 | 0.00 | 0.04 | 0.00 | 0.28 | 0.03 | 0.06 | 0.01 |
| 84 | 5,000 | Block | LQHPT | 0.8 | NA | 0.99 | 0.88 | 0.03 | 0.10 | 0.03 | 0.59 | 0.18 | 0.24 | 0.05 |
| 84 | 5,000 | Checkerboar1 | LQHPT | 2 | 0.00 | 0.98 | 0.95 | 0.00 | 0.02 | 0.00 | 0.18 | 0.00 | 0.06 | 0.01 |
| 84 | 5,000 | Checkerboar1 | LQ | 0.6 | 3.20 | 0.97 | 0.96 | 0.00 | 0.02 | 0.00 | 0.14 | 0.04 | 0.04 | 0.00 |
| 84 | 5,000 | Checkerboar1 | LQ | 0.8 | 6.39 | 0.97 | 0.96 | 0.00 | 0.02 | 0.00 | 0.17 | 0.06 | 0.04 | 0.00 |
| 84 | 5,000 | Checkerboar1 | LQHPT | 1.9 | 6.48 | 0.98 | 0.95 | 0.00 | 0.02 | 0.00 | 0.18 | 0.00 | 0.06 | 0.01 |
| 84 | 5,000 | Checkerboar1 | LQ | 0.1 | 8.12 | 0.97 | 0.96 | 0.00 | 0.02 | 0.00 | 0.13 | 0.02 | 0.04 | 0.00 |
| 84 | 5,000 | Checkerboar1 | LQ | 0.2 | 8.12 | 0.97 | 0.96 | 0.00 | 0.02 | 0.00 | 0.13 | 0.02 | 0.04 | 0.00 |
| 84 | 5,000 | Checkerboar1 | LQ | 0.3 | 8.12 | 0.97 | 0.96 | 0.00 | 0.02 | 0.00 | 0.13 | 0.02 | 0.04 | 0.00 |
| 84 | 5,000 | Checkerboar1 | LQ | 0.4 | 8.12 | 0.97 | 0.96 | 0.00 | 0.02 | 0.00 | 0.13 | 0.02 | 0.04 | 0.00 |
| 84 | 5,000 | Checkerboar1 | LQ | 0.5 | 8.12 | 0.97 | 0.96 | 0.00 | 0.02 | 0.00 | 0.18 | 0.05 | 0.04 | 0.00 |
| 84 | 5,000 | Checkerboar1 | LQHPT | 1.6 | 9.14 | 0.98 | 0.95 | 0.00 | 0.02 | 0.00 | 0.22 | 0.00 | 0.06 | 0.01 |
| 84 | 5,000 | Checkerboar1 | LQ | 0.7 | 10.32 | 0.97 | 0.96 | 0.00 | 0.02 | 0.00 | 0.14 | 0.04 | 0.04 | 0.00 |
| 84 | 5,000 | Checkerboar1 | LQ | 1.1 | 14.68 | 0.97 | 0.95 | 0.00 | 0.02 | 0.00 | 0.14 | 0.03 | 0.04 | 0.00 |
| 84 | 5,000 | Checkerboar1 | LQ | 1.2 | 15.01 | 0.97 | 0.95 | 0.00 | 0.02 | 0.00 | 0.14 | 0.03 | 0.05 | 0.01 |
| 84 | 5,000 | Checkerboar1 | LQHPT | 1.7 | 17.04 | 0.98 | 0.95 | 0.00 | 0.02 | 0.00 | 0.22 | 0.00 | 0.06 | 0.01 |
| 84 | 5,000 | Checkerboar1 | LQ | 0.9 | 17.96 | 0.97 | 0.95 | 0.00 | 0.02 | 0.00 | 0.17 | 0.06 | 0.04 | 0.00 |
| 84 | 5,000 | Checkerboar1 | LQ | 1.6 | 17.98 | 0.97 | 0.94 | 0.00 | 0.02 | 0.00 | 0.15 | 0.02 | 0.04 | 0.00 |
| 84 | 5,000 | Checkerboar1 | LQ | 1 | 18.95 | 0.97 | 0.95 | 0.00 | 0.02 | 0.00 | 0.14 | 0.04 | 0.04 | 0.00 |
| 84 | 5,000 | Checkerboar1 | LQ | 1.5 | 21.47 | 0.97 | 0.94 | 0.00 | 0.02 | 0.00 | 0.15 | 0.02 | 0.04 | 0.00 |
| 84 | 5,000 | Checkerboar1 | LQHPT | 1.5 | 22.80 | 0.98 | 0.95 | 0.00 | 0.02 | 0.00 | 0.22 | 0.00 | 0.06 | 0.01 |
| 84 | 5,000 | Checkerboar1 | LQ | 1.3 | 23.89 | 0.97 | 0.94 | 0.00 | 0.02 | 0.00 | 0.13 | 0.02 | 0.05 | 0.01 |
| 84 | 5,000 | Checkerboar1 | LQ | 1.4 | 25.56 | 0.97 | 0.94 | 0.00 | 0.02 | 0.00 | 0.13 | 0.02 | 0.04 | 0.00 |
| 84 | 5,000 | Checkerboar1 | LQ | 1.8 | 27.07 | 0.97 | 0.94 | 0.00 | 0.02 | 0.00 | 0.15 | 0.02 | 0.04 | 0.00 |
| 84 | 5,000 | Checkerboar1 | LQ | 1.9 | 29.36 | 0.97 | 0.94 | 0.00 | 0.02 | 0.00 | 0.14 | 0.02 | 0.04 | 0.00 |
| 84 | 5,000 | Checkerboar1 | LQ | 1.7 | 30.76 | 0.97 | 0.94 | 0.00 | 0.02 | 0.00 | 0.18 | 0.03 | 0.04 | 0.00 |
| 84 | 5,000 | Checkerboar1 | LQHPT | 1.8 | 32.96 | 0.98 | 0.95 | 0.00 | 0.02 | 0.00 | 0.19 | 0.00 | 0.06 | 0.01 |
| 84 | 5,000 | Checkerboar1 | LQ | 2 | 33.79 | 0.97 | 0.93 | 0.00 | 0.02 | 0.00 | 0.15 | 0.01 | 0.04 | 0.00 |
| 84 | 5,000 | Checkerboar1 | LQHPT | 1.4 | 82.28 | 0.98 | 0.95 | 0.00 | 0.02 | 0.00 | 0.24 | 0.00 | 0.06 | 0.01 |
| 84 | 5,000 | Checkerboar1 | LQHP | 1.6 | 95.53 | 0.97 | 0.95 | 0.00 | 0.02 | 0.00 | 0.14 | 0.02 | 0.10 | 0.02 |
| 84 | 5,000 | Checkerboar1 | LQH | 1.6 | 98.50 | 0.97 | 0.95 | 0.00 | 0.02 | 0.00 | 0.12 | 0.03 | 0.09 | 0.02 |
| 84 | 5,000 | Checkerboar1 | LQH | 1.5 | 102.89 | 0.97 | 0.95 | 0.00 | 0.02 | 0.00 | 0.13 | 0.02 | 0.09 | 0.02 |
| 84 | 5,000 | Checkerboar1 | LQHPT | 1.2 | 103.16 | 0.98 | 0.95 | 0.00 | 0.03 | 0.00 | 0.28 | 0.00 | 0.06 | 0.01 |
| 84 | 5,000 | Checkerboar1 | LQHP | 1.4 | 103.74 | 0.97 | 0.95 | 0.00 | 0.02 | 0.00 | 0.14 | 0.02 | 0.10 | 0.02 |
| 84 | 5,000 | Checkerboar1 | LQHP | 1.5 | 114.87 | 0.97 | 0.95 | 0.00 | 0.02 | 0.00 | 0.14 | 0.02 | 0.10 | 0.02 |
| 84 | 5,000 | Checkerboar1 | LQHP | 1.7 | 122.08 | 0.97 | 0.95 | 0.00 | 0.02 | 0.00 | 0.13 | 0.02 | 0.10 | 0.02 |
| 84 | 5,000 | Checkerboar1 | LQHPT | 1.3 | 127.86 | 0.98 | 0.95 | 0.00 | 0.03 | 0.00 | 0.28 | 0.00 | 0.06 | 0.01 |
| 84 | 5,000 | Checkerboar1 | LQH | 2 | 141.53 | 0.97 | 0.95 | 0.00 | 0.02 | 0.00 | 0.12 | 0.03 | 0.09 | 0.02 |
| 84 | 5,000 | Checkerboar1 | LQH | 1.8 | 142.56 | 0.97 | 0.95 | 0.00 | 0.02 | 0.00 | 0.12 | 0.03 | 0.09 | 0.02 |
| 84 | 5,000 | Checkerboar1 | LQHP | 1.3 | 146.08 | 0.97 | 0.95 | 0.00 | 0.02 | 0.00 | 0.15 | 0.01 | 0.10 | 0.02 |
| 84 | 5,000 | Checkerboar1 | LQHP | 1.8 | 151.80 | 0.97 | 0.95 | 0.00 | 0.02 | 0.00 | 0.12 | 0.03 | 0.10 | 0.02 |
| 84 | 5,000 | Checkerboar1 | LQH | 1.9 | 156.70 | 0.97 | 0.95 | 0.00 | 0.02 | 0.00 | 0.12 | 0.03 | 0.09 | 0.02 |
| 84 | 5,000 | Checkerboar1 | LQH | 1.7 | 158.91 | 0.97 | 0.95 | 0.00 | 0.02 | 0.00 | 0.12 | 0.03 | 0.09 | 0.02 |
| 84 | 5,000 | Checkerboar1 | LQH | 1.4 | 161.80 | 0.97 | 0.95 | 0.00 | 0.02 | 0.00 | 0.13 | 0.02 | 0.09 | 0.02 |
| 84 | 5,000 | Checkerboar1 | LQH | 1.3 | 168.47 | 0.97 | 0.95 | 0.00 | 0.02 | 0.00 | 0.14 | 0.02 | 0.09 | 0.02 |
| 84 | 5,000 | Checkerboar1 | LQHP | 1.9 | 176.62 | 0.97 | 0.95 | 0.00 | 0.02 | 0.00 | 0.12 | 0.03 | 0.10 | 0.02 |
| 84 | 5,000 | Checkerboar1 | LQHP | 2 | 179.32 | 0.97 | 0.95 | 0.00 | 0.02 | 0.00 | 0.12 | 0.03 | 0.10 | 0.02 |
| 84 | 5,000 | Checkerboar1 | LQHPT | 1.1 | 200.45 | 0.98 | 0.95 | 0.00 | 0.03 | 0.00 | 0.28 | 0.00 | 0.06 | 0.01 |
| 84 | 5,000 | Checkerboar1 | H | 1.2 | 201.40 | 0.97 | 0.95 | 0.00 | 0.02 | 0.00 | 0.14 | 0.02 | 0.09 | 0.02 |
| 84 | 5,000 | Checkerboar1 | LQHP | 1.2 | 216.66 | 0.97 | 0.95 | 0.00 | 0.02 | 0.00 | 0.15 | 0.01 | 0.10 | 0.02 |
| 84 | 5,000 | Checkerboar1 | LQH | 1.1 | 224.66 | 0.97 | 0.95 | 0.00 | 0.02 | 0.00 | 0.14 | 0.02 | 0.09 | 0.02 |
| 84 | 5,000 | Checkerboar1 | LQH | 1.2 | 229.40 | 0.97 | 0.95 | 0.00 | 0.02 | 0.00 | 0.14 | 0.02 | 0.09 | 0.02 |
| 84 | 5,000 | Checkerboar1 | H | 1.4 | 268.14 | 0.97 | 0.95 | 0.00 | 0.02 | 0.00 | 0.14 | 0.02 | 0.09 | 0.02 |
| 84 | 5,000 | Checkerboar1 | L | 0.1 | 296.13 | 0.82 | 0.81 | 0.00 | 0.01 | 0.00 | 0.12 | 0.00 | 0.04 | 0.00 |
| 84 | 5,000 | Checkerboar1 | L | 0.2 | 296.13 | 0.82 | 0.81 | 0.00 | 0.01 | 0.00 | 0.12 | 0.00 | 0.04 | 0.00 |
| 84 | 5,000 | Checkerboar1 | L | 0.3 | 296.13 | 0.82 | 0.81 | 0.00 | 0.01 | 0.00 | 0.12 | 0.00 | 0.04 | 0.00 |
| 84 | 5,000 | Checkerboar1 | L | 0.4 | 296.13 | 0.82 | 0.81 | 0.00 | 0.01 | 0.00 | 0.12 | 0.00 | 0.04 | 0.00 |
| 84 | 5,000 | Checkerboar1 | L | 0.5 | 296.13 | 0.82 | 0.81 | 0.00 | 0.01 | 0.00 | 0.12 | 0.00 | 0.04 | 0.00 |
| 84 | 5,000 | Checkerboar1 | L | 0.6 | 296.13 | 0.82 | 0.81 | 0.00 | 0.01 | 0.00 | 0.12 | 0.00 | 0.03 | 0.00 |
| 84 | 5,000 | Checkerboar1 | L | 0.7 | 296.15 | 0.82 | 0.81 | 0.00 | 0.01 | 0.00 | 0.12 | 0.00 | 0.03 | 0.00 |
| 84 | 5,000 | Checkerboar1 | L | 0.8 | 296.24 | 0.82 | 0.81 | 0.00 | 0.01 | 0.00 | 0.11 | 0.00 | 0.03 | 0.00 |
| 84 | 5,000 | Checkerboar1 | L | 0.9 | 296.31 | 0.82 | 0.81 | 0.00 | 0.01 | 0.00 | 0.11 | 0.00 | 0.03 | 0.00 |
| 84 | 5,000 | Checkerboar1 | L | 1 | 296.43 | 0.82 | 0.81 | 0.00 | 0.01 | 0.00 | 0.11 | 0.00 | 0.00 | 0.00 |
| 84 | 5,000 | Checkerboar1 | L | 1.1 | 296.48 | 0.82 | 0.81 | 0.00 | 0.01 | 0.00 | 0.11 | 0.00 | 0.00 | 0.00 |
| 84 | 5,000 | Checkerboar1 | L | 1.2 | 296.59 | 0.82 | 0.81 | 0.00 | 0.01 | 0.00 | 0.12 | 0.01 | 0.00 | 0.00 |
| 84 | 5,000 | Checkerboar1 | L | 1.3 | 296.74 | 0.82 | 0.80 | 0.00 | 0.01 | 0.00 | 0.12 | 0.01 | 0.00 | 0.00 |
| 84 | 5,000 | Checkerboar1 | L | 1.4 | 296.93 | 0.82 | 0.80 | 0.00 | 0.01 | 0.00 | 0.12 | 0.01 | 0.00 | 0.00 |
| 84 | 5,000 | Checkerboar1 | L | 1.5 | 297.10 | 0.82 | 0.80 | 0.00 | 0.02 | 0.00 | 0.12 | 0.01 | 0.00 | 0.00 |
| 84 | 5,000 | Checkerboar1 | L | 1.6 | 297.32 | 0.82 | 0.80 | 0.00 | 0.02 | 0.00 | 0.12 | 0.01 | 0.00 | 0.00 |
| 84 | 5,000 | Checkerboar1 | L | 1.7 | 297.57 | 0.82 | 0.80 | 0.00 | 0.02 | 0.00 | 0.12 | 0.01 | 0.00 | 0.00 |
| 84 | 5,000 | Checkerboar1 | L | 1.8 | 300.25 | 0.82 | 0.80 | 0.00 | 0.02 | 0.00 | 0.12 | 0.01 | 0.00 | 0.00 |
| 84 | 5,000 | Checkerboar1 | L | 1.9 | 300.52 | 0.82 | 0.80 | 0.00 | 0.02 | 0.00 | 0.12 | 0.01 | 0.00 | 0.00 |
| 84 | 5,000 | Checkerboar1 | L | 2 | 300.80 | 0.82 | 0.80 | 0.00 | 0.02 | 0.00 | 0.12 | 0.01 | 0.00 | 0.00 |
| 84 | 5,000 | Checkerboar1 | H | 1.9 | 320.19 | 0.97 | 0.95 | 0.00 | 0.02 | 0.00 | 0.12 | 0.03 | 0.09 | 0.02 |
| 84 | 5,000 | Checkerboar1 | H | 1.3 | 323.20 | 0.97 | 0.95 | 0.00 | 0.02 | 0.00 | 0.14 | 0.02 | 0.09 | 0.02 |
| 84 | 5,000 | Checkerboar1 | LQHP | 1 | 392.93 | 0.98 | 0.95 | 0.00 | 0.02 | 0.00 | 0.17 | 0.01 | 0.10 | 0.02 |
| 84 | 5,000 | Checkerboar1 | H | 2 | 417.69 | 0.97 | 0.95 | 0.00 | 0.02 | 0.00 | 0.12 | 0.03 | 0.09 | 0.02 |
| 84 | 5,000 | Checkerboar1 | H | 1.1 | 419.27 | 0.97 | 0.95 | 0.00 | 0.02 | 0.00 | 0.15 | 0.01 | 0.09 | 0.02 |
| 84 | 5,000 | Checkerboar1 | H | 1.5 | 433.88 | 0.97 | 0.95 | 0.00 | 0.02 | 0.00 | 0.14 | 0.02 | 0.09 | 0.02 |
| 84 | 5,000 | Checkerboar1 | H | 1.6 | 436.82 | 0.97 | 0.95 | 0.00 | 0.02 | 0.00 | 0.13 | 0.02 | 0.09 | 0.02 |
| 84 | 5,000 | Checkerboar1 | LQHPT | 1 | 457.38 | 0.99 | 0.95 | 0.00 | 0.03 | 0.00 | 0.28 | 0.00 | 0.10 | 0.02 |
| 84 | 5,000 | Checkerboar1 | H | 1.7 | 507.22 | 0.97 | 0.95 | 0.00 | 0.02 | 0.00 | 0.12 | 0.03 | 0.09 | 0.02 |
| 84 | 5,000 | Checkerboar1 | LQH | 1 | 515.16 | 0.97 | 0.95 | 0.00 | 0.02 | 0.00 | 0.15 | 0.01 | 0.09 | 0.02 |
| 84 | 5,000 | Checkerboar1 | H | 1.8 | 645.06 | 0.97 | 0.95 | 0.00 | 0.02 | 0.00 | 0.12 | 0.03 | 0.09 | 0.02 |
| 84 | 5,000 | Checkerboar1 | H | 1 | 668.95 | 0.97 | 0.96 | 0.00 | 0.02 | 0.00 | 0.16 | 0.01 | 0.09 | 0.02 |
| 84 | 5,000 | Checkerboar1 | LQHP | 1.1 | 889.13 | 0.98 | 0.95 | 0.00 | 0.02 | 0.00 | 0.16 | 0.01 | 0.10 | 0.02 |
| 84 | 5,000 | Checkerboar1 | LQHPT | 0.9 | 1346.06 | 0.99 | 0.95 | 0.00 | 0.03 | 0.00 | 0.29 | 0.00 | 0.09 | 0.02 |
| 84 | 5,000 | Checkerboar1 | LQH | 0.9 | 1452.44 | 0.97 | 0.95 | 0.00 | 0.02 | 0.00 | 0.17 | 0.01 | 0.09 | 0.02 |
| 84 | 5,000 | Checkerboar1 | H | 0.9 | 2020.67 | 0.97 | 0.96 | 0.00 | 0.02 | 0.00 | 0.16 | 0.01 | 0.09 | 0.02 |
| 84 | 5,000 | Checkerboar1 | LQHP | 0.9 | 2456.86 | 0.98 | 0.95 | 0.00 | 0.02 | 0.00 | 0.17 | 0.01 | 0.10 | 0.02 |
| 84 | 5,000 | Checkerboar1 | H | 0.8 | 13355.69 | 0.98 | 0.96 | 0.00 | 0.02 | 0.00 | 0.16 | 0.01 | 0.09 | 0.02 |
| 84 | 5,000 | Checkerboar1 | H | 0.1 | NA | 0.99 | 0.96 | 0.00 | 0.03 | 0.00 | 0.30 | 0.00 | 0.20 | 0.00 |
| 84 | 5,000 | Checkerboar1 | LQH | 0.1 | NA | 0.99 | 0.96 | 0.00 | 0.03 | 0.00 | 0.30 | 0.00 | 0.23 | 0.00 |
| 84 | 5,000 | Checkerboar1 | LQHP | 0.1 | NA | 0.99 | 0.96 | 0.00 | 0.03 | 0.00 | 0.33 | 0.01 | 0.22 | 0.00 |
| 84 | 5,000 | Checkerboar1 | LQHPT | 0.1 | NA | 0.99 | 0.91 | 0.00 | 0.08 | 0.00 | 0.44 | 0.01 | 0.25 | 0.00 |
| 84 | 5,000 | Checkerboar1 | H | 0.2 | NA | 0.99 | 0.95 | 0.00 | 0.03 | 0.00 | 0.30 | 0.00 | 0.17 | 0.00 |
| 84 | 5,000 | Checkerboar1 | LQH | 0.2 | NA | 0.99 | 0.95 | 0.00 | 0.03 | 0.00 | 0.30 | 0.00 | 0.17 | 0.00 |
| 84 | 5,000 | Checkerboar1 | LQHP | 0.2 | NA | 0.99 | 0.95 | 0.00 | 0.03 | 0.00 | 0.31 | 0.00 | 0.19 | 0.00 |
| 84 | 5,000 | Checkerboar1 | LQHPT | 0.2 | NA | 0.99 | 0.91 | 0.00 | 0.08 | 0.00 | 0.41 | 0.00 | 0.20 | 0.01 |
| 84 | 5,000 | Checkerboar1 | H | 0.3 | NA | 0.99 | 0.95 | 0.00 | 0.03 | 0.00 | 0.30 | 0.00 | 0.13 | 0.01 |
| 84 | 5,000 | Checkerboar1 | LQH | 0.3 | NA | 0.99 | 0.95 | 0.00 | 0.03 | 0.00 | 0.30 | 0.00 | 0.13 | 0.01 |
| 84 | 5,000 | Checkerboar1 | LQHP | 0.3 | NA | 0.99 | 0.95 | 0.00 | 0.03 | 0.00 | 0.29 | 0.00 | 0.13 | 0.01 |
| 84 | 5,000 | Checkerboar1 | LQHPT | 0.3 | NA | 0.99 | 0.91 | 0.00 | 0.08 | 0.00 | 0.39 | 0.01 | 0.23 | 0.01 |
| 84 | 5,000 | Checkerboar1 | H | 0.4 | NA | 0.98 | 0.95 | 0.00 | 0.03 | 0.00 | 0.26 | 0.00 | 0.11 | 0.02 |
| 84 | 5,000 | Checkerboar1 | LQH | 0.4 | NA | 0.98 | 0.95 | 0.00 | 0.03 | 0.00 | 0.26 | 0.00 | 0.13 | 0.01 |
| 84 | 5,000 | Checkerboar1 | LQHP | 0.4 | NA | 0.98 | 0.95 | 0.00 | 0.03 | 0.00 | 0.26 | 0.00 | 0.11 | 0.02 |
| 84 | 5,000 | Checkerboar1 | LQHPT | 0.4 | NA | 0.99 | 0.91 | 0.00 | 0.07 | 0.00 | 0.35 | 0.00 | 0.22 | 0.01 |
| 84 | 5,000 | Checkerboar1 | H | 0.5 | NA | 0.98 | 0.95 | 0.00 | 0.03 | 0.00 | 0.23 | 0.00 | 0.10 | 0.02 |
| 84 | 5,000 | Checkerboar1 | LQH | 0.5 | NA | 0.98 | 0.96 | 0.00 | 0.03 | 0.00 | 0.22 | 0.00 | 0.10 | 0.02 |
| 84 | 5,000 | Checkerboar1 | LQHP | 0.5 | NA | 0.98 | 0.95 | 0.00 | 0.03 | 0.00 | 0.26 | 0.00 | 0.10 | 0.02 |
| 84 | 5,000 | Checkerboar1 | LQHPT | 0.5 | NA | 0.99 | 0.92 | 0.00 | 0.07 | 0.00 | 0.36 | 0.01 | 0.22 | 0.01 |
| 84 | 5,000 | Checkerboar1 | H | 0.6 | NA | 0.98 | 0.95 | 0.00 | 0.02 | 0.00 | 0.21 | 0.00 | 0.10 | 0.02 |
| 84 | 5,000 | Checkerboar1 | LQH | 0.6 | NA | 0.98 | 0.96 | 0.00 | 0.02 | 0.00 | 0.23 | 0.00 | 0.10 | 0.02 |
| 84 | 5,000 | Checkerboar1 | LQHP | 0.6 | NA | 0.98 | 0.95 | 0.00 | 0.03 | 0.00 | 0.25 | 0.00 | 0.10 | 0.02 |
| 84 | 5,000 | Checkerboar1 | LQHPT | 0.6 | NA | 0.99 | 0.93 | 0.00 | 0.05 | 0.00 | 0.32 | 0.00 | 0.15 | 0.03 |
| 84 | 5,000 | Checkerboar1 | H | 0.7 | NA | 0.98 | 0.95 | 0.00 | 0.02 | 0.00 | 0.18 | 0.00 | 0.10 | 0.02 |
| 84 | 5,000 | Checkerboar1 | LQH | 0.7 | NA | 0.98 | 0.96 | 0.00 | 0.02 | 0.00 | 0.18 | 0.00 | 0.10 | 0.02 |
| 84 | 5,000 | Checkerboar1 | LQHP | 0.7 | NA | 0.98 | 0.95 | 0.00 | 0.03 | 0.00 | 0.22 | 0.00 | 0.10 | 0.02 |
| 84 | 5,000 | Checkerboar1 | LQHPT | 0.7 | NA | 0.99 | 0.93 | 0.00 | 0.05 | 0.00 | 0.31 | 0.00 | 0.14 | 0.04 |
| 84 | 5,000 | Checkerboar1 | LQH | 0.8 | NA | 0.98 | 0.96 | 0.00 | 0.02 | 0.00 | 0.17 | 0.01 | 0.09 | 0.02 |
| 84 | 5,000 | Checkerboar1 | LQHP | 0.8 | NA | 0.98 | 0.95 | 0.00 | 0.02 | 0.00 | 0.17 | 0.01 | 0.10 | 0.02 |
| 84 | 5,000 | Checkerboar1 | LQHPT | 0.8 | NA | 0.99 | 0.95 | 0.00 | 0.04 | 0.00 | 0.30 | 0.00 | 0.13 | 0.03 |
| 84 | 10,000 | Jackknife | LQ | 0.1 | 0.00 | 0.97 | 0.96 | 0.31 | 0.02 | 0.24 | 0.12 | 0.11 | 0.01 | 0.01 |
| 84 | 10,000 | Jackknife | LQ | 0.2 | 0.00 | 0.97 | 0.96 | 0.31 | 0.02 | 0.24 | 0.12 | 0.11 | 0.01 | 0.01 |
| 84 | 10,000 | Jackknife | LQ | 0.3 | 0.00 | 0.97 | 0.96 | 0.31 | 0.02 | 0.24 | 0.12 | 0.11 | 0.01 | 0.01 |
| 84 | 10,000 | Jackknife | LQ | 0.4 | 0.00 | 0.97 | 0.96 | 0.31 | 0.02 | 0.24 | 0.12 | 0.11 | 0.01 | 0.01 |
| 84 | 10,000 | Jackknife | LQ | 0.5 | 0.00 | 0.97 | 0.96 | 0.31 | 0.02 | 0.24 | 0.12 | 0.11 | 0.01 | 0.01 |
| 84 | 10,000 | Jackknife | LQ | 0.6 | 2.05 | 0.97 | 0.96 | 0.34 | 0.02 | 0.28 | 0.12 | 0.11 | 0.01 | 0.01 |
| 84 | 10,000 | Jackknife | LQ | 1.1 | 6.16 | 0.97 | 0.96 | 0.33 | 0.02 | 0.26 | 0.14 | 0.13 | 0.01 | 0.01 |
| 84 | 10,000 | Jackknife | LQ | 1.2 | 6.68 | 0.97 | 0.96 | 0.34 | 0.02 | 0.27 | 0.14 | 0.13 | 0.01 | 0.01 |
| 84 | 10,000 | Jackknife | LQ | 0.7 | 6.87 | 0.97 | 0.96 | 0.37 | 0.02 | 0.31 | 0.12 | 0.11 | 0.01 | 0.01 |
| 84 | 10,000 | Jackknife | LQ | 1 | 7.46 | 0.97 | 0.96 | 0.32 | 0.02 | 0.26 | 0.14 | 0.13 | 0.01 | 0.01 |
| 84 | 10,000 | Jackknife | LQ | 0.8 | 7.60 | 0.97 | 0.96 | 0.33 | 0.02 | 0.27 | 0.12 | 0.11 | 0.01 | 0.01 |
| 84 | 10,000 | Jackknife | LQ | 0.9 | 9.73 | 0.97 | 0.96 | 0.34 | 0.02 | 0.28 | 0.12 | 0.11 | 0.01 | 0.01 |
| 84 | 10,000 | Jackknife | LQ | 1.5 | 10.59 | 0.97 | 0.96 | 0.32 | 0.02 | 0.25 | 0.16 | 0.13 | 0.01 | 0.01 |
| 84 | 10,000 | Jackknife | LQ | 1.3 | 11.12 | 0.97 | 0.96 | 0.32 | 0.02 | 0.25 | 0.13 | 0.12 | 0.01 | 0.01 |
| 84 | 10,000 | Jackknife | LQ | 1.4 | 14.53 | 0.97 | 0.96 | 0.35 | 0.02 | 0.28 | 0.16 | 0.13 | 0.01 | 0.01 |
| 84 | 10,000 | Jackknife | LQ | 1.6 | 17.69 | 0.97 | 0.96 | 0.31 | 0.02 | 0.24 | 0.13 | 0.12 | 0.01 | 0.01 |
| 84 | 10,000 | Jackknife | LQ | 1.7 | 21.63 | 0.97 | 0.96 | 0.31 | 0.02 | 0.24 | 0.14 | 0.13 | 0.01 | 0.01 |
| 84 | 10,000 | Jackknife | LQ | 2 | 23.54 | 0.96 | 0.96 | 0.33 | 0.02 | 0.25 | 0.13 | 0.12 | 0.01 | 0.01 |
| 84 | 10,000 | Jackknife | LQ | 1.8 | 23.88 | 0.97 | 0.96 | 0.32 | 0.02 | 0.25 | 0.12 | 0.11 | 0.01 | 0.01 |
| 84 | 10,000 | Jackknife | LQ | 1.9 | 24.38 | 0.96 | 0.96 | 0.33 | 0.02 | 0.26 | 0.14 | 0.13 | 0.01 | 0.01 |
| 84 | 10,000 | Jackknife | LQHPT | 2 | 81.86 | 0.98 | 0.96 | 0.30 | 0.03 | 0.25 | 0.19 | 0.16 | 0.04 | 0.04 |
| 84 | 10,000 | Jackknife | LQH | 2 | 87.82 | 0.96 | 0.96 | 0.41 | 0.03 | 0.32 | 0.11 | 0.10 | 0.02 | 0.02 |
| 84 | 10,000 | Jackknife | LQHP | 1.9 | 102.51 | 0.97 | 0.96 | 0.26 | 0.02 | 0.20 | 0.14 | 0.13 | 0.02 | 0.02 |
| 84 | 10,000 | Jackknife | LQHPT | 1.3 | 105.35 | 0.98 | 0.96 | 0.30 | 0.03 | 0.26 | 0.18 | 0.15 | 0.05 | 0.05 |
| 84 | 10,000 | Jackknife | LQHPT | 1.9 | 105.40 | 0.98 | 0.96 | 0.29 | 0.03 | 0.24 | 0.19 | 0.16 | 0.04 | 0.04 |
| 84 | 10,000 | Jackknife | LQHP | 2 | 106.16 | 0.97 | 0.96 | 0.27 | 0.02 | 0.21 | 0.14 | 0.13 | 0.02 | 0.02 |
| 84 | 10,000 | Jackknife | LQHPT | 1.7 | 114.75 | 0.98 | 0.96 | 0.30 | 0.03 | 0.25 | 0.19 | 0.16 | 0.04 | 0.04 |
| 84 | 10,000 | Jackknife | LQHP | 1.7 | 123.02 | 0.97 | 0.96 | 0.25 | 0.02 | 0.19 | 0.12 | 0.11 | 0.02 | 0.02 |
| 84 | 10,000 | Jackknife | LQH | 1.9 | 125.52 | 0.97 | 0.96 | 0.40 | 0.03 | 0.31 | 0.13 | 0.12 | 0.02 | 0.02 |
| 84 | 10,000 | Jackknife | LQH | 1.8 | 131.08 | 0.97 | 0.96 | 0.39 | 0.03 | 0.30 | 0.14 | 0.13 | 0.04 | 0.04 |
| 84 | 10,000 | Jackknife | LQHPT | 1.6 | 132.34 | 0.98 | 0.96 | 0.29 | 0.03 | 0.24 | 0.20 | 0.16 | 0.04 | 0.04 |
| 84 | 10,000 | Jackknife | LQHPT | 1.4 | 141.46 | 0.98 | 0.96 | 0.31 | 0.03 | 0.27 | 0.19 | 0.16 | 0.05 | 0.05 |
| 84 | 10,000 | Jackknife | LQHP | 1.8 | 145.09 | 0.97 | 0.96 | 0.25 | 0.02 | 0.20 | 0.14 | 0.13 | 0.02 | 0.02 |
| 84 | 10,000 | Jackknife | LQHP | 1.6 | 146.91 | 0.97 | 0.96 | 0.25 | 0.02 | 0.19 | 0.12 | 0.11 | 0.02 | 0.02 |
| 84 | 10,000 | Jackknife | LQH | 1.7 | 155.29 | 0.97 | 0.96 | 0.37 | 0.03 | 0.29 | 0.14 | 0.13 | 0.04 | 0.04 |
| 84 | 10,000 | Jackknife | LQH | 1.5 | 159.28 | 0.97 | 0.96 | 0.36 | 0.02 | 0.28 | 0.14 | 0.13 | 0.04 | 0.04 |
| 84 | 10,000 | Jackknife | LQHPT | 1.8 | 160.69 | 0.98 | 0.96 | 0.29 | 0.03 | 0.24 | 0.19 | 0.16 | 0.04 | 0.04 |
| 84 | 10,000 | Jackknife | LQH | 1.6 | 162.57 | 0.97 | 0.96 | 0.36 | 0.03 | 0.28 | 0.14 | 0.13 | 0.04 | 0.04 |
| 84 | 10,000 | Jackknife | LQHP | 1.5 | 166.74 | 0.97 | 0.96 | 0.25 | 0.02 | 0.20 | 0.12 | 0.11 | 0.02 | 0.02 |
| 84 | 10,000 | Jackknife | LQHPT | 1.5 | 168.19 | 0.98 | 0.96 | 0.30 | 0.03 | 0.25 | 0.20 | 0.16 | 0.04 | 0.04 |
| 84 | 10,000 | Jackknife | LQHP | 1.4 | 187.56 | 0.97 | 0.97 | 0.25 | 0.02 | 0.19 | 0.12 | 0.11 | 0.02 | 0.02 |
| 84 | 10,000 | Jackknife | LQH | 1.4 | 202.61 | 0.97 | 0.96 | 0.35 | 0.02 | 0.27 | 0.14 | 0.13 | 0.04 | 0.04 |
| 84 | 10,000 | Jackknife | LQHP | 1.2 | 217.61 | 0.97 | 0.97 | 0.23 | 0.02 | 0.19 | 0.12 | 0.11 | 0.02 | 0.02 |
| 84 | 10,000 | Jackknife | LQH | 1.3 | 225.90 | 0.97 | 0.96 | 0.32 | 0.02 | 0.25 | 0.14 | 0.13 | 0.04 | 0.04 |
| 84 | 10,000 | Jackknife | LQHPT | 1.2 | 238.57 | 0.98 | 0.96 | 0.30 | 0.03 | 0.27 | 0.17 | 0.14 | 0.05 | 0.05 |
| 84 | 10,000 | Jackknife | H | 1.3 | 242.18 | 0.97 | 0.96 | 0.32 | 0.02 | 0.24 | 0.14 | 0.13 | 0.04 | 0.04 |
| 84 | 10,000 | Jackknife | LQH | 1.2 | 289.59 | 0.97 | 0.96 | 0.31 | 0.02 | 0.24 | 0.14 | 0.13 | 0.02 | 0.02 |
| 84 | 10,000 | Jackknife | L | 0.1 | 296.89 | 0.81 | 0.81 | 2.98 | 0.09 | 1.55 | 0.11 | 0.10 | 0.00 | 0.00 |
| 84 | 10,000 | Jackknife | L | 0.2 | 296.89 | 0.81 | 0.81 | 2.98 | 0.09 | 1.55 | 0.11 | 0.10 | 0.00 | 0.00 |
| 84 | 10,000 | Jackknife | L | 0.3 | 296.89 | 0.81 | 0.81 | 2.98 | 0.09 | 1.55 | 0.11 | 0.10 | 0.00 | 0.00 |
| 84 | 10,000 | Jackknife | L | 0.4 | 296.89 | 0.81 | 0.81 | 2.98 | 0.09 | 1.55 | 0.11 | 0.10 | 0.00 | 0.00 |
| 84 | 10,000 | Jackknife | L | 0.5 | 296.89 | 0.81 | 0.81 | 2.98 | 0.09 | 1.55 | 0.11 | 0.10 | 0.00 | 0.00 |
| 84 | 10,000 | Jackknife | L | 0.6 | 296.89 | 0.81 | 0.81 | 2.98 | 0.09 | 1.55 | 0.11 | 0.10 | 0.00 | 0.00 |
| 84 | 10,000 | Jackknife | L | 0.7 | 296.96 | 0.81 | 0.81 | 2.98 | 0.09 | 1.55 | 0.11 | 0.10 | 0.00 | 0.00 |
| 84 | 10,000 | Jackknife | L | 0.8 | 297.03 | 0.81 | 0.81 | 2.97 | 0.09 | 1.55 | 0.11 | 0.10 | 0.00 | 0.00 |
| 84 | 10,000 | Jackknife | L | 0.9 | 297.13 | 0.81 | 0.81 | 2.97 | 0.09 | 1.54 | 0.11 | 0.10 | 0.00 | 0.00 |
| 84 | 10,000 | Jackknife | L | 1 | 297.24 | 0.81 | 0.81 | 2.97 | 0.09 | 1.54 | 0.11 | 0.10 | 0.00 | 0.00 |
| 84 | 10,000 | Jackknife | L | 1.1 | 297.45 | 0.81 | 0.80 | 2.97 | 0.09 | 1.54 | 0.11 | 0.10 | 0.00 | 0.00 |
| 84 | 10,000 | Jackknife | L | 1.2 | 297.58 | 0.81 | 0.80 | 2.97 | 0.09 | 1.54 | 0.11 | 0.10 | 0.00 | 0.00 |
| 84 | 10,000 | Jackknife | L | 1.3 | 297.74 | 0.81 | 0.80 | 2.97 | 0.09 | 1.54 | 0.11 | 0.10 | 0.00 | 0.00 |
| 84 | 10,000 | Jackknife | L | 1.4 | 297.96 | 0.81 | 0.80 | 2.96 | 0.09 | 1.54 | 0.11 | 0.10 | 0.00 | 0.00 |
| 84 | 10,000 | Jackknife | L | 1.5 | 298.24 | 0.81 | 0.80 | 2.95 | 0.09 | 1.53 | 0.11 | 0.10 | 0.00 | 0.00 |
| 84 | 10,000 | Jackknife | L | 1.6 | 298.51 | 0.81 | 0.80 | 2.95 | 0.09 | 1.53 | 0.11 | 0.10 | 0.00 | 0.00 |
| 84 | 10,000 | Jackknife | L | 1.7 | 298.82 | 0.81 | 0.80 | 2.94 | 0.09 | 1.52 | 0.11 | 0.10 | 0.00 | 0.00 |
| 84 | 10,000 | Jackknife | L | 1.8 | 299.13 | 0.81 | 0.80 | 2.94 | 0.09 | 1.52 | 0.11 | 0.10 | 0.00 | 0.00 |
| 84 | 10,000 | Jackknife | L | 1.9 | 299.45 | 0.81 | 0.80 | 2.93 | 0.09 | 1.52 | 0.11 | 0.10 | 0.00 | 0.00 |
| 84 | 10,000 | Jackknife | L | 2 | 299.82 | 0.81 | 0.80 | 2.92 | 0.09 | 1.51 | 0.11 | 0.10 | 0.00 | 0.00 |
| 84 | 10,000 | Jackknife | LQHP | 1.1 | 309.70 | 0.98 | 0.97 | 0.24 | 0.02 | 0.19 | 0.16 | 0.13 | 0.02 | 0.02 |
| 84 | 10,000 | Jackknife | LQHP | 1.3 | 322.27 | 0.97 | 0.97 | 0.24 | 0.02 | 0.19 | 0.12 | 0.11 | 0.02 | 0.02 |
| 84 | 10,000 | Jackknife | H | 1.4 | 369.29 | 0.97 | 0.96 | 0.33 | 0.02 | 0.26 | 0.14 | 0.13 | 0.04 | 0.04 |
| 84 | 10,000 | Jackknife | H | 1.6 | 376.06 | 0.97 | 0.96 | 0.36 | 0.03 | 0.28 | 0.14 | 0.13 | 0.04 | 0.04 |
| 84 | 10,000 | Jackknife | LQHPT | 1.1 | 380.51 | 0.98 | 0.96 | 0.33 | 0.03 | 0.29 | 0.19 | 0.16 | 0.07 | 0.07 |
| 84 | 10,000 | Jackknife | H | 1.2 | 482.42 | 0.97 | 0.96 | 0.30 | 0.02 | 0.23 | 0.14 | 0.13 | 0.04 | 0.04 |
| 84 | 10,000 | Jackknife | H | 1.5 | 497.49 | 0.97 | 0.96 | 0.35 | 0.03 | 0.27 | 0.14 | 0.13 | 0.04 | 0.04 |
| 84 | 10,000 | Jackknife | H | 1.7 | 544.34 | 0.97 | 0.96 | 0.38 | 0.03 | 0.29 | 0.13 | 0.12 | 0.04 | 0.04 |
| 84 | 10,000 | Jackknife | H | 1.9 | 594.64 | 0.96 | 0.96 | 0.40 | 0.03 | 0.31 | 0.13 | 0.12 | 0.04 | 0.04 |
| 84 | 10,000 | Jackknife | H | 2 | 598.05 | 0.96 | 0.96 | 0.41 | 0.03 | 0.32 | 0.11 | 0.10 | 0.04 | 0.04 |
| 84 | 10,000 | Jackknife | H | 1.8 | 641.48 | 0.97 | 0.96 | 0.39 | 0.03 | 0.30 | 0.13 | 0.12 | 0.04 | 0.04 |
| 84 | 10,000 | Jackknife | LQH | 1.1 | 806.82 | 0.97 | 0.96 | 0.30 | 0.02 | 0.23 | 0.16 | 0.13 | 0.02 | 0.02 |
| 84 | 10,000 | Jackknife | H | 1.1 | 998.10 | 0.97 | 0.96 | 0.29 | 0.02 | 0.23 | 0.14 | 0.13 | 0.04 | 0.04 |
| 84 | 10,000 | Jackknife | LQHP | 0.9 | 1239.71 | 0.98 | 0.97 | 0.22 | 0.02 | 0.19 | 0.16 | 0.13 | 0.02 | 0.02 |
| 84 | 10,000 | Jackknife | LQHP | 1 | 1437.07 | 0.98 | 0.97 | 0.23 | 0.02 | 0.19 | 0.14 | 0.13 | 0.02 | 0.02 |
| 84 | 10,000 | Jackknife | LQH | 1 | 1454.07 | 0.97 | 0.96 | 0.28 | 0.02 | 0.23 | 0.16 | 0.13 | 0.02 | 0.02 |
| 84 | 10,000 | Jackknife | LQHPT | 0.9 | 3043.55 | 0.99 | 0.96 | 0.43 | 0.03 | 0.40 | 0.20 | 0.16 | 0.08 | 0.08 |
| 84 | 10,000 | Jackknife | LQHPT | 1 | 3058.32 | 0.99 | 0.96 | 0.36 | 0.03 | 0.33 | 0.18 | 0.15 | 0.07 | 0.07 |
| 84 | 10,000 | Jackknife | LQH | 0.9 | 6550.96 | 0.97 | 0.96 | 0.27 | 0.02 | 0.22 | 0.14 | 0.13 | 0.02 | 0.02 |
| 84 | 10,000 | Jackknife | H | 0.1 | NA | 0.99 | 0.98 | 0.19 | 0.02 | 0.18 | 0.17 | 0.14 | 0.05 | 0.05 |
| 84 | 10,000 | Jackknife | LQH | 0.1 | NA | 0.99 | 0.98 | 0.20 | 0.02 | 0.18 | 0.17 | 0.14 | 0.04 | 0.04 |
| 84 | 10,000 | Jackknife | LQHP | 0.1 | NA | 0.99 | 0.98 | 0.21 | 0.02 | 0.19 | 0.17 | 0.14 | 0.04 | 0.04 |
| 84 | 10,000 | Jackknife | LQHPT | 0.1 | NA | 0.99 | 0.96 | 0.59 | 0.03 | 0.57 | 0.28 | 0.20 | 0.06 | 0.06 |
| 84 | 10,000 | Jackknife | H | 0.2 | NA | 0.99 | 0.97 | 0.21 | 0.02 | 0.19 | 0.17 | 0.14 | 0.04 | 0.04 |
| 84 | 10,000 | Jackknife | LQH | 0.2 | NA | 0.99 | 0.97 | 0.20 | 0.02 | 0.18 | 0.17 | 0.14 | 0.04 | 0.04 |
| 84 | 10,000 | Jackknife | LQHP | 0.2 | NA | 0.99 | 0.98 | 0.20 | 0.02 | 0.18 | 0.17 | 0.14 | 0.05 | 0.05 |
| 84 | 10,000 | Jackknife | LQHPT | 0.2 | NA | 0.99 | 0.97 | 0.44 | 0.03 | 0.42 | 0.33 | 0.22 | 0.06 | 0.06 |
| 84 | 10,000 | Jackknife | H | 0.3 | NA | 0.98 | 0.97 | 0.21 | 0.02 | 0.19 | 0.17 | 0.14 | 0.04 | 0.04 |
| 84 | 10,000 | Jackknife | LQH | 0.3 | NA | 0.98 | 0.97 | 0.21 | 0.02 | 0.19 | 0.17 | 0.14 | 0.04 | 0.04 |
| 84 | 10,000 | Jackknife | LQHP | 0.3 | NA | 0.98 | 0.97 | 0.20 | 0.02 | 0.18 | 0.16 | 0.13 | 0.02 | 0.02 |
| 84 | 10,000 | Jackknife | LQHPT | 0.3 | NA | 0.99 | 0.97 | 0.40 | 0.03 | 0.39 | 0.34 | 0.23 | 0.06 | 0.06 |
| 84 | 10,000 | Jackknife | H | 0.4 | NA | 0.98 | 0.97 | 0.21 | 0.02 | 0.18 | 0.16 | 0.13 | 0.02 | 0.02 |
| 84 | 10,000 | Jackknife | LQH | 0.4 | NA | 0.98 | 0.97 | 0.21 | 0.02 | 0.18 | 0.17 | 0.14 | 0.04 | 0.04 |
| 84 | 10,000 | Jackknife | LQHP | 0.4 | NA | 0.98 | 0.97 | 0.18 | 0.02 | 0.16 | 0.18 | 0.15 | 0.04 | 0.04 |
| 84 | 10,000 | Jackknife | LQHPT | 0.4 | NA | 0.99 | 0.97 | 0.40 | 0.03 | 0.39 | 0.34 | 0.23 | 0.05 | 0.05 |
| 84 | 10,000 | Jackknife | H | 0.5 | NA | 0.98 | 0.97 | 0.22 | 0.02 | 0.19 | 0.14 | 0.13 | 0.02 | 0.02 |
| 84 | 10,000 | Jackknife | LQH | 0.5 | NA | 0.98 | 0.97 | 0.23 | 0.02 | 0.19 | 0.14 | 0.13 | 0.02 | 0.02 |
| 84 | 10,000 | Jackknife | LQHP | 0.5 | NA | 0.98 | 0.97 | 0.19 | 0.02 | 0.17 | 0.17 | 0.14 | 0.04 | 0.04 |
| 84 | 10,000 | Jackknife | LQHPT | 0.5 | NA | 0.99 | 0.97 | 0.48 | 0.03 | 0.46 | 0.28 | 0.20 | 0.05 | 0.05 |
| 84 | 10,000 | Jackknife | H | 0.6 | NA | 0.98 | 0.97 | 0.24 | 0.02 | 0.20 | 0.14 | 0.13 | 0.01 | 0.01 |
| 84 | 10,000 | Jackknife | LQH | 0.6 | NA | 0.98 | 0.97 | 0.26 | 0.02 | 0.22 | 0.14 | 0.13 | 0.02 | 0.02 |
| 84 | 10,000 | Jackknife | LQHP | 0.6 | NA | 0.98 | 0.97 | 0.21 | 0.02 | 0.18 | 0.17 | 0.14 | 0.02 | 0.02 |
| 84 | 10,000 | Jackknife | LQHPT | 0.6 | NA | 0.99 | 0.97 | 0.49 | 0.03 | 0.48 | 0.25 | 0.19 | 0.05 | 0.05 |
| 84 | 10,000 | Jackknife | H | 0.7 | NA | 0.98 | 0.97 | 0.24 | 0.02 | 0.20 | 0.12 | 0.11 | 0.04 | 0.04 |
| 84 | 10,000 | Jackknife | LQH | 0.7 | NA | 0.98 | 0.97 | 0.26 | 0.02 | 0.22 | 0.13 | 0.12 | 0.02 | 0.02 |
| 84 | 10,000 | Jackknife | LQHP | 0.7 | NA | 0.98 | 0.97 | 0.21 | 0.02 | 0.18 | 0.18 | 0.15 | 0.02 | 0.02 |
| 84 | 10,000 | Jackknife | LQHPT | 0.7 | NA | 0.99 | 0.96 | 0.49 | 0.03 | 0.47 | 0.22 | 0.17 | 0.06 | 0.06 |
| 84 | 10,000 | Jackknife | H | 0.8 | NA | 0.98 | 0.97 | 0.25 | 0.02 | 0.20 | 0.12 | 0.11 | 0.04 | 0.04 |
| 84 | 10,000 | Jackknife | LQH | 0.8 | NA | 0.97 | 0.96 | 0.27 | 0.02 | 0.22 | 0.16 | 0.13 | 0.02 | 0.02 |
| 84 | 10,000 | Jackknife | LQHP | 0.8 | NA | 0.98 | 0.97 | 0.21 | 0.02 | 0.17 | 0.18 | 0.15 | 0.04 | 0.04 |
| 84 | 10,000 | Jackknife | LQHPT | 0.8 | NA | 0.99 | 0.96 | 0.46 | 0.03 | 0.44 | 0.22 | 0.17 | 0.08 | 0.08 |
| 84 | 10,000 | Jackknife | H | 0.9 | NA | 0.97 | 0.96 | 0.27 | 0.02 | 0.22 | 0.14 | 0.13 | 0.04 | 0.04 |
| 84 | 10,000 | Jackknife | H | 1 | NA | 0.97 | 0.96 | 0.28 | 0.02 | 0.22 | 0.14 | 0.13 | 0.04 | 0.04 |
| 84 | 10,000 | Random-k-fold | LQ | 0.1 | 0.00 | 0.97 | 0.96 | 0.00 | 0.01 | 0.00 | 0.12 | 0.00 | 0.01 | 0.00 |
| 84 | 10,000 | Random-k-fold | LQ | 0.2 | 0.00 | 0.97 | 0.96 | 0.00 | 0.01 | 0.00 | 0.12 | 0.00 | 0.01 | 0.00 |
| 84 | 10,000 | Random-k-fold | LQ | 0.3 | 0.00 | 0.97 | 0.96 | 0.00 | 0.01 | 0.00 | 0.12 | 0.00 | 0.01 | 0.00 |
| 84 | 10,000 | Random-k-fold | LQ | 0.4 | 0.00 | 0.97 | 0.96 | 0.00 | 0.01 | 0.00 | 0.12 | 0.00 | 0.01 | 0.00 |
| 84 | 10,000 | Random-k-fold | LQ | 0.5 | 0.00 | 0.97 | 0.96 | 0.00 | 0.01 | 0.00 | 0.12 | 0.00 | 0.01 | 0.00 |
| 84 | 10,000 | Random-k-fold | LQ | 0.6 | 2.05 | 0.97 | 0.96 | 0.00 | 0.01 | 0.00 | 0.13 | 0.00 | 0.01 | 0.00 |
| 84 | 10,000 | Random-k-fold | LQ | 1.1 | 6.16 | 0.97 | 0.96 | 0.00 | 0.01 | 0.00 | 0.12 | 0.00 | 0.01 | 0.00 |
| 84 | 10,000 | Random-k-fold | LQ | 1.2 | 6.68 | 0.97 | 0.96 | 0.00 | 0.01 | 0.00 | 0.11 | 0.00 | 0.01 | 0.00 |
| 84 | 10,000 | Random-k-fold | LQ | 0.7 | 6.87 | 0.97 | 0.96 | 0.00 | 0.01 | 0.00 | 0.12 | 0.00 | 0.01 | 0.00 |
| 84 | 10,000 | Random-k-fold | LQ | 1 | 7.46 | 0.97 | 0.96 | 0.00 | 0.01 | 0.00 | 0.12 | 0.00 | 0.01 | 0.00 |
| 84 | 10,000 | Random-k-fold | LQ | 0.8 | 7.60 | 0.97 | 0.96 | 0.00 | 0.01 | 0.00 | 0.12 | 0.00 | 0.01 | 0.00 |
| 84 | 10,000 | Random-k-fold | LQ | 0.9 | 9.73 | 0.97 | 0.96 | 0.00 | 0.01 | 0.00 | 0.12 | 0.00 | 0.01 | 0.00 |
| 84 | 10,000 | Random-k-fold | LQ | 1.5 | 10.59 | 0.97 | 0.96 | 0.00 | 0.01 | 0.00 | 0.11 | 0.00 | 0.01 | 0.00 |
| 84 | 10,000 | Random-k-fold | LQ | 1.3 | 11.12 | 0.97 | 0.96 | 0.00 | 0.01 | 0.00 | 0.11 | 0.00 | 0.01 | 0.00 |
| 84 | 10,000 | Random-k-fold | LQ | 1.4 | 14.53 | 0.97 | 0.96 | 0.00 | 0.01 | 0.00 | 0.11 | 0.00 | 0.01 | 0.00 |
| 84 | 10,000 | Random-k-fold | LQ | 1.6 | 17.69 | 0.97 | 0.95 | 0.00 | 0.01 | 0.00 | 0.11 | 0.00 | 0.01 | 0.00 |
| 84 | 10,000 | Random-k-fold | LQ | 1.7 | 21.63 | 0.97 | 0.95 | 0.00 | 0.01 | 0.00 | 0.11 | 0.00 | 0.01 | 0.00 |
| 84 | 10,000 | Random-k-fold | LQ | 2 | 23.54 | 0.96 | 0.95 | 0.00 | 0.01 | 0.00 | 0.10 | 0.00 | 0.01 | 0.00 |
| 84 | 10,000 | Random-k-fold | LQ | 1.8 | 23.88 | 0.97 | 0.95 | 0.00 | 0.01 | 0.00 | 0.12 | 0.00 | 0.01 | 0.00 |
| 84 | 10,000 | Random-k-fold | LQ | 1.9 | 24.38 | 0.96 | 0.95 | 0.00 | 0.01 | 0.00 | 0.11 | 0.00 | 0.01 | 0.00 |
| 84 | 10,000 | Random-k-fold | LQHPT | 2 | 81.86 | 0.98 | 0.96 | 0.00 | 0.02 | 0.00 | 0.20 | 0.01 | 0.02 | 0.00 |
| 84 | 10,000 | Random-k-fold | LQH | 2 | 87.82 | 0.96 | 0.95 | 0.00 | 0.01 | 0.00 | 0.12 | 0.00 | 0.02 | 0.00 |
| 84 | 10,000 | Random-k-fold | LQHP | 1.9 | 102.51 | 0.97 | 0.96 | 0.00 | 0.01 | 0.00 | 0.13 | 0.00 | 0.04 | 0.00 |
| 84 | 10,000 | Random-k-fold | LQHPT | 1.3 | 105.35 | 0.98 | 0.96 | 0.00 | 0.02 | 0.00 | 0.23 | 0.02 | 0.06 | 0.00 |
| 84 | 10,000 | Random-k-fold | LQHPT | 1.9 | 105.40 | 0.98 | 0.96 | 0.00 | 0.02 | 0.00 | 0.19 | 0.01 | 0.01 | 0.00 |
| 84 | 10,000 | Random-k-fold | LQHP | 2 | 106.16 | 0.97 | 0.96 | 0.00 | 0.01 | 0.00 | 0.14 | 0.00 | 0.04 | 0.00 |
| 84 | 10,000 | Random-k-fold | LQHPT | 1.7 | 114.75 | 0.98 | 0.96 | 0.00 | 0.02 | 0.00 | 0.22 | 0.01 | 0.05 | 0.00 |
| 84 | 10,000 | Random-k-fold | LQHP | 1.7 | 123.02 | 0.97 | 0.96 | 0.00 | 0.01 | 0.00 | 0.14 | 0.00 | 0.04 | 0.00 |
| 84 | 10,000 | Random-k-fold | LQH | 1.9 | 125.52 | 0.97 | 0.96 | 0.00 | 0.01 | 0.00 | 0.12 | 0.00 | 0.04 | 0.00 |
| 84 | 10,000 | Random-k-fold | LQH | 1.8 | 131.08 | 0.97 | 0.96 | 0.00 | 0.01 | 0.00 | 0.12 | 0.00 | 0.04 | 0.00 |
| 84 | 10,000 | Random-k-fold | LQHPT | 1.6 | 132.34 | 0.98 | 0.96 | 0.00 | 0.02 | 0.00 | 0.19 | 0.01 | 0.05 | 0.00 |
| 84 | 10,000 | Random-k-fold | LQHPT | 1.4 | 141.46 | 0.98 | 0.96 | 0.00 | 0.02 | 0.00 | 0.22 | 0.01 | 0.06 | 0.00 |
| 84 | 10,000 | Random-k-fold | LQHP | 1.8 | 145.09 | 0.97 | 0.96 | 0.00 | 0.01 | 0.00 | 0.14 | 0.00 | 0.04 | 0.00 |
| 84 | 10,000 | Random-k-fold | LQHP | 1.6 | 146.91 | 0.97 | 0.96 | 0.00 | 0.01 | 0.00 | 0.14 | 0.00 | 0.04 | 0.00 |
| 84 | 10,000 | Random-k-fold | LQH | 1.7 | 155.29 | 0.97 | 0.96 | 0.00 | 0.01 | 0.00 | 0.12 | 0.00 | 0.04 | 0.00 |
| 84 | 10,000 | Random-k-fold | LQH | 1.5 | 159.28 | 0.97 | 0.96 | 0.00 | 0.01 | 0.00 | 0.13 | 0.00 | 0.04 | 0.00 |
| 84 | 10,000 | Random-k-fold | LQHPT | 1.8 | 160.69 | 0.98 | 0.96 | 0.00 | 0.02 | 0.00 | 0.20 | 0.01 | 0.02 | 0.00 |
| 84 | 10,000 | Random-k-fold | LQH | 1.6 | 162.57 | 0.97 | 0.96 | 0.00 | 0.01 | 0.00 | 0.12 | 0.00 | 0.02 | 0.00 |
| 84 | 10,000 | Random-k-fold | LQHP | 1.5 | 166.74 | 0.97 | 0.96 | 0.00 | 0.01 | 0.00 | 0.14 | 0.00 | 0.04 | 0.00 |
| 84 | 10,000 | Random-k-fold | LQHPT | 1.5 | 168.19 | 0.98 | 0.96 | 0.00 | 0.02 | 0.00 | 0.20 | 0.01 | 0.07 | 0.00 |
| 84 | 10,000 | Random-k-fold | LQHP | 1.4 | 187.56 | 0.97 | 0.96 | 0.00 | 0.01 | 0.00 | 0.14 | 0.00 | 0.04 | 0.00 |
| 84 | 10,000 | Random-k-fold | LQH | 1.4 | 202.61 | 0.97 | 0.96 | 0.00 | 0.01 | 0.00 | 0.13 | 0.00 | 0.04 | 0.00 |
| 84 | 10,000 | Random-k-fold | LQHP | 1.2 | 217.61 | 0.97 | 0.96 | 0.00 | 0.01 | 0.00 | 0.13 | 0.00 | 0.02 | 0.00 |
| 84 | 10,000 | Random-k-fold | LQH | 1.3 | 225.90 | 0.97 | 0.96 | 0.00 | 0.01 | 0.00 | 0.13 | 0.00 | 0.04 | 0.00 |
| 84 | 10,000 | Random-k-fold | LQHPT | 1.2 | 238.57 | 0.98 | 0.96 | 0.00 | 0.02 | 0.00 | 0.23 | 0.02 | 0.06 | 0.00 |
| 84 | 10,000 | Random-k-fold | H | 1.3 | 242.18 | 0.97 | 0.96 | 0.00 | 0.01 | 0.00 | 0.12 | 0.00 | 0.04 | 0.00 |
| 84 | 10,000 | Random-k-fold | LQH | 1.2 | 289.59 | 0.97 | 0.96 | 0.00 | 0.01 | 0.00 | 0.13 | 0.00 | 0.04 | 0.00 |
| 84 | 10,000 | Random-k-fold | L | 0.1 | 296.89 | 0.81 | 0.81 | 0.01 | 0.03 | 0.01 | 0.13 | 0.01 | 0.00 | 0.00 |
| 84 | 10,000 | Random-k-fold | L | 0.2 | 296.89 | 0.81 | 0.81 | 0.01 | 0.03 | 0.01 | 0.13 | 0.01 | 0.00 | 0.00 |
| 84 | 10,000 | Random-k-fold | L | 0.3 | 296.89 | 0.81 | 0.81 | 0.01 | 0.03 | 0.01 | 0.13 | 0.01 | 0.00 | 0.00 |
| 84 | 10,000 | Random-k-fold | L | 0.4 | 296.89 | 0.81 | 0.81 | 0.01 | 0.03 | 0.01 | 0.13 | 0.01 | 0.00 | 0.00 |
| 84 | 10,000 | Random-k-fold | L | 0.5 | 296.89 | 0.81 | 0.80 | 0.01 | 0.03 | 0.01 | 0.13 | 0.01 | 0.00 | 0.00 |
| 84 | 10,000 | Random-k-fold | L | 0.6 | 296.89 | 0.81 | 0.80 | 0.01 | 0.03 | 0.01 | 0.13 | 0.01 | 0.00 | 0.00 |
| 84 | 10,000 | Random-k-fold | L | 0.7 | 296.96 | 0.81 | 0.80 | 0.01 | 0.03 | 0.01 | 0.13 | 0.01 | 0.00 | 0.00 |
| 84 | 10,000 | Random-k-fold | L | 0.8 | 297.03 | 0.81 | 0.80 | 0.01 | 0.03 | 0.01 | 0.13 | 0.01 | 0.00 | 0.00 |
| 84 | 10,000 | Random-k-fold | L | 0.9 | 297.13 | 0.81 | 0.80 | 0.01 | 0.03 | 0.01 | 0.13 | 0.01 | 0.00 | 0.00 |
| 84 | 10,000 | Random-k-fold | L | 1 | 297.24 | 0.81 | 0.80 | 0.01 | 0.03 | 0.01 | 0.13 | 0.01 | 0.00 | 0.00 |
| 84 | 10,000 | Random-k-fold | L | 1.1 | 297.45 | 0.81 | 0.80 | 0.01 | 0.03 | 0.01 | 0.13 | 0.01 | 0.00 | 0.00 |
| 84 | 10,000 | Random-k-fold | L | 1.2 | 297.58 | 0.81 | 0.80 | 0.01 | 0.03 | 0.01 | 0.13 | 0.01 | 0.00 | 0.00 |
| 84 | 10,000 | Random-k-fold | L | 1.3 | 297.74 | 0.81 | 0.80 | 0.01 | 0.03 | 0.01 | 0.13 | 0.01 | 0.00 | 0.00 |
| 84 | 10,000 | Random-k-fold | L | 1.4 | 297.96 | 0.81 | 0.80 | 0.01 | 0.03 | 0.01 | 0.13 | 0.01 | 0.00 | 0.00 |
| 84 | 10,000 | Random-k-fold | L | 1.5 | 298.24 | 0.81 | 0.80 | 0.01 | 0.03 | 0.01 | 0.16 | 0.01 | 0.00 | 0.00 |
| 84 | 10,000 | Random-k-fold | L | 1.6 | 298.51 | 0.81 | 0.80 | 0.01 | 0.03 | 0.01 | 0.16 | 0.01 | 0.00 | 0.00 |
| 84 | 10,000 | Random-k-fold | L | 1.7 | 298.82 | 0.81 | 0.80 | 0.01 | 0.03 | 0.00 | 0.16 | 0.01 | 0.00 | 0.00 |
| 84 | 10,000 | Random-k-fold | L | 1.8 | 299.13 | 0.81 | 0.80 | 0.01 | 0.03 | 0.00 | 0.16 | 0.01 | 0.00 | 0.00 |
| 84 | 10,000 | Random-k-fold | L | 1.9 | 299.45 | 0.81 | 0.79 | 0.01 | 0.03 | 0.00 | 0.14 | 0.00 | 0.00 | 0.00 |
| 84 | 10,000 | Random-k-fold | L | 2 | 299.82 | 0.81 | 0.79 | 0.01 | 0.03 | 0.00 | 0.14 | 0.00 | 0.00 | 0.00 |
| 84 | 10,000 | Random-k-fold | LQHP | 1.1 | 309.70 | 0.98 | 0.96 | 0.00 | 0.01 | 0.00 | 0.12 | 0.00 | 0.02 | 0.00 |
| 84 | 10,000 | Random-k-fold | LQHP | 1.3 | 322.27 | 0.97 | 0.96 | 0.00 | 0.01 | 0.00 | 0.13 | 0.00 | 0.04 | 0.00 |
| 84 | 10,000 | Random-k-fold | H | 1.4 | 369.29 | 0.97 | 0.96 | 0.00 | 0.01 | 0.00 | 0.12 | 0.00 | 0.04 | 0.00 |
| 84 | 10,000 | Random-k-fold | H | 1.6 | 376.06 | 0.97 | 0.96 | 0.00 | 0.01 | 0.00 | 0.13 | 0.00 | 0.04 | 0.00 |
| 84 | 10,000 | Random-k-fold | LQHPT | 1.1 | 380.51 | 0.98 | 0.96 | 0.00 | 0.02 | 0.00 | 0.24 | 0.01 | 0.08 | 0.00 |
| 84 | 10,000 | Random-k-fold | H | 1.2 | 482.42 | 0.97 | 0.96 | 0.00 | 0.01 | 0.00 | 0.12 | 0.00 | 0.04 | 0.00 |
| 84 | 10,000 | Random-k-fold | H | 1.5 | 497.49 | 0.97 | 0.96 | 0.00 | 0.01 | 0.00 | 0.13 | 0.00 | 0.04 | 0.00 |
| 84 | 10,000 | Random-k-fold | H | 1.7 | 544.34 | 0.97 | 0.96 | 0.00 | 0.01 | 0.00 | 0.13 | 0.00 | 0.04 | 0.00 |
| 84 | 10,000 | Random-k-fold | H | 1.9 | 594.64 | 0.96 | 0.96 | 0.00 | 0.01 | 0.00 | 0.13 | 0.00 | 0.04 | 0.00 |
| 84 | 10,000 | Random-k-fold | H | 2 | 598.05 | 0.96 | 0.96 | 0.00 | 0.01 | 0.00 | 0.13 | 0.00 | 0.04 | 0.00 |
| 84 | 10,000 | Random-k-fold | H | 1.8 | 641.48 | 0.97 | 0.96 | 0.00 | 0.01 | 0.00 | 0.13 | 0.00 | 0.04 | 0.00 |
| 84 | 10,000 | Random-k-fold | LQH | 1.1 | 806.82 | 0.97 | 0.96 | 0.00 | 0.01 | 0.00 | 0.13 | 0.00 | 0.04 | 0.00 |
| 84 | 10,000 | Random-k-fold | H | 1.1 | 998.10 | 0.97 | 0.96 | 0.00 | 0.01 | 0.00 | 0.12 | 0.00 | 0.04 | 0.00 |
| 84 | 10,000 | Random-k-fold | LQHP | 0.9 | 1239.71 | 0.98 | 0.97 | 0.00 | 0.01 | 0.00 | 0.13 | 0.00 | 0.01 | 0.00 |
| 84 | 10,000 | Random-k-fold | LQHP | 1 | 1437.07 | 0.98 | 0.97 | 0.00 | 0.01 | 0.00 | 0.12 | 0.00 | 0.01 | 0.00 |
| 84 | 10,000 | Random-k-fold | LQH | 1 | 1454.07 | 0.97 | 0.96 | 0.00 | 0.01 | 0.00 | 0.13 | 0.00 | 0.04 | 0.00 |
| 84 | 10,000 | Random-k-fold | LQHPT | 0.9 | 3043.55 | 0.99 | 0.96 | 0.00 | 0.02 | 0.00 | 0.22 | 0.01 | 0.10 | 0.01 |
| 84 | 10,000 | Random-k-fold | LQHPT | 1 | 3058.32 | 0.99 | 0.96 | 0.00 | 0.02 | 0.00 | 0.23 | 0.02 | 0.08 | 0.00 |
| 84 | 10,000 | Random-k-fold | LQH | 0.9 | 6550.96 | 0.97 | 0.96 | 0.00 | 0.01 | 0.00 | 0.12 | 0.00 | 0.02 | 0.00 |
| 84 | 10,000 | Random-k-fold | H | 0.1 | NA | 0.99 | 0.97 | 0.00 | 0.02 | 0.00 | 0.18 | 0.02 | 0.08 | 0.01 |
| 84 | 10,000 | Random-k-fold | LQH | 0.1 | NA | 0.99 | 0.97 | 0.00 | 0.02 | 0.00 | 0.18 | 0.02 | 0.08 | 0.01 |
| 84 | 10,000 | Random-k-fold | LQHP | 0.1 | NA | 0.99 | 0.97 | 0.00 | 0.02 | 0.00 | 0.19 | 0.03 | 0.08 | 0.01 |
| 84 | 10,000 | Random-k-fold | LQHPT | 0.1 | NA | 0.99 | 0.95 | 0.00 | 0.05 | 0.00 | 0.49 | 0.01 | 0.17 | 0.01 |
| 84 | 10,000 | Random-k-fold | H | 0.2 | NA | 0.99 | 0.97 | 0.00 | 0.01 | 0.00 | 0.18 | 0.01 | 0.07 | 0.00 |
| 84 | 10,000 | Random-k-fold | LQH | 0.2 | NA | 0.99 | 0.97 | 0.00 | 0.01 | 0.00 | 0.18 | 0.01 | 0.07 | 0.00 |
| 84 | 10,000 | Random-k-fold | LQHP | 0.2 | NA | 0.99 | 0.97 | 0.00 | 0.01 | 0.00 | 0.17 | 0.01 | 0.06 | 0.00 |
| 84 | 10,000 | Random-k-fold | LQHPT | 0.2 | NA | 0.99 | 0.96 | 0.00 | 0.04 | 0.00 | 0.48 | 0.02 | 0.13 | 0.01 |
| 84 | 10,000 | Random-k-fold | H | 0.3 | NA | 0.98 | 0.97 | 0.00 | 0.01 | 0.00 | 0.18 | 0.01 | 0.07 | 0.00 |
| 84 | 10,000 | Random-k-fold | LQH | 0.3 | NA | 0.98 | 0.97 | 0.00 | 0.01 | 0.00 | 0.18 | 0.01 | 0.07 | 0.00 |
| 84 | 10,000 | Random-k-fold | LQHP | 0.3 | NA | 0.98 | 0.97 | 0.00 | 0.01 | 0.00 | 0.17 | 0.01 | 0.04 | 0.00 |
| 84 | 10,000 | Random-k-fold | LQHPT | 0.3 | NA | 0.99 | 0.96 | 0.00 | 0.04 | 0.00 | 0.48 | 0.02 | 0.13 | 0.01 |
| 84 | 10,000 | Random-k-fold | H | 0.4 | NA | 0.98 | 0.97 | 0.00 | 0.01 | 0.00 | 0.18 | 0.01 | 0.04 | 0.00 |
| 84 | 10,000 | Random-k-fold | LQH | 0.4 | NA | 0.98 | 0.97 | 0.00 | 0.01 | 0.00 | 0.18 | 0.01 | 0.05 | 0.00 |
| 84 | 10,000 | Random-k-fold | LQHP | 0.4 | NA | 0.98 | 0.97 | 0.00 | 0.01 | 0.00 | 0.19 | 0.00 | 0.04 | 0.00 |
| 84 | 10,000 | Random-k-fold | LQHPT | 0.4 | NA | 0.99 | 0.96 | 0.00 | 0.04 | 0.00 | 0.46 | 0.02 | 0.12 | 0.01 |
| 84 | 10,000 | Random-k-fold | H | 0.5 | NA | 0.98 | 0.97 | 0.00 | 0.01 | 0.00 | 0.17 | 0.01 | 0.05 | 0.00 |
| 84 | 10,000 | Random-k-fold | LQH | 0.5 | NA | 0.98 | 0.97 | 0.00 | 0.01 | 0.00 | 0.18 | 0.00 | 0.04 | 0.00 |
| 84 | 10,000 | Random-k-fold | LQHP | 0.5 | NA | 0.98 | 0.97 | 0.00 | 0.01 | 0.00 | 0.20 | 0.00 | 0.02 | 0.00 |
| 84 | 10,000 | Random-k-fold | LQHPT | 0.5 | NA | 0.99 | 0.96 | 0.00 | 0.03 | 0.00 | 0.38 | 0.03 | 0.10 | 0.01 |
| 84 | 10,000 | Random-k-fold | H | 0.6 | NA | 0.98 | 0.97 | 0.00 | 0.01 | 0.00 | 0.16 | 0.00 | 0.02 | 0.00 |
| 84 | 10,000 | Random-k-fold | LQH | 0.6 | NA | 0.98 | 0.97 | 0.00 | 0.01 | 0.00 | 0.16 | 0.00 | 0.02 | 0.00 |
| 84 | 10,000 | Random-k-fold | LQHP | 0.6 | NA | 0.98 | 0.97 | 0.00 | 0.01 | 0.00 | 0.20 | 0.00 | 0.01 | 0.00 |
| 84 | 10,000 | Random-k-fold | LQHPT | 0.6 | NA | 0.99 | 0.96 | 0.00 | 0.03 | 0.00 | 0.35 | 0.03 | 0.10 | 0.01 |
| 84 | 10,000 | Random-k-fold | H | 0.7 | NA | 0.98 | 0.97 | 0.00 | 0.01 | 0.00 | 0.11 | 0.00 | 0.02 | 0.00 |
| 84 | 10,000 | Random-k-fold | LQH | 0.7 | NA | 0.98 | 0.97 | 0.00 | 0.01 | 0.00 | 0.12 | 0.00 | 0.02 | 0.00 |
| 84 | 10,000 | Random-k-fold | LQHP | 0.7 | NA | 0.98 | 0.97 | 0.00 | 0.01 | 0.00 | 0.22 | 0.01 | 0.01 | 0.00 |
| 84 | 10,000 | Random-k-fold | LQHPT | 0.7 | NA | 0.99 | 0.96 | 0.00 | 0.03 | 0.00 | 0.26 | 0.02 | 0.10 | 0.01 |
| 84 | 10,000 | Random-k-fold | H | 0.8 | NA | 0.98 | 0.96 | 0.00 | 0.01 | 0.00 | 0.11 | 0.00 | 0.02 | 0.00 |
| 84 | 10,000 | Random-k-fold | LQH | 0.8 | NA | 0.97 | 0.96 | 0.00 | 0.01 | 0.00 | 0.12 | 0.00 | 0.02 | 0.00 |
| 84 | 10,000 | Random-k-fold | LQHP | 0.8 | NA | 0.98 | 0.97 | 0.00 | 0.01 | 0.00 | 0.16 | 0.01 | 0.01 | 0.00 |
| 84 | 10,000 | Random-k-fold | LQHPT | 0.8 | NA | 0.99 | 0.96 | 0.00 | 0.02 | 0.00 | 0.23 | 0.01 | 0.10 | 0.01 |
| 84 | 10,000 | Random-k-fold | H | 0.9 | NA | 0.97 | 0.96 | 0.00 | 0.01 | 0.00 | 0.12 | 0.00 | 0.02 | 0.00 |
| 84 | 10,000 | Random-k-fold | H | 1 | NA | 0.97 | 0.96 | 0.00 | 0.01 | 0.00 | 0.12 | 0.00 | 0.04 | 0.00 |
| 84 | 10,000 | Block | LQ | 0.1 | 0.00 | 0.97 | 0.95 | 0.00 | 0.02 | 0.00 | 0.15 | 0.01 | 0.02 | 0.00 |
| 84 | 10,000 | Block | LQ | 0.2 | 0.00 | 0.97 | 0.95 | 0.00 | 0.02 | 0.00 | 0.15 | 0.01 | 0.02 | 0.00 |
| 84 | 10,000 | Block | LQ | 0.3 | 0.00 | 0.97 | 0.95 | 0.00 | 0.02 | 0.00 | 0.16 | 0.01 | 0.01 | 0.00 |
| 84 | 10,000 | Block | LQ | 0.4 | 0.00 | 0.97 | 0.95 | 0.00 | 0.02 | 0.00 | 0.13 | 0.01 | 0.04 | 0.00 |
| 84 | 10,000 | Block | LQ | 0.5 | 0.00 | 0.97 | 0.95 | 0.00 | 0.02 | 0.00 | 0.15 | 0.01 | 0.02 | 0.00 |
| 84 | 10,000 | Block | LQ | 0.6 | 2.05 | 0.97 | 0.94 | 0.00 | 0.03 | 0.00 | 0.15 | 0.02 | 0.05 | 0.00 |
| 84 | 10,000 | Block | LQ | 1.1 | 6.16 | 0.97 | 0.94 | 0.00 | 0.03 | 0.00 | 0.13 | 0.01 | 0.05 | 0.00 |
| 84 | 10,000 | Block | LQ | 1.2 | 6.68 | 0.97 | 0.94 | 0.00 | 0.03 | 0.00 | 0.13 | 0.01 | 0.04 | 0.00 |
| 84 | 10,000 | Block | LQ | 0.7 | 6.87 | 0.97 | 0.94 | 0.00 | 0.03 | 0.00 | 0.13 | 0.01 | 0.05 | 0.00 |
| 84 | 10,000 | Block | LQ | 1 | 7.46 | 0.97 | 0.94 | 0.00 | 0.03 | 0.00 | 0.15 | 0.01 | 0.05 | 0.00 |
| 84 | 10,000 | Block | LQ | 0.8 | 7.60 | 0.97 | 0.94 | 0.00 | 0.03 | 0.00 | 0.15 | 0.01 | 0.05 | 0.00 |
| 84 | 10,000 | Block | LQ | 0.9 | 9.73 | 0.97 | 0.95 | 0.00 | 0.02 | 0.00 | 0.13 | 0.01 | 0.05 | 0.00 |
| 84 | 10,000 | Block | LQ | 1.5 | 10.59 | 0.97 | 0.94 | 0.01 | 0.03 | 0.00 | 0.16 | 0.02 | 0.04 | 0.00 |
| 84 | 10,000 | Block | LQ | 1.3 | 11.12 | 0.97 | 0.94 | 0.00 | 0.03 | 0.00 | 0.13 | 0.01 | 0.04 | 0.00 |
| 84 | 10,000 | Block | LQ | 1.4 | 14.53 | 0.97 | 0.94 | 0.01 | 0.03 | 0.00 | 0.16 | 0.02 | 0.04 | 0.00 |
| 84 | 10,000 | Block | LQ | 1.6 | 17.69 | 0.97 | 0.94 | 0.01 | 0.03 | 0.00 | 0.17 | 0.03 | 0.04 | 0.00 |
| 84 | 10,000 | Block | LQ | 1.7 | 21.63 | 0.97 | 0.94 | 0.01 | 0.03 | 0.00 | 0.18 | 0.03 | 0.04 | 0.00 |
| 84 | 10,000 | Block | LQ | 2 | 23.54 | 0.96 | 0.93 | 0.01 | 0.03 | 0.00 | 0.19 | 0.05 | 0.05 | 0.00 |
| 84 | 10,000 | Block | LQ | 1.8 | 23.88 | 0.97 | 0.94 | 0.01 | 0.03 | 0.00 | 0.19 | 0.04 | 0.04 | 0.00 |
| 84 | 10,000 | Block | LQ | 1.9 | 24.38 | 0.96 | 0.93 | 0.01 | 0.03 | 0.00 | 0.18 | 0.04 | 0.05 | 0.00 |
| 84 | 10,000 | Block | LQHPT | 2 | 81.86 | 0.98 | 0.93 | 0.01 | 0.05 | 0.01 | 0.37 | 0.12 | 0.06 | 0.01 |
| 84 | 10,000 | Block | LQH | 2 | 87.82 | 0.96 | 0.94 | 0.01 | 0.03 | 0.00 | 0.19 | 0.02 | 0.05 | 0.01 |
| 84 | 10,000 | Block | LQHP | 1.9 | 102.51 | 0.97 | 0.94 | 0.01 | 0.04 | 0.00 | 0.18 | 0.02 | 0.05 | 0.01 |
| 84 | 10,000 | Block | LQHPT | 1.3 | 105.35 | 0.98 | 0.93 | 0.01 | 0.05 | 0.01 | 0.45 | 0.16 | 0.13 | 0.01 |
| 84 | 10,000 | Block | LQHPT | 1.9 | 105.40 | 0.98 | 0.93 | 0.01 | 0.05 | 0.01 | 0.39 | 0.13 | 0.06 | 0.01 |
| 84 | 10,000 | Block | LQHP | 2 | 106.16 | 0.97 | 0.93 | 0.01 | 0.04 | 0.00 | 0.18 | 0.02 | 0.05 | 0.01 |
| 84 | 10,000 | Block | LQHPT | 1.7 | 114.75 | 0.98 | 0.93 | 0.01 | 0.05 | 0.01 | 0.43 | 0.12 | 0.07 | 0.01 |
| 84 | 10,000 | Block | LQHP | 1.7 | 123.02 | 0.97 | 0.94 | 0.01 | 0.04 | 0.00 | 0.19 | 0.02 | 0.05 | 0.01 |
| 84 | 10,000 | Block | LQH | 1.9 | 125.52 | 0.97 | 0.94 | 0.01 | 0.03 | 0.00 | 0.19 | 0.02 | 0.05 | 0.01 |
| 84 | 10,000 | Block | LQH | 1.8 | 131.08 | 0.97 | 0.94 | 0.01 | 0.03 | 0.00 | 0.18 | 0.02 | 0.05 | 0.01 |
| 84 | 10,000 | Block | LQHPT | 1.6 | 132.34 | 0.98 | 0.93 | 0.01 | 0.05 | 0.01 | 0.45 | 0.13 | 0.07 | 0.01 |
| 84 | 10,000 | Block | LQHPT | 1.4 | 141.46 | 0.98 | 0.93 | 0.01 | 0.06 | 0.01 | 0.44 | 0.15 | 0.13 | 0.01 |
| 84 | 10,000 | Block | LQHP | 1.8 | 145.09 | 0.97 | 0.94 | 0.01 | 0.04 | 0.00 | 0.18 | 0.02 | 0.05 | 0.01 |
| 84 | 10,000 | Block | LQHP | 1.6 | 146.91 | 0.97 | 0.94 | 0.01 | 0.04 | 0.00 | 0.21 | 0.03 | 0.05 | 0.01 |
| 84 | 10,000 | Block | LQH | 1.7 | 155.29 | 0.97 | 0.94 | 0.01 | 0.03 | 0.00 | 0.16 | 0.03 | 0.05 | 0.01 |
| 84 | 10,000 | Block | LQH | 1.5 | 159.28 | 0.97 | 0.94 | 0.01 | 0.03 | 0.00 | 0.16 | 0.03 | 0.05 | 0.01 |
| 84 | 10,000 | Block | LQHPT | 1.8 | 160.69 | 0.98 | 0.93 | 0.01 | 0.05 | 0.01 | 0.40 | 0.14 | 0.07 | 0.01 |
| 84 | 10,000 | Block | LQH | 1.6 | 162.57 | 0.97 | 0.94 | 0.01 | 0.03 | 0.00 | 0.16 | 0.03 | 0.05 | 0.01 |
| 84 | 10,000 | Block | LQHP | 1.5 | 166.74 | 0.97 | 0.94 | 0.01 | 0.04 | 0.00 | 0.21 | 0.03 | 0.05 | 0.01 |
| 84 | 10,000 | Block | LQHPT | 1.5 | 168.19 | 0.98 | 0.93 | 0.01 | 0.05 | 0.01 | 0.46 | 0.14 | 0.09 | 0.01 |
| 84 | 10,000 | Block | LQHP | 1.4 | 187.56 | 0.97 | 0.94 | 0.01 | 0.04 | 0.00 | 0.21 | 0.03 | 0.05 | 0.01 |
| 84 | 10,000 | Block | LQH | 1.4 | 202.61 | 0.97 | 0.94 | 0.01 | 0.03 | 0.00 | 0.16 | 0.03 | 0.06 | 0.00 |
| 84 | 10,000 | Block | LQHP | 1.2 | 217.61 | 0.97 | 0.94 | 0.01 | 0.04 | 0.00 | 0.23 | 0.03 | 0.05 | 0.01 |
| 84 | 10,000 | Block | LQH | 1.3 | 225.90 | 0.97 | 0.94 | 0.01 | 0.04 | 0.00 | 0.18 | 0.03 | 0.06 | 0.00 |
| 84 | 10,000 | Block | LQHPT | 1.2 | 238.57 | 0.98 | 0.93 | 0.01 | 0.06 | 0.01 | 0.46 | 0.17 | 0.13 | 0.01 |
| 84 | 10,000 | Block | H | 1.3 | 242.18 | 0.97 | 0.94 | 0.01 | 0.04 | 0.00 | 0.21 | 0.03 | 0.05 | 0.01 |
| 84 | 10,000 | Block | LQH | 1.2 | 289.59 | 0.97 | 0.94 | 0.01 | 0.04 | 0.00 | 0.24 | 0.04 | 0.06 | 0.00 |
| 84 | 10,000 | Block | L | 0.1 | 296.89 | 0.81 | 0.71 | 0.06 | 0.12 | 0.07 | 0.29 | 0.12 | 0.14 | 0.04 |
| 84 | 10,000 | Block | L | 0.2 | 296.89 | 0.81 | 0.71 | 0.06 | 0.12 | 0.07 | 0.29 | 0.12 | 0.14 | 0.04 |
| 84 | 10,000 | Block | L | 0.3 | 296.89 | 0.81 | 0.71 | 0.06 | 0.12 | 0.07 | 0.29 | 0.12 | 0.14 | 0.04 |
| 84 | 10,000 | Block | L | 0.4 | 296.89 | 0.81 | 0.71 | 0.06 | 0.12 | 0.07 | 0.29 | 0.12 | 0.14 | 0.04 |
| 84 | 10,000 | Block | L | 0.5 | 296.89 | 0.81 | 0.71 | 0.06 | 0.12 | 0.07 | 0.29 | 0.12 | 0.14 | 0.04 |
| 84 | 10,000 | Block | L | 0.6 | 296.89 | 0.81 | 0.71 | 0.06 | 0.12 | 0.07 | 0.29 | 0.12 | 0.14 | 0.04 |
| 84 | 10,000 | Block | L | 0.7 | 296.96 | 0.81 | 0.71 | 0.06 | 0.12 | 0.07 | 0.29 | 0.12 | 0.14 | 0.04 |
| 84 | 10,000 | Block | L | 0.8 | 297.03 | 0.81 | 0.71 | 0.06 | 0.12 | 0.07 | 0.28 | 0.11 | 0.14 | 0.04 |
| 84 | 10,000 | Block | L | 0.9 | 297.13 | 0.81 | 0.71 | 0.06 | 0.12 | 0.07 | 0.28 | 0.11 | 0.14 | 0.04 |
| 84 | 10,000 | Block | L | 1 | 297.24 | 0.81 | 0.71 | 0.06 | 0.12 | 0.07 | 0.28 | 0.11 | 0.14 | 0.04 |
| 84 | 10,000 | Block | L | 1.1 | 297.45 | 0.81 | 0.71 | 0.06 | 0.11 | 0.06 | 0.28 | 0.11 | 0.13 | 0.03 |
| 84 | 10,000 | Block | L | 1.2 | 297.58 | 0.81 | 0.71 | 0.06 | 0.11 | 0.06 | 0.28 | 0.11 | 0.14 | 0.04 |
| 84 | 10,000 | Block | L | 1.3 | 297.74 | 0.81 | 0.71 | 0.05 | 0.11 | 0.06 | 0.27 | 0.10 | 0.14 | 0.04 |
| 84 | 10,000 | Block | L | 1.4 | 297.96 | 0.81 | 0.71 | 0.05 | 0.11 | 0.06 | 0.27 | 0.10 | 0.14 | 0.04 |
| 84 | 10,000 | Block | L | 1.5 | 298.24 | 0.81 | 0.71 | 0.05 | 0.11 | 0.06 | 0.27 | 0.10 | 0.14 | 0.04 |
| 84 | 10,000 | Block | L | 1.6 | 298.51 | 0.81 | 0.71 | 0.05 | 0.11 | 0.06 | 0.27 | 0.10 | 0.14 | 0.04 |
| 84 | 10,000 | Block | L | 1.7 | 298.82 | 0.81 | 0.71 | 0.05 | 0.11 | 0.06 | 0.27 | 0.10 | 0.14 | 0.04 |
| 84 | 10,000 | Block | L | 1.8 | 299.13 | 0.81 | 0.71 | 0.05 | 0.11 | 0.06 | 0.27 | 0.10 | 0.14 | 0.04 |
| 84 | 10,000 | Block | L | 1.9 | 299.45 | 0.81 | 0.72 | 0.05 | 0.11 | 0.06 | 0.27 | 0.10 | 0.13 | 0.04 |
| 84 | 10,000 | Block | L | 2 | 299.82 | 0.81 | 0.72 | 0.05 | 0.11 | 0.06 | 0.27 | 0.10 | 0.13 | 0.04 |
| 84 | 10,000 | Block | LQHP | 1.1 | 309.70 | 0.98 | 0.94 | 0.00 | 0.04 | 0.00 | 0.27 | 0.03 | 0.06 | 0.00 |
| 84 | 10,000 | Block | LQHP | 1.3 | 322.27 | 0.97 | 0.94 | 0.01 | 0.04 | 0.00 | 0.21 | 0.03 | 0.05 | 0.01 |
| 84 | 10,000 | Block | H | 1.4 | 369.29 | 0.97 | 0.94 | 0.01 | 0.03 | 0.00 | 0.21 | 0.03 | 0.05 | 0.01 |
| 84 | 10,000 | Block | H | 1.6 | 376.06 | 0.97 | 0.94 | 0.01 | 0.03 | 0.00 | 0.21 | 0.03 | 0.05 | 0.01 |
| 84 | 10,000 | Block | LQHPT | 1.1 | 380.51 | 0.98 | 0.93 | 0.01 | 0.06 | 0.01 | 0.47 | 0.18 | 0.13 | 0.01 |
| 84 | 10,000 | Block | H | 1.2 | 482.42 | 0.97 | 0.94 | 0.01 | 0.04 | 0.00 | 0.21 | 0.03 | 0.07 | 0.01 |
| 84 | 10,000 | Block | H | 1.5 | 497.49 | 0.97 | 0.94 | 0.01 | 0.03 | 0.00 | 0.20 | 0.03 | 0.05 | 0.01 |
| 84 | 10,000 | Block | H | 1.7 | 544.34 | 0.97 | 0.94 | 0.01 | 0.03 | 0.00 | 0.20 | 0.03 | 0.05 | 0.01 |
| 84 | 10,000 | Block | H | 1.9 | 594.64 | 0.96 | 0.94 | 0.01 | 0.03 | 0.00 | 0.17 | 0.02 | 0.05 | 0.01 |
| 84 | 10,000 | Block | H | 2 | 598.05 | 0.96 | 0.94 | 0.01 | 0.03 | 0.00 | 0.17 | 0.02 | 0.05 | 0.01 |
| 84 | 10,000 | Block | H | 1.8 | 641.48 | 0.97 | 0.94 | 0.01 | 0.03 | 0.00 | 0.17 | 0.02 | 0.05 | 0.01 |
| 84 | 10,000 | Block | LQH | 1.1 | 806.82 | 0.97 | 0.94 | 0.00 | 0.04 | 0.00 | 0.23 | 0.03 | 0.07 | 0.00 |
| 84 | 10,000 | Block | H | 1.1 | 998.10 | 0.97 | 0.94 | 0.01 | 0.04 | 0.00 | 0.23 | 0.04 | 0.07 | 0.01 |
| 84 | 10,000 | Block | LQHP | 0.9 | 1239.71 | 0.98 | 0.94 | 0.00 | 0.05 | 0.00 | 0.34 | 0.06 | 0.09 | 0.01 |
| 84 | 10,000 | Block | LQHP | 1 | 1437.07 | 0.98 | 0.94 | 0.00 | 0.04 | 0.00 | 0.28 | 0.03 | 0.07 | 0.01 |
| 84 | 10,000 | Block | LQH | 1 | 1454.07 | 0.97 | 0.94 | 0.00 | 0.04 | 0.00 | 0.25 | 0.03 | 0.09 | 0.01 |
| 84 | 10,000 | Block | LQHPT | 0.9 | 3043.55 | 0.99 | 0.93 | 0.01 | 0.06 | 0.01 | 0.53 | 0.15 | 0.13 | 0.01 |
| 84 | 10,000 | Block | LQHPT | 1 | 3058.32 | 0.99 | 0.93 | 0.01 | 0.06 | 0.01 | 0.51 | 0.16 | 0.15 | 0.01 |
| 84 | 10,000 | Block | LQH | 0.9 | 6550.96 | 0.97 | 0.94 | 0.00 | 0.05 | 0.00 | 0.31 | 0.06 | 0.09 | 0.01 |
| 84 | 10,000 | Block | H | 0.1 | NA | 0.99 | 0.90 | 0.01 | 0.09 | 0.01 | 0.62 | 0.16 | 0.56 | 0.15 |
| 84 | 10,000 | Block | LQH | 0.1 | NA | 0.99 | 0.90 | 0.01 | 0.09 | 0.01 | 0.63 | 0.16 | 0.56 | 0.15 |
| 84 | 10,000 | Block | LQHP | 0.1 | NA | 0.99 | 0.90 | 0.01 | 0.09 | 0.01 | 0.63 | 0.16 | 0.56 | 0.15 |
| 84 | 10,000 | Block | LQHPT | 0.1 | NA | 0.99 | 0.88 | 0.02 | 0.11 | 0.02 | 0.70 | 0.11 | 0.41 | 0.12 |
| 84 | 10,000 | Block | H | 0.2 | NA | 0.99 | 0.92 | 0.01 | 0.07 | 0.01 | 0.61 | 0.18 | 0.38 | 0.08 |
| 84 | 10,000 | Block | LQH | 0.2 | NA | 0.99 | 0.92 | 0.01 | 0.07 | 0.01 | 0.61 | 0.18 | 0.39 | 0.08 |
| 84 | 10,000 | Block | LQHP | 0.2 | NA | 0.99 | 0.92 | 0.01 | 0.07 | 0.01 | 0.61 | 0.18 | 0.40 | 0.07 |
| 84 | 10,000 | Block | LQHPT | 0.2 | NA | 0.99 | 0.90 | 0.01 | 0.09 | 0.01 | 0.67 | 0.13 | 0.38 | 0.12 |
| 84 | 10,000 | Block | H | 0.3 | NA | 0.98 | 0.93 | 0.01 | 0.06 | 0.01 | 0.59 | 0.17 | 0.22 | 0.03 |
| 84 | 10,000 | Block | LQH | 0.3 | NA | 0.98 | 0.93 | 0.01 | 0.06 | 0.01 | 0.59 | 0.17 | 0.21 | 0.04 |
| 84 | 10,000 | Block | LQHP | 0.3 | NA | 0.98 | 0.93 | 0.01 | 0.06 | 0.01 | 0.61 | 0.18 | 0.23 | 0.03 |
| 84 | 10,000 | Block | LQHPT | 0.3 | NA | 0.99 | 0.91 | 0.01 | 0.08 | 0.01 | 0.69 | 0.10 | 0.40 | 0.11 |
| 84 | 10,000 | Block | H | 0.4 | NA | 0.98 | 0.93 | 0.01 | 0.06 | 0.01 | 0.51 | 0.13 | 0.12 | 0.01 |
| 84 | 10,000 | Block | LQH | 0.4 | NA | 0.98 | 0.93 | 0.01 | 0.06 | 0.01 | 0.51 | 0.13 | 0.16 | 0.02 |
| 84 | 10,000 | Block | LQHP | 0.4 | NA | 0.98 | 0.93 | 0.01 | 0.06 | 0.00 | 0.48 | 0.12 | 0.16 | 0.01 |
| 84 | 10,000 | Block | LQHPT | 0.4 | NA | 0.99 | 0.92 | 0.01 | 0.08 | 0.01 | 0.71 | 0.07 | 0.37 | 0.13 |
| 84 | 10,000 | Block | H | 0.5 | NA | 0.98 | 0.93 | 0.01 | 0.05 | 0.00 | 0.49 | 0.12 | 0.12 | 0.01 |
| 84 | 10,000 | Block | LQH | 0.5 | NA | 0.98 | 0.93 | 0.01 | 0.05 | 0.00 | 0.49 | 0.12 | 0.13 | 0.01 |
| 84 | 10,000 | Block | LQHP | 0.5 | NA | 0.98 | 0.93 | 0.00 | 0.05 | 0.00 | 0.47 | 0.12 | 0.12 | 0.01 |
| 84 | 10,000 | Block | LQHPT | 0.5 | NA | 0.99 | 0.92 | 0.01 | 0.07 | 0.01 | 0.69 | 0.10 | 0.33 | 0.12 |
| 84 | 10,000 | Block | H | 0.6 | NA | 0.98 | 0.94 | 0.00 | 0.05 | 0.00 | 0.45 | 0.11 | 0.12 | 0.01 |
| 84 | 10,000 | Block | LQH | 0.6 | NA | 0.98 | 0.93 | 0.00 | 0.05 | 0.00 | 0.45 | 0.11 | 0.13 | 0.01 |
| 84 | 10,000 | Block | LQHP | 0.6 | NA | 0.98 | 0.94 | 0.00 | 0.05 | 0.00 | 0.46 | 0.12 | 0.12 | 0.01 |
| 84 | 10,000 | Block | LQHPT | 0.6 | NA | 0.99 | 0.92 | 0.01 | 0.07 | 0.01 | 0.64 | 0.12 | 0.18 | 0.05 |
| 84 | 10,000 | Block | H | 0.7 | NA | 0.98 | 0.94 | 0.00 | 0.05 | 0.00 | 0.41 | 0.09 | 0.10 | 0.01 |
| 84 | 10,000 | Block | LQH | 0.7 | NA | 0.98 | 0.93 | 0.00 | 0.05 | 0.00 | 0.45 | 0.11 | 0.12 | 0.01 |
| 84 | 10,000 | Block | LQHP | 0.7 | NA | 0.98 | 0.94 | 0.00 | 0.05 | 0.00 | 0.45 | 0.11 | 0.11 | 0.01 |
| 84 | 10,000 | Block | LQHPT | 0.7 | NA | 0.99 | 0.92 | 0.01 | 0.07 | 0.01 | 0.64 | 0.15 | 0.13 | 0.02 |
| 84 | 10,000 | Block | H | 0.8 | NA | 0.98 | 0.94 | 0.00 | 0.05 | 0.00 | 0.31 | 0.05 | 0.07 | 0.01 |
| 84 | 10,000 | Block | LQH | 0.8 | NA | 0.97 | 0.94 | 0.00 | 0.05 | 0.00 | 0.40 | 0.08 | 0.09 | 0.01 |
| 84 | 10,000 | Block | LQHP | 0.8 | NA | 0.98 | 0.94 | 0.00 | 0.05 | 0.00 | 0.41 | 0.09 | 0.09 | 0.01 |
| 84 | 10,000 | Block | LQHPT | 0.8 | NA | 0.99 | 0.93 | 0.01 | 0.06 | 0.01 | 0.55 | 0.16 | 0.11 | 0.02 |
| 84 | 10,000 | Block | H | 0.9 | NA | 0.97 | 0.94 | 0.00 | 0.04 | 0.00 | 0.27 | 0.03 | 0.07 | 0.01 |
| 84 | 10,000 | Block | H | 1 | NA | 0.97 | 0.94 | 0.00 | 0.04 | 0.00 | 0.24 | 0.04 | 0.07 | 0.01 |
| 84 | 10,000 | Checkerboar1 | LQ | 0.1 | 0.00 | 0.97 | 0.96 | 0.00 | 0.02 | 0.00 | 0.18 | 0.05 | 0.04 | 0.00 |
| 84 | 10,000 | Checkerboar1 | LQ | 0.2 | 0.00 | 0.97 | 0.96 | 0.00 | 0.02 | 0.00 | 0.18 | 0.05 | 0.04 | 0.00 |
| 84 | 10,000 | Checkerboar1 | LQ | 0.3 | 0.00 | 0.97 | 0.96 | 0.00 | 0.02 | 0.00 | 0.18 | 0.05 | 0.04 | 0.00 |
| 84 | 10,000 | Checkerboar1 | LQ | 0.4 | 0.00 | 0.97 | 0.96 | 0.00 | 0.02 | 0.00 | 0.18 | 0.05 | 0.04 | 0.00 |
| 84 | 10,000 | Checkerboar1 | LQ | 0.5 | 0.00 | 0.97 | 0.96 | 0.00 | 0.02 | 0.00 | 0.18 | 0.05 | 0.04 | 0.00 |
| 84 | 10,000 | Checkerboar1 | LQ | 0.6 | 2.05 | 0.97 | 0.95 | 0.00 | 0.02 | 0.00 | 0.13 | 0.02 | 0.04 | 0.00 |
| 84 | 10,000 | Checkerboar1 | LQ | 1.1 | 6.16 | 0.97 | 0.95 | 0.00 | 0.02 | 0.00 | 0.14 | 0.04 | 0.05 | 0.01 |
| 84 | 10,000 | Checkerboar1 | LQ | 1.2 | 6.68 | 0.97 | 0.94 | 0.00 | 0.02 | 0.00 | 0.13 | 0.02 | 0.05 | 0.01 |
| 84 | 10,000 | Checkerboar1 | LQ | 0.7 | 6.87 | 0.97 | 0.96 | 0.00 | 0.02 | 0.00 | 0.17 | 0.06 | 0.04 | 0.00 |
| 84 | 10,000 | Checkerboar1 | LQ | 1 | 7.46 | 0.97 | 0.95 | 0.00 | 0.02 | 0.00 | 0.14 | 0.04 | 0.04 | 0.00 |
| 84 | 10,000 | Checkerboar1 | LQ | 0.8 | 7.60 | 0.97 | 0.95 | 0.00 | 0.02 | 0.00 | 0.14 | 0.04 | 0.04 | 0.00 |
| 84 | 10,000 | Checkerboar1 | LQ | 0.9 | 9.73 | 0.97 | 0.95 | 0.00 | 0.02 | 0.00 | 0.14 | 0.04 | 0.04 | 0.00 |
| 84 | 10,000 | Checkerboar1 | LQ | 1.5 | 10.59 | 0.97 | 0.94 | 0.00 | 0.02 | 0.00 | 0.14 | 0.03 | 0.04 | 0.00 |
| 84 | 10,000 | Checkerboar1 | LQ | 1.3 | 11.12 | 0.97 | 0.94 | 0.00 | 0.02 | 0.00 | 0.13 | 0.02 | 0.04 | 0.00 |
| 84 | 10,000 | Checkerboar1 | LQ | 1.4 | 14.53 | 0.97 | 0.94 | 0.00 | 0.02 | 0.00 | 0.13 | 0.02 | 0.04 | 0.00 |
| 84 | 10,000 | Checkerboar1 | LQ | 1.6 | 17.69 | 0.97 | 0.93 | 0.00 | 0.02 | 0.00 | 0.15 | 0.03 | 0.04 | 0.00 |
| 84 | 10,000 | Checkerboar1 | LQ | 1.7 | 21.63 | 0.97 | 0.93 | 0.00 | 0.02 | 0.00 | 0.17 | 0.04 | 0.04 | 0.00 |
| 84 | 10,000 | Checkerboar1 | LQ | 2 | 23.54 | 0.96 | 0.93 | 0.00 | 0.02 | 0.00 | 0.16 | 0.02 | 0.04 | 0.00 |
| 84 | 10,000 | Checkerboar1 | LQ | 1.8 | 23.88 | 0.97 | 0.93 | 0.00 | 0.02 | 0.00 | 0.19 | 0.03 | 0.04 | 0.00 |
| 84 | 10,000 | Checkerboar1 | LQ | 1.9 | 24.38 | 0.96 | 0.93 | 0.00 | 0.02 | 0.00 | 0.16 | 0.03 | 0.04 | 0.00 |
| 84 | 10,000 | Checkerboar1 | LQHPT | 2 | 81.86 | 0.98 | 0.95 | 0.00 | 0.02 | 0.00 | 0.21 | 0.01 | 0.05 | 0.01 |
| 84 | 10,000 | Checkerboar1 | LQH | 2 | 87.82 | 0.96 | 0.95 | 0.00 | 0.02 | 0.00 | 0.12 | 0.03 | 0.09 | 0.02 |
| 84 | 10,000 | Checkerboar1 | LQHP | 1.9 | 102.51 | 0.97 | 0.95 | 0.00 | 0.02 | 0.00 | 0.12 | 0.03 | 0.05 | 0.01 |
| 84 | 10,000 | Checkerboar1 | LQHPT | 1.3 | 105.35 | 0.98 | 0.96 | 0.00 | 0.02 | 0.00 | 0.25 | 0.00 | 0.07 | 0.00 |
| 84 | 10,000 | Checkerboar1 | LQHPT | 1.9 | 105.40 | 0.98 | 0.95 | 0.00 | 0.02 | 0.00 | 0.21 | 0.01 | 0.05 | 0.01 |
| 84 | 10,000 | Checkerboar1 | LQHP | 2 | 106.16 | 0.97 | 0.95 | 0.00 | 0.02 | 0.00 | 0.12 | 0.03 | 0.05 | 0.01 |
| 84 | 10,000 | Checkerboar1 | LQHPT | 1.7 | 114.75 | 0.98 | 0.95 | 0.00 | 0.02 | 0.00 | 0.22 | 0.01 | 0.07 | 0.00 |
| 84 | 10,000 | Checkerboar1 | LQHP | 1.7 | 123.02 | 0.97 | 0.95 | 0.00 | 0.02 | 0.00 | 0.12 | 0.03 | 0.05 | 0.01 |
| 84 | 10,000 | Checkerboar1 | LQH | 1.9 | 125.52 | 0.97 | 0.95 | 0.00 | 0.02 | 0.00 | 0.12 | 0.03 | 0.09 | 0.02 |
| 84 | 10,000 | Checkerboar1 | LQH | 1.8 | 131.08 | 0.97 | 0.95 | 0.00 | 0.02 | 0.00 | 0.12 | 0.03 | 0.06 | 0.01 |
| 84 | 10,000 | Checkerboar1 | LQHPT | 1.6 | 132.34 | 0.98 | 0.95 | 0.00 | 0.02 | 0.00 | 0.23 | 0.01 | 0.07 | 0.00 |
| 84 | 10,000 | Checkerboar1 | LQHPT | 1.4 | 141.46 | 0.98 | 0.95 | 0.00 | 0.02 | 0.00 | 0.22 | 0.00 | 0.07 | 0.00 |
| 84 | 10,000 | Checkerboar1 | LQHP | 1.8 | 145.09 | 0.97 | 0.95 | 0.00 | 0.02 | 0.00 | 0.12 | 0.03 | 0.05 | 0.01 |
| 84 | 10,000 | Checkerboar1 | LQHP | 1.6 | 146.91 | 0.97 | 0.95 | 0.00 | 0.02 | 0.00 | 0.12 | 0.03 | 0.05 | 0.01 |
| 84 | 10,000 | Checkerboar1 | LQH | 1.7 | 155.29 | 0.97 | 0.95 | 0.00 | 0.02 | 0.00 | 0.12 | 0.03 | 0.06 | 0.01 |
| 84 | 10,000 | Checkerboar1 | LQH | 1.5 | 159.28 | 0.97 | 0.95 | 0.00 | 0.02 | 0.00 | 0.12 | 0.03 | 0.05 | 0.01 |
| 84 | 10,000 | Checkerboar1 | LQHPT | 1.8 | 160.69 | 0.98 | 0.95 | 0.00 | 0.02 | 0.00 | 0.21 | 0.01 | 0.06 | 0.00 |
| 84 | 10,000 | Checkerboar1 | LQH | 1.6 | 162.57 | 0.97 | 0.95 | 0.00 | 0.02 | 0.00 | 0.12 | 0.03 | 0.06 | 0.01 |
| 84 | 10,000 | Checkerboar1 | LQHP | 1.5 | 166.74 | 0.97 | 0.95 | 0.00 | 0.02 | 0.00 | 0.14 | 0.02 | 0.05 | 0.01 |
| 84 | 10,000 | Checkerboar1 | LQHPT | 1.5 | 168.19 | 0.98 | 0.95 | 0.00 | 0.02 | 0.00 | 0.22 | 0.00 | 0.07 | 0.00 |
| 84 | 10,000 | Checkerboar1 | LQHP | 1.4 | 187.56 | 0.97 | 0.95 | 0.00 | 0.02 | 0.00 | 0.15 | 0.01 | 0.05 | 0.01 |
| 84 | 10,000 | Checkerboar1 | LQH | 1.4 | 202.61 | 0.97 | 0.95 | 0.00 | 0.02 | 0.00 | 0.13 | 0.02 | 0.05 | 0.01 |
| 84 | 10,000 | Checkerboar1 | LQHP | 1.2 | 217.61 | 0.97 | 0.95 | 0.00 | 0.02 | 0.00 | 0.16 | 0.01 | 0.05 | 0.01 |
| 84 | 10,000 | Checkerboar1 | LQH | 1.3 | 225.90 | 0.97 | 0.95 | 0.00 | 0.02 | 0.00 | 0.14 | 0.02 | 0.05 | 0.01 |
| 84 | 10,000 | Checkerboar1 | LQHPT | 1.2 | 238.57 | 0.98 | 0.96 | 0.00 | 0.02 | 0.00 | 0.27 | 0.00 | 0.07 | 0.00 |
| 84 | 10,000 | Checkerboar1 | H | 1.3 | 242.18 | 0.97 | 0.95 | 0.00 | 0.02 | 0.00 | 0.14 | 0.02 | 0.05 | 0.01 |
| 84 | 10,000 | Checkerboar1 | LQH | 1.2 | 289.59 | 0.97 | 0.95 | 0.00 | 0.02 | 0.00 | 0.14 | 0.02 | 0.05 | 0.01 |
| 84 | 10,000 | Checkerboar1 | L | 0.1 | 296.89 | 0.81 | 0.80 | 0.00 | 0.01 | 0.00 | 0.13 | 0.00 | 0.04 | 0.00 |
| 84 | 10,000 | Checkerboar1 | L | 0.2 | 296.89 | 0.81 | 0.80 | 0.00 | 0.01 | 0.00 | 0.13 | 0.00 | 0.04 | 0.00 |
| 84 | 10,000 | Checkerboar1 | L | 0.3 | 296.89 | 0.81 | 0.80 | 0.00 | 0.01 | 0.00 | 0.12 | 0.00 | 0.04 | 0.00 |
| 84 | 10,000 | Checkerboar1 | L | 0.4 | 296.89 | 0.81 | 0.80 | 0.00 | 0.01 | 0.00 | 0.12 | 0.00 | 0.04 | 0.00 |
| 84 | 10,000 | Checkerboar1 | L | 0.5 | 296.89 | 0.81 | 0.80 | 0.00 | 0.01 | 0.00 | 0.11 | 0.00 | 0.04 | 0.00 |
| 84 | 10,000 | Checkerboar1 | L | 0.6 | 296.89 | 0.81 | 0.80 | 0.00 | 0.01 | 0.00 | 0.10 | 0.00 | 0.04 | 0.00 |
| 84 | 10,000 | Checkerboar1 | L | 0.7 | 296.96 | 0.81 | 0.80 | 0.00 | 0.01 | 0.00 | 0.10 | 0.00 | 0.03 | 0.00 |
| 84 | 10,000 | Checkerboar1 | L | 0.8 | 297.03 | 0.81 | 0.80 | 0.00 | 0.01 | 0.00 | 0.10 | 0.00 | 0.03 | 0.00 |
| 84 | 10,000 | Checkerboar1 | L | 0.9 | 297.13 | 0.81 | 0.80 | 0.00 | 0.01 | 0.00 | 0.10 | 0.00 | 0.03 | 0.00 |
| 84 | 10,000 | Checkerboar1 | L | 1 | 297.24 | 0.81 | 0.80 | 0.00 | 0.01 | 0.00 | 0.10 | 0.00 | 0.03 | 0.00 |
| 84 | 10,000 | Checkerboar1 | L | 1.1 | 297.45 | 0.81 | 0.80 | 0.00 | 0.01 | 0.00 | 0.10 | 0.00 | 0.00 | 0.00 |
| 84 | 10,000 | Checkerboar1 | L | 1.2 | 297.58 | 0.81 | 0.79 | 0.00 | 0.01 | 0.00 | 0.10 | 0.00 | 0.00 | 0.00 |
| 84 | 10,000 | Checkerboar1 | L | 1.3 | 297.74 | 0.81 | 0.79 | 0.00 | 0.01 | 0.00 | 0.11 | 0.00 | 0.00 | 0.00 |
| 84 | 10,000 | Checkerboar1 | L | 1.4 | 297.96 | 0.81 | 0.79 | 0.00 | 0.01 | 0.00 | 0.11 | 0.00 | 0.00 | 0.00 |
| 84 | 10,000 | Checkerboar1 | L | 1.5 | 298.24 | 0.81 | 0.79 | 0.00 | 0.02 | 0.00 | 0.11 | 0.00 | 0.00 | 0.00 |
| 84 | 10,000 | Checkerboar1 | L | 1.6 | 298.51 | 0.81 | 0.79 | 0.00 | 0.02 | 0.00 | 0.11 | 0.00 | 0.00 | 0.00 |
| 84 | 10,000 | Checkerboar1 | L | 1.7 | 298.82 | 0.81 | 0.79 | 0.00 | 0.02 | 0.00 | 0.11 | 0.00 | 0.00 | 0.00 |
| 84 | 10,000 | Checkerboar1 | L | 1.8 | 299.13 | 0.81 | 0.79 | 0.00 | 0.01 | 0.00 | 0.11 | 0.00 | 0.00 | 0.00 |
| 84 | 10,000 | Checkerboar1 | L | 1.9 | 299.45 | 0.81 | 0.79 | 0.00 | 0.01 | 0.00 | 0.11 | 0.00 | 0.00 | 0.00 |
| 84 | 10,000 | Checkerboar1 | L | 2 | 299.82 | 0.81 | 0.79 | 0.00 | 0.01 | 0.00 | 0.11 | 0.00 | 0.00 | 0.00 |
| 84 | 10,000 | Checkerboar1 | LQHP | 1.1 | 309.70 | 0.98 | 0.95 | 0.00 | 0.02 | 0.00 | 0.16 | 0.01 | 0.05 | 0.01 |
| 84 | 10,000 | Checkerboar1 | LQHP | 1.3 | 322.27 | 0.97 | 0.95 | 0.00 | 0.02 | 0.00 | 0.16 | 0.01 | 0.05 | 0.01 |
| 84 | 10,000 | Checkerboar1 | H | 1.4 | 369.29 | 0.97 | 0.95 | 0.00 | 0.02 | 0.00 | 0.13 | 0.02 | 0.05 | 0.01 |
| 84 | 10,000 | Checkerboar1 | H | 1.6 | 376.06 | 0.97 | 0.95 | 0.00 | 0.02 | 0.00 | 0.12 | 0.03 | 0.05 | 0.01 |
| 84 | 10,000 | Checkerboar1 | LQHPT | 1.1 | 380.51 | 0.98 | 0.96 | 0.00 | 0.02 | 0.00 | 0.27 | 0.00 | 0.07 | 0.00 |
| 84 | 10,000 | Checkerboar1 | H | 1.2 | 482.42 | 0.97 | 0.95 | 0.00 | 0.02 | 0.00 | 0.16 | 0.01 | 0.05 | 0.01 |
| 84 | 10,000 | Checkerboar1 | H | 1.5 | 497.49 | 0.97 | 0.95 | 0.00 | 0.02 | 0.00 | 0.12 | 0.03 | 0.05 | 0.01 |
| 84 | 10,000 | Checkerboar1 | H | 1.7 | 544.34 | 0.97 | 0.95 | 0.00 | 0.02 | 0.00 | 0.12 | 0.03 | 0.05 | 0.01 |
| 84 | 10,000 | Checkerboar1 | H | 1.9 | 594.64 | 0.96 | 0.95 | 0.00 | 0.02 | 0.00 | 0.12 | 0.03 | 0.05 | 0.01 |
| 84 | 10,000 | Checkerboar1 | H | 2 | 598.05 | 0.96 | 0.95 | 0.00 | 0.02 | 0.00 | 0.12 | 0.03 | 0.05 | 0.01 |
| 84 | 10,000 | Checkerboar1 | H | 1.8 | 641.48 | 0.97 | 0.95 | 0.00 | 0.02 | 0.00 | 0.12 | 0.03 | 0.05 | 0.01 |
| 84 | 10,000 | Checkerboar1 | LQH | 1.1 | 806.82 | 0.97 | 0.95 | 0.00 | 0.02 | 0.00 | 0.16 | 0.01 | 0.05 | 0.01 |
| 84 | 10,000 | Checkerboar1 | H | 1.1 | 998.10 | 0.97 | 0.95 | 0.00 | 0.02 | 0.00 | 0.16 | 0.01 | 0.05 | 0.01 |
| 84 | 10,000 | Checkerboar1 | LQHP | 0.9 | 1239.71 | 0.98 | 0.95 | 0.00 | 0.02 | 0.00 | 0.17 | 0.01 | 0.05 | 0.01 |
| 84 | 10,000 | Checkerboar1 | LQHP | 1 | 1437.07 | 0.98 | 0.95 | 0.00 | 0.02 | 0.00 | 0.16 | 0.01 | 0.05 | 0.01 |
| 84 | 10,000 | Checkerboar1 | LQH | 1 | 1454.07 | 0.97 | 0.95 | 0.00 | 0.02 | 0.00 | 0.16 | 0.01 | 0.05 | 0.01 |
| 84 | 10,000 | Checkerboar1 | LQHPT | 0.9 | 3043.55 | 0.99 | 0.96 | 0.00 | 0.03 | 0.00 | 0.29 | 0.00 | 0.09 | 0.00 |
| 84 | 10,000 | Checkerboar1 | LQHPT | 1 | 3058.32 | 0.99 | 0.96 | 0.00 | 0.02 | 0.00 | 0.28 | 0.00 | 0.09 | 0.00 |
| 84 | 10,000 | Checkerboar1 | LQH | 0.9 | 6550.96 | 0.97 | 0.96 | 0.00 | 0.02 | 0.00 | 0.16 | 0.01 | 0.05 | 0.01 |
| 84 | 10,000 | Checkerboar1 | H | 0.1 | NA | 0.99 | 0.96 | 0.00 | 0.03 | 0.00 | 0.30 | 0.00 | 0.16 | 0.00 |
| 84 | 10,000 | Checkerboar1 | LQH | 0.1 | NA | 0.99 | 0.96 | 0.00 | 0.03 | 0.00 | 0.31 | 0.00 | 0.16 | 0.00 |
| 84 | 10,000 | Checkerboar1 | LQHP | 0.1 | NA | 0.99 | 0.96 | 0.00 | 0.03 | 0.00 | 0.31 | 0.00 | 0.18 | 0.00 |
| 84 | 10,000 | Checkerboar1 | LQHPT | 0.1 | NA | 0.99 | 0.94 | 0.00 | 0.05 | 0.00 | 0.57 | 0.00 | 0.13 | 0.02 |
| 84 | 10,000 | Checkerboar1 | H | 0.2 | NA | 0.99 | 0.96 | 0.00 | 0.03 | 0.00 | 0.26 | 0.01 | 0.09 | 0.00 |
| 84 | 10,000 | Checkerboar1 | LQH | 0.2 | NA | 0.99 | 0.96 | 0.00 | 0.03 | 0.00 | 0.26 | 0.01 | 0.09 | 0.00 |
| 84 | 10,000 | Checkerboar1 | LQHP | 0.2 | NA | 0.99 | 0.96 | 0.00 | 0.03 | 0.00 | 0.27 | 0.00 | 0.07 | 0.00 |
| 84 | 10,000 | Checkerboar1 | LQHPT | 0.2 | NA | 0.99 | 0.94 | 0.00 | 0.05 | 0.00 | 0.51 | 0.00 | 0.15 | 0.01 |
| 84 | 10,000 | Checkerboar1 | H | 0.3 | NA | 0.98 | 0.96 | 0.00 | 0.03 | 0.00 | 0.24 | 0.00 | 0.09 | 0.00 |
| 84 | 10,000 | Checkerboar1 | LQH | 0.3 | NA | 0.98 | 0.96 | 0.00 | 0.03 | 0.00 | 0.24 | 0.00 | 0.09 | 0.00 |
| 84 | 10,000 | Checkerboar1 | LQHP | 0.3 | NA | 0.98 | 0.96 | 0.00 | 0.03 | 0.00 | 0.25 | 0.00 | 0.07 | 0.00 |
| 84 | 10,000 | Checkerboar1 | LQHPT | 0.3 | NA | 0.99 | 0.94 | 0.00 | 0.05 | 0.00 | 0.42 | 0.00 | 0.16 | 0.01 |
| 84 | 10,000 | Checkerboar1 | H | 0.4 | NA | 0.98 | 0.96 | 0.00 | 0.02 | 0.00 | 0.24 | 0.00 | 0.09 | 0.00 |
| 84 | 10,000 | Checkerboar1 | LQH | 0.4 | NA | 0.98 | 0.96 | 0.00 | 0.02 | 0.00 | 0.23 | 0.01 | 0.09 | 0.00 |
| 84 | 10,000 | Checkerboar1 | LQHP | 0.4 | NA | 0.98 | 0.96 | 0.00 | 0.02 | 0.00 | 0.25 | 0.00 | 0.07 | 0.00 |
| 84 | 10,000 | Checkerboar1 | LQHPT | 0.4 | NA | 0.99 | 0.94 | 0.00 | 0.05 | 0.00 | 0.42 | 0.00 | 0.13 | 0.01 |
| 84 | 10,000 | Checkerboar1 | H | 0.5 | NA | 0.98 | 0.96 | 0.00 | 0.02 | 0.00 | 0.22 | 0.00 | 0.09 | 0.00 |
| 84 | 10,000 | Checkerboar1 | LQH | 0.5 | NA | 0.98 | 0.96 | 0.00 | 0.02 | 0.00 | 0.23 | 0.00 | 0.09 | 0.00 |
| 84 | 10,000 | Checkerboar1 | LQHP | 0.5 | NA | 0.98 | 0.96 | 0.00 | 0.02 | 0.00 | 0.25 | 0.00 | 0.07 | 0.00 |
| 84 | 10,000 | Checkerboar1 | LQHPT | 0.5 | NA | 0.99 | 0.95 | 0.00 | 0.04 | 0.00 | 0.36 | 0.00 | 0.11 | 0.00 |
| 84 | 10,000 | Checkerboar1 | H | 0.6 | NA | 0.98 | 0.96 | 0.00 | 0.02 | 0.00 | 0.18 | 0.00 | 0.09 | 0.00 |
| 84 | 10,000 | Checkerboar1 | LQH | 0.6 | NA | 0.98 | 0.96 | 0.00 | 0.02 | 0.00 | 0.18 | 0.00 | 0.09 | 0.00 |
| 84 | 10,000 | Checkerboar1 | LQHP | 0.6 | NA | 0.98 | 0.96 | 0.00 | 0.02 | 0.00 | 0.23 | 0.00 | 0.07 | 0.00 |
| 84 | 10,000 | Checkerboar1 | LQHPT | 0.6 | NA | 0.99 | 0.96 | 0.00 | 0.03 | 0.00 | 0.38 | 0.01 | 0.11 | 0.00 |
| 84 | 10,000 | Checkerboar1 | H | 0.7 | NA | 0.98 | 0.96 | 0.00 | 0.02 | 0.00 | 0.16 | 0.01 | 0.08 | 0.01 |
| 84 | 10,000 | Checkerboar1 | LQH | 0.7 | NA | 0.98 | 0.96 | 0.00 | 0.02 | 0.00 | 0.17 | 0.01 | 0.08 | 0.01 |
| 84 | 10,000 | Checkerboar1 | LQHP | 0.7 | NA | 0.98 | 0.96 | 0.00 | 0.02 | 0.00 | 0.22 | 0.00 | 0.05 | 0.01 |
| 84 | 10,000 | Checkerboar1 | LQHPT | 0.7 | NA | 0.99 | 0.96 | 0.00 | 0.03 | 0.00 | 0.29 | 0.00 | 0.10 | 0.00 |
| 84 | 10,000 | Checkerboar1 | H | 0.8 | NA | 0.98 | 0.96 | 0.00 | 0.02 | 0.00 | 0.16 | 0.01 | 0.06 | 0.01 |
| 84 | 10,000 | Checkerboar1 | LQH | 0.8 | NA | 0.97 | 0.96 | 0.00 | 0.02 | 0.00 | 0.16 | 0.01 | 0.05 | 0.01 |
| 84 | 10,000 | Checkerboar1 | LQHP | 0.8 | NA | 0.98 | 0.95 | 0.00 | 0.02 | 0.00 | 0.21 | 0.00 | 0.05 | 0.01 |
| 84 | 10,000 | Checkerboar1 | LQHPT | 0.8 | NA | 0.99 | 0.96 | 0.00 | 0.03 | 0.00 | 0.29 | 0.00 | 0.07 | 0.00 |
| 84 | 10,000 | Checkerboar1 | H | 0.9 | NA | 0.97 | 0.96 | 0.00 | 0.02 | 0.00 | 0.16 | 0.01 | 0.06 | 0.01 |
| 84 | 10,000 | Checkerboar1 | H | 1 | NA | 0.97 | 0.95 | 0.00 | 0.02 | 0.00 | 0.16 | 0.01 | 0.05 | 0.01 |

***Calibration area No 3: Starry stonewort entire species range considering maximum distance between closer neighbor occurrences (~700 km, M_d_)***

| n | BCK | Partition | FC | RM | ∆AICc | AUC test | AUC mean | AUC variance | AUC mean difference | AUC var. difference | OR mean 10% | OR var. 10% | OR mean MTP | OR var MTP |
| --- | --- | --- | --- | --- | --- | --- | --- | --- | --- | --- | --- | --- | --- | --- |
| 84 | 10,000 | Jackknife | LQ | 0.9 | 0.00 | 0.89 | 0.88 | 2.14 | 0.06 | 1.52 | 0.12 | 0.11 | 0.01 | 0.01 |
| 84 | 10,000 | Jackknife | LQ | 0.1 | 0.81 | 0.90 | 0.88 | 1.91 | 0.06 | 1.36 | 0.12 | 0.11 | 0.01 | 0.01 |
| 84 | 10,000 | Jackknife | LQ | 0.2 | 0.81 | 0.90 | 0.88 | 1.91 | 0.06 | 1.36 | 0.12 | 0.11 | 0.01 | 0.01 |
| 84 | 10,000 | Jackknife | LQ | 0.3 | 0.81 | 0.90 | 0.88 | 1.91 | 0.06 | 1.36 | 0.12 | 0.11 | 0.01 | 0.01 |
| 84 | 10,000 | Jackknife | LQ | 0.4 | 1.18 | 0.90 | 0.88 | 1.94 | 0.06 | 1.39 | 0.11 | 0.10 | 0.01 | 0.01 |
| 84 | 10,000 | Jackknife | LQ | 0.7 | 1.23 | 0.89 | 0.88 | 2.01 | 0.06 | 1.42 | 0.13 | 0.12 | 0.01 | 0.01 |
| 84 | 10,000 | Jackknife | LQ | 0.6 | 1.71 | 0.89 | 0.88 | 1.96 | 0.06 | 1.39 | 0.12 | 0.11 | 0.01 | 0.01 |
| 84 | 10,000 | Jackknife | LQ | 0.5 | 2.21 | 0.90 | 0.88 | 1.93 | 0.06 | 1.38 | 0.11 | 0.10 | 0.01 | 0.01 |
| 84 | 10,000 | Jackknife | LQ | 0.8 | 2.71 | 0.89 | 0.88 | 2.08 | 0.06 | 1.46 | 0.12 | 0.11 | 0.01 | 0.01 |
| 84 | 10,000 | Jackknife | LQ | 1 | 5.45 | 0.89 | 0.88 | 2.08 | 0.06 | 1.44 | 0.12 | 0.11 | 0.01 | 0.01 |
| 84 | 10,000 | Jackknife | LQ | 1.2 | 7.55 | 0.89 | 0.87 | 2.15 | 0.07 | 1.46 | 0.12 | 0.11 | 0.01 | 0.01 |
| 84 | 10,000 | Jackknife | LQ | 1.4 | 8.01 | 0.88 | 0.87 | 2.25 | 0.07 | 1.48 | 0.11 | 0.10 | 0.01 | 0.01 |
| 84 | 10,000 | Jackknife | LQ | 1.1 | 14.37 | 0.89 | 0.87 | 2.16 | 0.07 | 1.48 | 0.12 | 0.11 | 0.01 | 0.01 |
| 84 | 10,000 | Jackknife | LQ | 1.3 | 14.45 | 0.89 | 0.87 | 2.21 | 0.07 | 1.49 | 0.12 | 0.11 | 0.01 | 0.01 |
| 84 | 10,000 | Jackknife | LQ | 1.7 | 16.73 | 0.88 | 0.87 | 2.35 | 0.07 | 1.52 | 0.14 | 0.12 | 0.01 | 0.01 |
| 84 | 10,000 | Jackknife | LQ | 1.6 | 17.28 | 0.88 | 0.87 | 2.29 | 0.07 | 1.49 | 0.12 | 0.11 | 0.01 | 0.01 |
| 84 | 10,000 | Jackknife | LQ | 1.8 | 17.71 | 0.88 | 0.86 | 2.39 | 0.07 | 1.53 | 0.14 | 0.12 | 0.01 | 0.01 |
| 84 | 10,000 | Jackknife | LQ | 1.5 | 17.73 | 0.88 | 0.87 | 2.31 | 0.07 | 1.51 | 0.12 | 0.11 | 0.01 | 0.01 |
| 84 | 10,000 | Jackknife | LQ | 1.9 | 19.09 | 0.87 | 0.86 | 2.45 | 0.07 | 1.56 | 0.15 | 0.13 | 0.01 | 0.01 |
| 84 | 10,000 | Jackknife | LQ | 2 | 21.46 | 0.87 | 0.86 | 2.48 | 0.07 | 1.57 | 0.15 | 0.13 | 0.01 | 0.01 |
| 84 | 10,000 | Jackknife | LQH | 1.7 | 54.05 | 0.91 | 0.89 | 1.66 | 0.06 | 1.21 | 0.14 | 0.12 | 0.01 | 0.01 |
| 84 | 10,000 | Jackknife | LQH | 2 | 54.83 | 0.91 | 0.89 | 1.68 | 0.06 | 1.20 | 0.12 | 0.11 | 0.01 | 0.01 |
| 84 | 10,000 | Jackknife | LQHPT | 1.6 | 55.64 | 0.93 | 0.90 | 1.46 | 0.06 | 1.14 | 0.23 | 0.18 | 0.01 | 0.01 |
| 84 | 10,000 | Jackknife | LQH | 1.9 | 57.76 | 0.91 | 0.89 | 1.69 | 0.06 | 1.21 | 0.12 | 0.11 | 0.01 | 0.01 |
| 84 | 10,000 | Jackknife | LQHP | 1.8 | 61.96 | 0.91 | 0.89 | 1.61 | 0.06 | 1.16 | 0.14 | 0.12 | 0.01 | 0.01 |
| 84 | 10,000 | Jackknife | H | 1.7 | 63.46 | 0.91 | 0.89 | 1.63 | 0.06 | 1.20 | 0.14 | 0.12 | 0.01 | 0.01 |
| 84 | 10,000 | Jackknife | LQHP | 1.7 | 66.63 | 0.91 | 0.89 | 1.61 | 0.06 | 1.16 | 0.14 | 0.12 | 0.01 | 0.01 |
| 84 | 10,000 | Jackknife | H | 1.9 | 66.83 | 0.91 | 0.89 | 1.63 | 0.06 | 1.20 | 0.17 | 0.14 | 0.01 | 0.01 |
| 84 | 10,000 | Jackknife | LQHPT | 1.7 | 67.39 | 0.93 | 0.90 | 1.45 | 0.06 | 1.13 | 0.23 | 0.18 | 0.01 | 0.01 |
| 84 | 10,000 | Jackknife | LQH | 1.4 | 67.78 | 0.91 | 0.89 | 1.63 | 0.06 | 1.21 | 0.15 | 0.13 | 0.01 | 0.01 |
| 84 | 10,000 | Jackknife | H | 2 | 68.58 | 0.91 | 0.89 | 1.64 | 0.06 | 1.19 | 0.17 | 0.14 | 0.01 | 0.01 |
| 84 | 10,000 | Jackknife | LQH | 1.5 | 69.54 | 0.91 | 0.89 | 1.64 | 0.06 | 1.21 | 0.15 | 0.13 | 0.01 | 0.01 |
| 84 | 10,000 | Jackknife | H | 1.8 | 71.51 | 0.91 | 0.89 | 1.63 | 0.06 | 1.20 | 0.17 | 0.14 | 0.01 | 0.01 |
| 84 | 10,000 | Jackknife | LQH | 1.6 | 71.62 | 0.91 | 0.89 | 1.65 | 0.06 | 1.20 | 0.14 | 0.12 | 0.01 | 0.01 |
| 84 | 10,000 | Jackknife | LQHPT | 1.9 | 75.76 | 0.93 | 0.89 | 1.48 | 0.06 | 1.13 | 0.23 | 0.18 | 0.01 | 0.01 |
| 84 | 10,000 | Jackknife | LQHPT | 1.2 | 76.43 | 0.94 | 0.90 | 1.34 | 0.06 | 1.10 | 0.21 | 0.17 | 0.01 | 0.01 |
| 84 | 10,000 | Jackknife | LQHPT | 2 | 80.36 | 0.92 | 0.89 | 1.50 | 0.06 | 1.14 | 0.23 | 0.18 | 0.01 | 0.01 |
| 84 | 10,000 | Jackknife | H | 1.5 | 80.56 | 0.91 | 0.89 | 1.62 | 0.06 | 1.19 | 0.14 | 0.12 | 0.01 | 0.01 |
| 84 | 10,000 | Jackknife | H | 1.6 | 82.05 | 0.91 | 0.89 | 1.63 | 0.06 | 1.20 | 0.14 | 0.12 | 0.01 | 0.01 |
| 84 | 10,000 | Jackknife | LQHP | 2 | 84.64 | 0.91 | 0.89 | 1.63 | 0.06 | 1.16 | 0.12 | 0.11 | 0.01 | 0.01 |
| 84 | 10,000 | Jackknife | LQHPT | 1.3 | 85.66 | 0.94 | 0.90 | 1.37 | 0.06 | 1.10 | 0.23 | 0.18 | 0.01 | 0.01 |
| 84 | 10,000 | Jackknife | LQH | 1.8 | 88.35 | 0.91 | 0.89 | 1.67 | 0.06 | 1.21 | 0.13 | 0.12 | 0.01 | 0.01 |
| 84 | 10,000 | Jackknife | LQHPT | 1.8 | 88.38 | 0.93 | 0.89 | 1.47 | 0.06 | 1.13 | 0.23 | 0.18 | 0.01 | 0.01 |
| 84 | 10,000 | Jackknife | LQHP | 1.9 | 90.10 | 0.91 | 0.89 | 1.62 | 0.06 | 1.16 | 0.14 | 0.12 | 0.01 | 0.01 |
| 84 | 10,000 | Jackknife | LQHP | 1.6 | 92.58 | 0.91 | 0.89 | 1.59 | 0.06 | 1.16 | 0.14 | 0.12 | 0.01 | 0.01 |
| 84 | 10,000 | Jackknife | H | 1.4 | 94.25 | 0.91 | 0.89 | 1.61 | 0.06 | 1.20 | 0.15 | 0.13 | 0.01 | 0.01 |
| 84 | 10,000 | Jackknife | LQHP | 1.5 | 98.82 | 0.91 | 0.89 | 1.59 | 0.06 | 1.16 | 0.14 | 0.12 | 0.01 | 0.01 |
| 84 | 10,000 | Jackknife | LQHPT | 1.5 | 101.47 | 0.93 | 0.90 | 1.44 | 0.06 | 1.14 | 0.24 | 0.18 | 0.01 | 0.01 |
| 84 | 10,000 | Jackknife | LQHPT | 1.4 | 104.32 | 0.94 | 0.90 | 1.40 | 0.06 | 1.12 | 0.23 | 0.18 | 0.01 | 0.01 |
| 84 | 10,000 | Jackknife | LQHPT | 1.1 | 110.31 | 0.95 | 0.90 | 1.32 | 0.06 | 1.10 | 0.23 | 0.18 | 0.01 | 0.01 |
| 84 | 10,000 | Jackknife | LQH | 1.3 | 111.87 | 0.91 | 0.89 | 1.64 | 0.06 | 1.21 | 0.15 | 0.13 | 0.01 | 0.01 |
| 84 | 10,000 | Jackknife | LQHP | 1.3 | 121.13 | 0.91 | 0.89 | 1.62 | 0.06 | 1.21 | 0.14 | 0.12 | 0.01 | 0.01 |
| 84 | 10,000 | Jackknife | LQHP | 1.4 | 122.68 | 0.91 | 0.89 | 1.59 | 0.06 | 1.17 | 0.14 | 0.12 | 0.01 | 0.01 |
| 84 | 10,000 | Jackknife | LQHP | 1.2 | 127.86 | 0.92 | 0.89 | 1.65 | 0.06 | 1.24 | 0.14 | 0.12 | 0.01 | 0.01 |
| 84 | 10,000 | Jackknife | LQH | 1.2 | 127.86 | 0.91 | 0.89 | 1.67 | 0.06 | 1.25 | 0.15 | 0.13 | 0.01 | 0.01 |
| 84 | 10,000 | Jackknife | H | 1.2 | 135.98 | 0.91 | 0.89 | 1.64 | 0.06 | 1.23 | 0.15 | 0.13 | 0.01 | 0.01 |
| 84 | 10,000 | Jackknife | LQHPT | 1 | 139.22 | 0.95 | 0.90 | 1.31 | 0.06 | 1.11 | 0.20 | 0.16 | 0.01 | 0.01 |
| 84 | 10,000 | Jackknife | L | 0.1 | 146.15 | 0.72 | 0.71 | 6.09 | 0.13 | 3.12 | 0.14 | 0.12 | 0.01 | 0.01 |
| 84 | 10,000 | Jackknife | L | 0.2 | 146.15 | 0.72 | 0.71 | 6.09 | 0.13 | 3.12 | 0.14 | 0.12 | 0.01 | 0.01 |
| 84 | 10,000 | Jackknife | L | 0.3 | 146.15 | 0.72 | 0.71 | 6.09 | 0.13 | 3.12 | 0.14 | 0.12 | 0.01 | 0.01 |
| 84 | 10,000 | Jackknife | L | 0.4 | 146.15 | 0.72 | 0.71 | 6.09 | 0.13 | 3.12 | 0.14 | 0.12 | 0.01 | 0.01 |
| 84 | 10,000 | Jackknife | L | 0.5 | 146.15 | 0.72 | 0.71 | 6.10 | 0.13 | 3.12 | 0.14 | 0.12 | 0.01 | 0.01 |
| 84 | 10,000 | Jackknife | L | 0.6 | 146.39 | 0.72 | 0.71 | 6.15 | 0.13 | 3.16 | 0.14 | 0.12 | 0.01 | 0.01 |
| 84 | 10,000 | Jackknife | L | 0.7 | 146.73 | 0.72 | 0.71 | 6.22 | 0.13 | 3.19 | 0.15 | 0.13 | 0.01 | 0.01 |
| 84 | 10,000 | Jackknife | L | 0.8 | 147.14 | 0.72 | 0.71 | 6.31 | 0.13 | 3.24 | 0.15 | 0.13 | 0.01 | 0.01 |
| 84 | 10,000 | Jackknife | L | 0.9 | 147.69 | 0.72 | 0.70 | 6.40 | 0.13 | 3.29 | 0.15 | 0.13 | 0.01 | 0.01 |
| 84 | 10,000 | Jackknife | L | 1 | 148.08 | 0.72 | 0.70 | 6.51 | 0.13 | 3.35 | 0.14 | 0.12 | 0.01 | 0.01 |
| 84 | 10,000 | Jackknife | L | 1.1 | 148.87 | 0.72 | 0.70 | 6.62 | 0.13 | 3.41 | 0.14 | 0.12 | 0.01 | 0.01 |
| 84 | 10,000 | Jackknife | L | 1.2 | 149.56 | 0.72 | 0.70 | 6.71 | 0.13 | 3.46 | 0.14 | 0.12 | 0.01 | 0.01 |
| 84 | 10,000 | Jackknife | L | 1.3 | 150.26 | 0.72 | 0.70 | 6.81 | 0.14 | 3.52 | 0.13 | 0.12 | 0.01 | 0.01 |
| 84 | 10,000 | Jackknife | L | 1.4 | 151.06 | 0.72 | 0.70 | 6.91 | 0.14 | 3.58 | 0.14 | 0.12 | 0.01 | 0.01 |
| 84 | 10,000 | Jackknife | L | 1.5 | 151.94 | 0.71 | 0.70 | 6.99 | 0.14 | 3.63 | 0.14 | 0.12 | 0.01 | 0.01 |
| 84 | 10,000 | Jackknife | L | 1.6 | 152.98 | 0.71 | 0.70 | 7.05 | 0.14 | 3.67 | 0.13 | 0.12 | 0.01 | 0.01 |
| 84 | 10,000 | Jackknife | L | 1.7 | 153.44 | 0.71 | 0.69 | 7.13 | 0.14 | 3.73 | 0.13 | 0.12 | 0.01 | 0.01 |
| 84 | 10,000 | Jackknife | L | 1.8 | 154.52 | 0.71 | 0.69 | 7.20 | 0.14 | 3.79 | 0.14 | 0.12 | 0.01 | 0.01 |
| 84 | 10,000 | Jackknife | L | 1.9 | 155.93 | 0.71 | 0.69 | 7.24 | 0.14 | 3.83 | 0.14 | 0.12 | 0.04 | 0.03 |
| 84 | 10,000 | Jackknife | L | 2 | 157.15 | 0.71 | 0.69 | 7.27 | 0.14 | 3.87 | 0.15 | 0.13 | 0.04 | 0.03 |
| 84 | 10,000 | Jackknife | H | 1.3 | 170.85 | 0.91 | 0.89 | 1.61 | 0.06 | 1.20 | 0.15 | 0.13 | 0.01 | 0.01 |
| 84 | 10,000 | Jackknife | H | 1.1 | 178.35 | 0.92 | 0.89 | 1.66 | 0.06 | 1.25 | 0.14 | 0.12 | 0.01 | 0.01 |
| 84 | 10,000 | Jackknife | LQHP | 1.1 | 207.17 | 0.92 | 0.89 | 1.69 | 0.06 | 1.28 | 0.18 | 0.15 | 0.01 | 0.01 |
| 84 | 10,000 | Jackknife | LQHPT | 0.9 | 325.76 | 0.95 | 0.90 | 1.33 | 0.06 | 1.15 | 0.23 | 0.18 | 0.02 | 0.02 |
| 84 | 10,000 | Jackknife | LQH | 1.1 | 391.36 | 0.92 | 0.89 | 1.70 | 0.06 | 1.29 | 0.15 | 0.13 | 0.01 | 0.01 |
| 84 | 10,000 | Jackknife | LQHP | 1 | 484.96 | 0.92 | 0.89 | 1.69 | 0.06 | 1.30 | 0.18 | 0.15 | 0.01 | 0.01 |
| 84 | 10,000 | Jackknife | H | 1 | 571.63 | 0.92 | 0.89 | 1.68 | 0.06 | 1.28 | 0.17 | 0.14 | 0.01 | 0.01 |
| 84 | 10,000 | Jackknife | LQH | 1 | 623.20 | 0.92 | 0.89 | 1.70 | 0.06 | 1.30 | 0.19 | 0.16 | 0.01 | 0.01 |
| 84 | 10,000 | Jackknife | H | 0.9 | 740.69 | 0.92 | 0.89 | 1.70 | 0.06 | 1.31 | 0.18 | 0.15 | 0.01 | 0.01 |
| 84 | 10,000 | Jackknife | LQHPT | 0.8 | 807.34 | 0.96 | 0.90 | 1.37 | 0.07 | 1.21 | 0.23 | 0.18 | 0.02 | 0.02 |
| 84 | 10,000 | Jackknife | LQHP | 0.9 | 1138.93 | 0.92 | 0.89 | 1.72 | 0.06 | 1.34 | 0.18 | 0.15 | 0.01 | 0.01 |
| 84 | 10,000 | Jackknife | LQH | 0.9 | 2068.66 | 0.92 | 0.89 | 1.72 | 0.06 | 1.34 | 0.17 | 0.14 | 0.01 | 0.01 |
| 84 | 10,000 | Jackknife | LQHPT | 0.7 | 6599.86 | 0.96 | 0.90 | 1.41 | 0.07 | 1.28 | 0.24 | 0.18 | 0.05 | 0.05 |
| 84 | 10,000 | Jackknife | H | 0.1 | NA | 0.96 | 0.91 | 1.64 | 0.06 | 1.48 | 0.23 | 0.18 | 0.04 | 0.03 |
| 84 | 10,000 | Jackknife | LQH | 0.1 | NA | 0.96 | 0.91 | 1.63 | 0.06 | 1.48 | 0.23 | 0.18 | 0.02 | 0.02 |
| 84 | 10,000 | Jackknife | LQHP | 0.1 | NA | 0.96 | 0.91 | 1.68 | 0.06 | 1.52 | 0.23 | 0.18 | 0.02 | 0.02 |
| 84 | 10,000 | Jackknife | LQHPT | 0.1 | NA | 0.99 | 0.89 | 2.02 | 0.10 | 2.02 | 0.81 | 0.16 | 0.62 | 0.24 |
| 84 | 10,000 | Jackknife | H | 0.2 | NA | 0.95 | 0.91 | 1.63 | 0.06 | 1.44 | 0.19 | 0.16 | 0.02 | 0.02 |
| 84 | 10,000 | Jackknife | LQH | 0.2 | NA | 0.95 | 0.91 | 1.64 | 0.06 | 1.45 | 0.18 | 0.15 | 0.02 | 0.02 |
| 84 | 10,000 | Jackknife | LQHP | 0.2 | NA | 0.95 | 0.91 | 1.65 | 0.06 | 1.46 | 0.18 | 0.15 | 0.02 | 0.02 |
| 84 | 10,000 | Jackknife | LQHPT | 0.2 | NA | 0.99 | 0.90 | 1.82 | 0.09 | 1.81 | 0.68 | 0.22 | 0.55 | 0.25 |
| 84 | 10,000 | Jackknife | H | 0.3 | NA | 0.95 | 0.91 | 1.71 | 0.06 | 1.48 | 0.17 | 0.14 | 0.02 | 0.02 |
| 84 | 10,000 | Jackknife | LQH | 0.3 | NA | 0.95 | 0.91 | 1.73 | 0.06 | 1.50 | 0.17 | 0.14 | 0.02 | 0.02 |
| 84 | 10,000 | Jackknife | LQHP | 0.3 | NA | 0.95 | 0.91 | 1.73 | 0.06 | 1.50 | 0.17 | 0.14 | 0.02 | 0.02 |
| 84 | 10,000 | Jackknife | LQHPT | 0.3 | NA | 0.98 | 0.90 | 1.73 | 0.08 | 1.72 | 0.57 | 0.25 | 0.25 | 0.19 |
| 84 | 10,000 | Jackknife | H | 0.4 | NA | 0.94 | 0.90 | 1.73 | 0.06 | 1.47 | 0.15 | 0.13 | 0.02 | 0.02 |
| 84 | 10,000 | Jackknife | LQH | 0.4 | NA | 0.94 | 0.90 | 1.73 | 0.06 | 1.47 | 0.15 | 0.13 | 0.02 | 0.02 |
| 84 | 10,000 | Jackknife | LQHP | 0.4 | NA | 0.94 | 0.90 | 1.76 | 0.06 | 1.50 | 0.15 | 0.13 | 0.02 | 0.02 |
| 84 | 10,000 | Jackknife | LQHPT | 0.4 | NA | 0.98 | 0.90 | 1.64 | 0.08 | 1.60 | 0.49 | 0.25 | 0.13 | 0.12 |
| 84 | 10,000 | Jackknife | H | 0.5 | NA | 0.94 | 0.90 | 1.74 | 0.06 | 1.45 | 0.15 | 0.13 | 0.02 | 0.02 |
| 84 | 10,000 | Jackknife | LQH | 0.5 | NA | 0.94 | 0.90 | 1.73 | 0.06 | 1.45 | 0.15 | 0.13 | 0.02 | 0.02 |
| 84 | 10,000 | Jackknife | LQHP | 0.5 | NA | 0.94 | 0.90 | 1.80 | 0.06 | 1.51 | 0.17 | 0.14 | 0.02 | 0.02 |
| 84 | 10,000 | Jackknife | LQHPT | 0.5 | NA | 0.97 | 0.91 | 1.54 | 0.07 | 1.48 | 0.38 | 0.24 | 0.08 | 0.08 |
| 84 | 10,000 | Jackknife | H | 0.6 | NA | 0.93 | 0.90 | 1.72 | 0.06 | 1.42 | 0.15 | 0.13 | 0.01 | 0.01 |
| 84 | 10,000 | Jackknife | LQH | 0.6 | NA | 0.93 | 0.90 | 1.72 | 0.06 | 1.42 | 0.17 | 0.14 | 0.01 | 0.01 |
| 84 | 10,000 | Jackknife | LQHP | 0.6 | NA | 0.93 | 0.90 | 1.78 | 0.06 | 1.47 | 0.17 | 0.14 | 0.01 | 0.01 |
| 84 | 10,000 | Jackknife | LQHPT | 0.6 | NA | 0.97 | 0.90 | 1.48 | 0.07 | 1.39 | 0.30 | 0.21 | 0.06 | 0.06 |
| 84 | 10,000 | Jackknife | H | 0.7 | NA | 0.93 | 0.89 | 1.72 | 0.06 | 1.39 | 0.18 | 0.15 | 0.01 | 0.01 |
| 84 | 10,000 | Jackknife | LQH | 0.7 | NA | 0.93 | 0.89 | 1.73 | 0.06 | 1.41 | 0.19 | 0.16 | 0.01 | 0.01 |
| 84 | 10,000 | Jackknife | LQHP | 0.7 | NA | 0.93 | 0.89 | 1.77 | 0.06 | 1.44 | 0.19 | 0.16 | 0.01 | 0.01 |
| 84 | 10,000 | Jackknife | H | 0.8 | NA | 0.92 | 0.89 | 1.71 | 0.06 | 1.35 | 0.18 | 0.15 | 0.01 | 0.01 |
| 84 | 10,000 | Jackknife | LQH | 0.8 | NA | 0.92 | 0.89 | 1.73 | 0.06 | 1.37 | 0.19 | 0.16 | 0.01 | 0.01 |
| 84 | 10,000 | Jackknife | LQHP | 0.8 | NA | 0.92 | 0.89 | 1.74 | 0.06 | 1.38 | 0.18 | 0.15 | 0.01 | 0.01 |
| 84 | 10,000 | Random-k-fold | LQ | 0.9 | 0.00 | 0.89 | 0.87 | 0.00 | 0.02 | 0.00 | 0.12 | 0.00 | 0.02 | 0.00 |
| 84 | 10,000 | Random-k-fold | LQ | 0.1 | 0.81 | 0.90 | 0.88 | 0.00 | 0.02 | 0.00 | 0.13 | 0.00 | 0.02 | 0.00 |
| 84 | 10,000 | Random-k-fold | LQ | 0.2 | 0.81 | 0.90 | 0.88 | 0.00 | 0.02 | 0.00 | 0.13 | 0.00 | 0.02 | 0.00 |
| 84 | 10,000 | Random-k-fold | LQ | 0.3 | 0.81 | 0.90 | 0.88 | 0.00 | 0.02 | 0.00 | 0.13 | 0.00 | 0.02 | 0.00 |
| 84 | 10,000 | Random-k-fold | LQ | 0.4 | 1.18 | 0.90 | 0.88 | 0.00 | 0.02 | 0.00 | 0.13 | 0.00 | 0.02 | 0.00 |
| 84 | 10,000 | Random-k-fold | LQ | 0.7 | 1.23 | 0.89 | 0.87 | 0.00 | 0.02 | 0.00 | 0.12 | 0.00 | 0.02 | 0.00 |
| 84 | 10,000 | Random-k-fold | LQ | 0.6 | 1.71 | 0.89 | 0.87 | 0.00 | 0.02 | 0.00 | 0.12 | 0.00 | 0.02 | 0.00 |
| 84 | 10,000 | Random-k-fold | LQ | 0.5 | 2.21 | 0.90 | 0.88 | 0.00 | 0.02 | 0.00 | 0.12 | 0.00 | 0.02 | 0.00 |
| 84 | 10,000 | Random-k-fold | LQ | 0.8 | 2.71 | 0.89 | 0.87 | 0.00 | 0.02 | 0.00 | 0.12 | 0.00 | 0.02 | 0.00 |
| 84 | 10,000 | Random-k-fold | LQ | 1 | 5.45 | 0.89 | 0.86 | 0.00 | 0.02 | 0.00 | 0.12 | 0.00 | 0.02 | 0.00 |
| 84 | 10,000 | Random-k-fold | LQ | 1.2 | 7.55 | 0.89 | 0.86 | 0.00 | 0.02 | 0.00 | 0.14 | 0.00 | 0.02 | 0.00 |
| 84 | 10,000 | Random-k-fold | LQ | 1.4 | 8.01 | 0.88 | 0.86 | 0.00 | 0.02 | 0.00 | 0.14 | 0.00 | 0.02 | 0.00 |
| 84 | 10,000 | Random-k-fold | LQ | 1.1 | 14.37 | 0.89 | 0.86 | 0.00 | 0.02 | 0.00 | 0.14 | 0.00 | 0.02 | 0.00 |
| 84 | 10,000 | Random-k-fold | LQ | 1.3 | 14.45 | 0.89 | 0.86 | 0.00 | 0.02 | 0.00 | 0.13 | 0.00 | 0.02 | 0.00 |
| 84 | 10,000 | Random-k-fold | LQ | 1.7 | 16.73 | 0.88 | 0.85 | 0.00 | 0.02 | 0.00 | 0.13 | 0.00 | 0.02 | 0.00 |
| 84 | 10,000 | Random-k-fold | LQ | 1.6 | 17.28 | 0.88 | 0.85 | 0.00 | 0.02 | 0.00 | 0.13 | 0.00 | 0.02 | 0.00 |
| 84 | 10,000 | Random-k-fold | LQ | 1.8 | 17.71 | 0.88 | 0.85 | 0.00 | 0.02 | 0.00 | 0.13 | 0.00 | 0.02 | 0.00 |
| 84 | 10,000 | Random-k-fold | LQ | 1.5 | 17.73 | 0.88 | 0.86 | 0.00 | 0.02 | 0.00 | 0.13 | 0.00 | 0.02 | 0.00 |
| 84 | 10,000 | Random-k-fold | LQ | 1.9 | 19.09 | 0.87 | 0.85 | 0.00 | 0.02 | 0.00 | 0.13 | 0.00 | 0.02 | 0.00 |
| 84 | 10,000 | Random-k-fold | LQ | 2 | 21.46 | 0.87 | 0.85 | 0.00 | 0.02 | 0.00 | 0.13 | 0.00 | 0.02 | 0.00 |
| 84 | 10,000 | Random-k-fold | LQH | 1.7 | 54.05 | 0.91 | 0.89 | 0.00 | 0.02 | 0.00 | 0.13 | 0.00 | 0.02 | 0.00 |
| 84 | 10,000 | Random-k-fold | LQH | 2 | 54.83 | 0.91 | 0.88 | 0.00 | 0.02 | 0.00 | 0.14 | 0.00 | 0.04 | 0.01 |
| 84 | 10,000 | Random-k-fold | LQHPT | 1.6 | 55.64 | 0.93 | 0.89 | 0.00 | 0.03 | 0.00 | 0.18 | 0.00 | 0.01 | 0.00 |
| 84 | 10,000 | Random-k-fold | LQH | 1.9 | 57.76 | 0.91 | 0.88 | 0.00 | 0.02 | 0.00 | 0.13 | 0.00 | 0.04 | 0.01 |
| 84 | 10,000 | Random-k-fold | LQHP | 1.8 | 61.96 | 0.91 | 0.89 | 0.00 | 0.02 | 0.00 | 0.12 | 0.00 | 0.02 | 0.00 |
| 84 | 10,000 | Random-k-fold | H | 1.7 | 63.46 | 0.91 | 0.89 | 0.00 | 0.02 | 0.00 | 0.14 | 0.00 | 0.02 | 0.00 |
| 84 | 10,000 | Random-k-fold | LQHP | 1.7 | 66.63 | 0.91 | 0.89 | 0.00 | 0.02 | 0.00 | 0.13 | 0.00 | 0.02 | 0.00 |
| 84 | 10,000 | Random-k-fold | H | 1.9 | 66.83 | 0.91 | 0.89 | 0.00 | 0.02 | 0.00 | 0.14 | 0.00 | 0.02 | 0.00 |
| 84 | 10,000 | Random-k-fold | LQHPT | 1.7 | 67.39 | 0.93 | 0.89 | 0.00 | 0.03 | 0.00 | 0.18 | 0.00 | 0.01 | 0.00 |
| 84 | 10,000 | Random-k-fold | LQH | 1.4 | 67.78 | 0.91 | 0.89 | 0.00 | 0.02 | 0.00 | 0.13 | 0.00 | 0.02 | 0.00 |
| 84 | 10,000 | Random-k-fold | H | 2 | 68.58 | 0.91 | 0.89 | 0.00 | 0.02 | 0.00 | 0.12 | 0.00 | 0.02 | 0.00 |
| 84 | 10,000 | Random-k-fold | LQH | 1.5 | 69.54 | 0.91 | 0.89 | 0.00 | 0.02 | 0.00 | 0.13 | 0.00 | 0.02 | 0.00 |
| 84 | 10,000 | Random-k-fold | H | 1.8 | 71.51 | 0.91 | 0.89 | 0.00 | 0.02 | 0.00 | 0.15 | 0.00 | 0.02 | 0.00 |
| 84 | 10,000 | Random-k-fold | LQH | 1.6 | 71.62 | 0.91 | 0.89 | 0.00 | 0.02 | 0.00 | 0.13 | 0.00 | 0.02 | 0.00 |
| 84 | 10,000 | Random-k-fold | LQHPT | 1.9 | 75.76 | 0.93 | 0.89 | 0.00 | 0.03 | 0.00 | 0.19 | 0.00 | 0.01 | 0.00 |
| 84 | 10,000 | Random-k-fold | LQHPT | 1.2 | 76.43 | 0.94 | 0.90 | 0.00 | 0.03 | 0.00 | 0.20 | 0.00 | 0.01 | 0.00 |
| 84 | 10,000 | Random-k-fold | LQHPT | 2 | 80.36 | 0.92 | 0.89 | 0.00 | 0.03 | 0.00 | 0.18 | 0.01 | 0.01 | 0.00 |
| 84 | 10,000 | Random-k-fold | H | 1.5 | 80.56 | 0.91 | 0.89 | 0.00 | 0.02 | 0.00 | 0.15 | 0.00 | 0.02 | 0.00 |
| 84 | 10,000 | Random-k-fold | H | 1.6 | 82.05 | 0.91 | 0.89 | 0.00 | 0.02 | 0.00 | 0.14 | 0.00 | 0.02 | 0.00 |
| 84 | 10,000 | Random-k-fold | LQHP | 2 | 84.64 | 0.91 | 0.89 | 0.00 | 0.02 | 0.00 | 0.13 | 0.01 | 0.04 | 0.01 |
| 84 | 10,000 | Random-k-fold | LQHPT | 1.3 | 85.66 | 0.94 | 0.90 | 0.00 | 0.03 | 0.00 | 0.20 | 0.00 | 0.01 | 0.00 |
| 84 | 10,000 | Random-k-fold | LQH | 1.8 | 88.35 | 0.91 | 0.89 | 0.00 | 0.02 | 0.00 | 0.13 | 0.00 | 0.04 | 0.01 |
| 84 | 10,000 | Random-k-fold | LQHPT | 1.8 | 88.38 | 0.93 | 0.89 | 0.00 | 0.03 | 0.00 | 0.19 | 0.00 | 0.01 | 0.00 |
| 84 | 10,000 | Random-k-fold | LQHP | 1.9 | 90.10 | 0.91 | 0.89 | 0.00 | 0.02 | 0.00 | 0.12 | 0.00 | 0.02 | 0.00 |
| 84 | 10,000 | Random-k-fold | LQHP | 1.6 | 92.58 | 0.91 | 0.89 | 0.00 | 0.02 | 0.00 | 0.14 | 0.00 | 0.02 | 0.00 |
| 84 | 10,000 | Random-k-fold | H | 1.4 | 94.25 | 0.91 | 0.89 | 0.00 | 0.02 | 0.00 | 0.15 | 0.00 | 0.02 | 0.00 |
| 84 | 10,000 | Random-k-fold | LQHP | 1.5 | 98.82 | 0.91 | 0.89 | 0.00 | 0.02 | 0.00 | 0.13 | 0.00 | 0.02 | 0.00 |
| 84 | 10,000 | Random-k-fold | LQHPT | 1.5 | 101.47 | 0.93 | 0.90 | 0.00 | 0.03 | 0.00 | 0.18 | 0.00 | 0.01 | 0.00 |
| 84 | 10,000 | Random-k-fold | LQHPT | 1.4 | 104.32 | 0.94 | 0.90 | 0.00 | 0.03 | 0.00 | 0.20 | 0.00 | 0.01 | 0.00 |
| 84 | 10,000 | Random-k-fold | LQHPT | 1.1 | 110.31 | 0.95 | 0.90 | 0.00 | 0.03 | 0.00 | 0.18 | 0.01 | 0.01 | 0.00 |
| 84 | 10,000 | Random-k-fold | LQH | 1.3 | 111.87 | 0.91 | 0.89 | 0.00 | 0.02 | 0.00 | 0.14 | 0.00 | 0.02 | 0.00 |
| 84 | 10,000 | Random-k-fold | LQHP | 1.3 | 121.13 | 0.91 | 0.89 | 0.00 | 0.02 | 0.00 | 0.15 | 0.00 | 0.02 | 0.00 |
| 84 | 10,000 | Random-k-fold | LQHP | 1.4 | 122.68 | 0.91 | 0.89 | 0.00 | 0.02 | 0.00 | 0.14 | 0.00 | 0.02 | 0.00 |
| 84 | 10,000 | Random-k-fold | LQHP | 1.2 | 127.86 | 0.92 | 0.89 | 0.00 | 0.02 | 0.00 | 0.15 | 0.00 | 0.02 | 0.00 |
| 84 | 10,000 | Random-k-fold | LQH | 1.2 | 127.86 | 0.91 | 0.89 | 0.00 | 0.02 | 0.00 | 0.13 | 0.00 | 0.02 | 0.00 |
| 84 | 10,000 | Random-k-fold | H | 1.2 | 135.98 | 0.91 | 0.89 | 0.00 | 0.02 | 0.00 | 0.15 | 0.00 | 0.02 | 0.00 |
| 84 | 10,000 | Random-k-fold | LQHPT | 1 | 139.22 | 0.95 | 0.90 | 0.00 | 0.04 | 0.00 | 0.18 | 0.01 | 0.01 | 0.00 |
| 84 | 10,000 | Random-k-fold | L | 0.1 | 146.15 | 0.72 | 0.71 | 0.01 | 0.03 | 0.01 | 0.13 | 0.01 | 0.01 | 0.00 |
| 84 | 10,000 | Random-k-fold | L | 0.2 | 146.15 | 0.72 | 0.71 | 0.01 | 0.03 | 0.01 | 0.13 | 0.01 | 0.01 | 0.00 |
| 84 | 10,000 | Random-k-fold | L | 0.3 | 146.15 | 0.72 | 0.71 | 0.01 | 0.03 | 0.01 | 0.14 | 0.01 | 0.01 | 0.00 |
| 84 | 10,000 | Random-k-fold | L | 0.4 | 146.15 | 0.72 | 0.71 | 0.01 | 0.03 | 0.01 | 0.14 | 0.01 | 0.01 | 0.00 |
| 84 | 10,000 | Random-k-fold | L | 0.5 | 146.15 | 0.72 | 0.70 | 0.01 | 0.03 | 0.01 | 0.13 | 0.01 | 0.01 | 0.00 |
| 84 | 10,000 | Random-k-fold | L | 0.6 | 146.39 | 0.72 | 0.70 | 0.01 | 0.03 | 0.01 | 0.13 | 0.01 | 0.01 | 0.00 |
| 84 | 10,000 | Random-k-fold | L | 0.7 | 146.73 | 0.72 | 0.70 | 0.01 | 0.03 | 0.01 | 0.14 | 0.01 | 0.01 | 0.00 |
| 84 | 10,000 | Random-k-fold | L | 0.8 | 147.14 | 0.72 | 0.70 | 0.01 | 0.03 | 0.01 | 0.15 | 0.01 | 0.01 | 0.00 |
| 84 | 10,000 | Random-k-fold | L | 0.9 | 147.69 | 0.72 | 0.70 | 0.01 | 0.03 | 0.01 | 0.15 | 0.01 | 0.01 | 0.00 |
| 84 | 10,000 | Random-k-fold | L | 1 | 148.08 | 0.72 | 0.70 | 0.01 | 0.03 | 0.01 | 0.15 | 0.01 | 0.01 | 0.00 |
| 84 | 10,000 | Random-k-fold | L | 1.1 | 148.87 | 0.72 | 0.69 | 0.01 | 0.03 | 0.01 | 0.17 | 0.01 | 0.00 | 0.00 |
| 84 | 10,000 | Random-k-fold | L | 1.2 | 149.56 | 0.72 | 0.69 | 0.01 | 0.03 | 0.01 | 0.17 | 0.01 | 0.00 | 0.00 |
| 84 | 10,000 | Random-k-fold | L | 1.3 | 150.26 | 0.72 | 0.68 | 0.01 | 0.04 | 0.01 | 0.17 | 0.01 | 0.00 | 0.00 |
| 84 | 10,000 | Random-k-fold | L | 1.4 | 151.06 | 0.72 | 0.68 | 0.01 | 0.04 | 0.01 | 0.15 | 0.01 | 0.00 | 0.00 |
| 84 | 10,000 | Random-k-fold | L | 1.5 | 151.94 | 0.71 | 0.68 | 0.01 | 0.04 | 0.01 | 0.13 | 0.02 | 0.01 | 0.00 |
| 84 | 10,000 | Random-k-fold | L | 1.6 | 152.98 | 0.71 | 0.68 | 0.01 | 0.04 | 0.01 | 0.13 | 0.02 | 0.01 | 0.00 |
| 84 | 10,000 | Random-k-fold | L | 1.7 | 153.44 | 0.71 | 0.67 | 0.01 | 0.04 | 0.01 | 0.13 | 0.02 | 0.01 | 0.00 |
| 84 | 10,000 | Random-k-fold | L | 1.8 | 154.52 | 0.71 | 0.67 | 0.01 | 0.04 | 0.01 | 0.13 | 0.02 | 0.01 | 0.00 |
| 84 | 10,000 | Random-k-fold | L | 1.9 | 155.93 | 0.71 | 0.67 | 0.01 | 0.04 | 0.01 | 0.13 | 0.02 | 0.01 | 0.00 |
| 84 | 10,000 | Random-k-fold | L | 2 | 157.15 | 0.71 | 0.67 | 0.01 | 0.04 | 0.01 | 0.12 | 0.01 | 0.01 | 0.00 |
| 84 | 10,000 | Random-k-fold | H | 1.3 | 170.85 | 0.91 | 0.89 | 0.00 | 0.02 | 0.00 | 0.15 | 0.00 | 0.02 | 0.00 |
| 84 | 10,000 | Random-k-fold | H | 1.1 | 178.35 | 0.92 | 0.89 | 0.00 | 0.02 | 0.00 | 0.14 | 0.00 | 0.02 | 0.00 |
| 84 | 10,000 | Random-k-fold | LQHP | 1.1 | 207.17 | 0.92 | 0.89 | 0.00 | 0.02 | 0.00 | 0.15 | 0.00 | 0.02 | 0.00 |
| 84 | 10,000 | Random-k-fold | LQHPT | 0.9 | 325.76 | 0.95 | 0.91 | 0.00 | 0.04 | 0.00 | 0.21 | 0.01 | 0.01 | 0.00 |
| 84 | 10,000 | Random-k-fold | LQH | 1.1 | 391.36 | 0.92 | 0.89 | 0.00 | 0.02 | 0.00 | 0.14 | 0.00 | 0.02 | 0.00 |
| 84 | 10,000 | Random-k-fold | LQHP | 1 | 484.96 | 0.92 | 0.89 | 0.00 | 0.02 | 0.00 | 0.15 | 0.01 | 0.02 | 0.00 |
| 84 | 10,000 | Random-k-fold | H | 1 | 571.63 | 0.92 | 0.89 | 0.00 | 0.02 | 0.00 | 0.15 | 0.01 | 0.02 | 0.00 |
| 84 | 10,000 | Random-k-fold | LQH | 1 | 623.20 | 0.92 | 0.89 | 0.00 | 0.03 | 0.00 | 0.15 | 0.00 | 0.02 | 0.00 |
| 84 | 10,000 | Random-k-fold | H | 0.9 | 740.69 | 0.92 | 0.90 | 0.00 | 0.02 | 0.00 | 0.15 | 0.01 | 0.02 | 0.00 |
| 84 | 10,000 | Random-k-fold | LQHPT | 0.8 | 807.34 | 0.96 | 0.91 | 0.00 | 0.04 | 0.00 | 0.21 | 0.01 | 0.01 | 0.00 |
| 84 | 10,000 | Random-k-fold | LQHP | 0.9 | 1138.93 | 0.92 | 0.90 | 0.00 | 0.02 | 0.00 | 0.15 | 0.01 | 0.02 | 0.00 |
| 84 | 10,000 | Random-k-fold | LQH | 0.9 | 2068.66 | 0.92 | 0.89 | 0.00 | 0.03 | 0.00 | 0.15 | 0.01 | 0.02 | 0.00 |
| 84 | 10,000 | Random-k-fold | LQHPT | 0.7 | 6599.86 | 0.96 | 0.91 | 0.00 | 0.05 | 0.00 | 0.21 | 0.01 | 0.02 | 0.00 |
| 84 | 10,000 | Random-k-fold | H | 0.1 | NA | 0.96 | 0.91 | 0.00 | 0.05 | 0.00 | 0.20 | 0.01 | 0.08 | 0.01 |
| 84 | 10,000 | Random-k-fold | LQH | 0.1 | NA | 0.96 | 0.91 | 0.00 | 0.05 | 0.00 | 0.23 | 0.00 | 0.08 | 0.01 |
| 84 | 10,000 | Random-k-fold | LQHP | 0.1 | NA | 0.96 | 0.91 | 0.00 | 0.05 | 0.00 | 0.25 | 0.00 | 0.08 | 0.01 |
| 84 | 10,000 | Random-k-fold | LQHPT | 0.1 | NA | 0.99 | 0.90 | 0.00 | 0.10 | 0.00 | 0.94 | 0.00 | 0.71 | 0.05 |
| 84 | 10,000 | Random-k-fold | H | 0.2 | NA | 0.95 | 0.91 | 0.00 | 0.04 | 0.00 | 0.17 | 0.01 | 0.07 | 0.01 |
| 84 | 10,000 | Random-k-fold | LQH | 0.2 | NA | 0.95 | 0.91 | 0.00 | 0.04 | 0.00 | 0.17 | 0.01 | 0.07 | 0.01 |
| 84 | 10,000 | Random-k-fold | LQHP | 0.2 | NA | 0.95 | 0.91 | 0.00 | 0.04 | 0.00 | 0.17 | 0.01 | 0.07 | 0.01 |
| 84 | 10,000 | Random-k-fold | LQHPT | 0.2 | NA | 0.99 | 0.90 | 0.00 | 0.09 | 0.00 | 0.83 | 0.00 | 0.50 | 0.02 |
| 84 | 10,000 | Random-k-fold | H | 0.3 | NA | 0.95 | 0.91 | 0.00 | 0.04 | 0.00 | 0.17 | 0.02 | 0.06 | 0.01 |
| 84 | 10,000 | Random-k-fold | LQH | 0.3 | NA | 0.95 | 0.91 | 0.00 | 0.04 | 0.00 | 0.15 | 0.01 | 0.06 | 0.01 |
| 84 | 10,000 | Random-k-fold | LQHP | 0.3 | NA | 0.95 | 0.91 | 0.00 | 0.04 | 0.00 | 0.17 | 0.02 | 0.06 | 0.01 |
| 84 | 10,000 | Random-k-fold | LQHPT | 0.3 | NA | 0.98 | 0.91 | 0.00 | 0.08 | 0.00 | 0.62 | 0.01 | 0.20 | 0.00 |
| 84 | 10,000 | Random-k-fold | H | 0.4 | NA | 0.94 | 0.91 | 0.00 | 0.04 | 0.00 | 0.17 | 0.00 | 0.06 | 0.01 |
| 84 | 10,000 | Random-k-fold | LQH | 0.4 | NA | 0.94 | 0.91 | 0.00 | 0.04 | 0.00 | 0.15 | 0.01 | 0.06 | 0.01 |
| 84 | 10,000 | Random-k-fold | LQHP | 0.4 | NA | 0.94 | 0.91 | 0.00 | 0.04 | 0.00 | 0.17 | 0.00 | 0.06 | 0.01 |
| 84 | 10,000 | Random-k-fold | LQHPT | 0.4 | NA | 0.98 | 0.91 | 0.00 | 0.07 | 0.00 | 0.40 | 0.02 | 0.19 | 0.00 |
| 84 | 10,000 | Random-k-fold | H | 0.5 | NA | 0.94 | 0.90 | 0.00 | 0.03 | 0.00 | 0.15 | 0.00 | 0.04 | 0.01 |
| 84 | 10,000 | Random-k-fold | LQH | 0.5 | NA | 0.94 | 0.90 | 0.00 | 0.03 | 0.00 | 0.17 | 0.00 | 0.04 | 0.01 |
| 84 | 10,000 | Random-k-fold | LQHP | 0.5 | NA | 0.94 | 0.90 | 0.00 | 0.03 | 0.00 | 0.17 | 0.00 | 0.04 | 0.01 |
| 84 | 10,000 | Random-k-fold | LQHPT | 0.5 | NA | 0.97 | 0.91 | 0.00 | 0.06 | 0.00 | 0.26 | 0.01 | 0.15 | 0.01 |
| 84 | 10,000 | Random-k-fold | H | 0.6 | NA | 0.93 | 0.90 | 0.00 | 0.03 | 0.00 | 0.18 | 0.01 | 0.04 | 0.01 |
| 84 | 10,000 | Random-k-fold | LQH | 0.6 | NA | 0.93 | 0.90 | 0.00 | 0.03 | 0.00 | 0.19 | 0.01 | 0.04 | 0.01 |
| 84 | 10,000 | Random-k-fold | LQHP | 0.6 | NA | 0.93 | 0.90 | 0.00 | 0.03 | 0.00 | 0.17 | 0.01 | 0.04 | 0.01 |
| 84 | 10,000 | Random-k-fold | LQHPT | 0.6 | NA | 0.97 | 0.91 | 0.00 | 0.05 | 0.00 | 0.23 | 0.01 | 0.07 | 0.01 |
| 84 | 10,000 | Random-k-fold | H | 0.7 | NA | 0.93 | 0.90 | 0.00 | 0.03 | 0.00 | 0.17 | 0.02 | 0.02 | 0.00 |
| 84 | 10,000 | Random-k-fold | LQH | 0.7 | NA | 0.93 | 0.90 | 0.00 | 0.03 | 0.00 | 0.15 | 0.01 | 0.02 | 0.00 |
| 84 | 10,000 | Random-k-fold | LQHP | 0.7 | NA | 0.93 | 0.90 | 0.00 | 0.03 | 0.00 | 0.15 | 0.01 | 0.02 | 0.00 |
| 84 | 10,000 | Random-k-fold | H | 0.8 | NA | 0.92 | 0.90 | 0.00 | 0.03 | 0.00 | 0.13 | 0.01 | 0.02 | 0.00 |
| 84 | 10,000 | Random-k-fold | LQH | 0.8 | NA | 0.92 | 0.90 | 0.00 | 0.03 | 0.00 | 0.13 | 0.01 | 0.02 | 0.00 |
| 84 | 10,000 | Random-k-fold | LQHP | 0.8 | NA | 0.92 | 0.90 | 0.00 | 0.03 | 0.00 | 0.13 | 0.01 | 0.02 | 0.00 |
| 84 | 10,000 | Block | LQ | 0.9 | 0.00 | 0.89 | 0.88 | 0.02 | 0.03 | 0.01 | 0.07 | 0.00 | 0.01 | 0.00 |
| 84 | 10,000 | Block | LQ | 0.1 | 0.81 | 0.90 | 0.88 | 0.02 | 0.03 | 0.01 | 0.08 | 0.01 | 0.01 | 0.00 |
| 84 | 10,000 | Block | LQ | 0.2 | 0.81 | 0.90 | 0.88 | 0.02 | 0.03 | 0.01 | 0.08 | 0.01 | 0.01 | 0.00 |
| 84 | 10,000 | Block | LQ | 0.3 | 0.81 | 0.90 | 0.88 | 0.02 | 0.03 | 0.01 | 0.10 | 0.01 | 0.01 | 0.00 |
| 84 | 10,000 | Block | LQ | 0.4 | 1.18 | 0.90 | 0.88 | 0.02 | 0.02 | 0.01 | 0.11 | 0.01 | 0.01 | 0.00 |
| 84 | 10,000 | Block | LQ | 0.7 | 1.23 | 0.89 | 0.88 | 0.02 | 0.03 | 0.01 | 0.10 | 0.00 | 0.01 | 0.00 |
| 84 | 10,000 | Block | LQ | 0.6 | 1.71 | 0.89 | 0.88 | 0.02 | 0.03 | 0.00 | 0.10 | 0.01 | 0.01 | 0.00 |
| 84 | 10,000 | Block | LQ | 0.5 | 2.21 | 0.90 | 0.88 | 0.02 | 0.03 | 0.01 | 0.11 | 0.01 | 0.01 | 0.00 |
| 84 | 10,000 | Block | LQ | 0.8 | 2.71 | 0.89 | 0.88 | 0.02 | 0.03 | 0.00 | 0.07 | 0.00 | 0.01 | 0.00 |
| 84 | 10,000 | Block | LQ | 1 | 5.45 | 0.89 | 0.87 | 0.02 | 0.03 | 0.01 | 0.07 | 0.00 | 0.01 | 0.00 |
| 84 | 10,000 | Block | LQ | 1.2 | 7.55 | 0.89 | 0.87 | 0.03 | 0.04 | 0.01 | 0.10 | 0.01 | 0.01 | 0.00 |
| 84 | 10,000 | Block | LQ | 1.4 | 8.01 | 0.88 | 0.86 | 0.03 | 0.04 | 0.01 | 0.11 | 0.01 | 0.01 | 0.00 |
| 84 | 10,000 | Block | LQ | 1.1 | 14.37 | 0.89 | 0.87 | 0.02 | 0.04 | 0.01 | 0.07 | 0.00 | 0.01 | 0.00 |
| 84 | 10,000 | Block | LQ | 1.3 | 14.45 | 0.89 | 0.86 | 0.03 | 0.04 | 0.01 | 0.10 | 0.01 | 0.01 | 0.00 |
| 84 | 10,000 | Block | LQ | 1.7 | 16.73 | 0.88 | 0.85 | 0.04 | 0.05 | 0.02 | 0.12 | 0.01 | 0.01 | 0.00 |
| 84 | 10,000 | Block | LQ | 1.6 | 17.28 | 0.88 | 0.85 | 0.04 | 0.05 | 0.02 | 0.12 | 0.01 | 0.01 | 0.00 |
| 84 | 10,000 | Block | LQ | 1.8 | 17.71 | 0.88 | 0.84 | 0.04 | 0.06 | 0.02 | 0.12 | 0.01 | 0.01 | 0.00 |
| 84 | 10,000 | Block | LQ | 1.5 | 17.73 | 0.88 | 0.85 | 0.03 | 0.05 | 0.01 | 0.11 | 0.01 | 0.01 | 0.00 |
| 84 | 10,000 | Block | LQ | 1.9 | 19.09 | 0.87 | 0.84 | 0.05 | 0.06 | 0.02 | 0.12 | 0.01 | 0.01 | 0.00 |
| 84 | 10,000 | Block | LQ | 2 | 21.46 | 0.87 | 0.83 | 0.05 | 0.06 | 0.02 | 0.13 | 0.01 | 0.01 | 0.00 |
| 84 | 10,000 | Block | LQH | 1.7 | 54.05 | 0.91 | 0.88 | 0.02 | 0.04 | 0.01 | 0.15 | 0.05 | 0.01 | 0.00 |
| 84 | 10,000 | Block | LQH | 2 | 54.83 | 0.91 | 0.87 | 0.02 | 0.04 | 0.01 | 0.18 | 0.05 | 0.02 | 0.00 |
| 84 | 10,000 | Block | LQHPT | 1.6 | 55.64 | 0.93 | 0.88 | 0.02 | 0.05 | 0.01 | 0.13 | 0.03 | 0.01 | 0.00 |
| 84 | 10,000 | Block | LQH | 1.9 | 57.76 | 0.91 | 0.87 | 0.02 | 0.04 | 0.01 | 0.15 | 0.05 | 0.02 | 0.00 |
| 84 | 10,000 | Block | LQHP | 1.8 | 61.96 | 0.91 | 0.88 | 0.02 | 0.03 | 0.01 | 0.13 | 0.03 | 0.01 | 0.00 |
| 84 | 10,000 | Block | H | 1.7 | 63.46 | 0.91 | 0.88 | 0.01 | 0.03 | 0.01 | 0.10 | 0.01 | 0.01 | 0.00 |
| 84 | 10,000 | Block | LQHP | 1.7 | 66.63 | 0.91 | 0.88 | 0.01 | 0.03 | 0.01 | 0.12 | 0.02 | 0.01 | 0.00 |
| 84 | 10,000 | Block | H | 1.9 | 66.83 | 0.91 | 0.88 | 0.01 | 0.03 | 0.01 | 0.11 | 0.02 | 0.01 | 0.00 |
| 84 | 10,000 | Block | LQHPT | 1.7 | 67.39 | 0.93 | 0.88 | 0.02 | 0.05 | 0.01 | 0.13 | 0.03 | 0.01 | 0.00 |
| 84 | 10,000 | Block | LQH | 1.4 | 67.78 | 0.91 | 0.88 | 0.02 | 0.04 | 0.02 | 0.14 | 0.05 | 0.01 | 0.00 |
| 84 | 10,000 | Block | H | 2 | 68.58 | 0.91 | 0.88 | 0.01 | 0.03 | 0.01 | 0.11 | 0.02 | 0.01 | 0.00 |
| 84 | 10,000 | Block | LQH | 1.5 | 69.54 | 0.91 | 0.88 | 0.02 | 0.04 | 0.01 | 0.14 | 0.05 | 0.01 | 0.00 |
| 84 | 10,000 | Block | H | 1.8 | 71.51 | 0.91 | 0.88 | 0.01 | 0.03 | 0.01 | 0.11 | 0.02 | 0.01 | 0.00 |
| 84 | 10,000 | Block | LQH | 1.6 | 71.62 | 0.91 | 0.88 | 0.02 | 0.04 | 0.01 | 0.15 | 0.05 | 0.01 | 0.00 |
| 84 | 10,000 | Block | LQHPT | 1.9 | 75.76 | 0.93 | 0.87 | 0.02 | 0.04 | 0.01 | 0.13 | 0.03 | 0.01 | 0.00 |
| 84 | 10,000 | Block | LQHPT | 1.2 | 76.43 | 0.94 | 0.88 | 0.02 | 0.05 | 0.01 | 0.17 | 0.04 | 0.02 | 0.00 |
| 84 | 10,000 | Block | LQHPT | 2 | 80.36 | 0.92 | 0.87 | 0.02 | 0.04 | 0.01 | 0.13 | 0.03 | 0.01 | 0.00 |
| 84 | 10,000 | Block | H | 1.5 | 80.56 | 0.91 | 0.89 | 0.01 | 0.03 | 0.01 | 0.10 | 0.01 | 0.01 | 0.00 |
| 84 | 10,000 | Block | H | 1.6 | 82.05 | 0.91 | 0.88 | 0.01 | 0.03 | 0.01 | 0.10 | 0.01 | 0.01 | 0.00 |
| 84 | 10,000 | Block | LQHP | 2 | 84.64 | 0.91 | 0.88 | 0.02 | 0.03 | 0.01 | 0.13 | 0.03 | 0.01 | 0.00 |
| 84 | 10,000 | Block | LQHPT | 1.3 | 85.66 | 0.94 | 0.88 | 0.02 | 0.05 | 0.01 | 0.14 | 0.03 | 0.01 | 0.00 |
| 84 | 10,000 | Block | LQH | 1.8 | 88.35 | 0.91 | 0.88 | 0.02 | 0.04 | 0.01 | 0.15 | 0.05 | 0.01 | 0.00 |
| 84 | 10,000 | Block | LQHPT | 1.8 | 88.38 | 0.93 | 0.87 | 0.02 | 0.04 | 0.01 | 0.13 | 0.03 | 0.01 | 0.00 |
| 84 | 10,000 | Block | LQHP | 1.9 | 90.10 | 0.91 | 0.88 | 0.01 | 0.03 | 0.01 | 0.12 | 0.02 | 0.01 | 0.00 |
| 84 | 10,000 | Block | LQHP | 1.6 | 92.58 | 0.91 | 0.88 | 0.01 | 0.03 | 0.01 | 0.12 | 0.02 | 0.01 | 0.00 |
| 84 | 10,000 | Block | H | 1.4 | 94.25 | 0.91 | 0.88 | 0.01 | 0.03 | 0.01 | 0.11 | 0.02 | 0.01 | 0.00 |
| 84 | 10,000 | Block | LQHP | 1.5 | 98.82 | 0.91 | 0.88 | 0.01 | 0.03 | 0.01 | 0.12 | 0.02 | 0.01 | 0.00 |
| 84 | 10,000 | Block | LQHPT | 1.5 | 101.47 | 0.93 | 0.88 | 0.02 | 0.05 | 0.01 | 0.14 | 0.03 | 0.01 | 0.00 |
| 84 | 10,000 | Block | LQHPT | 1.4 | 104.32 | 0.94 | 0.88 | 0.02 | 0.05 | 0.01 | 0.14 | 0.03 | 0.01 | 0.00 |
| 84 | 10,000 | Block | LQHPT | 1.1 | 110.31 | 0.95 | 0.88 | 0.02 | 0.06 | 0.01 | 0.17 | 0.04 | 0.05 | 0.00 |
| 84 | 10,000 | Block | LQH | 1.3 | 111.87 | 0.91 | 0.88 | 0.02 | 0.04 | 0.01 | 0.14 | 0.05 | 0.01 | 0.00 |
| 84 | 10,000 | Block | LQHP | 1.3 | 121.13 | 0.91 | 0.88 | 0.01 | 0.03 | 0.01 | 0.12 | 0.03 | 0.01 | 0.00 |
| 84 | 10,000 | Block | LQHP | 1.4 | 122.68 | 0.91 | 0.88 | 0.01 | 0.03 | 0.01 | 0.11 | 0.02 | 0.01 | 0.00 |
| 84 | 10,000 | Block | LQHP | 1.2 | 127.86 | 0.92 | 0.88 | 0.01 | 0.04 | 0.01 | 0.14 | 0.05 | 0.01 | 0.00 |
| 84 | 10,000 | Block | LQH | 1.2 | 127.86 | 0.91 | 0.88 | 0.02 | 0.04 | 0.01 | 0.14 | 0.05 | 0.01 | 0.00 |
| 84 | 10,000 | Block | H | 1.2 | 135.98 | 0.91 | 0.88 | 0.01 | 0.04 | 0.01 | 0.14 | 0.05 | 0.01 | 0.00 |
| 84 | 10,000 | Block | LQHPT | 1 | 139.22 | 0.95 | 0.88 | 0.02 | 0.06 | 0.01 | 0.17 | 0.04 | 0.05 | 0.00 |
| 84 | 10,000 | Block | L | 0.1 | 146.15 | 0.72 | 0.67 | 0.14 | 0.12 | 0.16 | 0.27 | 0.16 | 0.11 | 0.03 |
| 84 | 10,000 | Block | L | 0.2 | 146.15 | 0.72 | 0.67 | 0.14 | 0.12 | 0.16 | 0.27 | 0.16 | 0.11 | 0.03 |
| 84 | 10,000 | Block | L | 0.3 | 146.15 | 0.72 | 0.67 | 0.14 | 0.12 | 0.16 | 0.27 | 0.16 | 0.11 | 0.03 |
| 84 | 10,000 | Block | L | 0.4 | 146.15 | 0.72 | 0.67 | 0.14 | 0.12 | 0.16 | 0.27 | 0.16 | 0.12 | 0.04 |
| 84 | 10,000 | Block | L | 0.5 | 146.15 | 0.72 | 0.66 | 0.14 | 0.12 | 0.16 | 0.27 | 0.16 | 0.12 | 0.04 |
| 84 | 10,000 | Block | L | 0.6 | 146.39 | 0.72 | 0.66 | 0.13 | 0.12 | 0.16 | 0.27 | 0.16 | 0.12 | 0.04 |
| 84 | 10,000 | Block | L | 0.7 | 146.73 | 0.72 | 0.66 | 0.13 | 0.12 | 0.16 | 0.27 | 0.16 | 0.12 | 0.04 |
| 84 | 10,000 | Block | L | 0.8 | 147.14 | 0.72 | 0.65 | 0.13 | 0.11 | 0.16 | 0.27 | 0.16 | 0.14 | 0.07 |
| 84 | 10,000 | Block | L | 0.9 | 147.69 | 0.72 | 0.65 | 0.12 | 0.11 | 0.15 | 0.27 | 0.16 | 0.14 | 0.07 |
| 84 | 10,000 | Block | L | 1 | 148.08 | 0.72 | 0.64 | 0.11 | 0.11 | 0.15 | 0.27 | 0.16 | 0.18 | 0.07 |
| 84 | 10,000 | Block | L | 1.1 | 148.87 | 0.72 | 0.64 | 0.11 | 0.11 | 0.14 | 0.27 | 0.16 | 0.18 | 0.07 |
| 84 | 10,000 | Block | L | 1.2 | 149.56 | 0.72 | 0.63 | 0.10 | 0.11 | 0.14 | 0.27 | 0.16 | 0.17 | 0.06 |
| 84 | 10,000 | Block | L | 1.3 | 150.26 | 0.72 | 0.62 | 0.10 | 0.11 | 0.14 | 0.27 | 0.16 | 0.18 | 0.07 |
| 84 | 10,000 | Block | L | 1.4 | 151.06 | 0.72 | 0.62 | 0.09 | 0.11 | 0.14 | 0.27 | 0.16 | 0.17 | 0.06 |
| 84 | 10,000 | Block | L | 1.5 | 151.94 | 0.71 | 0.62 | 0.09 | 0.11 | 0.14 | 0.27 | 0.16 | 0.17 | 0.06 |
| 84 | 10,000 | Block | L | 1.6 | 152.98 | 0.71 | 0.62 | 0.09 | 0.11 | 0.14 | 0.29 | 0.16 | 0.17 | 0.06 |
| 84 | 10,000 | Block | L | 1.7 | 153.44 | 0.71 | 0.61 | 0.09 | 0.11 | 0.14 | 0.29 | 0.16 | 0.17 | 0.06 |
| 84 | 10,000 | Block | L | 1.8 | 154.52 | 0.71 | 0.61 | 0.09 | 0.11 | 0.14 | 0.29 | 0.16 | 0.15 | 0.05 |
| 84 | 10,000 | Block | L | 1.9 | 155.93 | 0.71 | 0.61 | 0.09 | 0.11 | 0.14 | 0.29 | 0.16 | 0.14 | 0.04 |
| 84 | 10,000 | Block | L | 2 | 157.15 | 0.71 | 0.61 | 0.09 | 0.11 | 0.14 | 0.29 | 0.16 | 0.13 | 0.03 |
| 84 | 10,000 | Block | H | 1.3 | 170.85 | 0.91 | 0.89 | 0.01 | 0.04 | 0.01 | 0.13 | 0.04 | 0.01 | 0.00 |
| 84 | 10,000 | Block | H | 1.1 | 178.35 | 0.92 | 0.88 | 0.02 | 0.04 | 0.01 | 0.14 | 0.05 | 0.01 | 0.00 |
| 84 | 10,000 | Block | LQHP | 1.1 | 207.17 | 0.92 | 0.88 | 0.01 | 0.04 | 0.01 | 0.14 | 0.05 | 0.01 | 0.00 |
| 84 | 10,000 | Block | LQHPT | 0.9 | 325.76 | 0.95 | 0.88 | 0.02 | 0.06 | 0.01 | 0.18 | 0.04 | 0.05 | 0.00 |
| 84 | 10,000 | Block | LQH | 1.1 | 391.36 | 0.92 | 0.88 | 0.01 | 0.04 | 0.01 | 0.14 | 0.05 | 0.01 | 0.00 |
| 84 | 10,000 | Block | LQHP | 1 | 484.96 | 0.92 | 0.88 | 0.01 | 0.04 | 0.01 | 0.14 | 0.05 | 0.01 | 0.00 |
| 84 | 10,000 | Block | H | 1 | 571.63 | 0.92 | 0.88 | 0.01 | 0.04 | 0.01 | 0.14 | 0.05 | 0.01 | 0.00 |
| 84 | 10,000 | Block | LQH | 1 | 623.20 | 0.92 | 0.88 | 0.01 | 0.04 | 0.01 | 0.14 | 0.05 | 0.01 | 0.00 |
| 84 | 10,000 | Block | H | 0.9 | 740.69 | 0.92 | 0.88 | 0.01 | 0.05 | 0.02 | 0.14 | 0.05 | 0.02 | 0.00 |
| 84 | 10,000 | Block | LQHPT | 0.8 | 807.34 | 0.96 | 0.88 | 0.01 | 0.06 | 0.01 | 0.20 | 0.05 | 0.05 | 0.00 |
| 84 | 10,000 | Block | LQHP | 0.9 | 1138.93 | 0.92 | 0.88 | 0.01 | 0.04 | 0.02 | 0.14 | 0.05 | 0.02 | 0.00 |
| 84 | 10,000 | Block | LQH | 0.9 | 2068.66 | 0.92 | 0.88 | 0.01 | 0.04 | 0.01 | 0.14 | 0.05 | 0.01 | 0.00 |
| 84 | 10,000 | Block | LQHPT | 0.7 | 6599.86 | 0.96 | 0.89 | 0.01 | 0.06 | 0.01 | 0.27 | 0.07 | 0.05 | 0.00 |
| 84 | 10,000 | Block | H | 0.1 | NA | 0.96 | 0.82 | 0.02 | 0.13 | 0.02 | 0.54 | 0.06 | 0.23 | 0.06 |
| 84 | 10,000 | Block | LQH | 0.1 | NA | 0.96 | 0.82 | 0.02 | 0.13 | 0.02 | 0.54 | 0.04 | 0.25 | 0.07 |
| 84 | 10,000 | Block | LQHP | 0.1 | NA | 0.96 | 0.82 | 0.02 | 0.13 | 0.02 | 0.51 | 0.05 | 0.25 | 0.07 |
| 84 | 10,000 | Block | LQHPT | 0.1 | NA | 0.99 | 0.87 | 0.00 | 0.12 | 0.00 | 0.88 | 0.02 | 0.76 | 0.04 |
| 84 | 10,000 | Block | H | 0.2 | NA | 0.95 | 0.84 | 0.02 | 0.11 | 0.03 | 0.48 | 0.08 | 0.21 | 0.06 |
| 84 | 10,000 | Block | LQH | 0.2 | NA | 0.95 | 0.84 | 0.02 | 0.11 | 0.02 | 0.46 | 0.07 | 0.21 | 0.06 |
| 84 | 10,000 | Block | LQHP | 0.2 | NA | 0.95 | 0.84 | 0.02 | 0.11 | 0.02 | 0.46 | 0.07 | 0.21 | 0.06 |
| 84 | 10,000 | Block | LQHPT | 0.2 | NA | 0.99 | 0.87 | 0.00 | 0.12 | 0.00 | 0.88 | 0.02 | 0.71 | 0.04 |
| 84 | 10,000 | Block | H | 0.3 | NA | 0.95 | 0.85 | 0.02 | 0.10 | 0.03 | 0.46 | 0.07 | 0.18 | 0.04 |
| 84 | 10,000 | Block | LQH | 0.3 | NA | 0.95 | 0.85 | 0.02 | 0.10 | 0.03 | 0.46 | 0.07 | 0.18 | 0.04 |
| 84 | 10,000 | Block | LQHP | 0.3 | NA | 0.95 | 0.85 | 0.02 | 0.10 | 0.03 | 0.48 | 0.08 | 0.19 | 0.05 |
| 84 | 10,000 | Block | LQHPT | 0.3 | NA | 0.98 | 0.88 | 0.00 | 0.10 | 0.00 | 0.79 | 0.02 | 0.35 | 0.06 |
| 84 | 10,000 | Block | H | 0.4 | NA | 0.94 | 0.85 | 0.02 | 0.09 | 0.02 | 0.38 | 0.08 | 0.15 | 0.03 |
| 84 | 10,000 | Block | LQH | 0.4 | NA | 0.94 | 0.86 | 0.02 | 0.09 | 0.02 | 0.36 | 0.07 | 0.13 | 0.02 |
| 84 | 10,000 | Block | LQHP | 0.4 | NA | 0.94 | 0.85 | 0.02 | 0.09 | 0.02 | 0.37 | 0.07 | 0.15 | 0.03 |
| 84 | 10,000 | Block | LQHPT | 0.4 | NA | 0.98 | 0.89 | 0.00 | 0.08 | 0.00 | 0.67 | 0.01 | 0.14 | 0.03 |
| 84 | 10,000 | Block | H | 0.5 | NA | 0.94 | 0.86 | 0.02 | 0.08 | 0.02 | 0.31 | 0.08 | 0.10 | 0.01 |
| 84 | 10,000 | Block | LQH | 0.5 | NA | 0.94 | 0.86 | 0.01 | 0.07 | 0.02 | 0.30 | 0.08 | 0.08 | 0.01 |
| 84 | 10,000 | Block | LQHP | 0.5 | NA | 0.94 | 0.86 | 0.01 | 0.08 | 0.02 | 0.30 | 0.08 | 0.10 | 0.01 |
| 84 | 10,000 | Block | LQHPT | 0.5 | NA | 0.97 | 0.89 | 0.01 | 0.07 | 0.01 | 0.42 | 0.07 | 0.11 | 0.01 |
| 84 | 10,000 | Block | H | 0.6 | NA | 0.93 | 0.87 | 0.01 | 0.07 | 0.02 | 0.21 | 0.06 | 0.06 | 0.01 |
| 84 | 10,000 | Block | LQH | 0.6 | NA | 0.93 | 0.87 | 0.01 | 0.06 | 0.01 | 0.21 | 0.06 | 0.05 | 0.00 |
| 84 | 10,000 | Block | LQHP | 0.6 | NA | 0.93 | 0.87 | 0.01 | 0.06 | 0.02 | 0.21 | 0.06 | 0.05 | 0.00 |
| 84 | 10,000 | Block | LQHPT | 0.6 | NA | 0.97 | 0.89 | 0.01 | 0.07 | 0.01 | 0.35 | 0.08 | 0.08 | 0.01 |
| 84 | 10,000 | Block | H | 0.7 | NA | 0.93 | 0.87 | 0.01 | 0.06 | 0.02 | 0.18 | 0.06 | 0.02 | 0.00 |
| 84 | 10,000 | Block | LQH | 0.7 | NA | 0.93 | 0.88 | 0.01 | 0.05 | 0.01 | 0.19 | 0.05 | 0.02 | 0.00 |
| 84 | 10,000 | Block | LQHP | 0.7 | NA | 0.93 | 0.88 | 0.01 | 0.05 | 0.02 | 0.19 | 0.05 | 0.02 | 0.00 |
| 84 | 10,000 | Block | H | 0.8 | NA | 0.92 | 0.88 | 0.01 | 0.05 | 0.01 | 0.15 | 0.05 | 0.02 | 0.00 |
| 84 | 10,000 | Block | LQH | 0.8 | NA | 0.92 | 0.88 | 0.01 | 0.05 | 0.01 | 0.15 | 0.05 | 0.01 | 0.00 |
| 84 | 10,000 | Block | LQHP | 0.8 | NA | 0.92 | 0.88 | 0.01 | 0.05 | 0.01 | 0.15 | 0.05 | 0.02 | 0.00 |
| 84 | 10,000 | Checkerboar1 | LQ | 0.9 | 0.00 | 0.89 | 0.85 | 0.00 | 0.02 | 0.00 | 0.19 | 0.00 | 0.02 | 0.00 |
| 84 | 10,000 | Checkerboar1 | LQ | 0.1 | 0.81 | 0.90 | 0.88 | 0.00 | 0.03 | 0.00 | 0.15 | 0.00 | 0.04 | 0.00 |
| 84 | 10,000 | Checkerboar1 | LQ | 0.2 | 0.81 | 0.90 | 0.88 | 0.00 | 0.03 | 0.00 | 0.18 | 0.00 | 0.04 | 0.00 |
| 84 | 10,000 | Checkerboar1 | LQ | 0.3 | 0.81 | 0.90 | 0.87 | 0.00 | 0.03 | 0.00 | 0.20 | 0.00 | 0.04 | 0.00 |
| 84 | 10,000 | Checkerboar1 | LQ | 0.4 | 1.18 | 0.90 | 0.87 | 0.00 | 0.03 | 0.00 | 0.18 | 0.00 | 0.05 | 0.00 |
| 84 | 10,000 | Checkerboar1 | LQ | 0.7 | 1.23 | 0.89 | 0.86 | 0.00 | 0.02 | 0.00 | 0.20 | 0.00 | 0.02 | 0.00 |
| 84 | 10,000 | Checkerboar1 | LQ | 0.6 | 1.71 | 0.89 | 0.86 | 0.00 | 0.03 | 0.00 | 0.19 | 0.00 | 0.05 | 0.00 |
| 84 | 10,000 | Checkerboar1 | LQ | 0.5 | 2.21 | 0.90 | 0.86 | 0.00 | 0.03 | 0.00 | 0.17 | 0.00 | 0.02 | 0.00 |
| 84 | 10,000 | Checkerboar1 | LQ | 0.8 | 2.71 | 0.89 | 0.86 | 0.00 | 0.02 | 0.00 | 0.20 | 0.00 | 0.02 | 0.00 |
| 84 | 10,000 | Checkerboar1 | LQ | 1 | 5.45 | 0.89 | 0.85 | 0.00 | 0.02 | 0.00 | 0.19 | 0.00 | 0.02 | 0.00 |
| 84 | 10,000 | Checkerboar1 | LQ | 1.2 | 7.55 | 0.89 | 0.85 | 0.00 | 0.02 | 0.00 | 0.14 | 0.00 | 0.02 | 0.00 |
| 84 | 10,000 | Checkerboar1 | LQ | 1.4 | 8.01 | 0.88 | 0.84 | 0.00 | 0.02 | 0.00 | 0.14 | 0.00 | 0.02 | 0.00 |
| 84 | 10,000 | Checkerboar1 | LQ | 1.1 | 14.37 | 0.89 | 0.85 | 0.00 | 0.02 | 0.00 | 0.15 | 0.00 | 0.02 | 0.00 |
| 84 | 10,000 | Checkerboar1 | LQ | 1.3 | 14.45 | 0.89 | 0.84 | 0.00 | 0.02 | 0.00 | 0.14 | 0.00 | 0.02 | 0.00 |
| 84 | 10,000 | Checkerboar1 | LQ | 1.7 | 16.73 | 0.88 | 0.83 | 0.00 | 0.02 | 0.00 | 0.14 | 0.00 | 0.02 | 0.00 |
| 84 | 10,000 | Checkerboar1 | LQ | 1.6 | 17.28 | 0.88 | 0.83 | 0.00 | 0.02 | 0.00 | 0.14 | 0.00 | 0.02 | 0.00 |
| 84 | 10,000 | Checkerboar1 | LQ | 1.8 | 17.71 | 0.88 | 0.82 | 0.00 | 0.02 | 0.00 | 0.14 | 0.00 | 0.02 | 0.00 |
| 84 | 10,000 | Checkerboar1 | LQ | 1.5 | 17.73 | 0.88 | 0.83 | 0.00 | 0.02 | 0.00 | 0.14 | 0.00 | 0.02 | 0.00 |
| 84 | 10,000 | Checkerboar1 | LQ | 1.9 | 19.09 | 0.87 | 0.82 | 0.00 | 0.02 | 0.00 | 0.12 | 0.00 | 0.02 | 0.00 |
| 84 | 10,000 | Checkerboar1 | LQ | 2 | 21.46 | 0.87 | 0.82 | 0.00 | 0.02 | 0.00 | 0.12 | 0.00 | 0.02 | 0.00 |
| 84 | 10,000 | Checkerboar1 | LQH | 1.7 | 54.05 | 0.91 | 0.88 | 0.00 | 0.03 | 0.00 | 0.23 | 0.00 | 0.10 | 0.02 |
| 84 | 10,000 | Checkerboar1 | LQH | 2 | 54.83 | 0.91 | 0.88 | 0.00 | 0.03 | 0.00 | 0.23 | 0.00 | 0.05 | 0.00 |
| 84 | 10,000 | Checkerboar1 | LQHPT | 1.6 | 55.64 | 0.93 | 0.88 | 0.00 | 0.04 | 0.00 | 0.24 | 0.01 | 0.10 | 0.02 |
| 84 | 10,000 | Checkerboar1 | LQH | 1.9 | 57.76 | 0.91 | 0.88 | 0.00 | 0.03 | 0.00 | 0.24 | 0.00 | 0.07 | 0.01 |
| 84 | 10,000 | Checkerboar1 | LQHP | 1.8 | 61.96 | 0.91 | 0.88 | 0.00 | 0.04 | 0.00 | 0.23 | 0.00 | 0.10 | 0.02 |
| 84 | 10,000 | Checkerboar1 | H | 1.7 | 63.46 | 0.91 | 0.88 | 0.00 | 0.04 | 0.00 | 0.24 | 0.00 | 0.08 | 0.01 |
| 84 | 10,000 | Checkerboar1 | LQHP | 1.7 | 66.63 | 0.91 | 0.88 | 0.00 | 0.04 | 0.00 | 0.24 | 0.00 | 0.08 | 0.01 |
| 84 | 10,000 | Checkerboar1 | H | 1.9 | 66.83 | 0.91 | 0.88 | 0.00 | 0.04 | 0.00 | 0.23 | 0.00 | 0.10 | 0.02 |
| 84 | 10,000 | Checkerboar1 | LQHPT | 1.7 | 67.39 | 0.93 | 0.88 | 0.00 | 0.04 | 0.00 | 0.24 | 0.01 | 0.10 | 0.02 |
| 84 | 10,000 | Checkerboar1 | LQH | 1.4 | 67.78 | 0.91 | 0.88 | 0.00 | 0.04 | 0.00 | 0.23 | 0.00 | 0.10 | 0.02 |
| 84 | 10,000 | Checkerboar1 | H | 2 | 68.58 | 0.91 | 0.88 | 0.00 | 0.04 | 0.00 | 0.24 | 0.00 | 0.10 | 0.02 |
| 84 | 10,000 | Checkerboar1 | LQH | 1.5 | 69.54 | 0.91 | 0.88 | 0.00 | 0.04 | 0.00 | 0.23 | 0.00 | 0.11 | 0.02 |
| 84 | 10,000 | Checkerboar1 | H | 1.8 | 71.51 | 0.91 | 0.88 | 0.00 | 0.04 | 0.00 | 0.23 | 0.00 | 0.08 | 0.01 |
| 84 | 10,000 | Checkerboar1 | LQH | 1.6 | 71.62 | 0.91 | 0.88 | 0.00 | 0.03 | 0.00 | 0.23 | 0.00 | 0.10 | 0.02 |
| 84 | 10,000 | Checkerboar1 | LQHPT | 1.9 | 75.76 | 0.93 | 0.88 | 0.00 | 0.04 | 0.00 | 0.21 | 0.00 | 0.11 | 0.02 |
| 84 | 10,000 | Checkerboar1 | LQHPT | 1.2 | 76.43 | 0.94 | 0.89 | 0.00 | 0.05 | 0.00 | 0.24 | 0.01 | 0.11 | 0.02 |
| 84 | 10,000 | Checkerboar1 | LQHPT | 2 | 80.36 | 0.92 | 0.88 | 0.00 | 0.04 | 0.00 | 0.23 | 0.01 | 0.11 | 0.02 |
| 84 | 10,000 | Checkerboar1 | H | 1.5 | 80.56 | 0.91 | 0.88 | 0.00 | 0.04 | 0.00 | 0.24 | 0.00 | 0.10 | 0.02 |
| 84 | 10,000 | Checkerboar1 | H | 1.6 | 82.05 | 0.91 | 0.88 | 0.00 | 0.04 | 0.00 | 0.24 | 0.00 | 0.10 | 0.02 |
| 84 | 10,000 | Checkerboar1 | LQHP | 2 | 84.64 | 0.91 | 0.88 | 0.00 | 0.04 | 0.00 | 0.24 | 0.00 | 0.10 | 0.02 |
| 84 | 10,000 | Checkerboar1 | LQHPT | 1.3 | 85.66 | 0.94 | 0.88 | 0.00 | 0.05 | 0.00 | 0.24 | 0.01 | 0.11 | 0.02 |
| 84 | 10,000 | Checkerboar1 | LQH | 1.8 | 88.35 | 0.91 | 0.88 | 0.00 | 0.03 | 0.00 | 0.24 | 0.00 | 0.08 | 0.01 |
| 84 | 10,000 | Checkerboar1 | LQHPT | 1.8 | 88.38 | 0.93 | 0.88 | 0.00 | 0.04 | 0.00 | 0.21 | 0.00 | 0.10 | 0.02 |
| 84 | 10,000 | Checkerboar1 | LQHP | 1.9 | 90.10 | 0.91 | 0.88 | 0.00 | 0.04 | 0.00 | 0.23 | 0.00 | 0.10 | 0.02 |
| 84 | 10,000 | Checkerboar1 | LQHP | 1.6 | 92.58 | 0.91 | 0.88 | 0.00 | 0.04 | 0.00 | 0.24 | 0.00 | 0.10 | 0.02 |
| 84 | 10,000 | Checkerboar1 | H | 1.4 | 94.25 | 0.91 | 0.88 | 0.00 | 0.04 | 0.00 | 0.25 | 0.00 | 0.10 | 0.02 |
| 84 | 10,000 | Checkerboar1 | LQHP | 1.5 | 98.82 | 0.91 | 0.88 | 0.00 | 0.04 | 0.00 | 0.24 | 0.00 | 0.10 | 0.02 |
| 84 | 10,000 | Checkerboar1 | LQHPT | 1.5 | 101.47 | 0.93 | 0.88 | 0.00 | 0.04 | 0.00 | 0.24 | 0.01 | 0.10 | 0.02 |
| 84 | 10,000 | Checkerboar1 | LQHPT | 1.4 | 104.32 | 0.94 | 0.88 | 0.00 | 0.05 | 0.00 | 0.24 | 0.01 | 0.10 | 0.02 |
| 84 | 10,000 | Checkerboar1 | LQHPT | 1.1 | 110.31 | 0.95 | 0.89 | 0.00 | 0.05 | 0.00 | 0.21 | 0.02 | 0.11 | 0.02 |
| 84 | 10,000 | Checkerboar1 | LQH | 1.3 | 111.87 | 0.91 | 0.88 | 0.00 | 0.04 | 0.00 | 0.25 | 0.00 | 0.10 | 0.02 |
| 84 | 10,000 | Checkerboar1 | LQHP | 1.3 | 121.13 | 0.91 | 0.88 | 0.00 | 0.04 | 0.00 | 0.25 | 0.00 | 0.11 | 0.02 |
| 84 | 10,000 | Checkerboar1 | LQHP | 1.4 | 122.68 | 0.91 | 0.88 | 0.00 | 0.04 | 0.00 | 0.25 | 0.00 | 0.10 | 0.02 |
| 84 | 10,000 | Checkerboar1 | LQHP | 1.2 | 127.86 | 0.92 | 0.88 | 0.00 | 0.04 | 0.00 | 0.25 | 0.00 | 0.11 | 0.02 |
| 84 | 10,000 | Checkerboar1 | LQH | 1.2 | 127.86 | 0.91 | 0.88 | 0.00 | 0.04 | 0.00 | 0.25 | 0.00 | 0.10 | 0.02 |
| 84 | 10,000 | Checkerboar1 | H | 1.2 | 135.98 | 0.91 | 0.88 | 0.00 | 0.04 | 0.00 | 0.25 | 0.00 | 0.11 | 0.02 |
| 84 | 10,000 | Checkerboar1 | LQHPT | 1 | 139.22 | 0.95 | 0.89 | 0.00 | 0.05 | 0.00 | 0.23 | 0.02 | 0.11 | 0.02 |
| 84 | 10,000 | Checkerboar1 | L | 0.1 | 146.15 | 0.72 | 0.70 | 0.00 | 0.03 | 0.00 | 0.18 | 0.01 | 0.01 | 0.00 |
| 84 | 10,000 | Checkerboar1 | L | 0.2 | 146.15 | 0.72 | 0.70 | 0.00 | 0.03 | 0.00 | 0.18 | 0.01 | 0.01 | 0.00 |
| 84 | 10,000 | Checkerboar1 | L | 0.3 | 146.15 | 0.72 | 0.70 | 0.00 | 0.03 | 0.00 | 0.17 | 0.01 | 0.04 | 0.00 |
| 84 | 10,000 | Checkerboar1 | L | 0.4 | 146.15 | 0.72 | 0.70 | 0.00 | 0.03 | 0.00 | 0.14 | 0.00 | 0.05 | 0.00 |
| 84 | 10,000 | Checkerboar1 | L | 0.5 | 146.15 | 0.72 | 0.69 | 0.00 | 0.03 | 0.00 | 0.15 | 0.01 | 0.05 | 0.00 |
| 84 | 10,000 | Checkerboar1 | L | 0.6 | 146.39 | 0.72 | 0.69 | 0.00 | 0.03 | 0.00 | 0.14 | 0.01 | 0.06 | 0.01 |
| 84 | 10,000 | Checkerboar1 | L | 0.7 | 146.73 | 0.72 | 0.68 | 0.00 | 0.03 | 0.00 | 0.13 | 0.01 | 0.08 | 0.00 |
| 84 | 10,000 | Checkerboar1 | L | 0.8 | 147.14 | 0.72 | 0.67 | 0.00 | 0.03 | 0.00 | 0.12 | 0.00 | 0.10 | 0.00 |
| 84 | 10,000 | Checkerboar1 | L | 0.9 | 147.69 | 0.72 | 0.66 | 0.00 | 0.03 | 0.00 | 0.12 | 0.00 | 0.07 | 0.00 |
| 84 | 10,000 | Checkerboar1 | L | 1 | 148.08 | 0.72 | 0.66 | 0.00 | 0.04 | 0.00 | 0.12 | 0.00 | 0.05 | 0.00 |
| 84 | 10,000 | Checkerboar1 | L | 1.1 | 148.87 | 0.72 | 0.66 | 0.00 | 0.04 | 0.00 | 0.12 | 0.00 | 0.04 | 0.00 |
| 84 | 10,000 | Checkerboar1 | L | 1.2 | 149.56 | 0.72 | 0.65 | 0.00 | 0.04 | 0.00 | 0.12 | 0.00 | 0.02 | 0.00 |
| 84 | 10,000 | Checkerboar1 | L | 1.3 | 150.26 | 0.72 | 0.65 | 0.00 | 0.04 | 0.00 | 0.12 | 0.00 | 0.02 | 0.00 |
| 84 | 10,000 | Checkerboar1 | L | 1.4 | 151.06 | 0.72 | 0.65 | 0.00 | 0.04 | 0.00 | 0.12 | 0.00 | 0.02 | 0.00 |
| 84 | 10,000 | Checkerboar1 | L | 1.5 | 151.94 | 0.71 | 0.65 | 0.00 | 0.04 | 0.00 | 0.12 | 0.00 | 0.02 | 0.00 |
| 84 | 10,000 | Checkerboar1 | L | 1.6 | 152.98 | 0.71 | 0.65 | 0.00 | 0.04 | 0.00 | 0.12 | 0.00 | 0.02 | 0.00 |
| 84 | 10,000 | Checkerboar1 | L | 1.7 | 153.44 | 0.71 | 0.65 | 0.00 | 0.04 | 0.00 | 0.12 | 0.00 | 0.02 | 0.00 |
| 84 | 10,000 | Checkerboar1 | L | 1.8 | 154.52 | 0.71 | 0.64 | 0.00 | 0.04 | 0.00 | 0.12 | 0.00 | 0.02 | 0.00 |
| 84 | 10,000 | Checkerboar1 | L | 1.9 | 155.93 | 0.71 | 0.64 | 0.00 | 0.04 | 0.00 | 0.12 | 0.00 | 0.02 | 0.00 |
| 84 | 10,000 | Checkerboar1 | L | 2 | 157.15 | 0.71 | 0.64 | 0.00 | 0.04 | 0.00 | 0.12 | 0.00 | 0.02 | 0.00 |
| 84 | 10,000 | Checkerboar1 | H | 1.3 | 170.85 | 0.91 | 0.88 | 0.00 | 0.04 | 0.00 | 0.25 | 0.00 | 0.11 | 0.02 |
| 84 | 10,000 | Checkerboar1 | H | 1.1 | 178.35 | 0.92 | 0.88 | 0.00 | 0.04 | 0.00 | 0.25 | 0.00 | 0.11 | 0.02 |
| 84 | 10,000 | Checkerboar1 | LQHP | 1.1 | 207.17 | 0.92 | 0.88 | 0.00 | 0.04 | 0.00 | 0.25 | 0.00 | 0.11 | 0.02 |
| 84 | 10,000 | Checkerboar1 | LQHPT | 0.9 | 325.76 | 0.95 | 0.89 | 0.00 | 0.06 | 0.00 | 0.25 | 0.01 | 0.12 | 0.03 |
| 84 | 10,000 | Checkerboar1 | LQH | 1.1 | 391.36 | 0.92 | 0.88 | 0.00 | 0.04 | 0.00 | 0.25 | 0.00 | 0.11 | 0.02 |
| 84 | 10,000 | Checkerboar1 | LQHP | 1 | 484.96 | 0.92 | 0.88 | 0.00 | 0.04 | 0.00 | 0.23 | 0.01 | 0.11 | 0.02 |
| 84 | 10,000 | Checkerboar1 | H | 1 | 571.63 | 0.92 | 0.88 | 0.00 | 0.04 | 0.00 | 0.23 | 0.01 | 0.11 | 0.02 |
| 84 | 10,000 | Checkerboar1 | LQH | 1 | 623.20 | 0.92 | 0.88 | 0.00 | 0.04 | 0.00 | 0.23 | 0.01 | 0.11 | 0.02 |
| 84 | 10,000 | Checkerboar1 | H | 0.9 | 740.69 | 0.92 | 0.88 | 0.00 | 0.04 | 0.00 | 0.23 | 0.01 | 0.11 | 0.02 |
| 84 | 10,000 | Checkerboar1 | LQHPT | 0.8 | 807.34 | 0.96 | 0.89 | 0.00 | 0.06 | 0.00 | 0.27 | 0.01 | 0.12 | 0.03 |
| 84 | 10,000 | Checkerboar1 | LQHP | 0.9 | 1138.93 | 0.92 | 0.88 | 0.00 | 0.04 | 0.00 | 0.23 | 0.01 | 0.11 | 0.02 |
| 84 | 10,000 | Checkerboar1 | LQH | 0.9 | 2068.66 | 0.92 | 0.88 | 0.00 | 0.04 | 0.00 | 0.23 | 0.01 | 0.11 | 0.02 |
| 84 | 10,000 | Checkerboar1 | LQHPT | 0.7 | 6599.86 | 0.96 | 0.89 | 0.00 | 0.07 | 0.00 | 0.31 | 0.00 | 0.13 | 0.02 |
| 84 | 10,000 | Checkerboar1 | H | 0.1 | NA | 0.96 | 0.89 | 0.00 | 0.08 | 0.00 | 0.38 | 0.00 | 0.21 | 0.00 |
| 84 | 10,000 | Checkerboar1 | LQH | 0.1 | NA | 0.96 | 0.89 | 0.00 | 0.08 | 0.00 | 0.37 | 0.01 | 0.21 | 0.00 |
| 84 | 10,000 | Checkerboar1 | LQHP | 0.1 | NA | 0.96 | 0.89 | 0.00 | 0.08 | 0.00 | 0.37 | 0.01 | 0.20 | 0.00 |
| 84 | 10,000 | Checkerboar1 | LQHPT | 0.1 | NA | 0.99 | 0.87 | 0.00 | 0.12 | 0.00 | 0.87 | 0.00 | 0.71 | 0.00 |
| 84 | 10,000 | Checkerboar1 | H | 0.2 | NA | 0.95 | 0.89 | 0.00 | 0.07 | 0.00 | 0.36 | 0.04 | 0.17 | 0.00 |
| 84 | 10,000 | Checkerboar1 | LQH | 0.2 | NA | 0.95 | 0.89 | 0.00 | 0.07 | 0.00 | 0.35 | 0.03 | 0.18 | 0.00 |
| 84 | 10,000 | Checkerboar1 | LQHP | 0.2 | NA | 0.95 | 0.89 | 0.00 | 0.07 | 0.00 | 0.36 | 0.04 | 0.18 | 0.00 |
| 84 | 10,000 | Checkerboar1 | LQHPT | 0.2 | NA | 0.99 | 0.88 | 0.00 | 0.11 | 0.00 | 0.76 | 0.01 | 0.52 | 0.02 |
| 84 | 10,000 | Checkerboar1 | H | 0.3 | NA | 0.95 | 0.89 | 0.00 | 0.06 | 0.00 | 0.29 | 0.02 | 0.15 | 0.01 |
| 84 | 10,000 | Checkerboar1 | LQH | 0.3 | NA | 0.95 | 0.89 | 0.00 | 0.06 | 0.00 | 0.29 | 0.02 | 0.15 | 0.01 |
| 84 | 10,000 | Checkerboar1 | LQHP | 0.3 | NA | 0.95 | 0.89 | 0.00 | 0.06 | 0.00 | 0.29 | 0.02 | 0.15 | 0.01 |
| 84 | 10,000 | Checkerboar1 | LQHPT | 0.3 | NA | 0.98 | 0.89 | 0.00 | 0.10 | 0.00 | 0.57 | 0.03 | 0.39 | 0.00 |
| 84 | 10,000 | Checkerboar1 | H | 0.4 | NA | 0.94 | 0.89 | 0.00 | 0.06 | 0.00 | 0.26 | 0.02 | 0.14 | 0.00 |
| 84 | 10,000 | Checkerboar1 | LQH | 0.4 | NA | 0.94 | 0.89 | 0.00 | 0.06 | 0.00 | 0.26 | 0.02 | 0.14 | 0.00 |
| 84 | 10,000 | Checkerboar1 | LQHP | 0.4 | NA | 0.94 | 0.89 | 0.00 | 0.06 | 0.00 | 0.27 | 0.01 | 0.14 | 0.00 |
| 84 | 10,000 | Checkerboar1 | LQHPT | 0.4 | NA | 0.98 | 0.89 | 0.00 | 0.09 | 0.00 | 0.48 | 0.01 | 0.20 | 0.01 |
| 84 | 10,000 | Checkerboar1 | H | 0.5 | NA | 0.94 | 0.88 | 0.00 | 0.06 | 0.00 | 0.23 | 0.01 | 0.13 | 0.01 |
| 84 | 10,000 | Checkerboar1 | LQH | 0.5 | NA | 0.94 | 0.88 | 0.00 | 0.06 | 0.00 | 0.24 | 0.01 | 0.13 | 0.01 |
| 84 | 10,000 | Checkerboar1 | LQHP | 0.5 | NA | 0.94 | 0.88 | 0.00 | 0.06 | 0.00 | 0.24 | 0.01 | 0.13 | 0.01 |
| 84 | 10,000 | Checkerboar1 | LQHPT | 0.5 | NA | 0.97 | 0.89 | 0.00 | 0.08 | 0.00 | 0.33 | 0.00 | 0.20 | 0.01 |
| 84 | 10,000 | Checkerboar1 | H | 0.6 | NA | 0.93 | 0.88 | 0.00 | 0.05 | 0.00 | 0.21 | 0.02 | 0.10 | 0.01 |
| 84 | 10,000 | Checkerboar1 | LQH | 0.6 | NA | 0.93 | 0.88 | 0.00 | 0.05 | 0.00 | 0.20 | 0.01 | 0.10 | 0.01 |
| 84 | 10,000 | Checkerboar1 | LQHP | 0.6 | NA | 0.93 | 0.88 | 0.00 | 0.05 | 0.00 | 0.23 | 0.01 | 0.10 | 0.01 |
| 84 | 10,000 | Checkerboar1 | LQHPT | 0.6 | NA | 0.97 | 0.89 | 0.00 | 0.08 | 0.00 | 0.31 | 0.00 | 0.21 | 0.02 |
| 84 | 10,000 | Checkerboar1 | H | 0.7 | NA | 0.93 | 0.88 | 0.00 | 0.05 | 0.00 | 0.21 | 0.02 | 0.08 | 0.01 |
| 84 | 10,000 | Checkerboar1 | LQH | 0.7 | NA | 0.93 | 0.88 | 0.00 | 0.05 | 0.00 | 0.23 | 0.01 | 0.08 | 0.01 |
| 84 | 10,000 | Checkerboar1 | LQHP | 0.7 | NA | 0.93 | 0.88 | 0.00 | 0.05 | 0.00 | 0.23 | 0.01 | 0.08 | 0.01 |
| 84 | 10,000 | Checkerboar1 | H | 0.8 | NA | 0.92 | 0.88 | 0.00 | 0.04 | 0.00 | 0.24 | 0.01 | 0.10 | 0.02 |
| 84 | 10,000 | Checkerboar1 | LQH | 0.8 | NA | 0.92 | 0.88 | 0.00 | 0.04 | 0.00 | 0.21 | 0.01 | 0.08 | 0.01 |
| 84 | 10,000 | Checkerboar1 | LQHP | 0.8 | NA | 0.92 | 0.88 | 0.00 | 0.05 | 0.00 | 0.24 | 0.01 | 0.10 | 0.02 |
